# CD44 knockdown alters miRNA expression and their target genes in colon cancer

**DOI:** 10.3389/fimmu.2025.1552665

**Published:** 2025-05-14

**Authors:** Diana Maltseva, Anton Zhiyanov, Tobias Lange, Alexander Tonevitsky

**Affiliations:** ^1^ Faculty of Biology and Biotechnology, National Research University Higher School of Economics, Moscow, Russia; ^2^ Institute of Anatomy, University of Lübeck, Lübeck, Germany; ^3^ Shemyakin-Ovchinnikov Institute of Bioorganic Chemistry of the Russian Academy of Sciences, Moscow, Russia

**Keywords:** CD44, Let-7 miRNAs, miR-203a-3p, miR-101-3p, miR-125a-5p, miR-185-5p, miRNA 5’-isoform, colon cancer metastasis

## Abstract

**Introduction:**

Metastasis formation poses a significant challenge to oncologists, as it severely limits the survival of colorectal cancer (CRC) patients. Recently, we demonstrated that CD44 promotes spontaneous distant metastasis in a CRC xenograft model. The depletion of CD44 was associated with reduction in hypoxia, EMT, as well as improved mitochondrial metabolism in primary tumor. Collectively, these effects decreased the metastatic potential of the CRC xenograft tumors under investigation. In this study we explore the molecular mechanisms by which CD44 knockdown (kd) leads to such substantial changes of tumor properties.

**Methods:**

Using miRNA-Seq data combined with bioinformatic analysis, we investigated the role of miRNA expression changes in the metastasis prevention observed with CD44 kd.

**Results:**

Among the differentially expressed miRNAs, three members of Let-7 family (let-7a-5p, let-7b-5p, and let-7c-5p), two isoforms of miR-203a (canonical miR-203a-3p and its +1 5’-isoform), miR-101-3p, miR-200b-3p|+1 5’-isoform, miR-125a-5p, and miR-185-5p were identified as potentially involved in regulating CD44-mediated metastasis. Gene set analysis of differentially expressed mRNA targets of these miRNAs, along with an examination of key regulators driving the observed changes in both mRNA and miRNA expression profiles, suggests that the CD44-STAT3-Let-7 miRNA axis as one of the most relevant in regulation of colon cancer metastasis via the CD44 receptor.

**Discussion:**

Our findings suggest a regulatory relationship between CD44, Let-7 miRNAs, and STAT3 in HT-29 tumors. Additionally, we propose the potential involvement of both isoforms of miR-203a (canonical and its +1 5’-isoform) in this regulatory network and suggest a role for miR-101-3p and miR-125a-5p in metastasis regulation through CD44 kd.

## 1 Introduction

Despite significant advancements in colorectal cancer (CRC) treatment in high-income countries, CRC remains one of the most prevalent malignant cancers worldwide (1, 2). While the overall 5-year survival rate for CRC patients is approximately 64%, prognosis largely depends on the presence of metastases. For patients with metastatic CRC, the survival rate drops to less than 15% (2), highlighting metastasis as a major clinical challenge (3, 4).

Recently we demonstrated that cluster of differentiation 44 (CD44), a multifunctional transmembrane glycoprotein, promotes spontaneous distant metastasis in a CRC xenograft model (5). Specifically, CD44 depletion reduces the intermediate epithelial/mesenchymal phenotype and stem cell-like properties in HT-29 xenografts while enhancing mitochondrial content, oxidative phosphorylation, and angiogenesis in CD44 knockdown (kd) xenograft tumors. In this study, we investigate the molecular mechanisms underlying these substantial changes in tumor properties following CD44 kd. Previous studies have shown that CD44 interaction with matrix hyaluronan (HA) activates microRNAs (miRNAs) signaling pathways associated with tumor progression, invasion, and chemoresistance (6). Therefore, miRNAs may play a role in the molecular mechanisms triggered by CD44 knockdown.

MiRNAs are small noncoding RNAs that regulate mRNA stability or translation by complementary binding of their seed region (nucleotides 2–7/8 at the 5′ end of a miRNA) to the 3′ untranslated region (3′-UTR) of target mRNAs (7). In various cancers, including CRC, miRNAs often exhibit dysregulated expression, acting as either oncogenes or tumor suppressors that influence cancer initiation and progression (8–11).

Additionally, the processing of miRNA precursors (pri-miRNAs and pre-miRNAs) by RNase-III family nucleases, Drosha and Dicer, can generate not only canonical miRNAs but also isoforms due to shifts in the cleavage position, primarily by Dicer (12–14). If this shift occurs at the 5’-end of a miRNA, it alters the seed region sequence, resulting in 5’-isoforms with potentially modified mRNA target profile. Emerging evidence suggests that both canonical miRNAs and their 5’-isoforms play significant roles in regulating cellular biological processes (15–17). Therefore, in this study, we investigate how changes in the expression of both canonical miRNAs and their 5’-isoforms contribute to the suppression of metastasis following CD44 kd in CRC tumors.

## 2 Materials and methods

### 2.1 Xenograft tumor samples

Xenograft tumor samples were generated in our previous study (5). Briefly, CD44 was knocked down (CD44 kd) in HT-29 cells using a pan-CD44 shRNA construct (stable reduced total CD44 expression by > 95%). HT-29 cells transduced by the same lentiviral vector containing a sequence against firefly luciferase (Luc) was used as a control cell line. Pathogen-free Balb/c severe combined immunodeficient (scid) mice (n = 12) aged 9–14 weeks with a weight of 25–30 g at the beginning of the experiment were inoculated subcutaneously above the right scapula with 1 × 10^6^ HT-29 Luc or HT-29 CD44 kd cells in a medium without supplements. All of mice developed s. c. primary tumor (PT). All mice had to be euthanized due to tumor ulceration except two animals of the CD44 kd group. Of these two, one tumor reached the endpoint tumor weight (10% of the body weight), and the other animal had not reached any termination criteria when the experiment was stopped after 71 days. PTs were dissected and cut into several pieces for histology, proteomics, kinomic profiling, RNA sequencing.

### 2.2 RNA extraction

Approximately 50 mg of fresh-frozen xenograft PT tissue was crushed in liquid nitrogen in the presence of QIAzol Lysis Reagent (Qiagen, Hilden, Germany). The total RNA was isolated using miRNeasy Mini Kit (Qiagen, Hilden, Germany) according to the manufacturer’s instructions. All RNA samples were treated with DNase I during the isolation procedure. The RNA yield was determined by UV absorbance using a NanoDrop 1000 spectrophotometer (Peqlab, Erlangen, Germany). The RNA quality was assessed by analyzing the ribosomal RNA integrity number (RIN) on an Agilent 2100 Bioanalyzer using the RNA 6000 Nano kit (Agilent Technologies, Palo Alto, CA, USA). The RIN values of the isolated RNA samples ranged from 6.7 to 9.6.

### 2.3 Library preparation and sequencing

Libraries for miRNA sequencing were prepared from total RNA samples using NEBNext Multiplex Small RNA Library Prep Kit for Illumina. Each sample was sequenced on the Illumina NextSeq 550 to generate single-end 50-nucleotide reads: 7 control samples (shLuc) and 8 samples with CD44 knockdown (shCD44).

Libraries for mRNA sequencing were prepared as part of our previous study, all details are described in (5).

### 2.4 RNA-seq data processing

mRNA-seq data were obtained from our previous work (5), while miRNA-seq were processed using the standard bioinformatical pipeline. Adapter sequences (AGATCGGAAGAGCACACGTCT) were removed using cutadapt v2.10 (18). The quality of RNA-seq FASTQ files was assessed by FastQC v0.11.9 (Babraham Bioinformatics, Cambridge, UK).

miRNA 5’-isoform (isomiR) read counts were produced by IsoMiRMap v1.0 (19), using miRbase v22.1 database (20) as a source of reference miRNA sequences. The annotation of isomiRs was conducted using the method proposed in (21), which builds on a modified version of the approach in (19). Specifically, the number following the initial “|” character represents the shift from the canonical 5′-end in the 5′→3′ direction. For example, miR-203a-3p|0 represents the canonical isomiR of miR-203a-3p, while miR-203a-3p|+1 denotes an isomiR shortened by one nucleotide at the 5′-end of the canonical miR-203a-3p.

Both mRNA and isomiR read counts tables were normalized for size factors using DESeq2 v 1.44.0 (22). Then, after gene length normalization, the mRNA table was transformed to fragments per kilobase of transcript per million mapped reads (FPKM) scale. Similarly, the miRNA table was transformed to per million mapped reads (RPM) scale.

Unsupervised principal component analysis (PCA) in the presence of RIN factor showed no significant difference between CD44 kd and control groups (**Supplementary Figure S1**). Moreover, applying t-test to RIN of these groups we confirm this notion (t-test p-value > 0.05). Other covariates (mice sex and age) unrelated to the sequencing were verified in our previous publication (5).

### 2.5 Differential expression analysis

Differentially expressed genes and isomiRs were identified using DESeq2 with 0.05 false discovery rate (FDR) threshold. Also, we required genes to change their expression level more than 2 times to classify them as differentially expressed.

Among the differentially expressed isomiRs, the greatest attention was paid to the ones of significant expression, i.e, with RPM greater than 100. Also, the 10.000 most expressed protein coding genes were selected for the analysis.

### 2.6 RT-qPCR

cDNA was synthesized using 500 ng of total RNA as a starting material and SuperScript VILO cDNA Synthesis Kit (Invitrogen, Carlsbad, USA) according to the manufacturer’s recommendations. qPCR analysis was carried out using the SYBR Green 5x qPCRmix-HS SYBR reaction mix (Evrogen, Moscow, Russia) as described in (23). Primer pairs were designed and characterized as described in (24). *PTMA*, *SF3A1*, *HPRT1*, and *MRPL19* were selected as reference genes based on the validation procedure described in (25). PCR efficiencies of the used primer sets were higher than 1.9. RNA samples were analyzed in triplicate and averaged. Ct values for target genes were normalized to the reference genes and processed based on the ΔΔCt method (26).

### 2.7 Prediction of isomiR target genes

Validated targets of differentially expressed canonical miRNA isoforms were extracted from DIANA-Tarbase v8 database (27). Predicted targets of non-canonical miRNA isoforms were obtained from TargetScan v8 conserved miRNA database (28) provided by IsomiRTar portal (21). These targets were analyzed for a seed region presence to identify more conservative isomiR-mRNA interactions.

### 2.8 Enrichment analysis

For canonical isomiRs, Fisher’s exact test was used to prove the validated target genes to be overrepresented among the differentially expressed ones. Also, the test was applied to the collection of down-regulated targets to identify enriched hallmark gene sets taken from MSigDB v3 database (29). For both procedures, we fixed a 0.05 FDR threshold to adjust for multiple testing.

Using EnrichMir tool (30) for analysis of non-canonical isomiRs, fold change distributions of the predicted targets and non-target genes were compared Kolmogorov-Smirnov test was utilized to distinguish fold change distributions of genes with and without seed regions.

### 2.9 Analysis of key transcription factors regulating differentially expressed mRNA and miRNAs

The search of possible key transcription factors regulating differentially expressed mRNA upon CD44 kd were carry out using TRRUST online tool (31). For analysis top 500 down- and up-regulated genes were selected.

Transcription factors regulating miRNAs were found based on TransmiR v3.0 database (32). TF–miRNA regulations are incorporated into TransmiR v3.0 based on ChIP-seq data.

## 3 Results

### 3.1 CD44 kd is associated with changes in isomiR expression profiles

**Figure 1:**
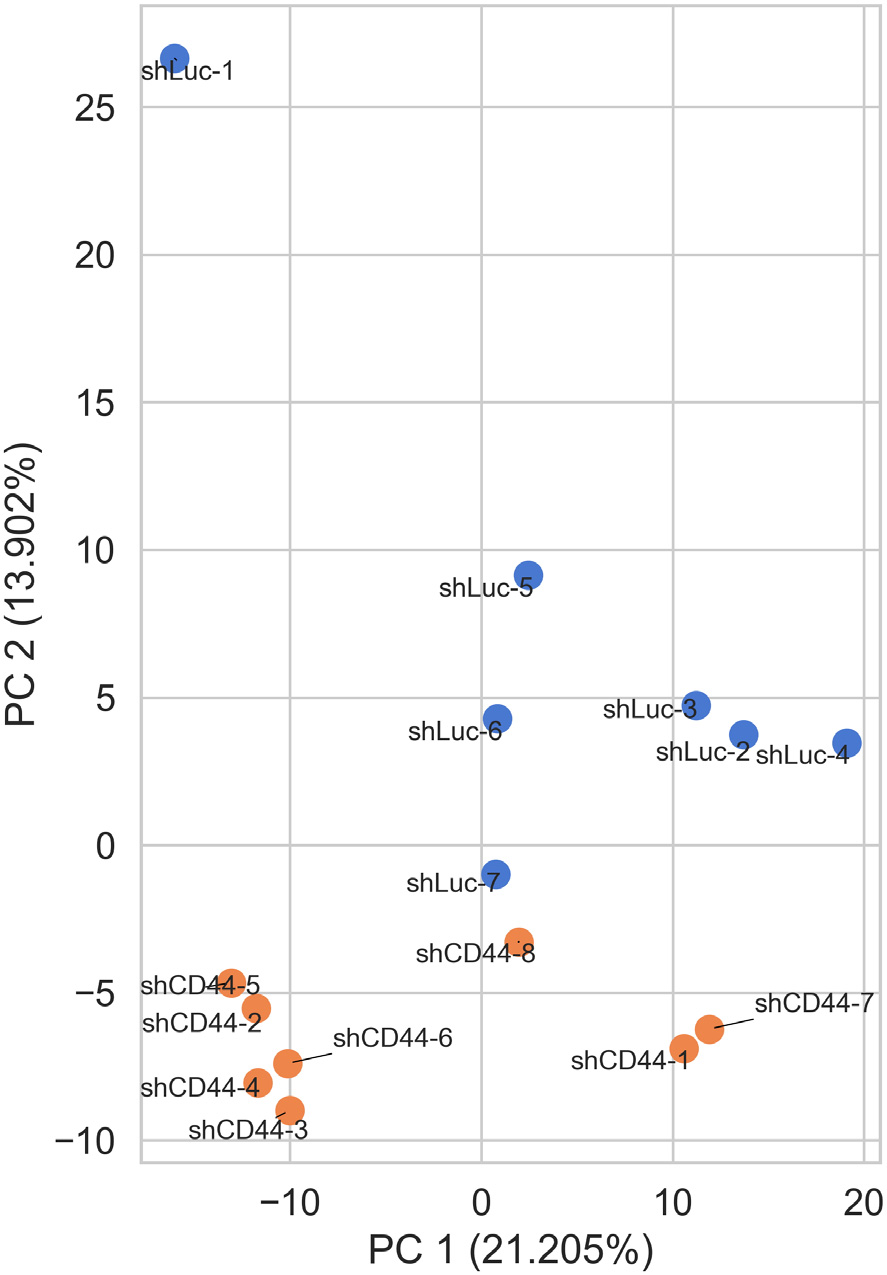
IsomiR expression analysis in HT-29 xenograft tumors. Control (shLuc) and CD44 kd (shCD44) samples grouped well by isomiR expression profiles (PCA analysis).

Growing evidence suggests that not only canonical miRNAs but also their 5’-isoforms with shifted seed regions play a role in post-transcriptional gene regulation. To explore the potential significance of this phenomenon we performed deep sequencing of HT-29 xenograft tumor samples to identify differentially expressed canonical miRNAs and their 5’-isoforms following CD44 kd. The complete set of canonical and non-canonical miRNA 5′-isoforms will be collectively referred to as isomiRs, with canonical miRNAs representing a subset of all isomiRs. Unsupervised principal component analysis (PCA) showed that the control (shLuc) and shCD44 samples grouped well by isomiR expression profiles (**Figure 1**).

Firstly, we distinguished significantly expressed isomiRs from lowly expressed ones using a threshold of 100 RPM in average expression, identifying a total of 200 isomiRs. Among these, the top 92 most highly expressed isomiRs accounted for 95% of the total isomiR expression and were selected for further analysis.

**Figure 2:**
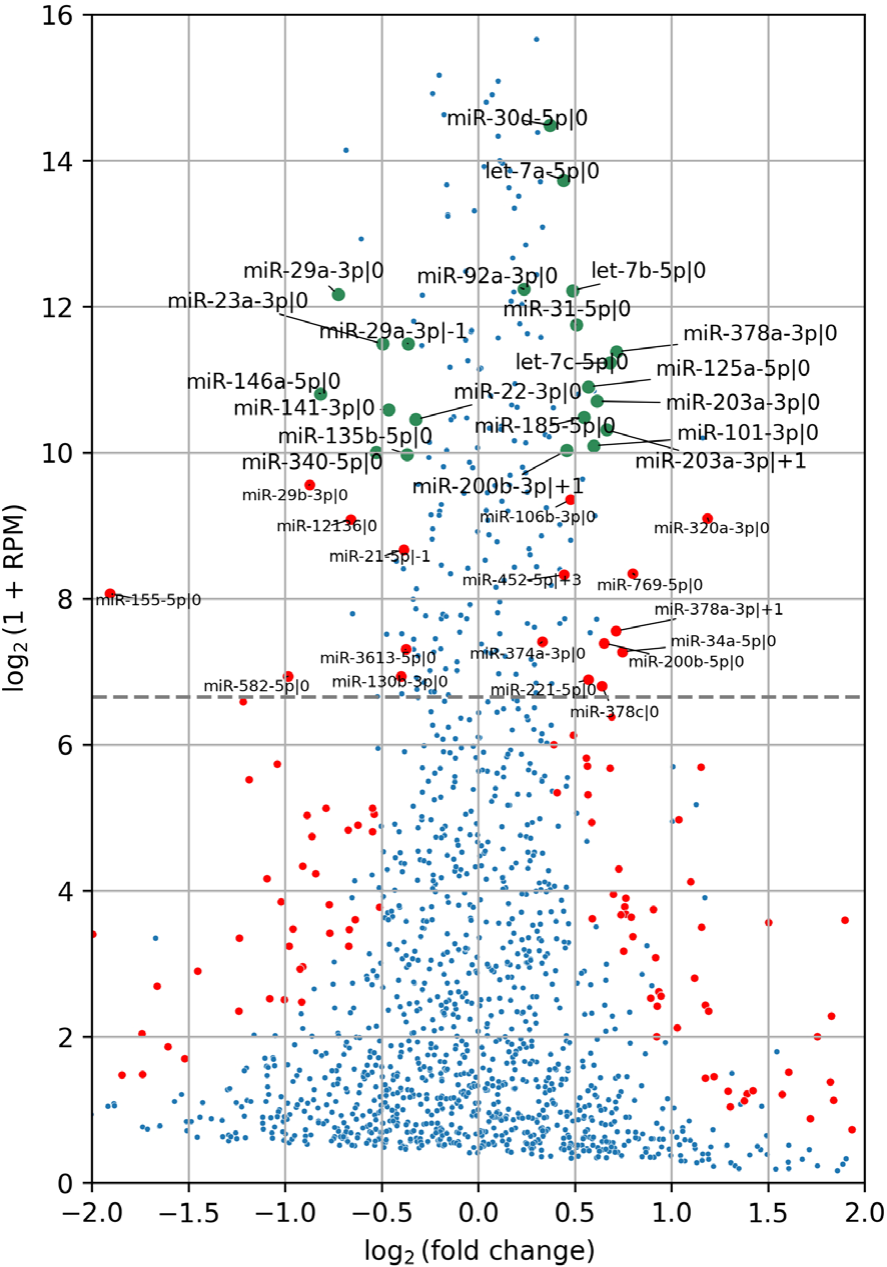
IsomiR differential expression analysis in HT-29 xenograft tumors following CD44 kd. Red and green points indicate differentially expressed isomiRs (FDR < 0.05), with green points representing the most abundant isomiRs, accounting for 95% of cumulative expression. Red points denote isomiRs with expression levels exceeding 100 RPM threshold. The grey horizontal line marks this threshold. In total, 13 highly abundant isomiRs were upregulated, while 8 were downregulated. IsomiR annotation was conducted following the methods described in (19, 21). Specifically, the number following the initial “|” character indicates the shift from the canonical 5′-end in the 5′→3′ direction. For example, miR-203a-3p|0 represents the canonical isomiR of miR-203a-3p, while miR-203a-3p|+1 denotes an isomiR shortened by one nucleotide at the 5′-end of the canonical miR-203a-3p.

Following CD44 kd, 13 of the most abundant isomiRs exhibited a statistically significant increase in expression, while 8 showed a decrease (**Figure 2**). Notably, the majority of differentially expressed isomiRs (18 of 21) were canonical, allowing us to leverage experimentally validated miRNA targets databases to analyze their regulatory activity. Consequently, we extracted the targets of these canonical isomiRs from the DIANA-Tarbase database and assessed their overrepresentation among differentially expressed protein-coding genes. For upregulated isomiRs, we analyzed the downregulated genes, and vice versa. Among the differentially expressed canonical isomiRs, only 8 of 18 showed statistically significant enrichment (Fisher’s test FDR < 0.05), and all of them were upregulated in the shCD44 group (**Table 1**). Notably, three of these isomiRs belong to the Let-7 family and share the same seed region, leading to a substantial overlap in their target genes.

**Table 1:** Enrichment analysis of validated mRNA targets of canonical isomiRs.

miRNA	odds ratio*	p-value	FDR
miR-378a-3p	2.1	< 0.001	0.001
miR-185-5p	1.8	0.002	0.007
miR-125a-5p	1.5	0.016	0.026
miR-203a-3p	1.5	0.009	0.017
let-7a-5p	1.5	0.002	0.007
let-7c-5p	1.5	0.006	0.014
let-7b-5p	1.5	0.002	0.007
miR-101-3p	1.4	0.021	0.030

*Odds ratio is equal to the ratio (td/tn)/(nd/nn), where “td” and “tn” denote the number of targets among downregulated and non-regulated genes, respectively. Similarly, “nd” and “nn” denote the number of non-target genes among the same classes.

**Figure 3:**
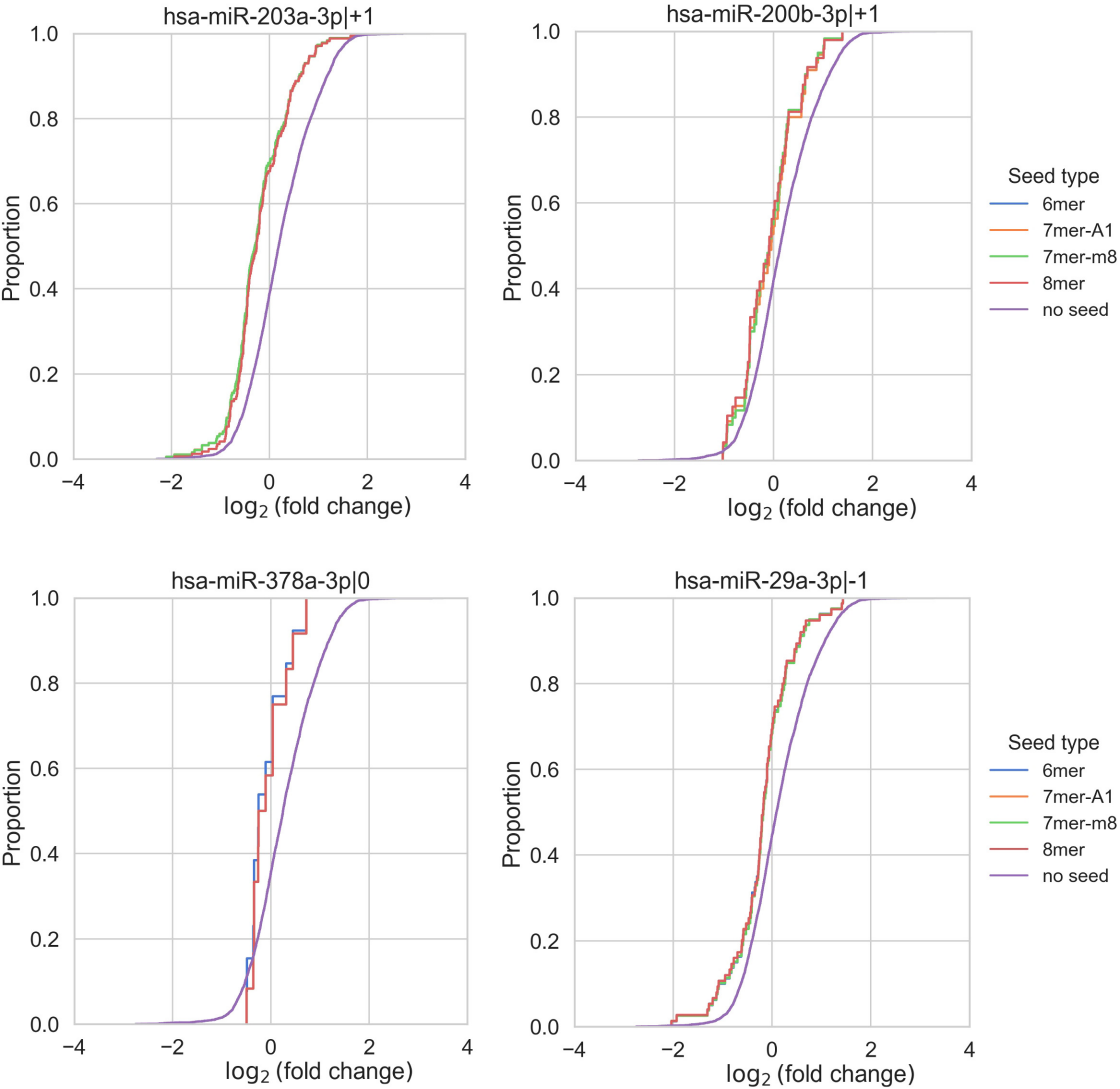
Enrichment analysis of isomiR seed regions. Enrichment analysis of isomiR seed regions in differentially expressed mRNAs revealed significant differences in the log2(fold change) distributions of genes with and without a corresponding seed (Kolmogorov-Smirnov test FDR < 0.05) for the 5’-isoforms miR-203a-3p|+1 and miR-200b-3p|+1. In contrast, for miR-378a-3p, the FDR was greater than 0.05. MiR-29a-3p|-1 exhibited a conflicting shift in fold change distribution, as its predicted targets showed decreased expression despite miR-29a-3p|-1 itself being downregulated.

Unfortunately, no comprehensive databases currently exist for experimentally validated targets of non-canonical isomiRs (5'-isoforms). Therefore, predicted targets were retrieved from the TargetScan database. It is well known that such databases have limited overlap and contain a substantial number of false positive interactions (22). To improve target specificity, we filtered the predicted targets to include only those containing seed regions. These refined targets were then used to assess the overall impact of upregulated isomiRs following CD44 kd. As shown in **Table 2** and **Supplementary Figure S2**, the predicted targets of nearly all selected canonical isomiRs were significantly overrepresented among differentially expressed mRNAs (Kolmogorov-Smirnov test FDR < 0.05), with the exception of miR-378a-3p|0 (**Table 2**, **Figure 3**). Among the 5'-isoforms, the predicted targets were significantly overrepresented for miR-203a-3p|+1 and miR-200b-3p|+1 (**Table 2**, **Figure 3**). We excluded two isomiRs from further analysis: miR-378a-3p|0, due to the lack of significant overrepresentation of targets, and miR-29a-3p|-1, because its fold change direction was inconsistent with the fold change distribution of its targets. As a result, seven canonical miRNAs and two 5’-isoforms were selected for further investigation.

**Table 2:** Enrichment analysis of the predicted mRNA targets of differentially expressed canonical and non-canonical isomiRs.

isomiR	distance*	p-value	FDR
miR-185-5p|0	0.51	< 0.001	< 0.001
miR-125a-5p|0	0.36	< 0.001	< 0.001
let-7a-5p|0	0.35	< 0.001	< 0.001
let-7b-5p|0	0.35	< 0.001	< 0.001
miR-203a-3p|+1	0.34	< 0.001	< 0.001
let-7c-5p|0	0.34	< 0.001	< 0.001
miR-101-3p|0	0.31	< 0.001	< 0.001
miR-203a-3p|0	0.28	< 0.001	< 0.001
miR-29a-3p|-1	0.26	< 0.001	< 0.001
miR-200b-3p|+1	0.22	0.005	0.017
miR-378a-3p|0	0.39	0.027	0.082

*Distance is Kolmogorov-Smirnov test statistic equal to maximum difference between fold change cumulative distribution functions of targets with 6-mers and non-target genes. miRNA-378-3p|+1 failed the test. miRNA-29a-3p|+1 passed the test according to the FDR value, however, its predicted targets decreased expression while miR-29a-3p|-1 was down-regulated itself (**Figure 3**).

### 3.2 Pathway enrichment analysis of differentially expressed mRNA-targets of changed miRNAs

**Figure 4:**
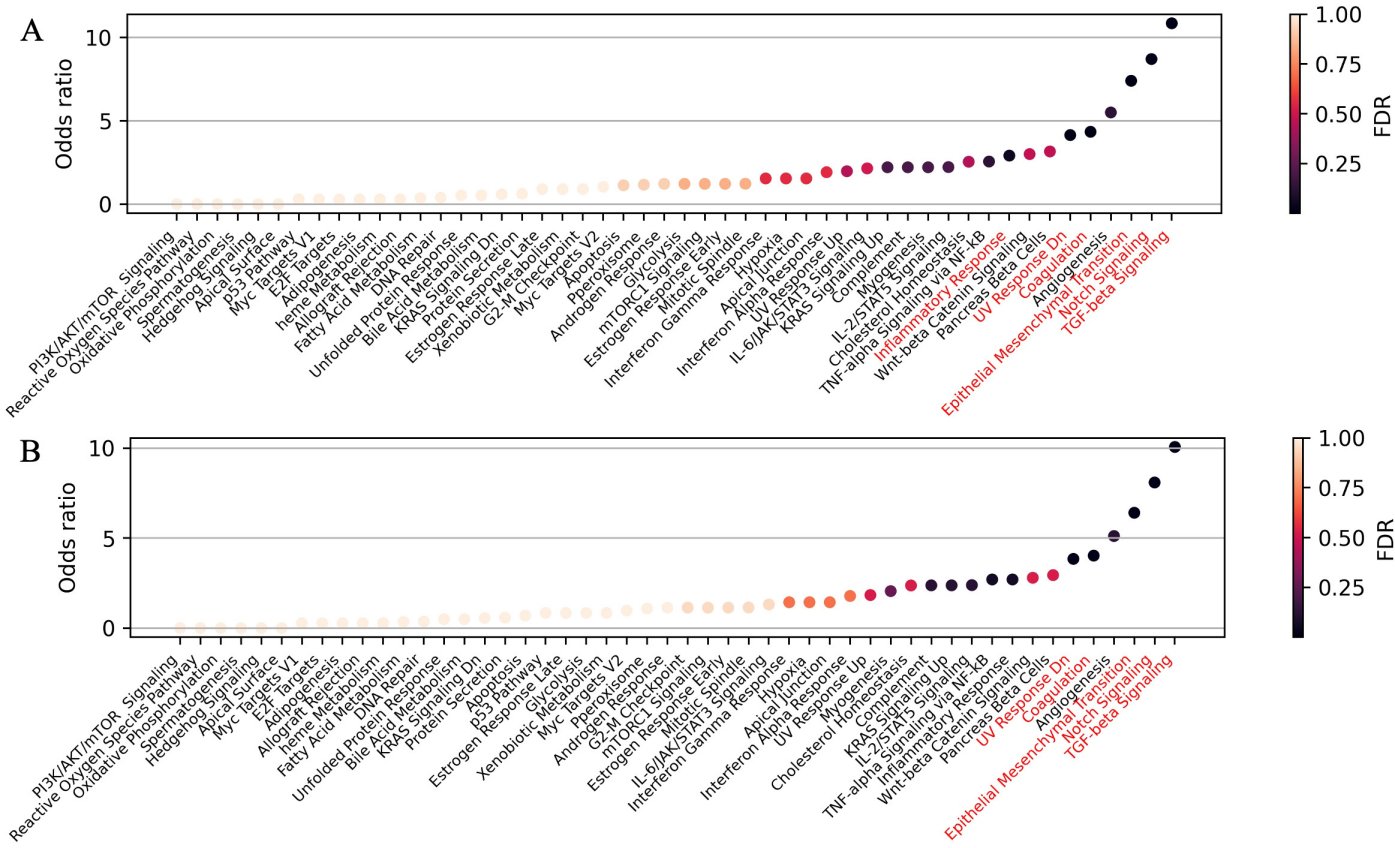
Pathway enrichment analysis of differentially expressed mRNA-targets for **(A)** seven selected canonical miRNAs—let-7a-5p, let-7b-5p, let-7c-5p, miR-203a-3p, miR-101-3p, miR-125a-5p, and miR-185-5p—and **(B)** all 11 highly expressed upregulated canonical miRNAs. Overrepresented biological processes are highlighted in red.

To evaluate the potential biological roles of the identified canonical miRNAs—let-7a-5p, let-7b-5p, let-7c-5p, miR-203a-3p, miR-101-3p, miR-185-5p, and miR-125a-5p—we performed a gene set enrichment analysis on the differentially expressed, validated mRNA targets for each miRNA individually (**Supplementary Figure S3**) and collectively for all seven miRNAs (**Figure 4A**). The 5'-isoforms were not included in the analysis, as no experimentally confirmed mRNA targets are currently known for them. This analysis highlighted such key processes associated with tumor metastasis as Epithelial-to-Mesenchymal Transition (EMT), TGF-β signaling, and Notch signaling.

We also performed gene set enrichment analysis on the differentially expressed mRNA targets of all 11 highly expressed, upregulated canonical miRNAs (**Figure 2**). Interestingly, when we included the mRNA targets of the four additional miRNAs (miR-378a-3p, miR-30d-5p, miR-92a-3p, and miR-31-5p) alongside with the seven previously selected, no new overrepresented processes were identified, while Inflammatory Response lost statistical significance (**Figure 4B**). This suggests that the initially selected miRNAs may play a pivotal role in regulating tumor metastasis. A similar analysis of seven highly expressed downregulated miRNAs (**Figure 2**) revealed no overrepresented pathways among the upregulated mRNA targets (**Supplementary Figure S3**, panel **H**).

### 3.3 Possible regulators of miRNA changes in HT-29 xenograft tumors upon CD44 kd

In the genome, eight of the nine miRNAs/isomiRs identified in our study are localized in intergenic regions as part of several miRNA clusters [**Table 3** (33, 34)], each with its own promoters. Therefore, we sought to identify transcription factors that could serve as key regulator of miRNA/isomiR expression in HT-29 xenografts with CD44 kd. Notably, the ninth miRNA, canonical miR-185-5p, is encoded within an intron of the protein-coding gene *TANGO2*.

**Table 3:** MiRNA localization in genome.*.

miRNA	miRNA cluster	Genome location
let-7a-5p	let-7a-1, let-7f-1, let-7d	Chromosome 9, intergenic region
	⁠miR-100, let-7a-2	Chromosome 11, intergenic region
	let-7a-3, miR-4763, let-7b	Chromosome 22, intergenic region
let-7b-5p	⁠let-7a-3, miR-4763, let-7b	Chromosome 22, intergenic region
let-7c-5p	miR-99a, let-7c	Chromosome 21, intergenic region
miR-203a-3p	miR-203a, miR-203b	Chromosome 14, intergenic region
miR-101-3p	miR-101, miR-3671	Chromosome 1, intergenic region
miR-200b-3p	miR-200a, miR-200b, miR-429	Chromosome 1, intergenic region
miR-185-5p	–	Chromosome 22, intron of *TANGO2* gene

* According to the study of Kabekkodu et al. (33).

First, we analyzed transcription factors (TFs), which are key regulators that may contribute to changes in mRNA expression profiles following CD44 kd. Using the TRRUST online tool, we identified 75 TFs. We then hypothesized that if some of these TFs regulate gene expression in xenograft tumors, their expression should also change after CD44 kd. As a result, we identified 16 TFs as potential regulators of mRNA expression changes. Building on this, we reasoned that TFs involved in miRNA expression regulation should also be among these 16. To investigate further, we searched the TransmiR v3.0 database for TFs that could regulate the expression of differentially expressed miRNAs/isomiRs and selected those that were also present in the list of 16 TFs. This analysis identified 11 TFs, which are presented in **Table 4**. For the differentially expressed 5’-isomiRs we identified, we considered the TFs that regulate their canonical forms, as both the canonical miRNA and its 5’-isoforms are encoded by the same gene. Thus, we considered eight canonical miRNAs in our analysis of key TFs (**Table 4**).

**Table 4:** Possible regulators (transcription factors) of miRNA expression.

let-7a-1	let-7a-2	let-7a-3	let-7b-5p	let-7c-5p	miR-203a-3p	miR-200b-3p	miR-101-3p	miR-125a-5p	miR-185-5p
EGR1	EGR1	EGR1	EGR1		EGR1	EGR1	EGR1	EGR1	EGR1
EP300	EP300	EP300	EP300	EP300	EP300	EP300		EP300	EP300
EPAS1								EPAS1	
KLF4	KLF4	KLF4	KLF4	KLF4	KLF4	KLF4	KLF4		
NFKB1		NFKB1	NFKB1			NFKB1			
NPM1	NPM1	NPM1	NPM1	NPM1	NPM1	NPM1		NPM1	NPM1
SMAD3	SMAD3	SMAD3	SMAD3			SMAD3		SMAD3	SMAD3
SMARCA4	SMARCA4	SMARCA4	SMARCA4	SMARCA4	SMARCA4	SMARCA4		SMARCA4	SMARCA4
		SREBF1	SREBF1						SREBF1
SREBF2	SREBF2	SREBF2	SREBF2					SREBF2	SREBF2
STAT3	STAT3	STAT3	STAT3	STAT3	STAT3	STAT3		STAT3	

Among the identified TFs, STAT3 and NF-κB were also found to be key regulators of transcriptome changes in HT-29 xenograft tumors following CD44 kd, as indicated by gene enrichment analysis in our previous study (5). Another gene identified gene, *EP300*, encodes the transcriptional co-activator p300, which activates STAT3 and NF-κB by acetylating them (35, 36). According to mRNA-Seq data, the expression of *STAT3* and *EP300* was downregulated following CD44 kd (5). This downregulation was further confirmed by qPCR analysis in our study. Interestingly, STAT3 and p300 were identified as possible regulators of six and seven out of the eight miRNAs we studied, respectively (**Table 4**). We propose that TFs regulating nearly all identified miRNAs play the most important role. These key TFs include EGR1, NPM1, SMARCA4, and KLF4. *NPM1* was the most highly expressed regulator gene identified (**Supplementary Table S1**). It encodes nucleophosmin, a nucleolar phosphoprotein (37), which has been reported to function as both an oncogene and a tumor suppressor depending on cell types.

*SMAD3* and *SREBF2* were the second and third most highly expressed TF genes (**Supplementary Table S1**), but they were identified as potential regulators of only five and four of the eight miRNAs, respectively (**Table 4**).

Notably, *EPAS1* was the second most downregulated TF gene (2.7-fold), encoding HIF-2α, a key factor in the hypoxic response. However, we did not observe its downregulation at a protein level (5). Additionally, HIF-2α was identified as a regulator of only two of eight miRNAs. For these reasons, we did not include this TF in further consideration.

## 4 Discussion

In our previous study, we demonstrated that CD44 kd induces substantial changes in HT-29 xenograft tumors, leading to reduced metastasis. In this study, we investigate the molecular mechanisms underlying this effect. Our analysis of isomiR profile identified seven canonical miRNAs and two miRNA 5’-isoforms—let-7a-5p, let-7b-5p, let-7c-5p, miR-203a-3p, miR-203a-3p|+1, miR-101-3p, miR-200b-3p|+1, miR-125a-5p, and miR-185-5p—that may play a role in these mechanisms.

Pathway enrichment analysis of differentially expressed mRNA-targets of the altered miRNAs revealed several key pathways critical for tumor metastasis, including TGF-β signaling, Notch signaling, and EMT. Notably, the Notch signaling pathway plays a crucial role in maintaining stem-like properties in tumor cells (38). Dysregulation of this pathway has been linked to EMT, angiogenesis, and metastasis (38). In CRC, elevated Notch1 expression is associated with lymph node metastasis, while reduced Notch2 expression predicts poor prognosis (38). Notch ligands JAG1, JAG2, and DLL4 are significantly upregulated in CRC, correlating with poor outcomes (38). Interestingly, Let-7 miRNAs and miR-203a-3p have been reported to inhibit the Notch signaling pathway (39), potentially contributing to metastasis suppression.

**Figure 5:**
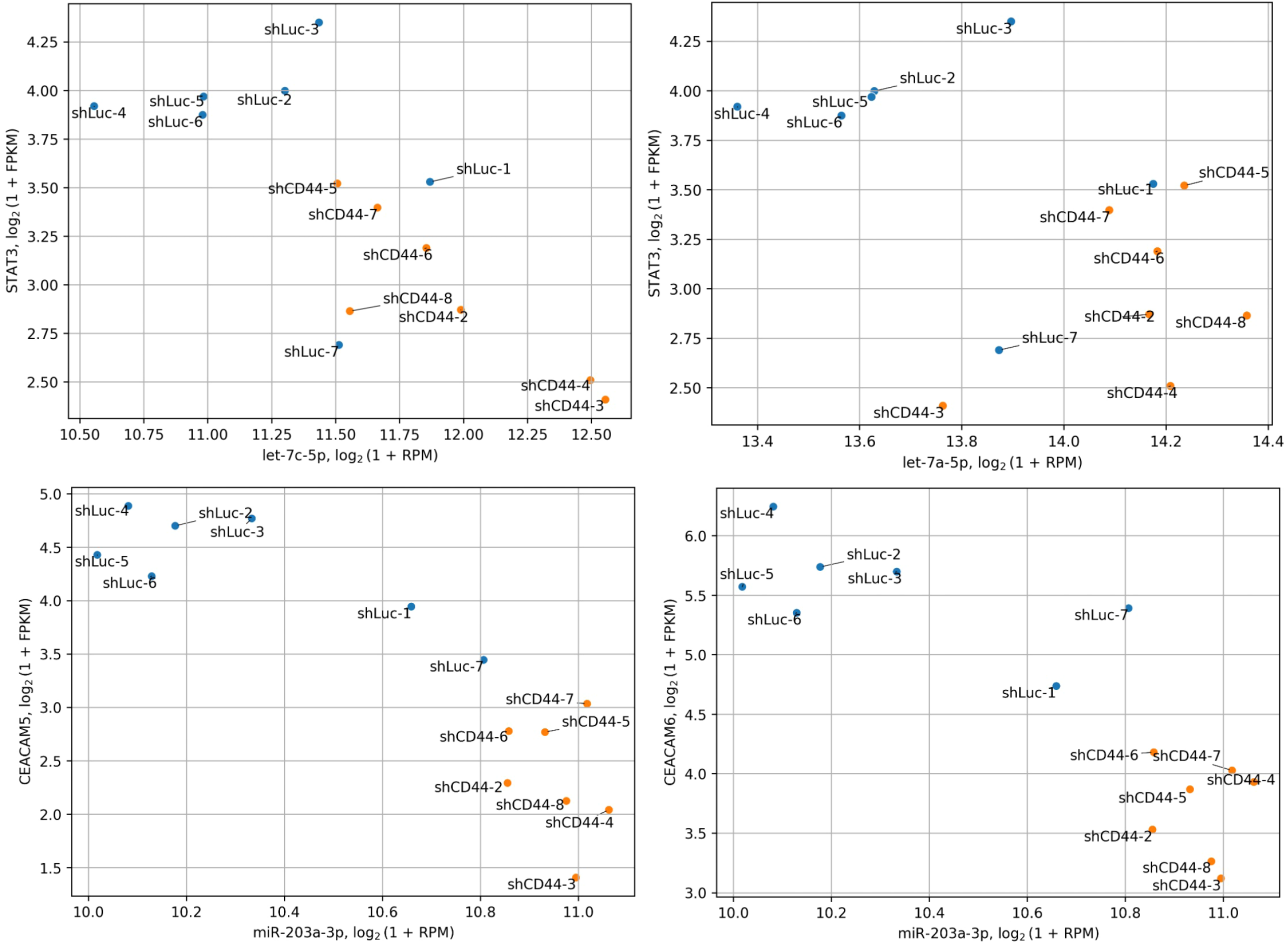
Co-distribution of miRNA and target gene expression. The expression of Let-7 miRNAs significantly anticorrelates with their target gene, *STAT3*, while miR-203a-3p shows a similar anticorrelation with *CEACAM5* and *CEACAM6*. *STAT3*, *CEACAM5* and *CEACAM6* are significantly downregulated following CD44 kd.

Additionally, the Let-7 family of miRNAs acts as tumor suppressors by inhibiting STAT3 expression, thereby hindering tumorigenesis and metastasis (40–43). Aberrant STAT3 signaling is associated with CRC (44). Consistently, let-7a-5p expression inversely correlates with metastasis in CRC patients (45). Suppression of Let-7 miRNA biogenesis or expression promotes EMT and metastasis in CRC (46–48). Importantly, our analysis suggests that STAT3 may be a key regulator in CD44 kd HT-29 xenografts. CD44 cooperates with STAT3 in various tumor types, contributing to cancer invasion, metastasis, disease recurrence, and chemoresistance (49). HA-activated CD44 can activate STAT3 via phosphorylation through PI3K signaling cascades (50). Additionally, CD44 can bind nuclear STAT3 and p300 acetyltransferase, promoting STAT3 acetylation, which enhances cell proliferation and cancer stem cell like properties in colon and other cancer cells (51, 52). In our study, CD44 kd reduced expression of *EP300* (which encodes p300), indicating diminished STAT3 activity. Beyond activation, CD44 also regulates STAT3 expression (49), aligning with our observation of decreased STAT3 levels in CD44 kd tumors. Notably, STAT3 functions as both an activator and a repressor of miRNA expression (44, 53, 54). Our analysis identified STAT3 as a potential regulator of nearly all significant miRNAs found in this study (**Table 4**). Considering all these findings, we hypothesize a bidirectional regulatory relationship in HT-29 tumors: Let-7 miRNAs downregulate STAT3 expression, while STAT3 suppresses miRNA expression, forming a complex interplay that regulates CRC properties. Notably, miR-203a-3p upregulation observed in CD44 kd tumors may also contribute to the inhibition of the STAT3 signaling (39). We illustrated the possibility of such interactions in HT-29 xenograft tumors on scatterplots (**Figure 5**).

Both increased and decreased expression of miR-203a-3p has been linked to CRC recurrence and poorer survival (34, 55, 56). This discrepancy may be due to patient variability (race/ethnicity, disease stage) (57), or the distinct roles played by the two isoforms of miR-203a-3p identified in our study. While existing data only provide information on the total miR-203a-3p expression in CRC, further research is needed to delineate the functions of its isoforms. Despite these uncertainties, miR-203a-3p inhibits invasion and migration of SW480 and HT29 cells (58), downregulates EMT markers, and increased E−cadherin expression (58), supporting its tumor-suppressive role in CRC. Additionally, the canonical miR-203a-3p isoform targets stem cell markers CEACAM5/6, which were downregulated at the protein level in xenograft tumors with CD44 kd (5). Thus, miR-203a-3p upregulation may reduce CEACAM5/6 expression (**Figure 5**) and stem-like properties in CRC cells following CD44 kd. Testing this hypothesis could provide valuable insights into the molecular underpinnings of this effect.

MiRNA-101-3p has also been shown to have a tumor suppressive role in CRC (59–64). Notably, its expression can be inhibited by EGR1 TF (64), which was downregulated upon CD44 kd and identified as a potential regulator of nearly all significant miRNAs found in this study (**Table 4**). MiRNA-101-3p may exert its antitumor effects in CRC by suppressing the Wnt/β-catenin signaling pathway (59).

MiR-125a-5p has also been identified as a tumor suppressor. Specifically, it inhibits CRC cell proliferation, migration, invasion, and EMT (65–68). Additionally, miR-125a-5p has been shown to suppress HT-29 xenograft tumor growth (69).

MiR-185-5p has not been as extensively studied in CRC as other canonical miRNAs identified in our study. However, several lncRNAs were identified as molecular sponges for miR-185-5p, promoting CRC cell proliferation, migration, and invasion (70, 71). This miRNA warrants further investigation for its role in colorectal cancer. To our knowledge, no information is available on the miR-200b-3p|+1 isoform in CRC. However, the extensive data supporting the tumor suppressor function of its canonical isoform, miR-200b-3p|0 (e.g (61, 72–74).), makes miR-200b-3p|+1 particularly intriguing for further investigation.

NPM1 was the most highly expressed regulatory gene in this study. It serves as a histone chaperone and transcriptional co-regulator, interacting with various proteins such as c-Myc, c-Fos, p53, ARF, ATF5, RUNX1, and Rb (37, 75–77). It may also directly regulate transcription by binding gene promoters, though this role remains understudied (78). Interestingly, NPM1 can act as either an oncogene or a tumor suppressor, depending on the cell type (37). Altered expression of Let-7 miRNAs has been associated with mutated form of NPM1 in acute myeloid leukemia (79), suggesting that NPM1 may influence miRNA expression in CRC. However, this connection remains ambiguous and warrants further investigation.

Overall, our findings suggest that the CD44-STAT3-Let-7 miRNA axis is one of the most plausible regulatory pathways in CRC metastasis via the CD44 receptor. Additionally, our study indicates the potential involvement of both the canonical miR-203a-3p and its +1 5'-isoform in this network. Our data also suggest a role for miR-101-3p and miR-125a-5p in metastasis regulation through CD44 kd. A limitation of this study is the lack of gain-of-function and loss-of-function experiments using miRNA mimics and inhibitors to directly demonstrate the role of Let-7 miRNAs, miR-203a-3p, miR-101-3p, and miR-125a-5p in metastasis. However, previous studies support their tumor-suppressive and antimetastatic effects in CRC (46, 47, 56, 58, 61, 65, 80, 81). A particularly valuable future experiment would involve using miRNA mimics or inhibitors followed by RNA-seq to identify genes directly regulated by these miRNAs and those independently affected by CD44 kd. Further investigation into the regulation of Let-7 miRNAs and miR-203a-3p by transcription factors NPM1, KLF4, and SMARCA4, as well as miRNA-101-3p by KLF4, could provide deeper insights into the molecular mechanisms underlying metastatic progression in CRC.

## Data Availability

The datasets presented in this study can be found in online repositories. The names of the repository/repositories and accession number(s) can be found below: GSE284708 (GEO).
