# Supplementary material for: CD44 knockdown alters miRNA expression and their target genes in colon cancer
Source: Front Immunol. 2025 May 14;16:1552665. doi: 10.3389/fimmu.2025.1552665 (PMC12116639; doi:10.3389/fimmu.2025.1552665)
Supplement: Supplementary file 2 [file DataSheet2.pdf]

Supplementary Material

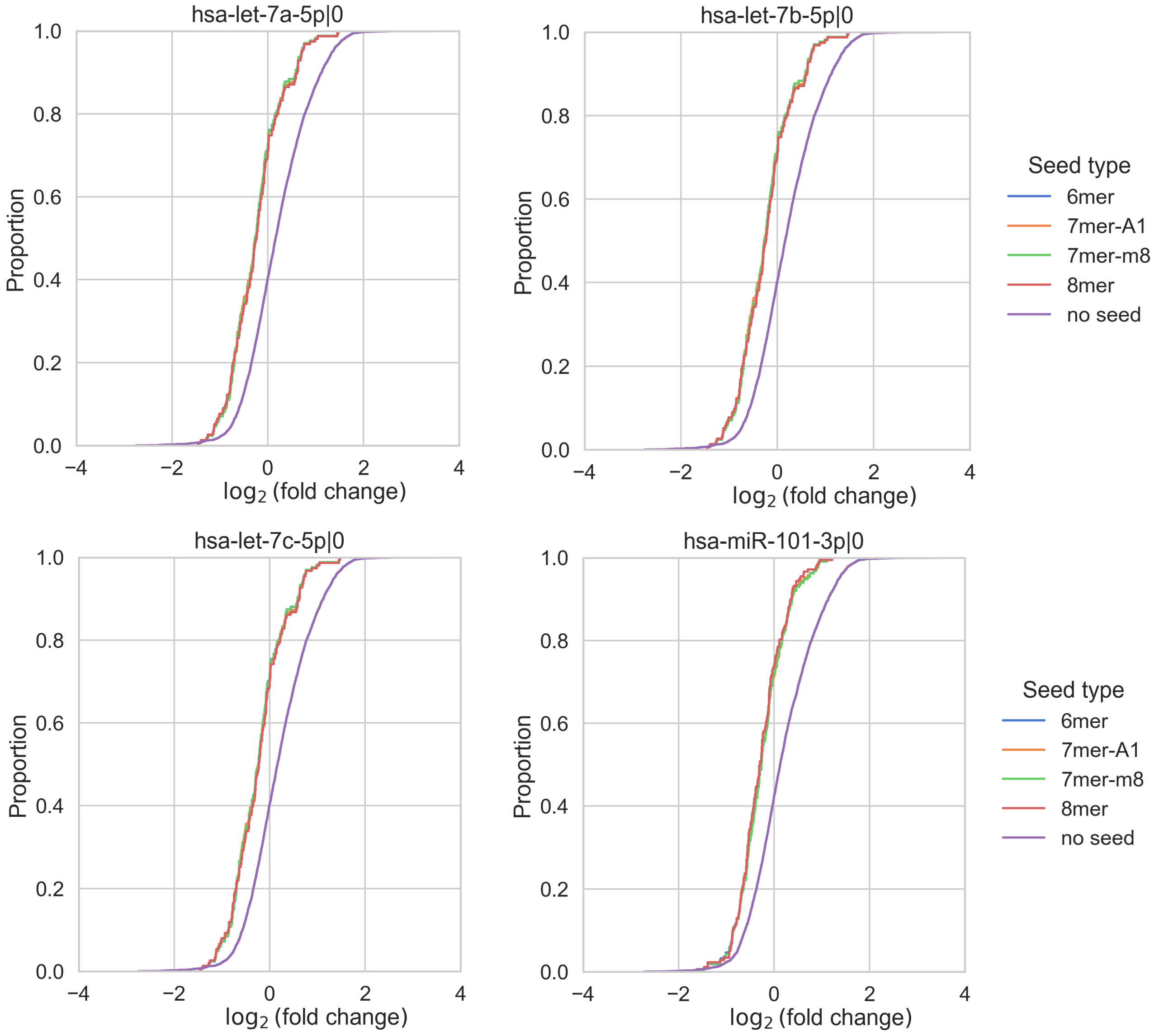

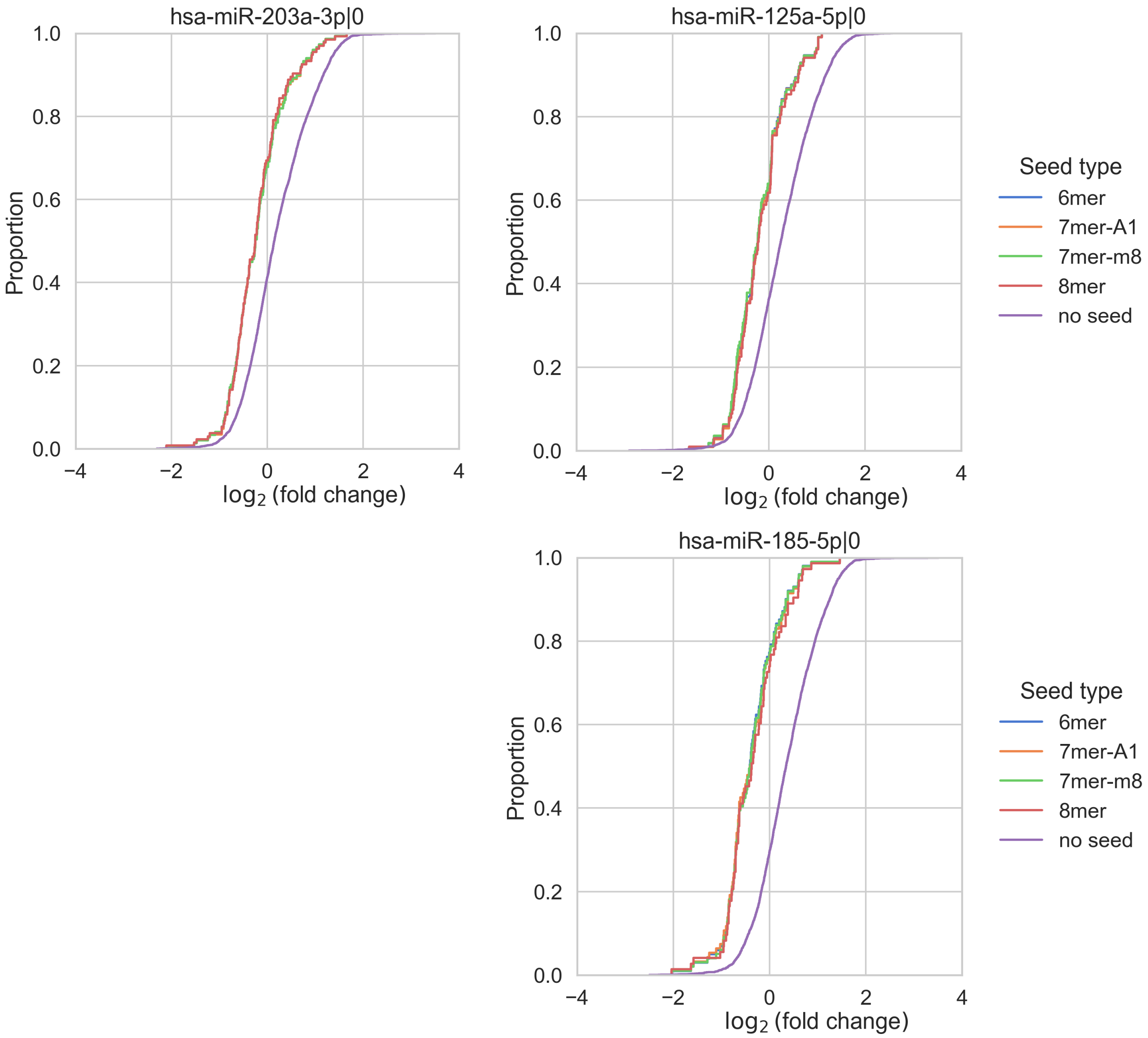

Supplementary Figure S2
Enrichment analysis of seed regions. The log2(fold change) distributions of differentially expressed genes with and without a corresponding seed differ significantly (FDR < 0.05) for seven canonical differentially expressed isomiRs.
