# Supplementary material for: CD44 knockdown alters miRNA expression and their target genes in colon cancer
Source: Front Immunol. 2025 May 14;16:1552665. doi: 10.3389/fimmu.2025.1552665 (PMC12116639; doi:10.3389/fimmu.2025.1552665)
Supplement: Supplementary file 3 [file DataSheet3.pdf]

Supplementary Material

A

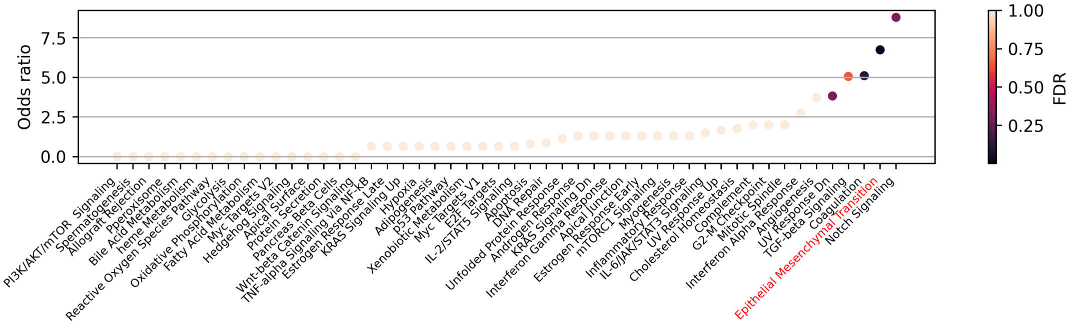

B

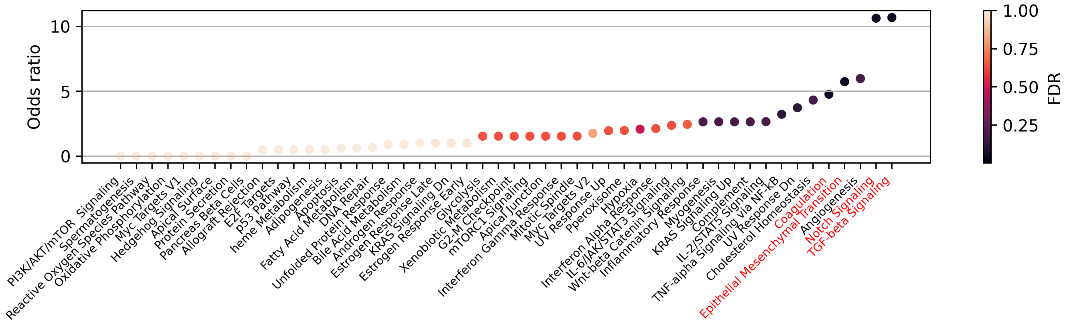

C

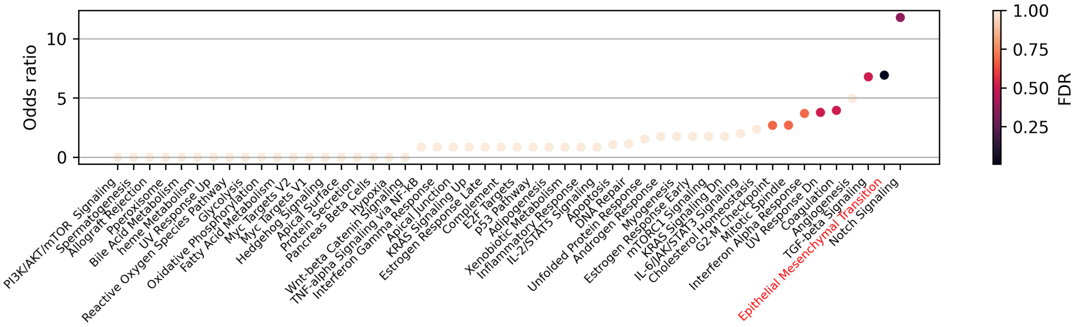

D

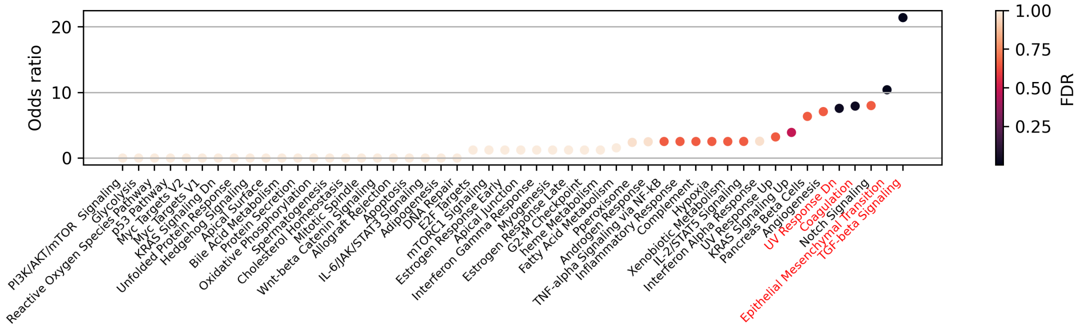

E

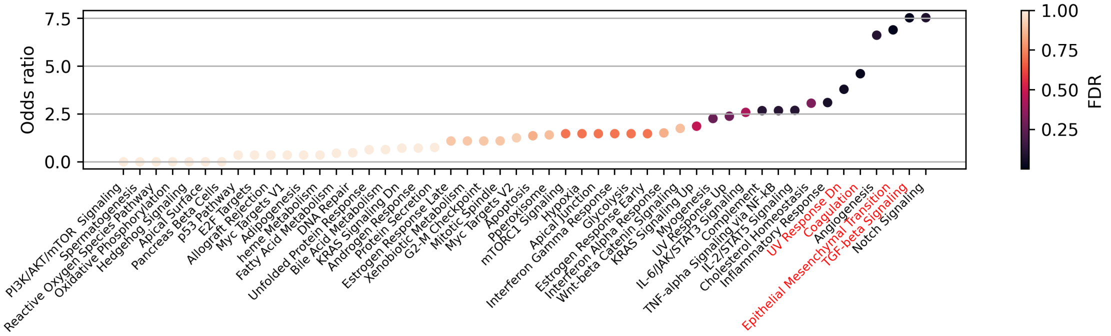

F

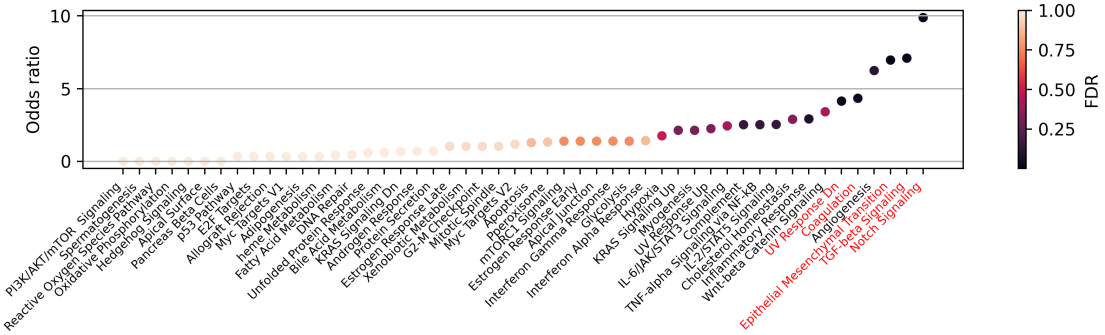

G

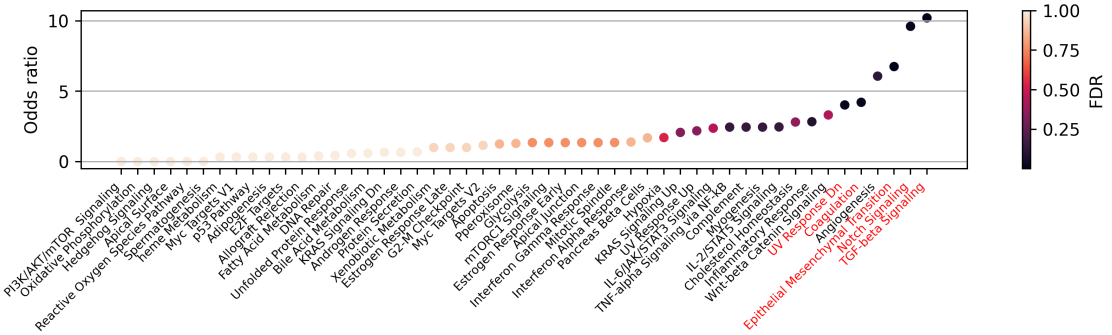

H

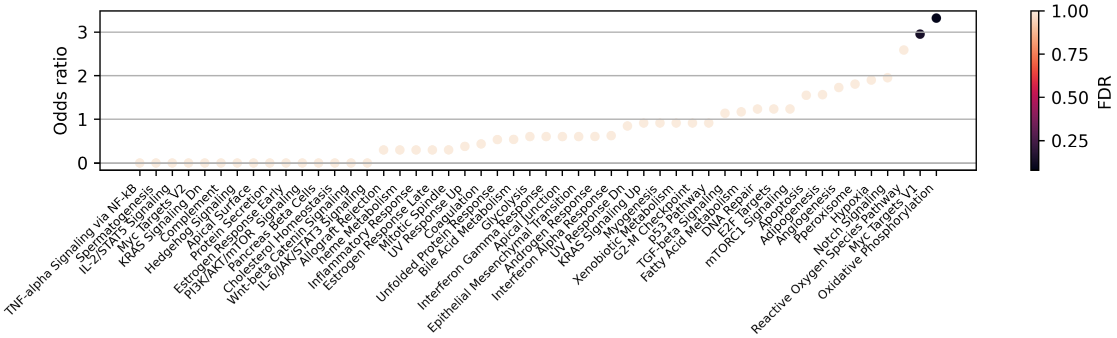

Supplementary Figure S3. Pathway enrichment analysis of differentially expressed mRNA-targets for individual miRNAs—let-7a-5p (A), let-7b-5p (B), let-7c-5p (C), miR-203a-3p (D), miR-101-3p (E), miR-125a-5p (F), miR-185-5p (G) —as well as for a group of seven highly expressed downregulated miRNAs (H). Overrepresented biological process names are highlighted in red
