# Supplementary material for: CD44 knockdown alters miRNA expression and their target genes in colon cancer
Source: Front Immunol. 2025 May 14;16:1552665. doi: 10.3389/fimmu.2025.1552665 (PMC12116639; doi:10.3389/fimmu.2025.1552665)
Supplement: Supplementary file 4 [file DataSheet4.pdf]

Supplementary Material

Supplementary Table S2 Extression changes of possible regulators (transcription factors) of miRNAs expression in HT-29 xenografts upon CD44 kd.

TF	FC	Base Mean
EGR1	-1,7	329
EP300	-2,4	412
EPAS1	-2,7	731
KLF4	-2,1	660
NFKB1	-1,6	496
NPM1	2,2	33356
SMAD3	-1,8	1860
SMARCA4	-1,7	728
SREBF1	-1,6	497
SREBF2	-1,6	1649
STAT3	-1,9	1125
