# Supplementary material for: CD44 knockdown alters miRNA expression and their target genes in colon cancer
Source: Front Immunol. 2025 May 14;16:1552665. doi: 10.3389/fimmu.2025.1552665 (PMC12116639; doi:10.3389/fimmu.2025.1552665)
Supplement: Supplementary file 5 [file DataSheet5.zip › QC reports/shLUC_1.fastq.gz FastQC Report.pdf]

FastQC Report

Mon 31 Mar 2025
shLUC_1.fastq.gz

Summary

- Basic Statistics
- Per base sequence quality
- Per tile sequence quality
- Per sequence quality scores
- Per base sequence content
- Per sequence GC content
- Per base N content
- Sequence Length Distribution
- Sequence Duplication Levels
- Overrepresented sequences
- Adapter Content

Basic Statistics

Measure	Value
Filename	shLUC_1.fastq.gz
File type	Conventional base calls
Encoding	Sanger / Illumina 1.9
Total Sequences	5391323
Sequences flagged as poor quality	0
Sequence length	18-36
%GC	48

Per base sequence quality

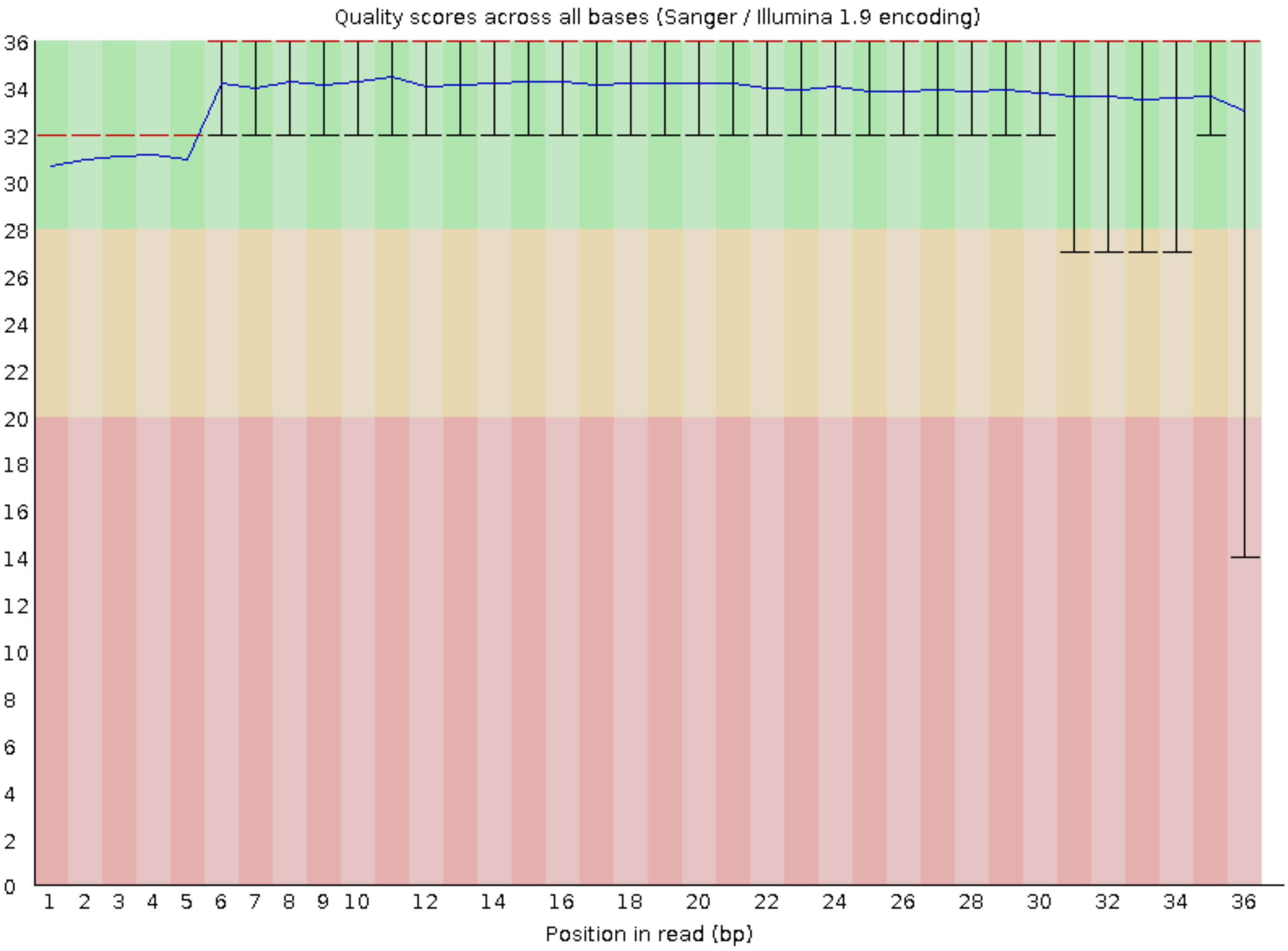

Per tile sequence quality

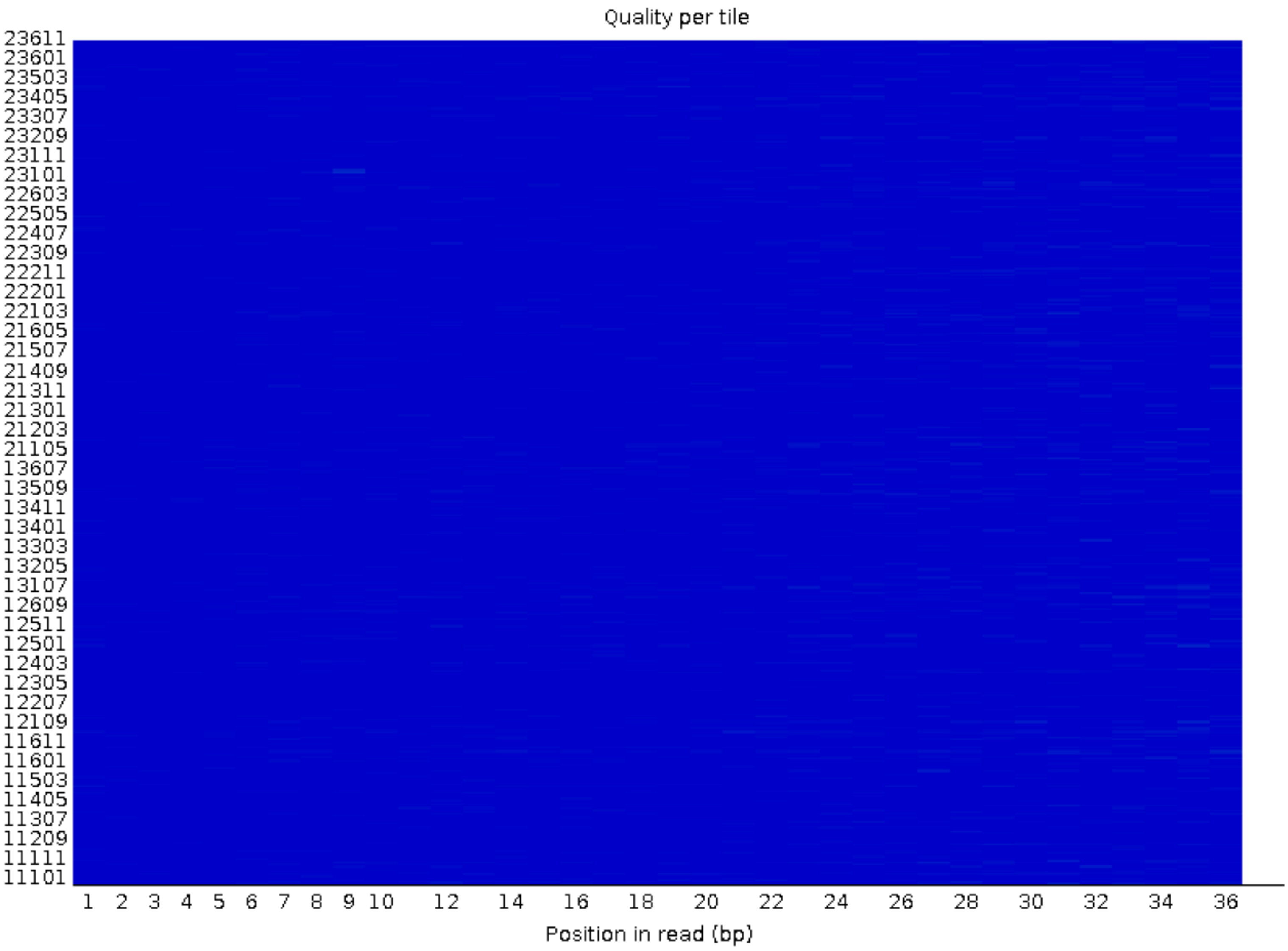

Per sequence quality scores

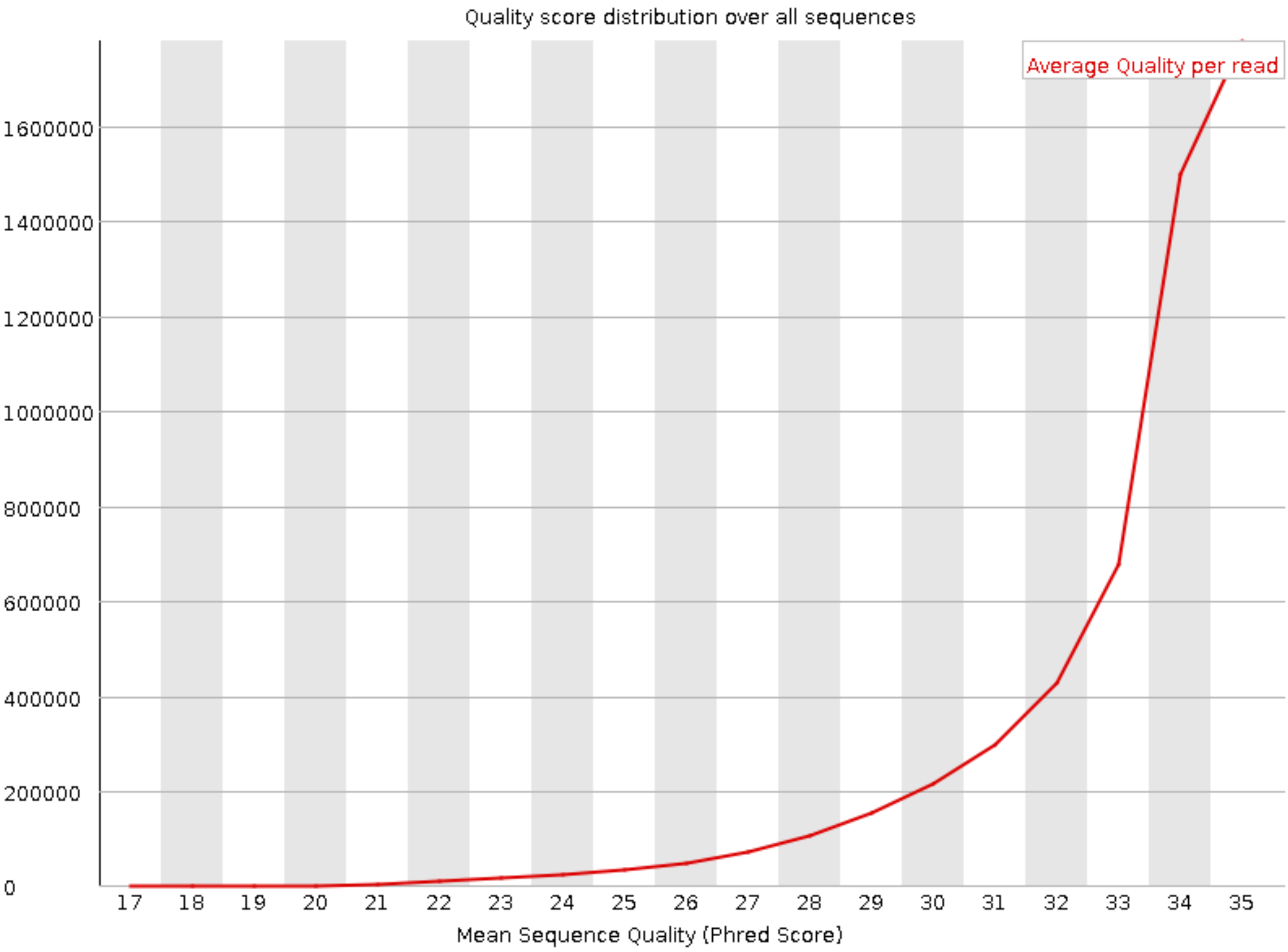

Per base sequence content

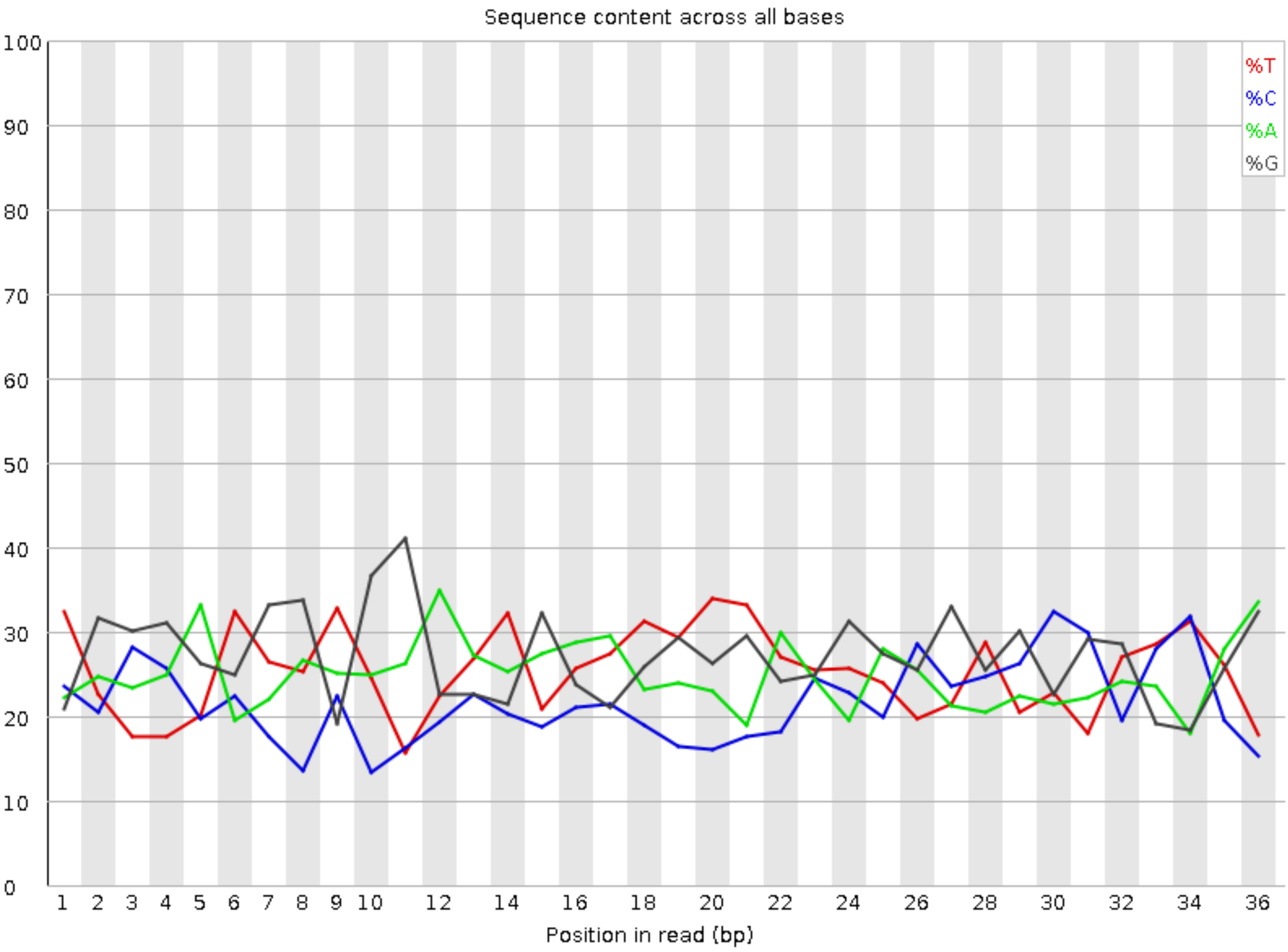

Per sequence GC content

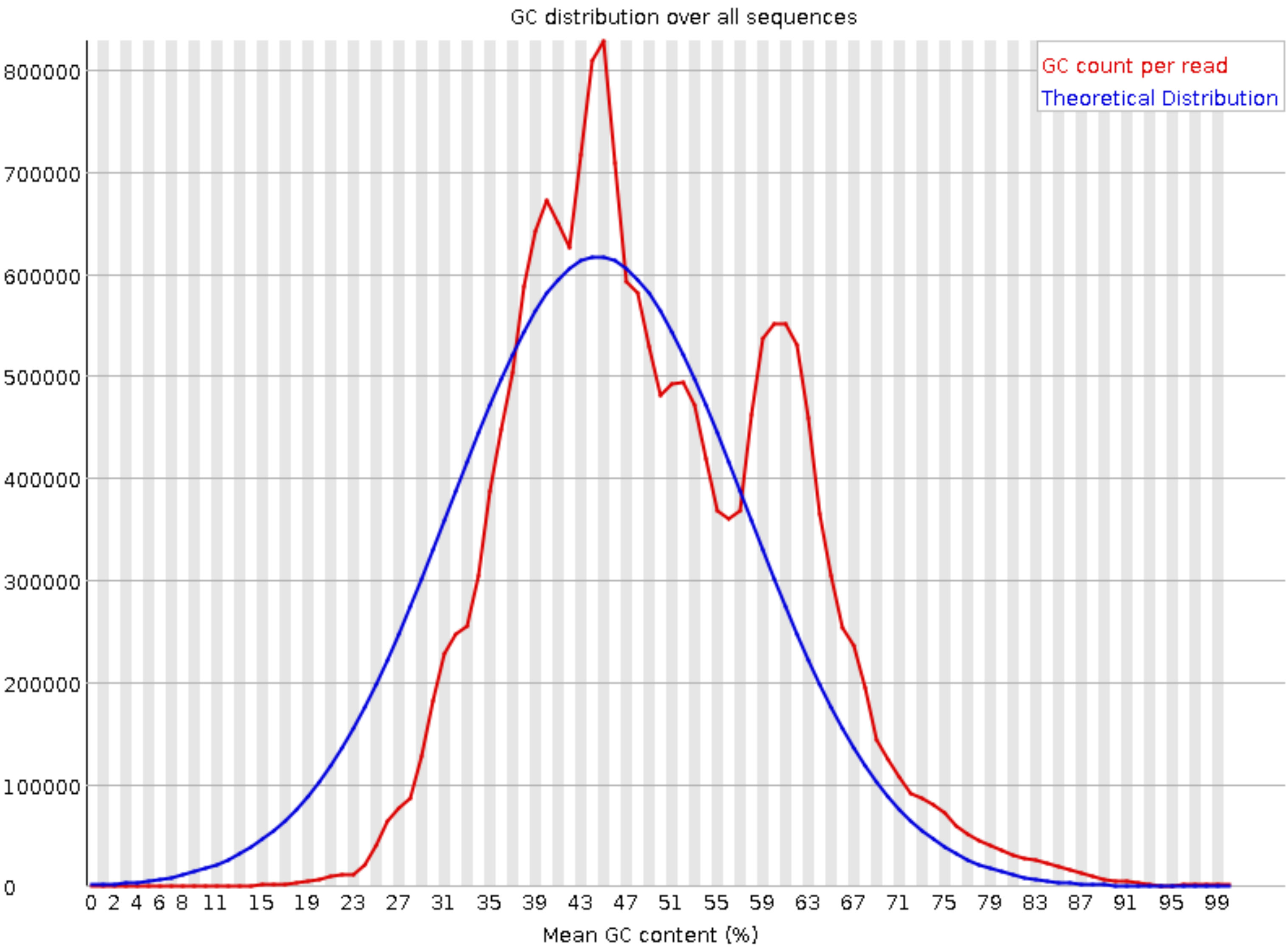

Per base N content

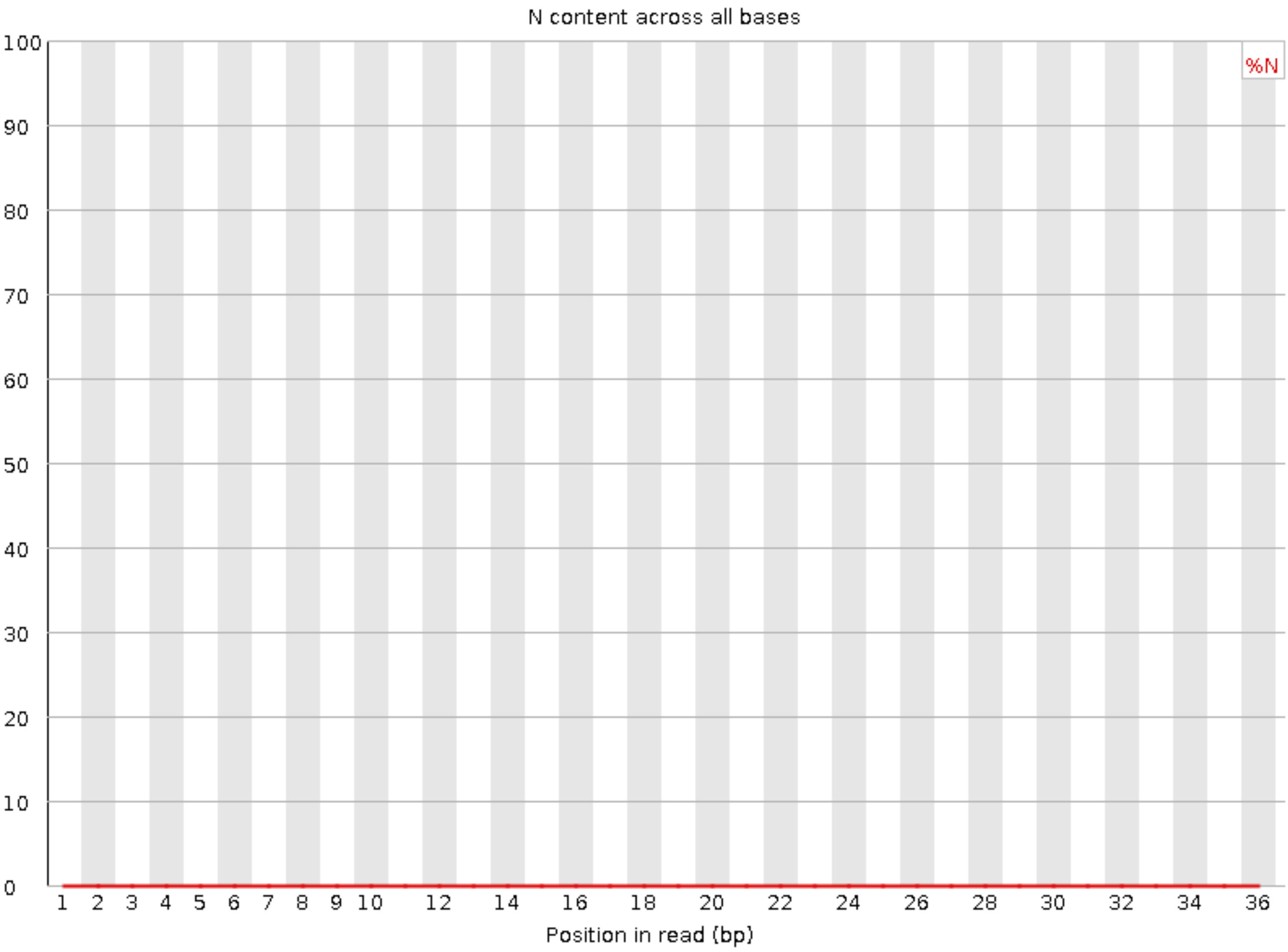

Sequence Length Distribution

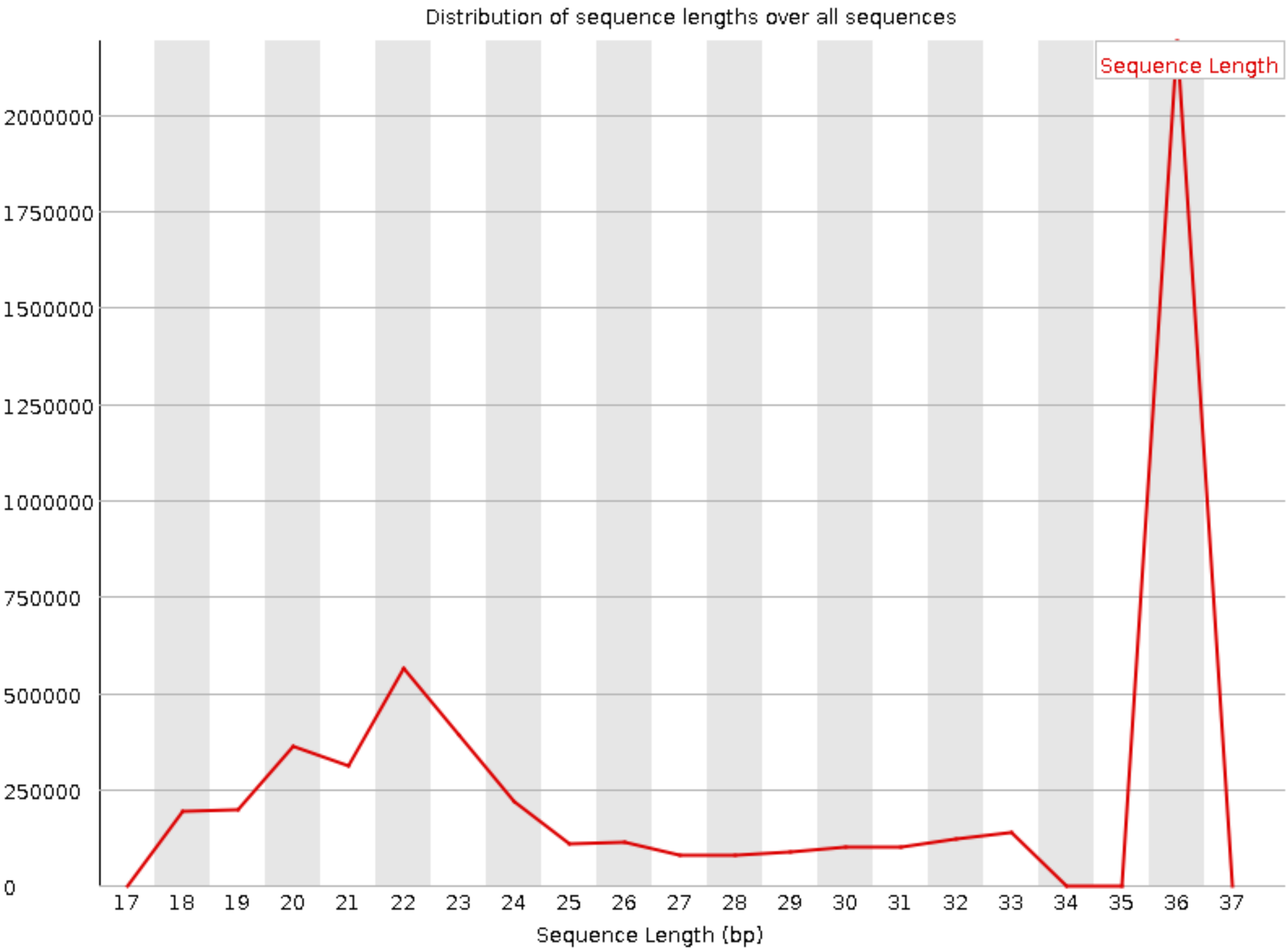

Sequence Duplication Levels

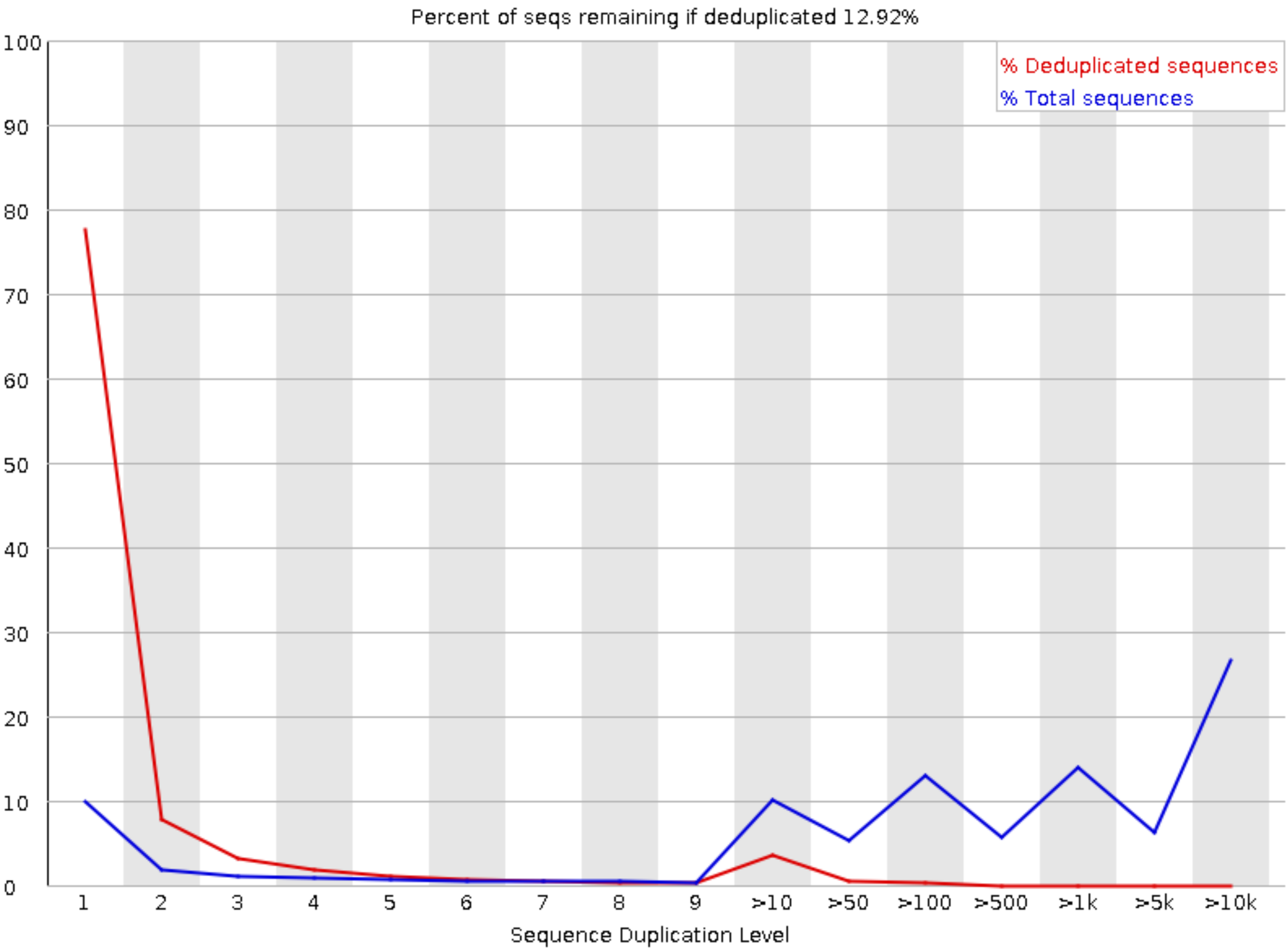

Overrepresented sequences

Sequence	Count	Percentage	Possible Source
CGCGACCTCAGATCAGACGT	132025	2.4488423342470855	No Hit
TGCTCTGATGAAATCACTAATAGGAAGTGCCGTCAG	114891	2.131035369240537	No Hit
TAGCTTATCAGACTGATGTTGAC	79061	1.466448958817715	No Hit
GTGAAATGATGGCAATCATCTTTCGGGACTGACCTG	63617	1.1799886595553633	No Hit
GTTTGTGATGACTTACATGGAATCTCGTTCGGCTGA	51283	0.9512136445915038	No Hit
TAGCTTATCAGACTGATGTTGA	49074	0.9102403992489414	No Hit
TTGAATGATGACTTTAATTGTCGGATACCCCTTCAC	41673	0.7729642612768702	No Hit
CCTGGATGATGATAAGCAAATGCTGACTGAACATGA	40425	0.7498159542657711	No Hit
AGTAGTGATGAAATTCCACTTCATTGGTCCGTGTTT	37838	0.7018314428573469	No Hit
GCCTCTGATGAAGCCTGTGTTGGTAGGGACATCTGA	36399	0.6751404061674657	No Hit
TGAGGTAGTAGATTGTATAGTT	32254	0.5982576076410187	No Hit
GTGCAATGATGTATTTTATTCAACACATCATTCTGA	32031	0.5941213316286188	No Hit

Sequence	Count	Percentage	Possible Source
ACCGGGTGCTGTAGGCTT	31936	0.5923592409506906	No Hit
TGGAAGACTAGTGATTTTGTTGTT	30941	0.5739036596397582	No Hit
ACCGGGTGCTGTAGGCTTT	30606	0.5676899714596956	No Hit
TATCTGTGATGATCTTATCCCGAACCTGAACTTCTG	27691	0.5136216101316875	No Hit
TCGCTGCGATCTATTGAAAGTCAGCCCTCGACACAA	27377	0.5077974367330617	No Hit
CGACTCTTAGCGGTGGATCACTCGGCTCGTGCGTCG	26747	0.49611199328995875	No Hit
GAGAAGACGGTCGAACTTGACTATCT	26737	0.49592651006070304	No Hit
TGAAATGATGGCAATCATCTTTCGGGACTGACCTGA	26291	0.4876539580359033	No Hit
TGAGGTAGTAGTTTGTGCTGTT	23837	0.44213637357657853	No Hit
ATACATGATGATCTCAATCCAACTTGAACTCTCTCA	22856	0.42394046878660396	No Hit
CGCGACCTCAGATCAGACGC	21506	0.39890023283709763	No Hit
TTTCTATGATGAATCAAACTAGCTCACTATGACCGA	18829	0.34924637236537304	No Hit
TTGGTACTAGCAACGCACTTT	18502	0.34318107076871485	No Hit
GATGGGAGACCGCCTGGGAATACCGGGTGCTGTAGG	18091	0.3355577100463096	No Hit
CTAGACTGAAGCTCCTTGAGG	18088	0.33550206507753294	No Hit
CTCGCTGCGATCTATTGAAAGTCAGCCCTCGACACA	17894	0.33190369042997425	No Hit
CGCTGCGATCTATTGAAAGTCAGCCCTCGACACAAG	17290	0.3207005033829359	No Hit
CAGGACGGTGGCCATGGAAGTCGGAATCCGCTAAGG	17024	0.31576664948473687	No Hit
TGGAATGTAAAGAAGTATGTAT	16693	0.30962715459637646	No Hit
CGCGACCTCAGATCAGACGTGGCGACCCGCTGAATT	16325	0.3028013717597703	No Hit
TTTGAATGATGACTTTAATTGTCGGATACCCCTTCA	16214	0.3007425079150331	No Hit
TACCCTGTAGATCCGAATTTGT	16134	0.2992586420809883	No Hit
TAGCTTATCAGACTGATGTTGAT	15878	0.2945102714120449	No Hit
CTGGATGATGATAAGCAAATGCTGACTGAACATGAA	15248	0.2828248279689419	No Hit
CTACGGGGATGATTTTACGAACTGAACTCTCTCTTT	15079	0.2796901613945223	No Hit
TAGCTTATCAGACTGATGTTGACT	14871	0.27583211022600573	No Hit
TCAGTGCACTACAGAACTTTGT	14493	0.268820844160144	No Hit
TGCCTCTGATGAAGCCTGTGTTGGTAGGGACATCTG	13611	0.2524612233397999	No Hit
AGAAGACGGTCGAACTTGACTATCT	13276	0.24624753515973724	No Hit
GTGAAATGATGGCAAATCATCTTTCGGGACTGACCT	13134	0.2436136733043077	No Hit
CTCCTACTTGGATAACTGTGGTAATTCTAGAGCTAA	12579	0.2333193540806218	No Hit
TAGCTTATCAGACTGATGTTGACA	12374	0.22951694788088192	No Hit
TGGGAGACCGCCTGGGAATACCGGGTGCTGTAGGCT	12363	0.22931291632870077	No Hit
ACGGCCCTGGCGGAGCGCTGAGAAGACGGTCGAACT	11741	0.2177758594690023	No Hit
ACAAATGATGAATAACAAAGGGACTTAATACTG	11627	0.21566135065548847	No Hit
TTCAAGTAATCCAGGATAGGCT	11201	0.20775976508919983	No Hit
TGGAAGACTAGTGATTTTGTTGT	11133	0.20649847913026173	No Hit

Sequence	Count	Percentage	Possible Source
GCAAATGATGATAAACTGGATCTGACTGACTGTGCT	11065	0.20523719317132363	No Hit
TGAGGTAGTAGGTTGTATAGTT	11027	0.20453235690015234	No Hit
GCATTGGTGGTTCAGTGGTAGAATTCTCGCCT	10924	0.20262187963881964	No Hit
CTTAATGATGACTGTTTTTTTTGATTGCTTGAAGCA	10579	0.19622270822950136	No Hit
CTCACTGATGAGTACGTTCTGACTTTCGTTCTTCTG	10128	0.1878574145900737	No Hit
TGAAATGATGGCAAATCATCTTTCGGGACTGACCTG	9953	0.1846114580781007	No Hit
TAATACTGCCTGGTAATGATGAC	9950	0.184555813109324	No Hit
GGCTGGTCCGATGGTAGTGGGTTATCAGAACT	9791	0.18160662976415995	No Hit
AACTGTGATGAAAGATTTGGTCTGTATGTAAT	9275	0.17203569513457087	No Hit
TCTCCTACTTGGATAACTGTGGTAATTCTAGAGCTA	9210	0.17083005414440944	No Hit
TGAGGTAGTAGTTTGTACAGTT	9120	0.16916070508110903	No Hit
CTGACCTATGAATTGACAGCC	9028	0.16745425937195751	No Hit
CTGCAGTGATGACTTTCTTAGGACACCTTTGGATTT	8984	0.16663813316323287	No Hit
CACAGATGATGAACTTATTGACGGGCGGACAGAAAC	8804	0.16329943503663202	No Hit
TAACACTGTCTGGTAACGATGTT	8702	0.16140750609822488	No Hit
CTGAATGATGATATCCCACTAACTGAGCAGTCAGTA	8420	0.1561768790332169	No Hit
ACTCCATGATGAACACAAAATGACAAGCATATGGCT	8329	0.1544889816469909	No Hit
TAGGGTGATGAAAAAGAATCCTTAGGCGTGGTTGTG	7774	0.144194662423305	No Hit
TAGCTTATCAGACTGATGTTG	7737	0.14350837447505926	No Hit
TACCCTGTAGATCCGAATTTGTG	7618	0.1413011240469176	No Hit
TAATACTGCCGGGTAATGATGGA	7610	0.14115273746351314	No Hit
GAGAAGACGGTCGAACTTGACTATCTAGAGGAAGTA	7394	0.13714629971159212	No Hit
AATGGATTTTTGGAGCAGG	7369	0.13668259163845314	No Hit
CGGCCCTGGCGGAGCGCTGAGAAGACGGTCGAACTT	7321	0.13579227213802622	No Hit
GCAGCTGATGATACAGCTTCTTTCCCCATC	7223	0.13397453649132135	No Hit
TCGTACGACTCTTAGCGGTGGATCACTCGGCTCGTG	7023	0.13026487190620928	No Hit
TAATACTGCCTGGTAATGATGA	6944	0.12879955439509003	No Hit
TTGGTACTAGCAACGCACTTTT	6815	0.12640682073769277	No Hit
GACTCTTAGCGGTGGATCACTCGGCTCGTGCGTCGA	6707	0.12440360186173227	No Hit
TCAGATGATGAATTTAACTGTTCAACTGCTGAATGA	6705	0.12436650521588115	No Hit
CTGACCTATGAATTGACAGCCAT	6595	0.12232618969406954	No Hit
TGTAACAGCAACTCCATGTGGA	6503	0.12061974398491798	No Hit
TAATACTGTCTGGTAAAACCGT	6394	0.11859797678603194	No Hit
AGCAGCATTGTACAGGGCTATGA	6333	0.11746652908757274	No Hit
CGCGACCTCAGATCAGACG	6332	0.1174479807646472	No Hit
ATACATGATGATCTCACACAACTTGAACTCTCTCAC	6193	0.11486976387799432	No Hit
CTGACCTATGAATTGACAGCT	6126	0.11362702624198179	No Hit

Sequence	Count	Percentage	Possible Source
GCATTGGTGGTTCAGTGGTAGAATTCTCGCC	5973	0.11078913283437108	No Hit
CGCGACCTCAGATCAGACGG	5918	0.10976897507346527	No Hit
CGCGACCTCAGATCAGACGA	5756	0.10676414675952452	No Hit
AGACGTGGCGACCCGCTGAATTT	5746	0.10657866353026892	No Hit
ATATATGATGACTTAGCTTTTTTCCCCGAC	5667	0.10511334601914965	No Hit
TTCACAGTGGCTAAGTTCTGC	5493	0.10188593783010218	No Hit
TCCTACTTGGATAACTGTGGTAATTCTAGAGCTAAT	5403	0.10021658876680176	No Hit
ACCCTGTAGATCCGAATTTGTG	5403	0.10021658876680176	No Hit

Adapter Content

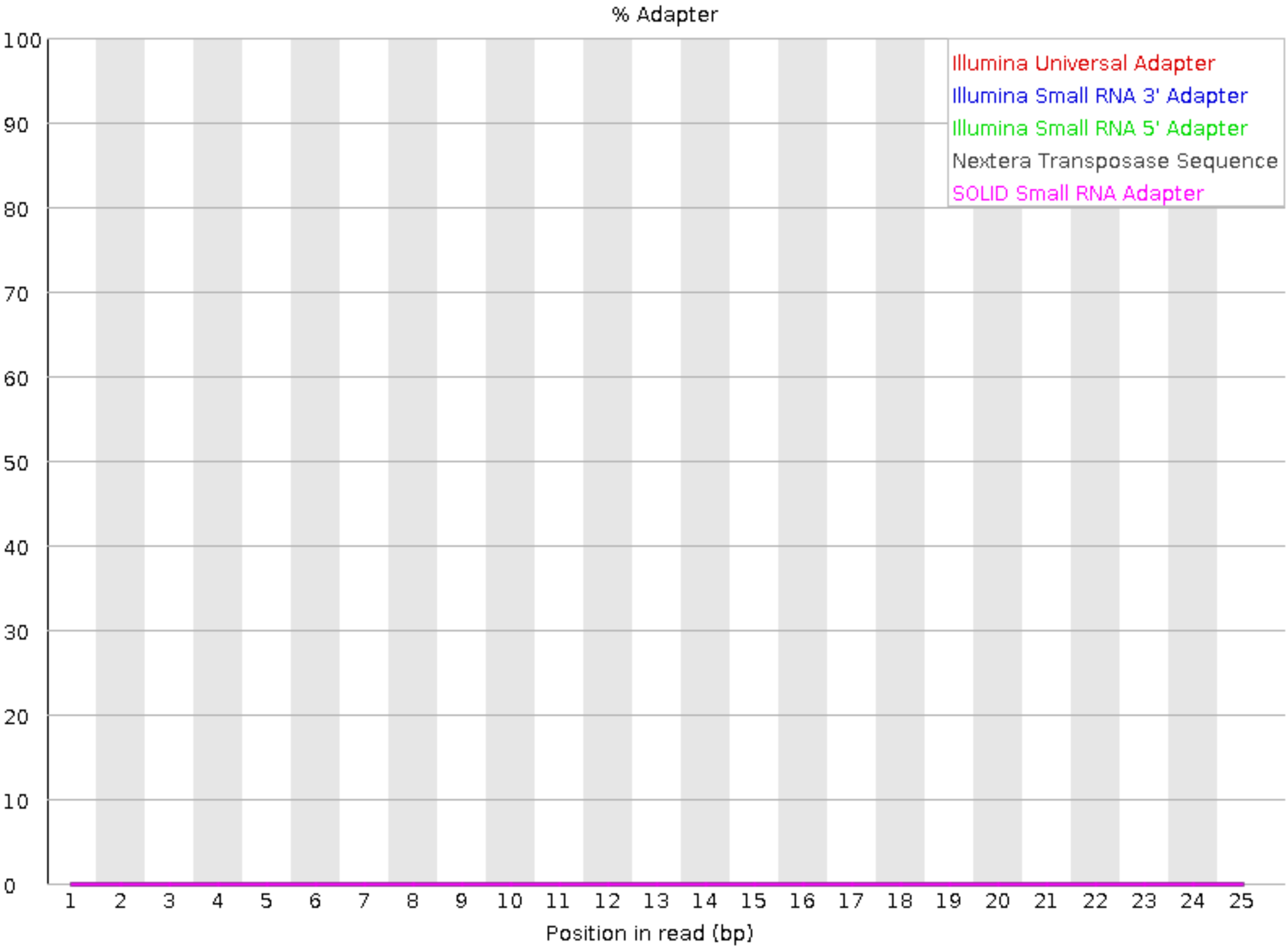

Produced by FastQC (version 0.11.9)
