# Supplementary material for: CD44 knockdown alters miRNA expression and their target genes in colon cancer
Source: Front Immunol. 2025 May 14;16:1552665. doi: 10.3389/fimmu.2025.1552665 (PMC12116639; doi:10.3389/fimmu.2025.1552665)

FastQC Report

Mon 31 Mar 2025
shCD44_5.fastq.gz

Summary

- Basic Statistics
- Per base sequence quality
- Per tile sequence quality
- Per sequence quality scores
- Per base sequence content
- Per sequence GC content
- Per base N content
- Sequence Length Distribution
- Sequence Duplication Levels
- Overrepresented sequences
- Adapter Content

Basic Statistics

Measure	Value
Filename	shCD44_5.fastq.gz
File type	Conventional base calls
Encoding	Sanger / Illumina 1.9
Total Sequences	8893467
Sequences flagged as poor quality	0
Sequence length	18-36
%GC	46

Per base sequence quality

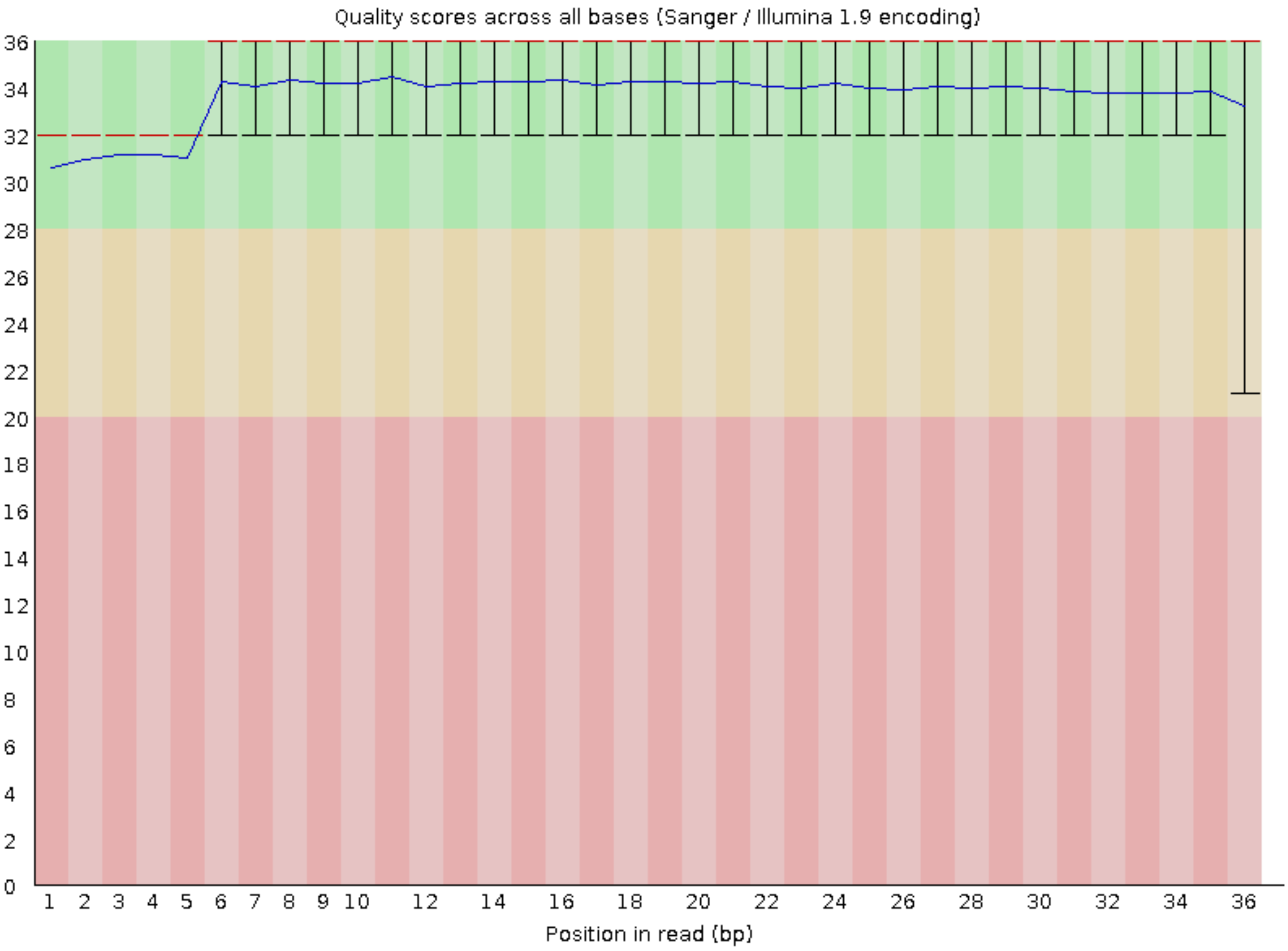

Per tile sequence quality

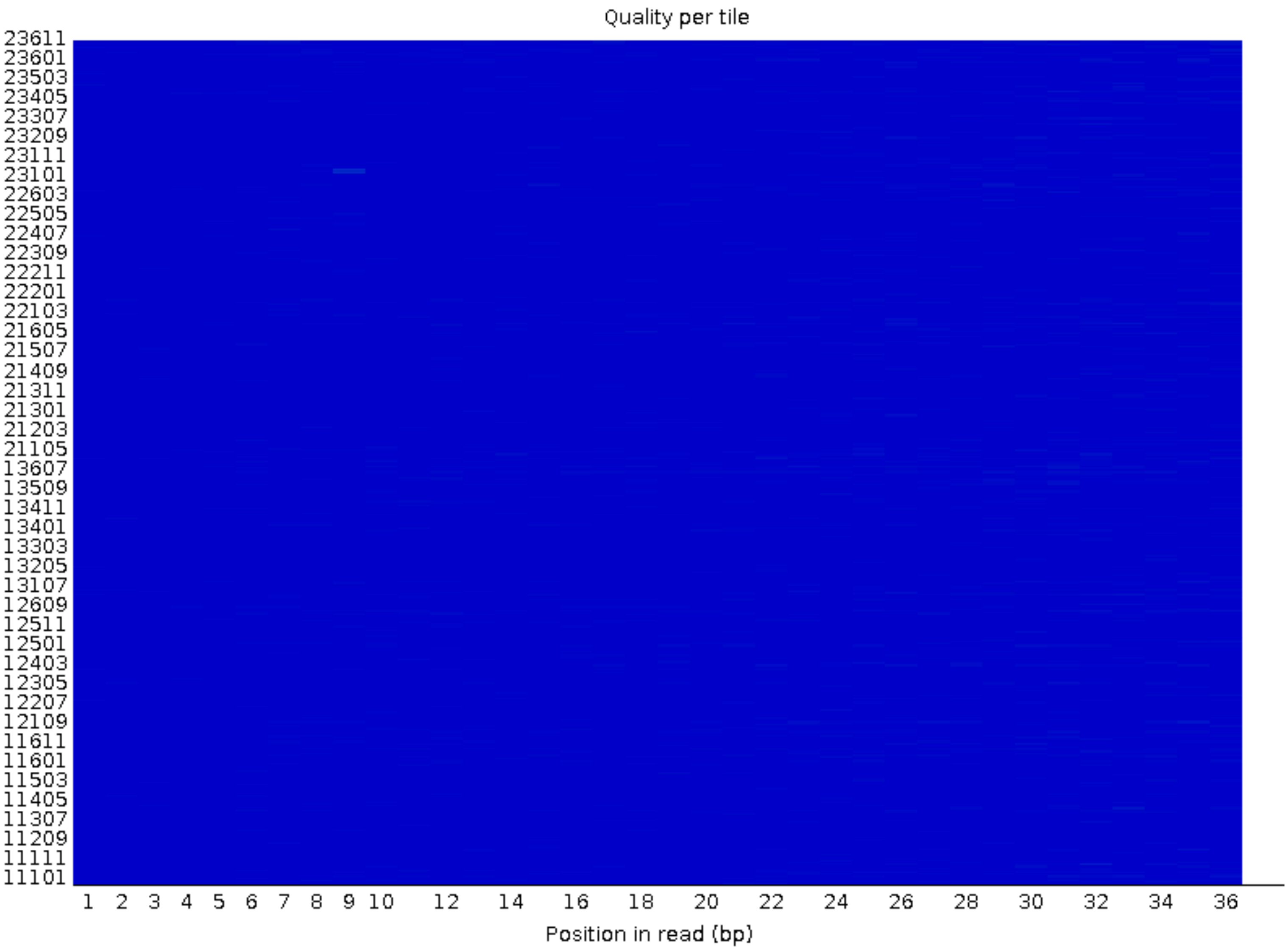

Per sequence quality scores

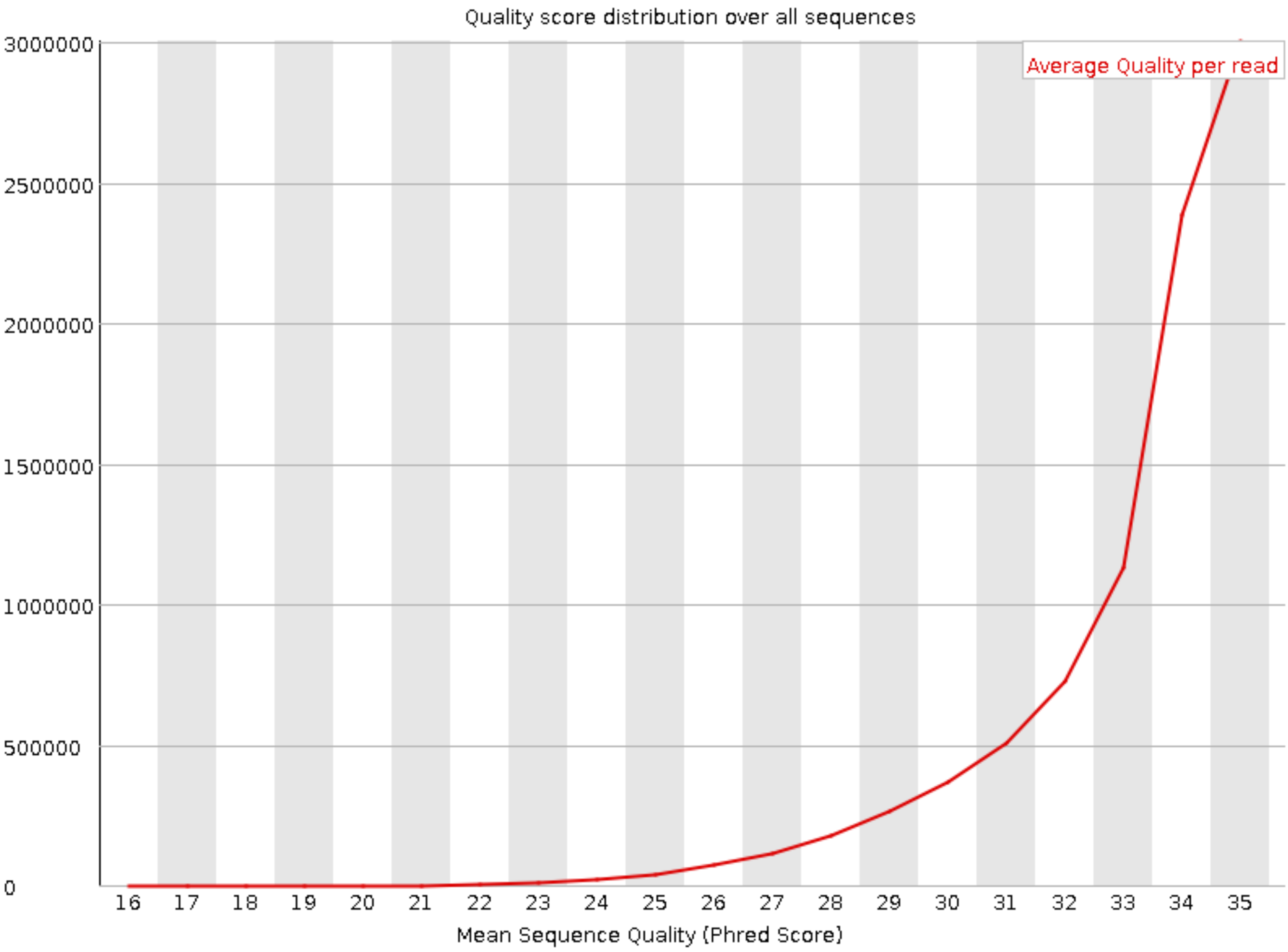

Per base sequence content

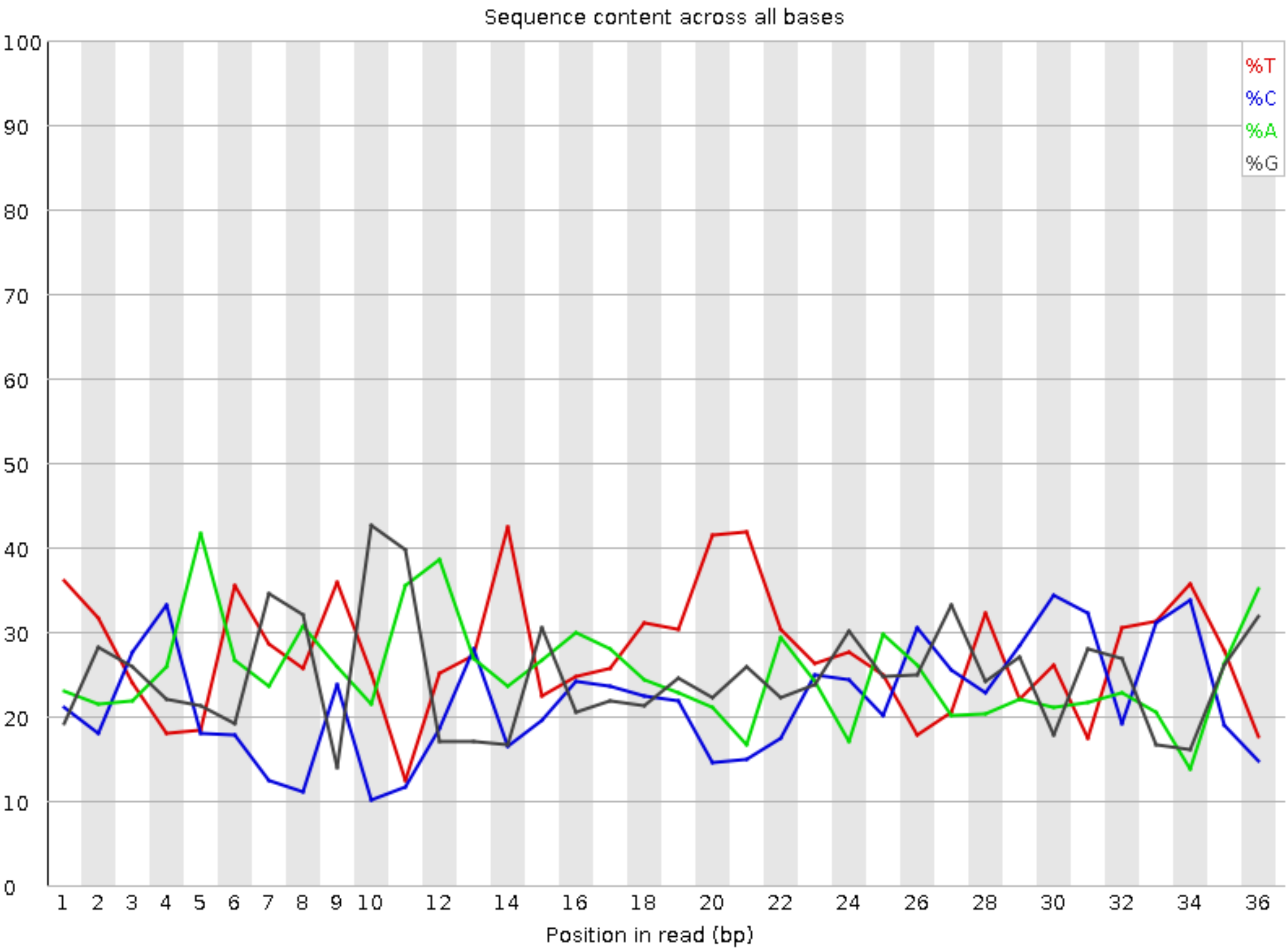

Per sequence GC content

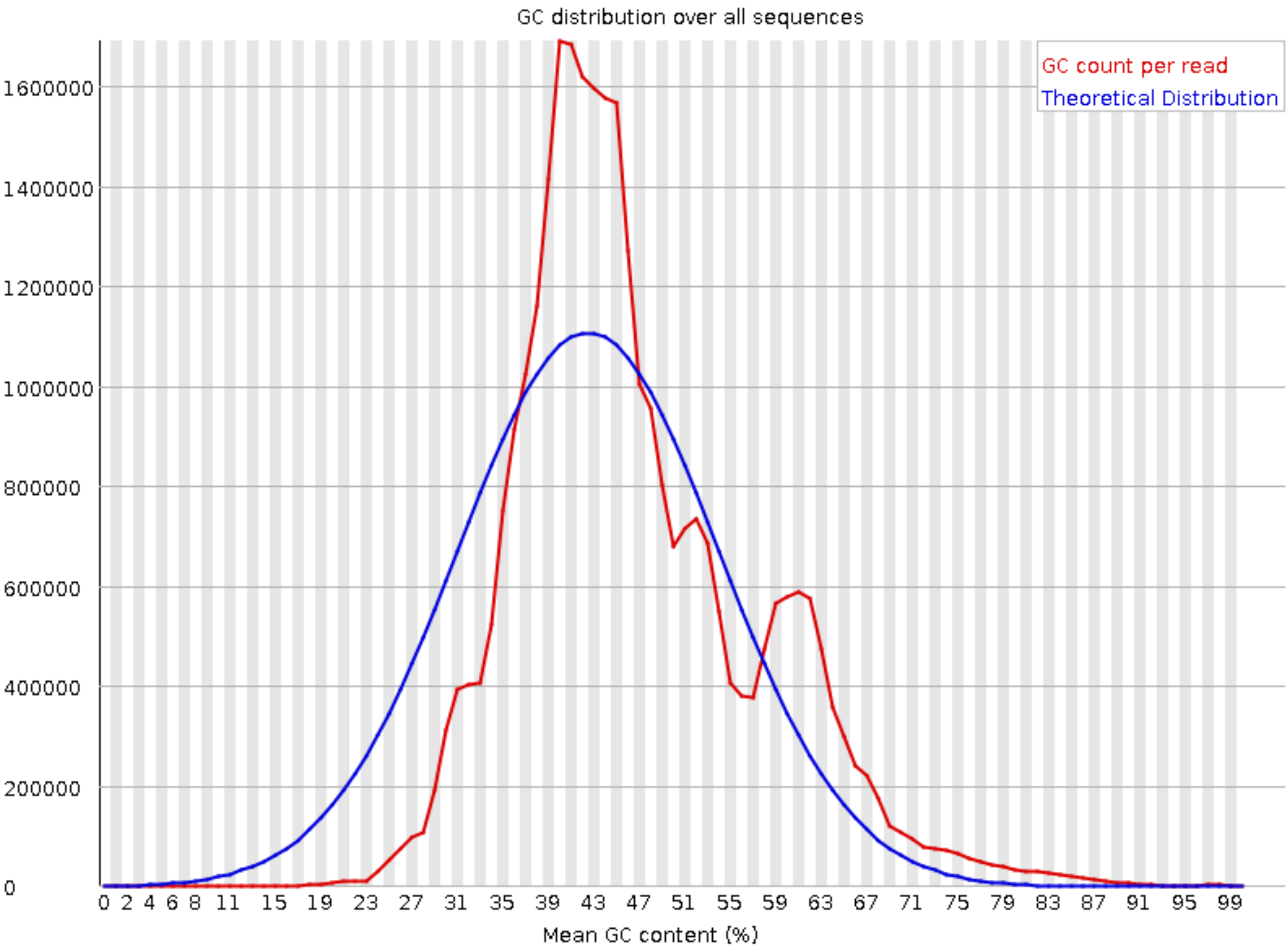

Per base N content

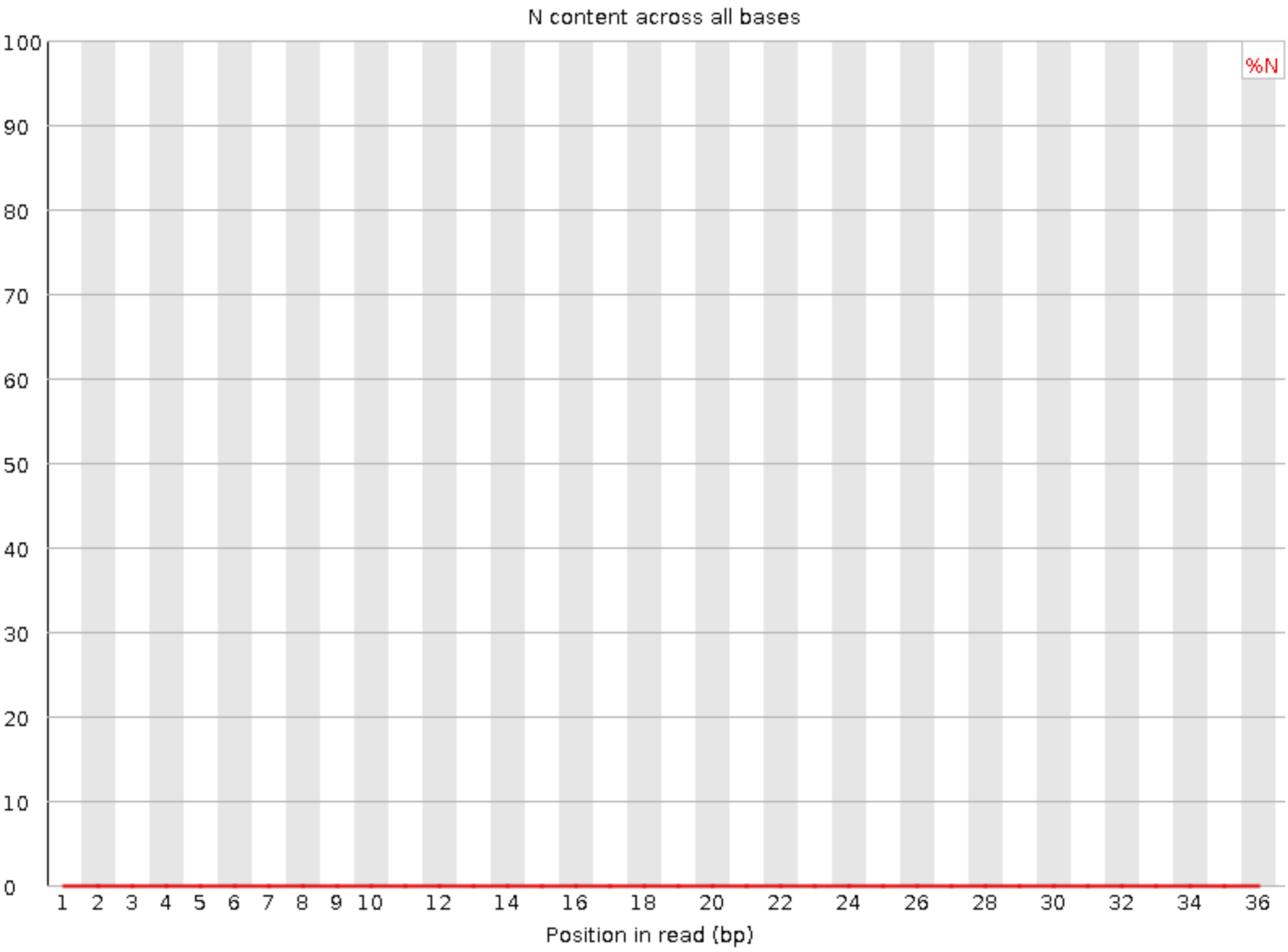

Sequence Length Distribution

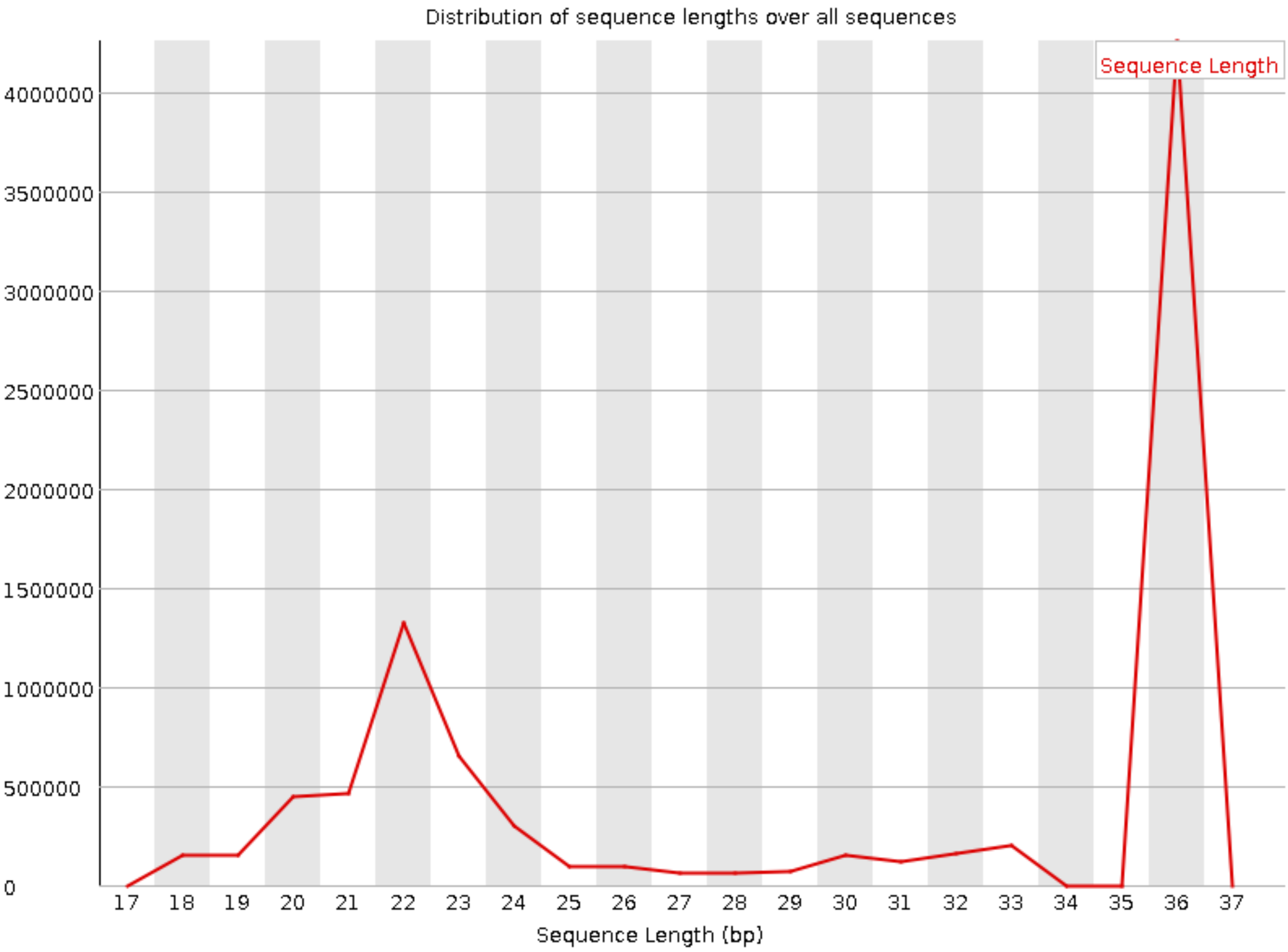

Sequence Duplication Levels

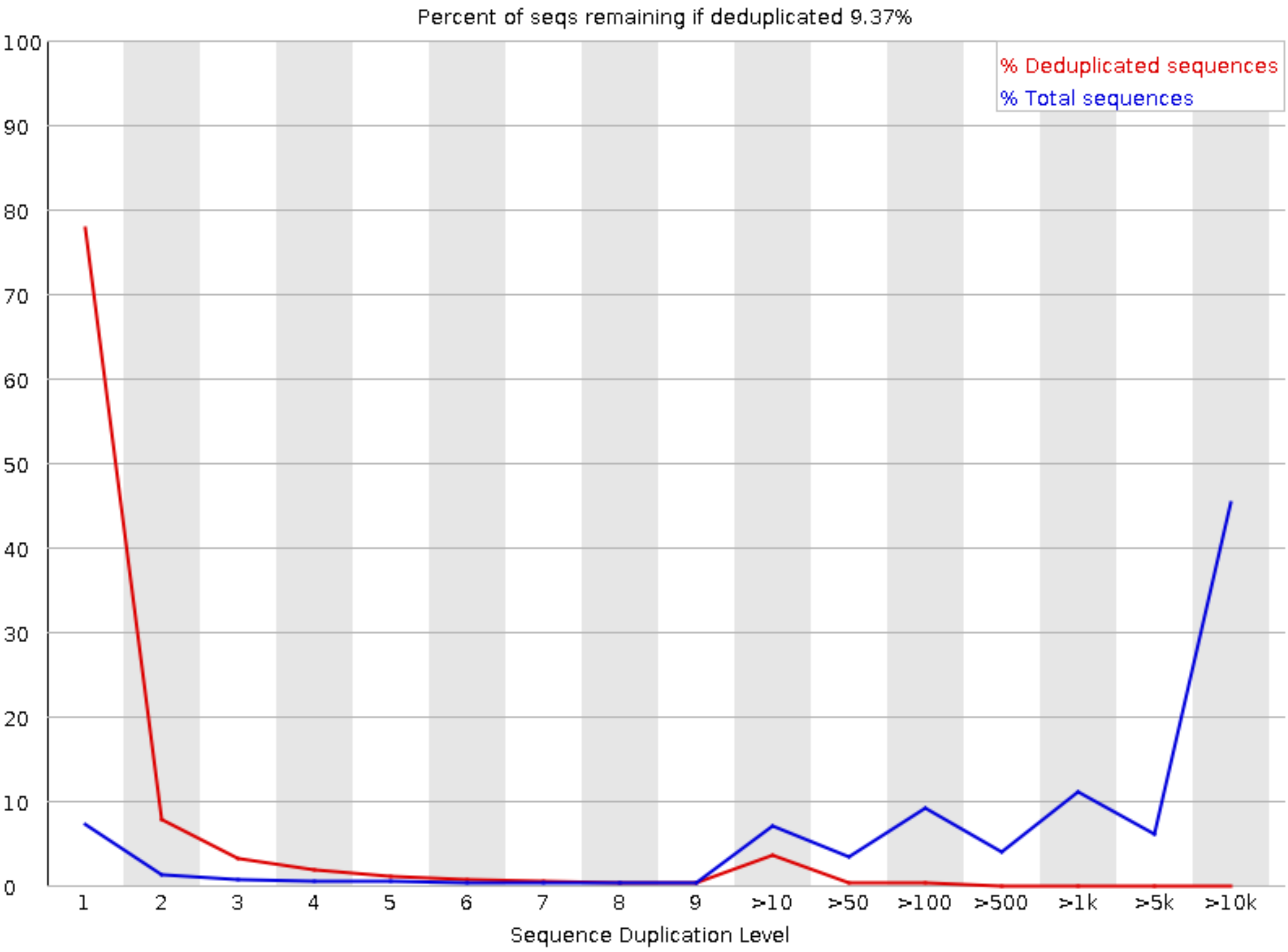

Overrepresented sequences

Sequence	Count	Percentage	Possible Source
TGCTCTGATGAAATCACTAATAGGAAGTGCCGTCAG	268949	3.0241187154570874	No Hit
ATTCAAATCGATCTGCGCCTTT	268222	3.0159441756516325	No Hit
CGCGACCTCAGATCAGACGT	182044	2.0469407487541136	No Hit
GTGAAATGATGGCAATCATCTTTCGGGACTGACCTG	162863	1.8312655795540704	No Hit
TAGCTTATCAGACTGATGTTGAC	146147	1.6433073850726607	No Hit
GTTTGTGATGACTTACATGGAATCTCGTTCGGCTGA	123248	1.385826247514046	No Hit
CCTGGATGATGATAAGCAAATGCTGACTGAACATGA	104480	1.1747949365528652	No Hit
GCCTCTGATGAAGCCTGTGTTGGTAGGGACATCTGA	98910	1.1121646934766836	No Hit
AGTAGTGATGAAATTCCACTTCATTGGTCCGTGTTT	95808	1.0772851577455678	No Hit
TTGAATGATGACTTTAATTGTCGGATACCCCTTCAC	91467	1.0284740472978648	No Hit
TAGCTTATCAGACTGATGTTGA	90566	1.0183430151593298	No Hit
GTGCAATGATGTATTTTATTCAACACATCATTCTGA	89238	1.0034107058585813	No Hit

Sequence	Count	Percentage	Possible Source
TATCTGTGATGATCTTATCCCGAACCTGAACTTCTG	77597	0.8725168710920048	No Hit
ATACATGATGATCTCAATCCAACTTGAACTCTCTCA	76537	0.8605980097525522	No Hit
TTTCTATGATGAATCAAACTAGCTCACTATGACCGA	66757	0.7506296475828831	No Hit
ATTCAAATCGAACTGCGCCTTT	64838	0.729052010874949	No Hit
TCGCTGCGATCTATTGAAAGTCAGCCCTCGACACAA	62498	0.7027405622576662	No Hit
TGAAATGATGGCAATCATCTTTCGGGACTGACCTGA	59792	0.6723137332156289	No Hit
TGGAAGACTAGTGATTTTGTTGTT	51929	0.5839005193362724	No Hit
CTACGGGGATGATTTTACGAACTGAACTCTCTCTTT	46857	0.5268698922478714	No Hit
ATTCAAATCGATCTGCGCCTTC	45340	0.5098124274818808	No Hit
ACAAATGATGAATAACAAAGGGACTTAATACTG	43919	0.49383440676172746	No Hit
GCAAATGATGATAAACTGGATCTGACTGACTGTGCT	42382	0.47655205781952076	No Hit
CTGGATGATGATAAGCAAATGCTGACTGAACATGAA	40762	0.45833643954601727	No Hit
CGACTCTTAGCGGTGGATCACTCGGCTCGTGCGTCG	39698	0.44637260137132123	No Hit
CTCGCTGCGATCTATTGAAAGTCAGCCCTCGACACA	39383	0.4428306755959178	No Hit
TTTGAATGATGACTTTAATTGTCGGATACCCCTTCA	37812	0.4251660235541437	No Hit
CGCTGCGATCTATTGAAAGTCAGCCCTCGACACAAG	37435	0.4209269568324704	No Hit
TAGCTTATCAGACTGATGTTGAT	34385	0.38663211995951635	No Hit
GCAGCTGATGATACAGCTTCTTTCCCCATC	33830	0.3803915840695198	No Hit
TGAGGTAGTAGATTGTATAGTT	33494	0.3766135299090894	No Hit
TGCCTCTGATGAAGCCTGTGTTGGTAGGGACATCTG	33019	0.3712725307239573	No Hit
CGCGACCTCAGATCAGACGC	32323	0.3634465613916372	No Hit
CTCACTGATGAGTACGTTCTGACTTTCGTTCTTCTG	31271	0.35161765372267084	No Hit
ACTCCATGATGAACACAAAATGACAAGCATATGGCT	29667	0.3335819427901402	No Hit
CTGCAGTGATGACTTTCTTAGGACACCTTTGGATTT	29580	0.33260369662360023	No Hit
TAGCTTATCAGACTGATGTTGACA	29098	0.32718398797679243	No Hit
TGAGGTAGTAGTTTGTGCTGTT	29040	0.32653182386576574	No Hit
TTCAAGTAATCCAGGATAGGCT	28486	0.32030253218458	No Hit
GAGAAGACGGTCGAACTTGACTATCT	26584	0.2989160470264296	No Hit
TTCAAATCGATCTGCGCCTTT	26361	0.2964085884616202	No Hit
TAGCTTATCAGACTGATGTTGACT	24820	0.2790812626841703	No Hit
ATATATGATGACTTAGCTTTTTTCCCCGAC	24047	0.2703894892734183	No Hit
TACCCTGTAGATCCGAATTTGT	23691	0.2663865509367719	No Hit
CTTAATGATGACTGTTTTTTTTGATTGCTTGAAGCA	23461	0.26380038291028685	No Hit
CTGAATGATGATATCCCACTAACTGAGCAGTCAGTA	23196	0.2608206675754236	No Hit
CACAGATGATGAACTTATTGACGGGCGGACAGAAAC	23155	0.2603596550141806	No Hit
TAACACTGTCTGGTAACGATGTT	23117	0.25993237507937006	No Hit
TAGGGTGATGAAAAAGAATCCTTAGGCGTGGTTGTG	22051	0.24794604848705237	No Hit

Sequence	Count	Percentage	Possible Source
ATTCAAATCGATCTGCGCCTT	21918	0.24645056871521534	No Hit
AACTGTGATGAAAGATTTGGTCTGTATGTAAT	21445	0.24113205794770476	No Hit
TGAGGTAGTAGGTTGTATAGTT	20876	0.23473410313435694	No Hit
CGCGACCTCAGATCAGACGTGGCGACCCGCTGAATT	20745	0.23326111178014153	No Hit
GGCTGGTCCGATGGTAGTGGGTTATCAGAACT	20394	0.22931439448754914	No Hit
CAGGACGGTGGCCATGGAAGTCGGAATCCGCTAAGG	20231	0.22748158845138797	No Hit
CTAGACTGAAGCTCCTTGAGG	20153	0.22660454016414522	No Hit
TAATACTGCCGGGTAATGATGGA	19824	0.2229051954653905	No Hit
ACCGGGTGCTGTAGGCTT	19181	0.21567516920004315	No Hit
CTGCTGTGATGACATTCCAATTAAAGCACGTGTTAG	18713	0.21041287947658657	No Hit
GCATATGATGGAAAAGTTTTAATCTCCTGACACTTG	18608	0.20923223755145212	No Hit
TACGGGGATGATTTTACGAACTGAACTCTCTCTTTC	18449	0.20744440835053415	No Hit
GTGAAATGATGGCAAATCATCTTTCGGGACTGACCT	18322	0.206016393831562	No Hit
TACAATGATGATAACATAGTTCAGCAGACTAACGCT	18304	0.20581399807296752	No Hit
TGGAAGACTAGTGATTTTGTTGT	18053	0.20299170166145553	No Hit
TAATACTGCCTGGTAATGATGAC	17308	0.1946147660974061	No Hit
TCGCGTGATGACATTCTCCGGAATCGCTGTACGGCC	17265	0.1941312651185415	No Hit
TAGCTTATCAGACTGATGTTG	17149	0.19282693689648817	No Hit
GCATTGGTGGTTCAGTGGTAGAATTCTCGCCT	16930	0.19036445516692196	No Hit
TCAGTGCACTACAGAACTTTGT	16733	0.18814934603119346	No Hit
CTCCTACTTGGATAACTGTGGTAATTCTAGAGCTAA	16635	0.1870474135677346	No Hit
TGAGGTAGTAGTTTGTACAGTT	16465	0.18513589806989783	No Hit
TTCAAATCGAACTGCGCCTTT	16369	0.18405645402406057	No Hit
AGAAATGAAGAAACTAAAATTGGTCTTAGTATTGAA	16108	0.1811217155244406	No Hit
GATGGGAGACCGCCTGGGAATACCGGGTGCTGTAGG	15910	0.17889536217990126	No Hit
TTCAAATCGATCTGCGCCTTTT	15524	0.1745550975789307	No Hit
TGAAATGATGGCAAATCATCTTTCGGGACTGACCTG	15383	0.17296966413660725	No Hit
AAGCTATGATGAATTTGATTGCATTGATCGTCTGAC	14816	0.16659419774088102	No Hit
TAATACTGCCTGGTAATGATGA	14755	0.16590830100342197	No Hit
GCTTAATGATGACTGTTTTTTTTGATTGCTTGAAGC	14680	0.1650649853426116	No Hit
ACCGGGTGCTGTAGGCTTT	14552	0.16362572661482863	No Hit
TTCCTATGATGAGGACCTTTTCACAGACCTGTACTG	14466	0.1626587246570994	No Hit
ATTCAAATCGATCTGCGCCTTA	14196	0.1596227882781822	No Hit
ACGGCCCTGGCGGAGCGCTGAGAAGACGGTCGAACT	14105	0.15859956527639896	No Hit
AGAAGACGGTCGAACTTGACTATCT	13522	0.15204419153969986	No Hit
CTCCATGATGAACACAAAATGACAAGCATATGGCTG	13383	0.15048124651499803	No Hit
TAATACTGTCTGGTAAAACCGT	13319	0.14976161715110653	No Hit

Sequence	Count	Percentage	Possible Source
CTGACCTATGAATTGACAGCC	13017	0.14636586609024355	No Hit
TGTAAACATCCCCGACTGGAAGC	12876	0.1447804326479201	No Hit
CGCGACCTCAGATCAGACG	12838	0.1443531527131095	No Hit
TACCCTGTAGATCCGAATTTGTG	12761	0.14348734863467758	No Hit
CTGACCTATGAATTGACAGCCAT	12680	0.1425765677210024	No Hit
TGTAACAGCAACTCCATGTGGA	12672	0.14248661405051594	No Hit
CTGCGATGATGGCATTTCTTAGGACACCTTTGGATT	12665	0.14240790458884034	No Hit
ACAGATGATGAACTTATTGACGGGCGGACAGAAACT	12263	0.13788773264689688	No Hit
TCAGATGATGAATTTAACTGTTCAACTGCTGAATGA	12133	0.1364259855014923	No Hit
TTCAAATCGAACTGCGCCTTTT	12000	0.13493050572965526	No Hit
ATACATGATGATCTCACACAACTTGAACTCTCTCAC	11796	0.1326366871322511	No Hit
AGTCTGTGATGAATTGCTTTGACTTCTGACACCTCG	11780	0.13245677979127826	No Hit
TGTAAACATCCCCGACTGGAAGCT	11348	0.12759928158501066	No Hit
TCTCCTACTTGGATAACTGTGGTAATTCTAGAGCTA	11051	0.12425975156820168	No Hit
GACTCTTAGCGGTGGATCACTCGGCTCGTGCGTCGA	10829	0.12176353721220308	No Hit
TGGGAGACCGCCTGGGAATACCGGGTGCTGTAGGCT	10795	0.12138123411263571	No Hit
TTCACAGTGGCTAAGTTCTGC	10658	0.11984077750555547	No Hit
TGTAAACATCCCCGACTGGAAG	10341	0.11627636331253041	No Hit
AATACATGATGATCTCAATCCAACTTGAACTCTCTC	10325	0.11609645597155756	No Hit
ATTCAAATCGAACTGCGCCTTC	10219	0.11490456983761226	No Hit
GTGTATGATGACAACTCGGTAATGCTGCATACTCCC	10177	0.11443231306755848	No Hit
CGCGACCTCAGATCAGACGA	10091	0.11346531110982927	No Hit
TCAAATGATGAAATCACCCAAAATAGCTGGAATTAC	10061	0.11312798484550513	No Hit
AGCAGCATTGTACAGGGCTATGA	9897	0.11128393460053318	No Hit
GTAGGGTGATGAAAAAGAATCCTTAGGCGTGGTTGT	9721	0.10930495384983156	No Hit
CCTCACTGATGAGTACGTTCTGACTTTCGTTCTTCT	9656	0.10857408027712928	No Hit
TTGCATGATGACTTGAATTGTCGGATACCCCTTCAC	9599	0.1079331603749134	No Hit
CTGACCTATGAATTGACAGCT	9571	0.10761832252821088	No Hit
TGCATATGATGGAAAAGTTTTAATCTCCTGACACTT	9386	0.10553814389821202	No Hit
TTGCTGTGATGACTATCTTAGGACACCTTTGGAATA	9369	0.10534699234842834	No Hit
TGGAAGACTAGTGATTTTGTTGTC	9292	0.1044811882699964	No Hit
GTTGAGGTCTATCCCGATGGGGCTTTTCCTGTAGCC	9283	0.10437999039069916	No Hit
TAAAGTGCTTATAGTGCAGGTAG	9249	0.10399768729113179	No Hit
TTCACAGTGGCTAAGTTCCG	9079	0.10208617179329502	No Hit
TCGTACGACTCTTAGCGGTGGATCACTCGGCTCGTG	8929	0.1003995404716743	No Hit

Adapter Content

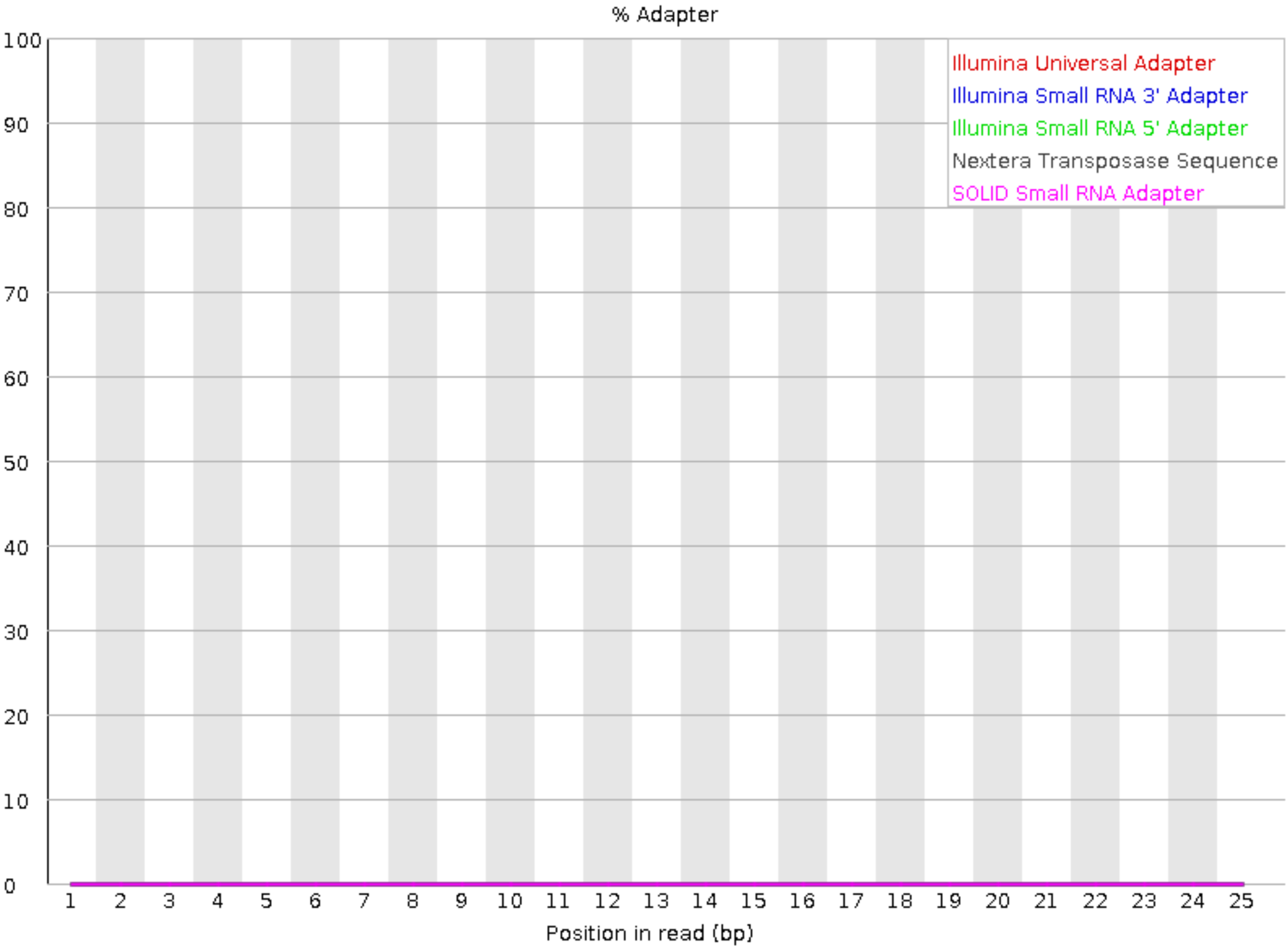

Supplement: Supplementary file 5 [file DataSheet5.zip › QC reports/shCD44_5.fastq.gz FastQC Report.pdf]
