# Supplementary material for: CD44 knockdown alters miRNA expression and their target genes in colon cancer
Source: Front Immunol. 2025 May 14;16:1552665. doi: 10.3389/fimmu.2025.1552665 (PMC12116639; doi:10.3389/fimmu.2025.1552665)

FastQC Report

Mon 31 Mar 2025
shCD44_2.fastq.gz

Summary

- Basic Statistics
- Per base sequence quality
- Per tile sequence quality
- Per sequence quality scores
- Per base sequence content
- Per sequence GC content
- Per base N content
- Sequence Length Distribution
- Sequence Duplication Levels
- Overrepresented sequences
- Adapter Content

Basic Statistics

Measure	Value
Filename	shCD44_2.fastq.gz
File type	Conventional base calls
Encoding	Sanger / Illumina 1.9
Total Sequences	22048418
Sequences flagged as poor quality	0
Sequence length	18-36
%GC	44

Per base sequence quality

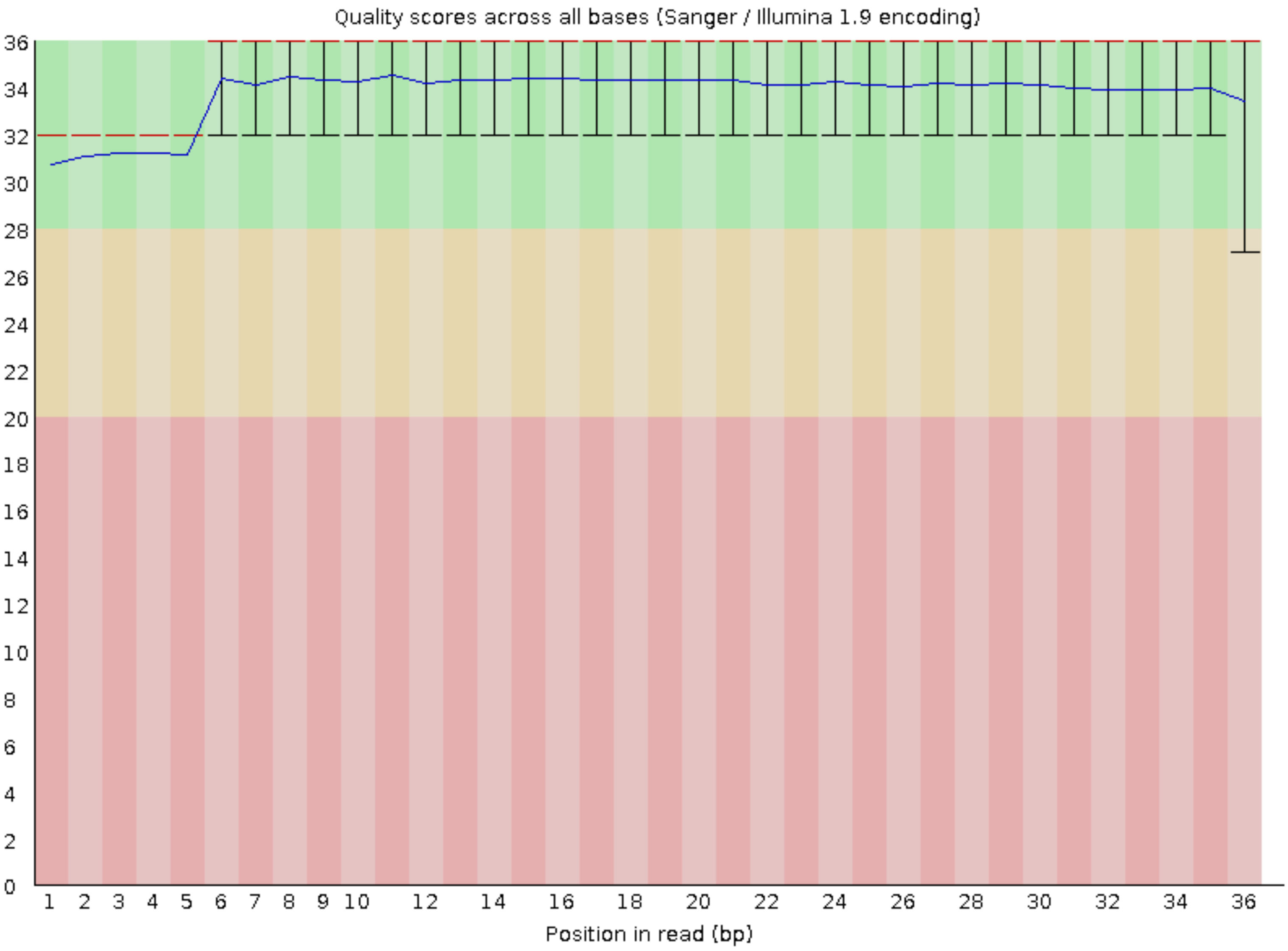

Per tile sequence quality

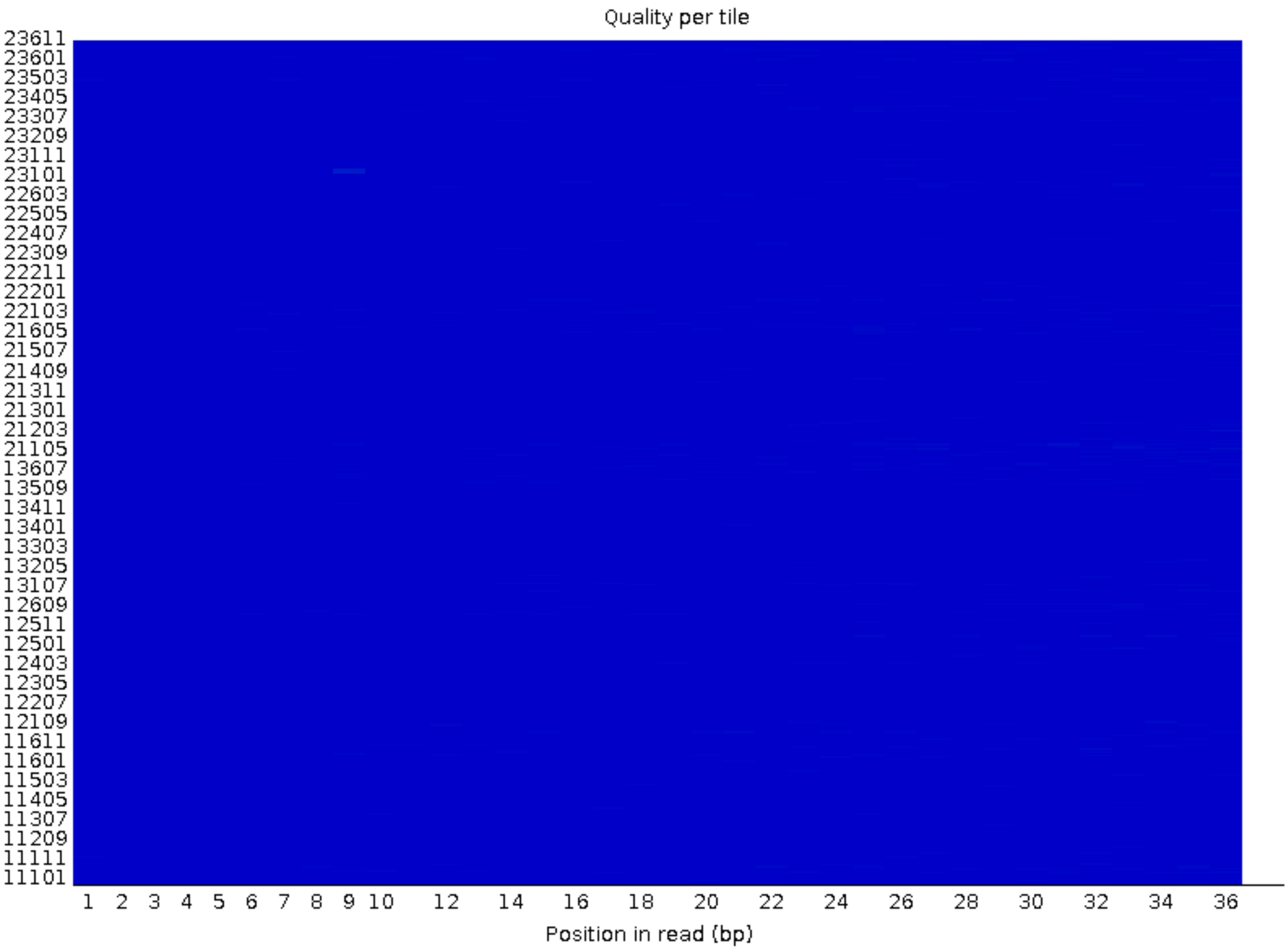

Per sequence quality scores

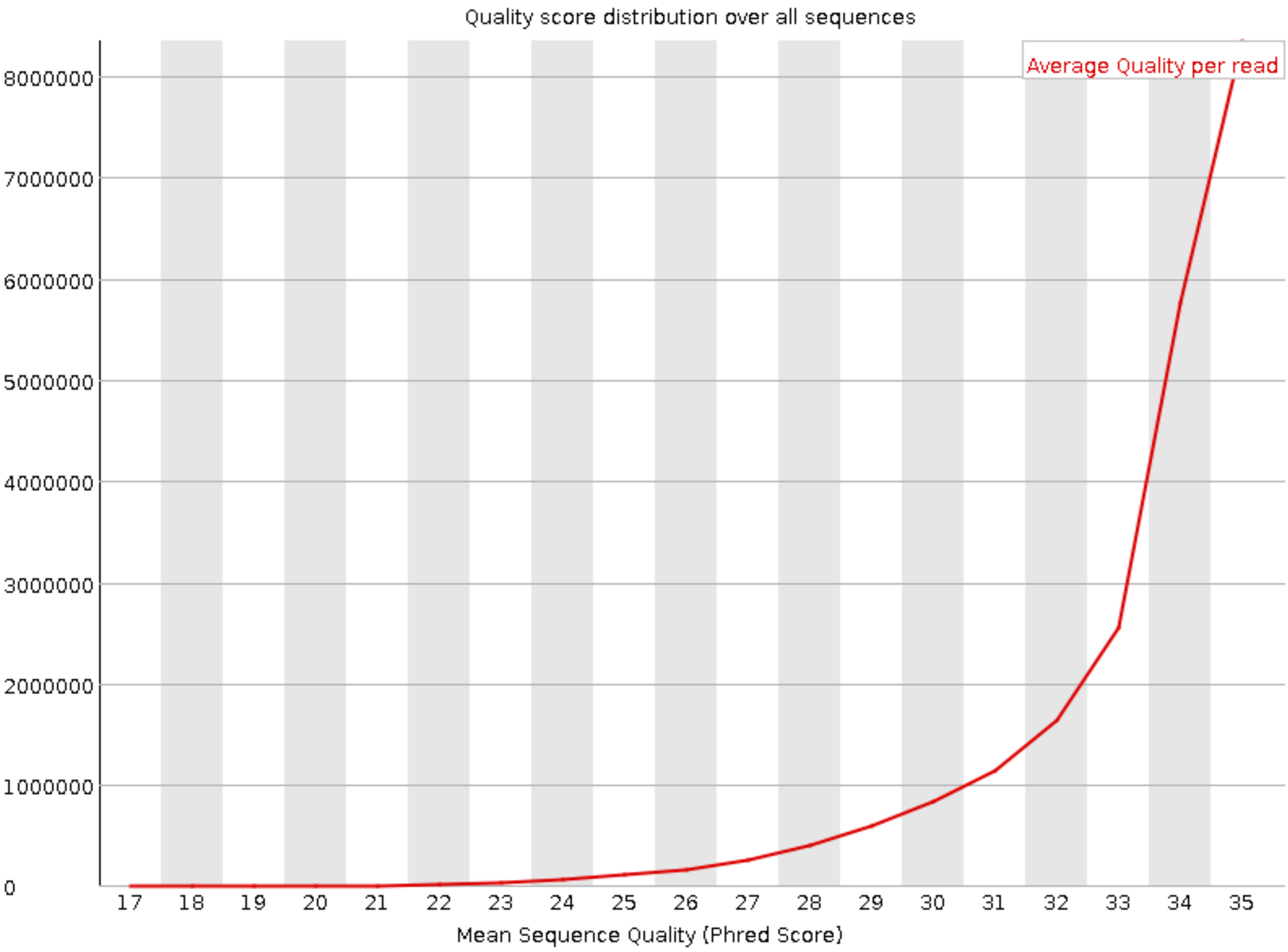

Per base sequence content

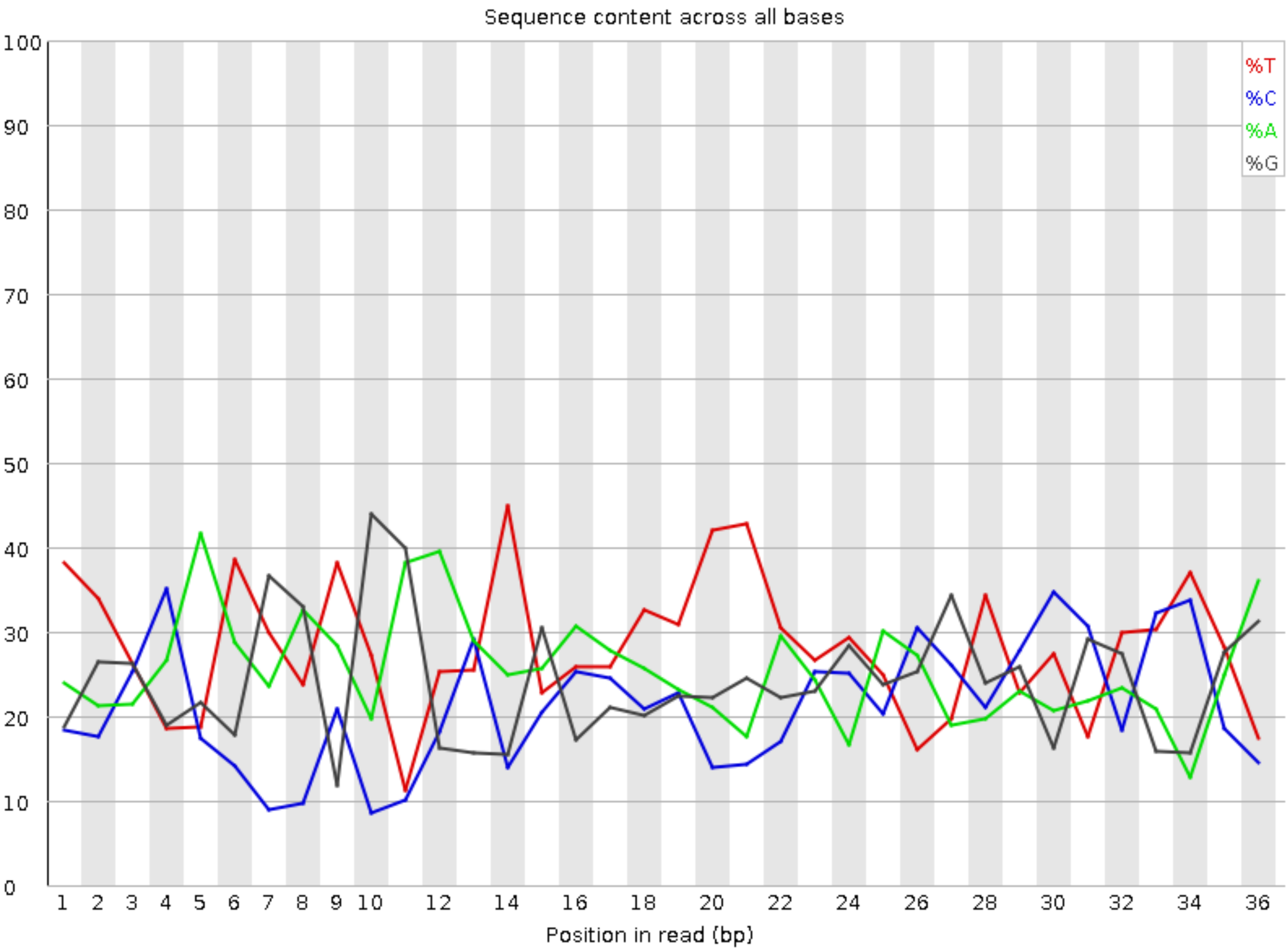

Per sequence GC content

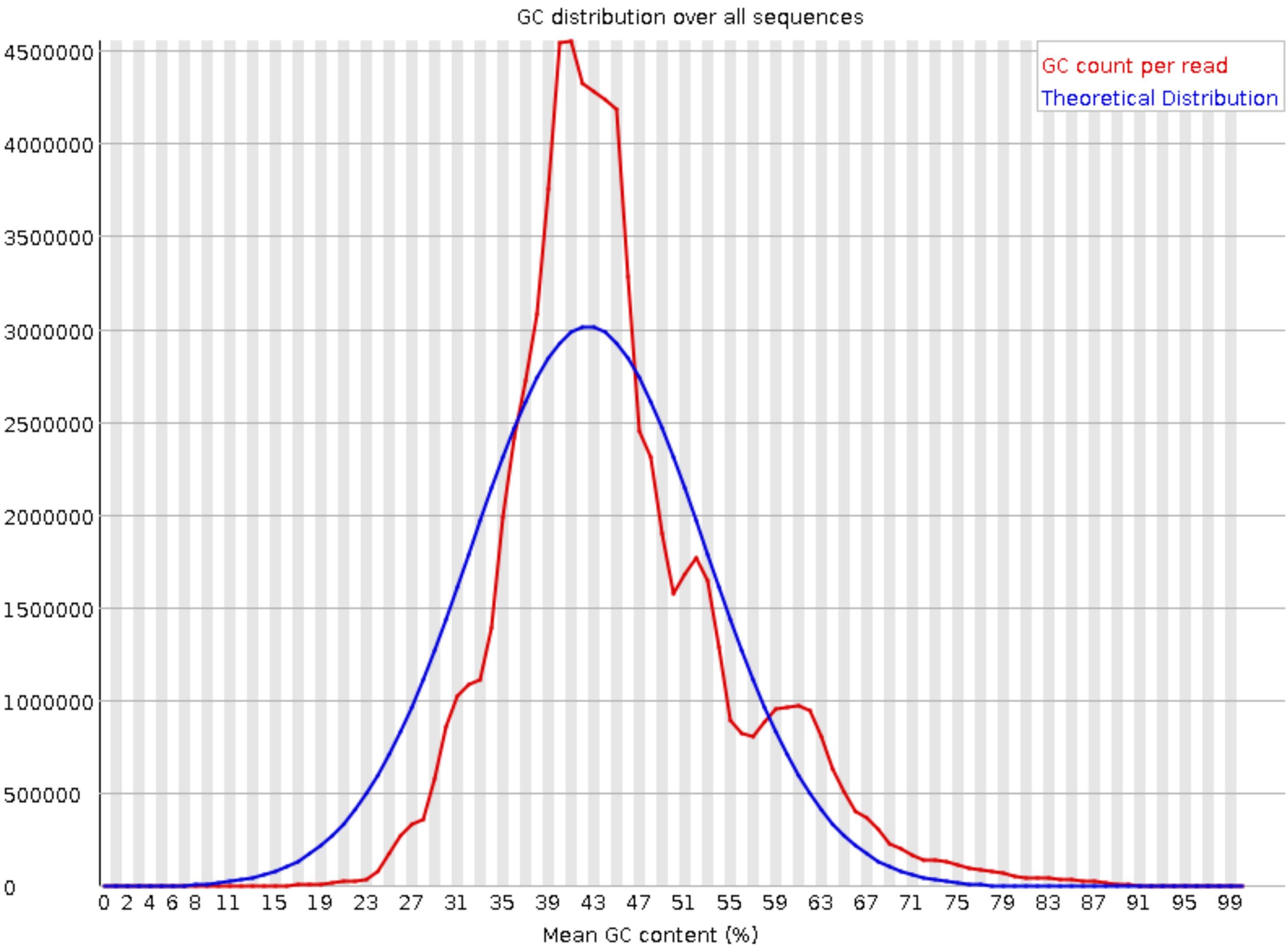

Per base N content

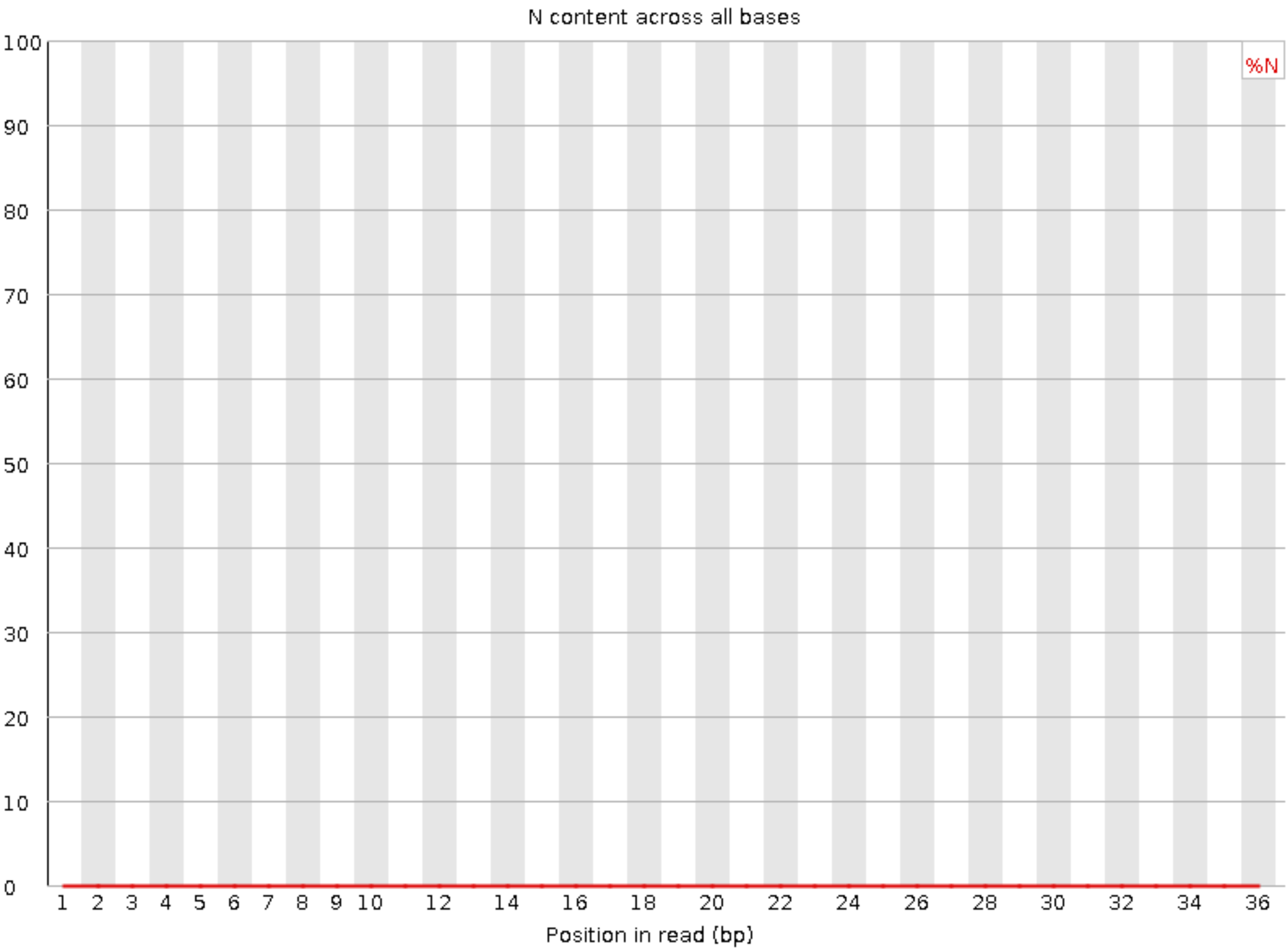

Sequence Length Distribution

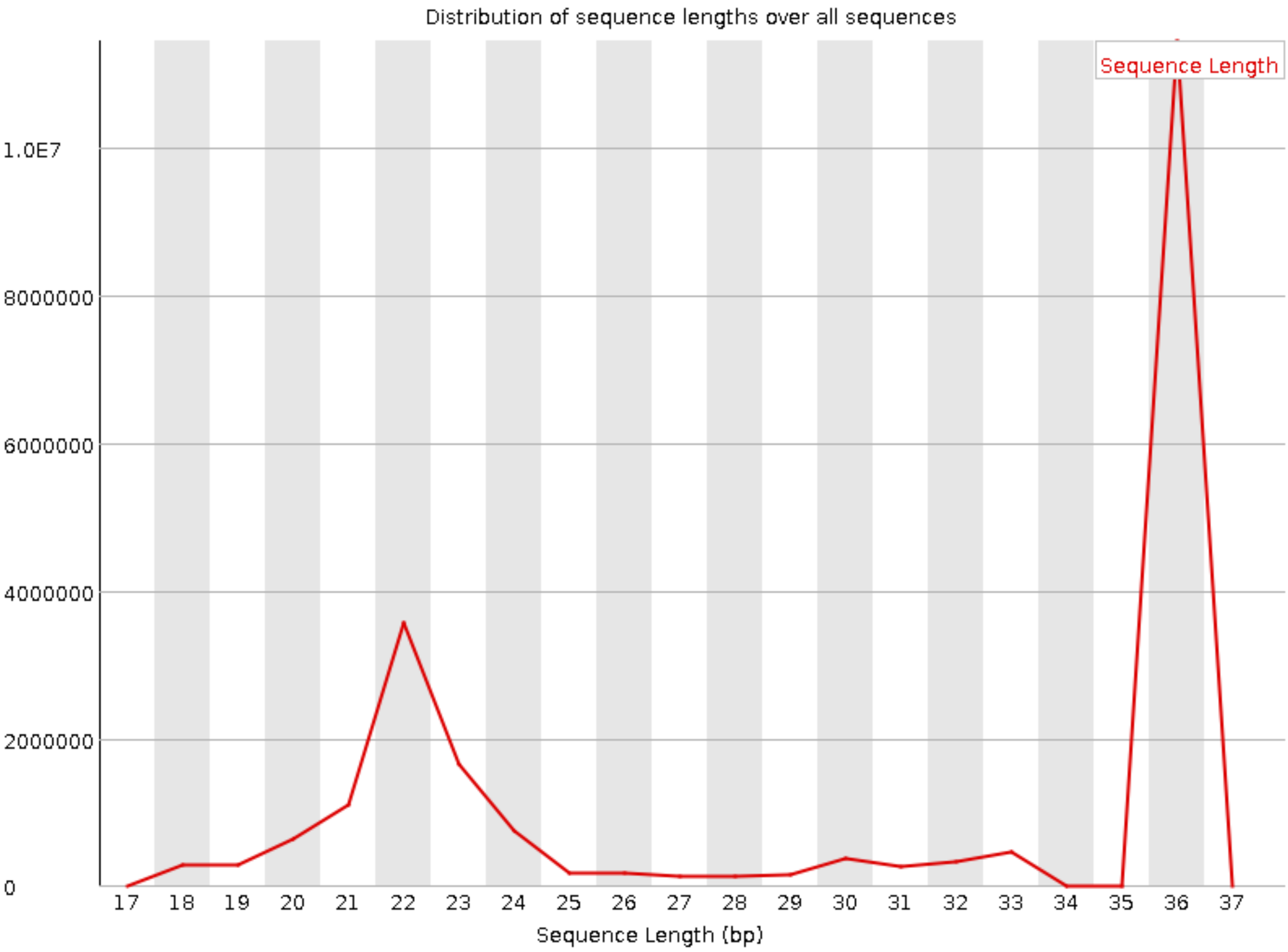

Sequence Duplication Levels

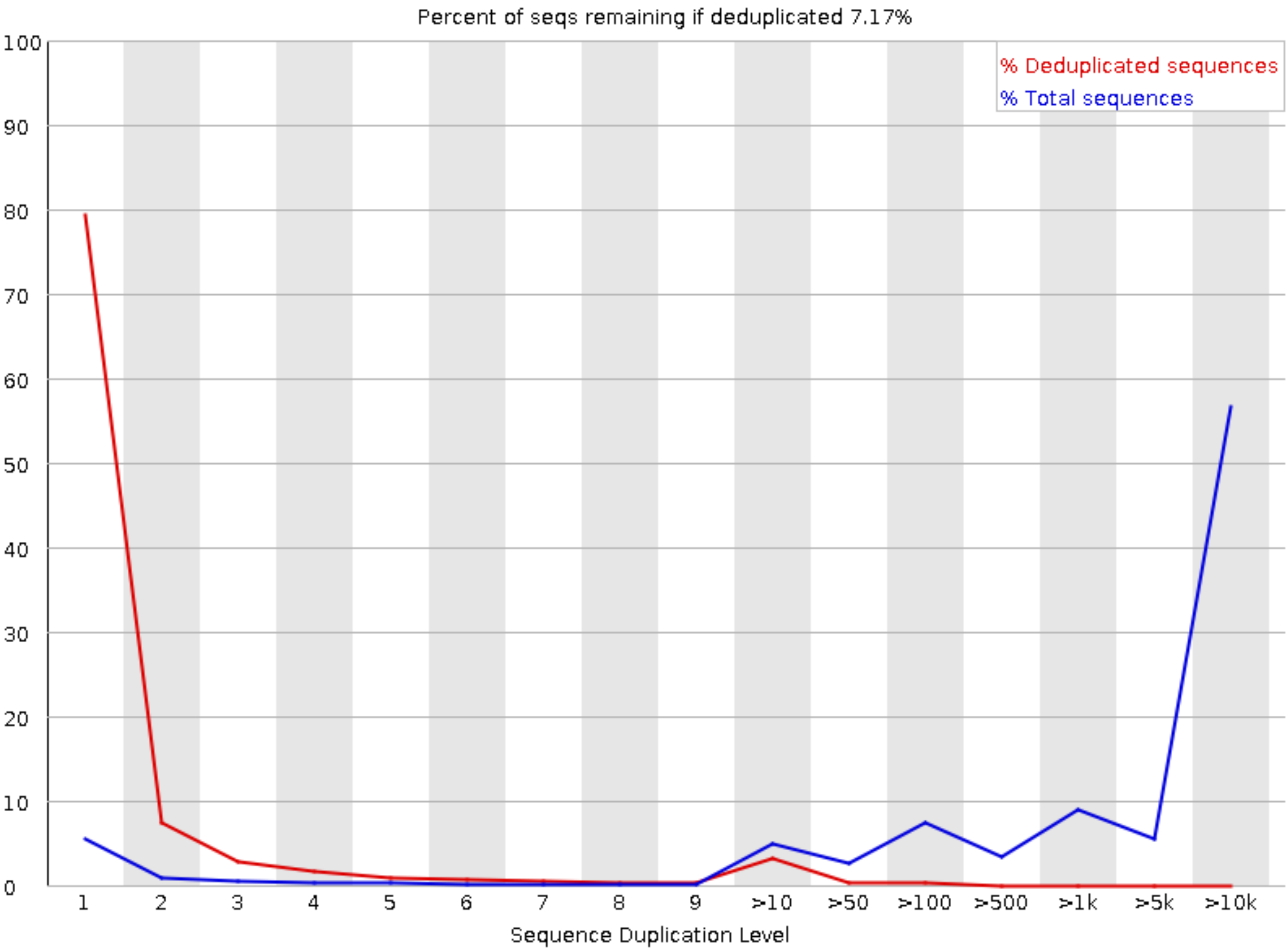

Overrepresented sequences

Sequence	Count	Percentage	Possible Source
ATTCAAATCGATCTGCGCCTTT	762598	3.4587424821136823	No Hit
TGCTCTGATGAAATCACTAATAGGAAGTGCCGTCAG	719763	3.2644655049627596	No Hit
GTTTGTGATGACTTACATGGAATCTCGTTCGGCTGA	442157	2.0053910443824132	No Hit
TAGCTTATCAGACTGATGTTGAC	428337	1.9427108103629025	No Hit
GTGAAATGATGGCAATCATCTTTCGGGACTGACCTG	416510	1.8890697736227604	No Hit
CCTGGATGATGATAAGCAAATGCTGACTGAACATGA	310805	1.4096476218838014	No Hit
AGTAGTGATGAAATTCCACTTCATTGGTCCGTGTTT	273180	1.2390004579920428	No Hit
GCCTCTGATGAAGCCTGTGTTGGTAGGGACATCTGA	268879	1.2194933895030473	No Hit
TAGCTTATCAGACTGATGTTGA	248959	1.1291467714372976	No Hit
TTTCTATGATGAATCAAACTAGCTCACTATGACCGA	241505	1.0953393572273529	No Hit
GTGCAATGATGTATTTTATTCAACACATCATTCTGA	233228	1.0577992489075634	No Hit
TATCTGTGATGATCTTATCCCGAACCTGAACTTCTG	220298	0.9991555856751264	No Hit

Sequence	Count	Percentage	Possible Source
ATACATGATGATCTCAATCCAACTTGAACTCTCTCA	209665	0.9509299034515764	No Hit
ATTCAAATCGAACTGCGCCTTT	190844	0.8655677699869443	No Hit
TTGAATGATGACTTTAATTGTCGGATACCCCTTCAC	180525	0.8187662262208563	No Hit
CGCGACCTCAGATCAGACGT	152635	0.6922718899832179	No Hit
TGGAAGACTAGTGATTTTGTTGTT	140915	0.639116148832084	No Hit
TCGCTGCGATCTATTGAAAGTCAGCCCTCGACACAA	140582	0.6376058363915271	No Hit
TGAAATGATGGCAATCATCTTTCGGGACTGACCTGA	131693	0.5972900187215245	No Hit
CTACGGGGATGATTTTACGAACTGAACTCTCTCTTT	131540	0.5965960913839714	No Hit
GCAAATGATGATAAACTGGATCTGACTGACTGTGCT	128841	0.5843548503117094	No Hit
CTGGATGATGATAAGCAAATGCTGACTGAACATGAA	114895	0.5211031467200957	No Hit
CTCGCTGCGATCTATTGAAAGTCAGCCCTCGACACA	111304	0.5048162639151706	No Hit
ATTCAAATCGATCTGCGCCTTC	108730	0.49314195694221696	No Hit
ACAAATGATGAATAACAAAGGGACTTAATACTG	104564	0.4742471772804743	No Hit
ACTCCATGATGAACACAAAATGACAAGCATATGGCT	100789	0.4571257674813676	No Hit
TGCCTCTGATGAAGCCTGTGTTGGTAGGGACATCTG	100582	0.4561869246129132	No Hit
CTGCAGTGATGACTTTCTTAGGACACCTTTGGATTT	86445	0.39206894571755674	No Hit
CGACTCTTAGCGGTGGATCACTCGGCTCGTGCGTCG	84868	0.38491650512068487	No Hit
GTGAAATGATGGCAAATCATCTTTCGGGACTGACCT	82514	0.3742400021625134	No Hit
CGCTGCGATCTATTGAAAGTCAGCCCTCGACACAAG	81406	0.3692146983062458	No Hit
TGAGGTAGTAGATTGTATAGTT	80924	0.36702860041931357	No Hit
TAGCTTATCAGACTGATGTTGACA	79365	0.35995779833274205	No Hit
TGAGGTAGTAGTTTGTGCTGTT	77723	0.35251055200423	No Hit
CTCACTGATGAGTACGTTCTGACTTTCGTTCTTCTG	76998	0.3492223342282426	No Hit
TGGAATGTAAAGAAGTATGTAT	76581	0.3473310420729505	No Hit
GCAGCTGATGATACAGCTTCTTTCCCCATC	70696	0.320639784677522	No Hit
TTTGAATGATGACTTTAATTGTCGGATACCCCTTCA	69973	0.31736063784712354	No Hit
ATATATGATGACTTAGCTTTTTTCCCCGAC	69195	0.3138320400130295	No Hit
CTGAATGATGATATCCCACTAACTGAGCAGTCAGTA	68363	0.31005852664803435	No Hit
CACAGATGATGAACTTATTGACGGGCGGACAGAAAC	68024	0.3085210013707106	No Hit
TAGCTTATCAGACTGATGTTGACT	65985	0.29927317234279577	No Hit
TAGCTTATCAGACTGATGTTGAT	63588	0.2884016440544623	No Hit
TGAAATGATGGCAAATCATCTTTCGGGACTGACCTG	63212	0.2866963062837433	No Hit
TTCAAGTAATCCAGGATAGGCT	63100	0.2861883333307632	No Hit
TTCAAATCGATCTGCGCCTTT	62053	0.281439693314958	No Hit
CAGGACGGTGGCCATGGAAGTCGGAATCCGCTAAGG	61632	0.2795302592684881	No Hit
TAGGGTGATGAAAAAGAATCCTTAGGCGTGGTTGTG	60978	0.2765640600609078	No Hit
TGGAAGACTAGTGATTTTGTTGT	60855	0.27600619690718853	No Hit

Sequence	Count	Percentage	Possible Source
TCGCGTGATGACATTCTCCGGAATCGCTGTACGGCC	59825	0.2713346599288892	No Hit
TGAGGTAGTAGGTTGTATAGTT	57212	0.2594834695169513	No Hit
TACCCTGTAGATCCGAATTTGT	55330	0.25094770971776753	No Hit
TTCCTATGATGAGGACCTTTTCACAGACCTGTACTG	54366	0.2465755139439029	No Hit
CTTAATGATGACTGTTTTTTTTGATTGCTTGAAGCA	54203	0.2458362318784051	No Hit
CTAGACTGAAGCTCCTTGAGG	52631	0.23870646864550554	No Hit
ATTCAAATCGATCTGCGCCTT	52216	0.2368242474358024	No Hit
TAACACTGTCTGGTAACGATGTT	52155	0.23654758359534003	No Hit
AACTGTGATGAAAGATTTGGTCTGTATGTAAT	52061	0.2361212491526603	No Hit
GCATATGATGGAAAAGTTTTAATCTCCTGACACTTG	51585	0.2339623641024948	No Hit
CTGCTGTGATGACATTCCAATTAAAGCACGTGTTAG	50827	0.23052447572429005	No Hit
TACAATGATGATAACATAGTTCAGCAGACTAACGCT	48887	0.22172565850302728	No Hit
TCAGTGCACTACAGAACTTTGT	47171	0.21394278718772478	No Hit
TGAGGTAGTAGTTTGTACAGTT	46249	0.2097610812712277	No Hit
AAGCTATGATGAATTTGATTGCATTGATCGTCTGAC	46019	0.2087179225285007	No Hit
ATACATGATGATCTCACACAACTTGAACTCTCTCAC	45983	0.20855464550789993	No Hit
TACGGGGATGATTTTACGAACTGAACTCTCTCTTTC	45554	0.20660892767907427	No Hit
GCTTAATGATGACTGTTTTTTTTGATTGCTTGAAGC	44866	0.20348852239648213	No Hit
TAATACTGCCGGGTAATGATGGA	42913	0.19463074402889133	No Hit
TTCAAATCGATCTGCGCCTTTT	42434	0.19245825256034244	No Hit
AGAAATGAAGAAACTAAAATTGGTCTTAGTATTGAA	41833	0.18973243341086876	No Hit
TGCTATGATGAAGGCTATGTTGGTAGGGACAACTGA	41388	0.1877141480173317	No Hit
TAGCTTATCAGACTGATGTTG	41361	0.1875916902518811	No Hit
TAATACTGCCTGGTAATGATGAC	41090	0.186362577124581	No Hit
CTCCATGATGAACACAAAATGACAAGCATATGGCTG	40871	0.1853693085825931	No Hit
TTCAAATCGAACTGCGCCTTT	40590	0.18409484072734833	No Hit
TTCACAGTGGCTAAGTTCTGC	40259	0.1825935992323803	No Hit
TCAGATGATGAATTTAACTGTTCAACTGCTGAATGA	40230	0.1824620705213408	No Hit
ACCGGGTGCTGTAGGCTT	39011	0.17693332918488755	No Hit
AGTCTGTGATGAATTGCTTTGACTTCTGACACCTCG	37285	0.16910510314164037	No Hit
ACAGATGATGAACTTATTGACGGGCGGACAGAAACT	36312	0.1646920881126256	No Hit
CTGCGATGATGGCATTTCTTAGGACACCTTTGGATT	36310	0.16468301716703665	No Hit
TAATACTGTCTGGTAAAACCGT	35095	0.15917241772176127	No Hit
ATTCAAATCGATCTGCGCCTTA	34256	0.15536715604720483	No Hit
GGCTGGTCCGATGGTAGTGGGTTATCAGAACT	34098	0.15465055134567932	No Hit
GCATTGGTGGTTCAGTGGTAGAATTCTCGCCT	33926	0.15387045002503127	No Hit
GAGAAGACGGTCGAACTTGACTATCT	33745	0.15304952944923306	No Hit

Sequence	Count	Percentage	Possible Source
TTCAAATCGAACTGCGCCTTTT	33041	0.14985655660192945	No Hit
ACCGGGTGCTGTAGGCTTT	32942	0.14940754479527737	No Hit
TGTAACAGCAACTCCATGTGGA	32926	0.14933497723056594	No Hit
CTGACCTATGAATTGACAGCC	32556	0.14765685229661377	No Hit
TACCCTGTAGATCCGAATTTGTG	32300	0.14649577126123062	No Hit
AGCAGCATTGTACAGGGCTATGA	32087	0.14552971555600952	No Hit
AATACATGATGATCTCAATCCAACTTGAACTCTCTC	31463	0.14269958053226314	No Hit
TGTAAACATCCCCGACTGGAAGC	31005	0.140622333992398	No Hit
TCAAATGATGAAATCACCCAAAATAGCTGGAATTAC	30961	0.14042277318944155	No Hit
CACCAGTGATGAGTTGAATACCGCCCCAGTCTGATC	30842	0.13988305192690015	No Hit
GTTGAGGTCTATCCCGATGGGGCTTTTCCTGTAGCC	30801	0.13969709754232706	No Hit
CTCCTACTTGGATAACTGTGGTAATTCTAGAGCTAA	30123	0.13662204698767957	No Hit
TCTCGTGATGAAAACTCTGTCCAGTTCTGCTACTGA	29965	0.13590544228615403	No Hit
TGCATATGATGGAAAAGTTTTAATCTCCTGACACTT	29821	0.13525233420375102	No Hit
TAAAGTGCTTATAGTGCAGGTAG	29818	0.13523872778536764	No Hit
TGGAATGATGACATTCTCCGGAATCGCTGTACTGAC	29623	0.1343543105904469	No Hit
TAATACTGCCTGGTAATGATGA	29547	0.13400961465806754	No Hit
GTAGGGTGATGAAAAAGAATCCTTAGGCGTGGTTGT	29287	0.13283039173150654	No Hit
CGCGACCTCAGATCAGACGTGGCGACCCGCTGAATT	28954	0.1313200792909496	No Hit
TGTAAACATCCCCGACTGGAAGCT	28667	0.13001839859893805	No Hit
TTGCATGATGACTTGAATTGTCGGATACCCCTTCAC	28640	0.12989594083348746	No Hit
GATGGGAGACCGCCTGGGAATACCGGGTGCTGTAGG	27904	0.12655783285676098	No Hit
TGTAAACATCCCCGACTGGAAG	27817	0.12616324672364249	No Hit
TGTAATGATGTTGATCAAATGTCTGACCTGAAATGA	27610	0.12522440385518815	No Hit
ACGGCCCTGGCGGAGCGCTGAGAAGACGGTCGAACT	27127	0.1230337704954614	No Hit
GGTCCAGGATGAAACCTAATTTGAGTGGACATCCAT	26851	0.12178198000418897	No Hit
AGCAAATGATGATAAACTGGATCTGACTGACTGTGC	26772	0.12142367765342621	No Hit
CTGACCTATGAATTGACAGCCAT	26358	0.11954599191651755	No Hit
ATTCAAATCGAACTGCGCCTTC	26145	0.11857993621129644	No Hit
CCTCACTGATGAGTACGTTCTGACTTTCGTTCTTCT	25497	0.11564094984048288	No Hit
TTGCTGTGATGACTATCTTAGGACACCTTTGGATTA	24927	0.11305573034763763	No Hit
TGCTGTGATGAGATGACTAAGTAGGAAGTGCCGTCA	24870	0.11279720839835311	No Hit
CCACAATGATGACAGTTTATTTGCTACTCTTGAGTG	24641	0.11175858512842056	No Hit
TTGCTGTGATGACTATCTTAGGACACCTTTGGAATA	24564	0.11140935372324673	No Hit
GACTCTTAGCGGTGGATCACTCGGCTCGTGCGTCGA	24562	0.1114002827776578	No Hit
AGTCTATGATGATCCTATCCCGAACCTGAATTCCTG	24504	0.1111372253555788	No Hit
ACCCTGTAGATCCGAATTTGTG	24290	0.11016663417756321	No Hit

Sequence	Count	Percentage	Possible Source
TGTCCTGATGATACTTGTAATAGGAAGTGCCGTCAG	23809	0.1079850717634254	No Hit
CGCGACCTCAGATCAGACGC	23801	0.10794878798106966	No Hit
TTTGCATGATGACTTGAATTGTCGGATACCCCTTCA	23793	0.10791250419871394	No Hit
TGTTTGTGATGACTTACATGGAATCTCGTTCGGCTG	23312	0.10573094178457611	No Hit
TTCACAGTGGCTAAGTTCCG	23251	0.10545427794411372	No Hit
GTAAATGATGACTTCACTTTTTTCCCCATC	22993	0.10428412596314167	No Hit
TAACACTGTCTGGTAACGATGT	22468	0.10190300274604736	No Hit
TGGAAGACTAGTGATTTTGTTGTC	22459	0.10186218349089718	No Hit
GCTAATGATGGAAAAATCATTATTGGAAAAGAATGA	22332	0.10128617844600007	No Hit
ATGTTATGATGATGGGCGAAATGTTCAACTGCTCTG	22261	0.10096415987759302	No Hit

Adapter Content

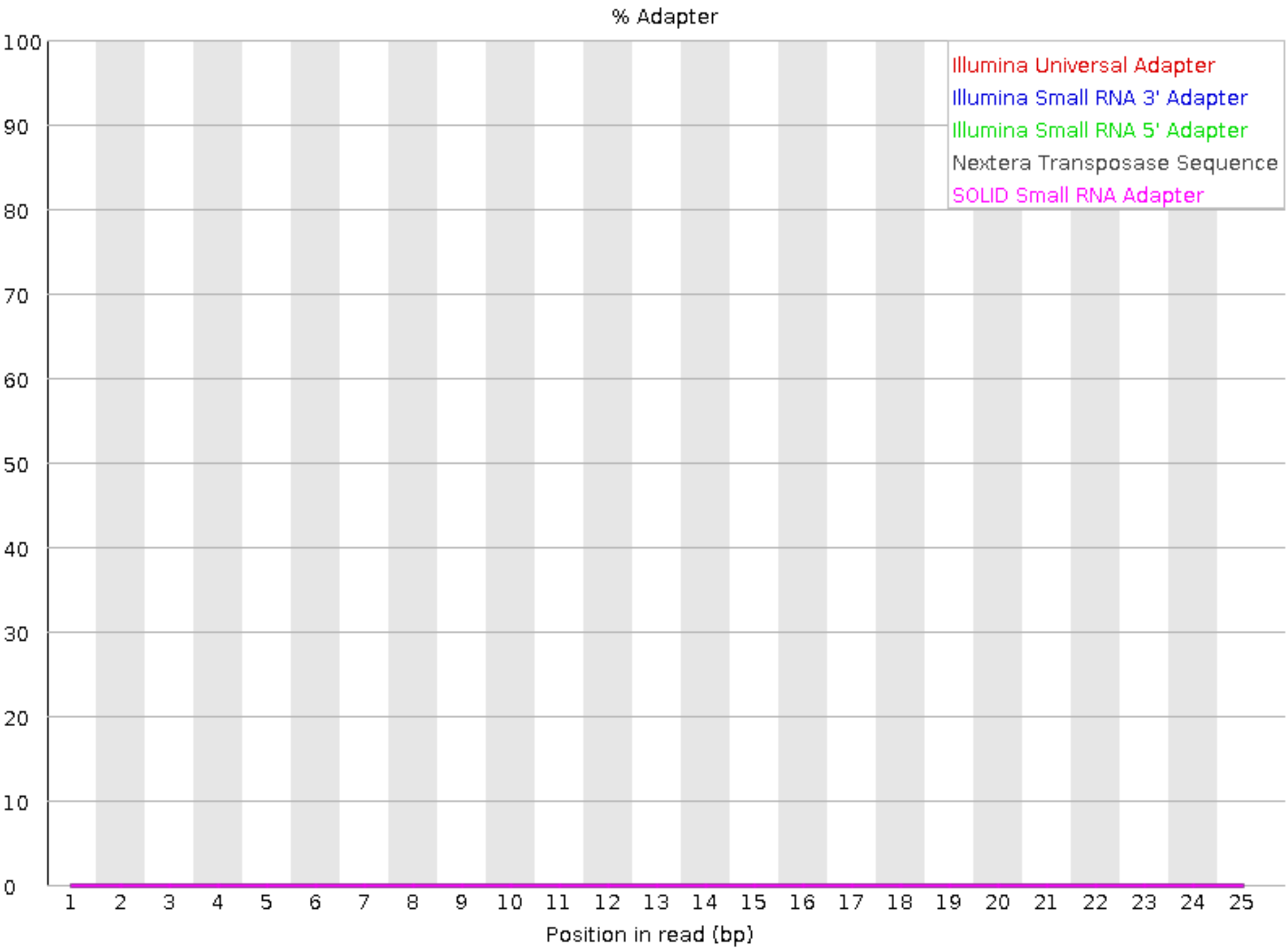

Supplement: Supplementary file 5 [file DataSheet5.zip › QC reports/shCD44_2.fastq.gz FastQC Report.pdf]
