# Supplementary material for: CD44 knockdown alters miRNA expression and their target genes in colon cancer
Source: Front Immunol. 2025 May 14;16:1552665. doi: 10.3389/fimmu.2025.1552665 (PMC12116639; doi:10.3389/fimmu.2025.1552665)

FastQC Report

Mon 31 Mar 2025
shLUC_6.fastq.gz

Summary

- Basic Statistics
- Per base sequence quality
- Per tile sequence quality
- Per sequence quality scores
- Per base sequence content
- Per sequence GC content
- Per base N content
- Sequence Length Distribution
- Sequence Duplication Levels
- Overrepresented sequences
- Adapter Content

Basic Statistics

Measure	Value
Filename	shLUC_6.fastq.gz
File type	Conventional base calls
Encoding	Sanger / Illumina 1.9
Total Sequences	16780608
Sequences flagged as poor quality	0
Sequence length	18-36
%GC	49

Per base sequence quality

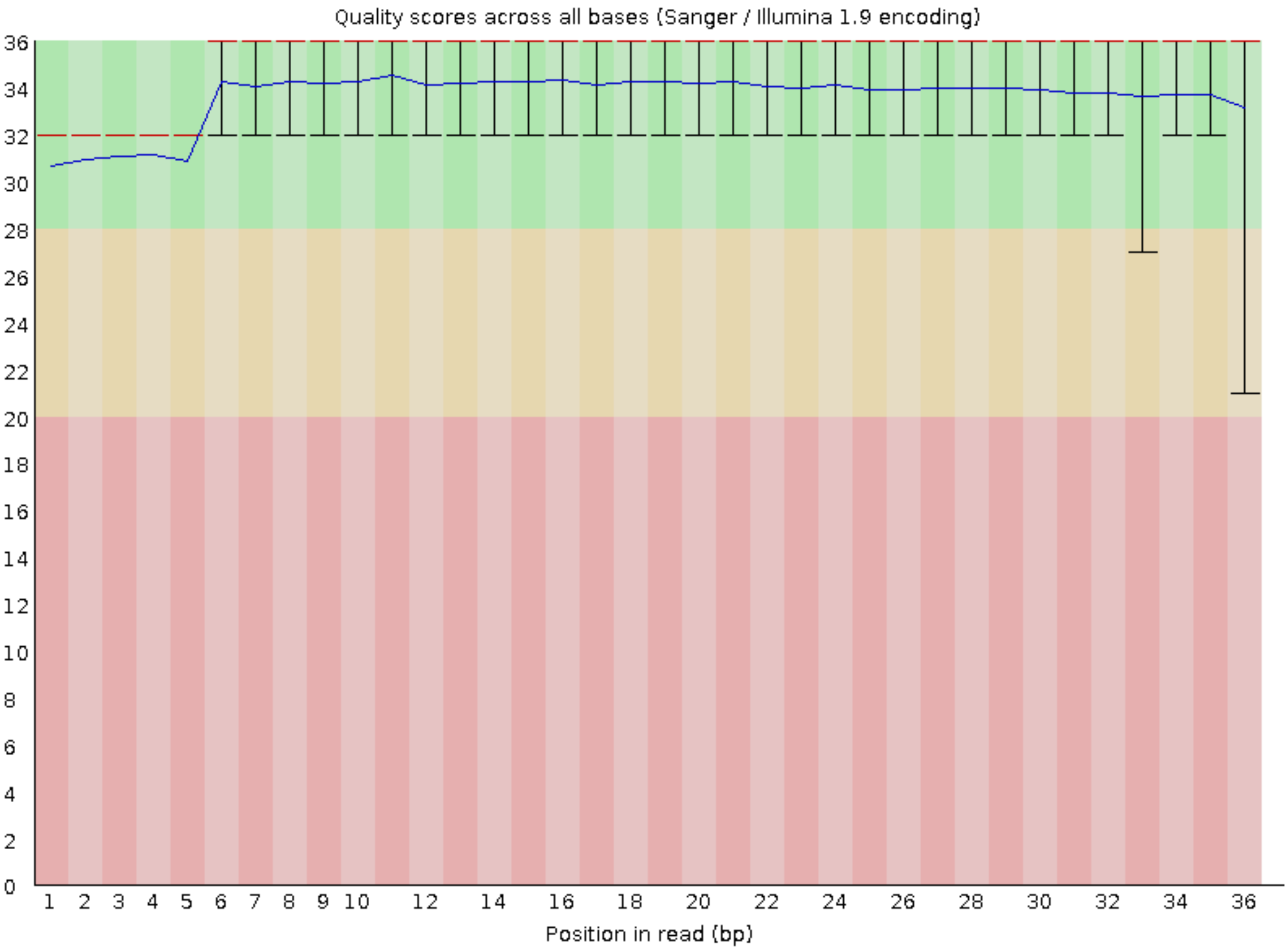

Per tile sequence quality

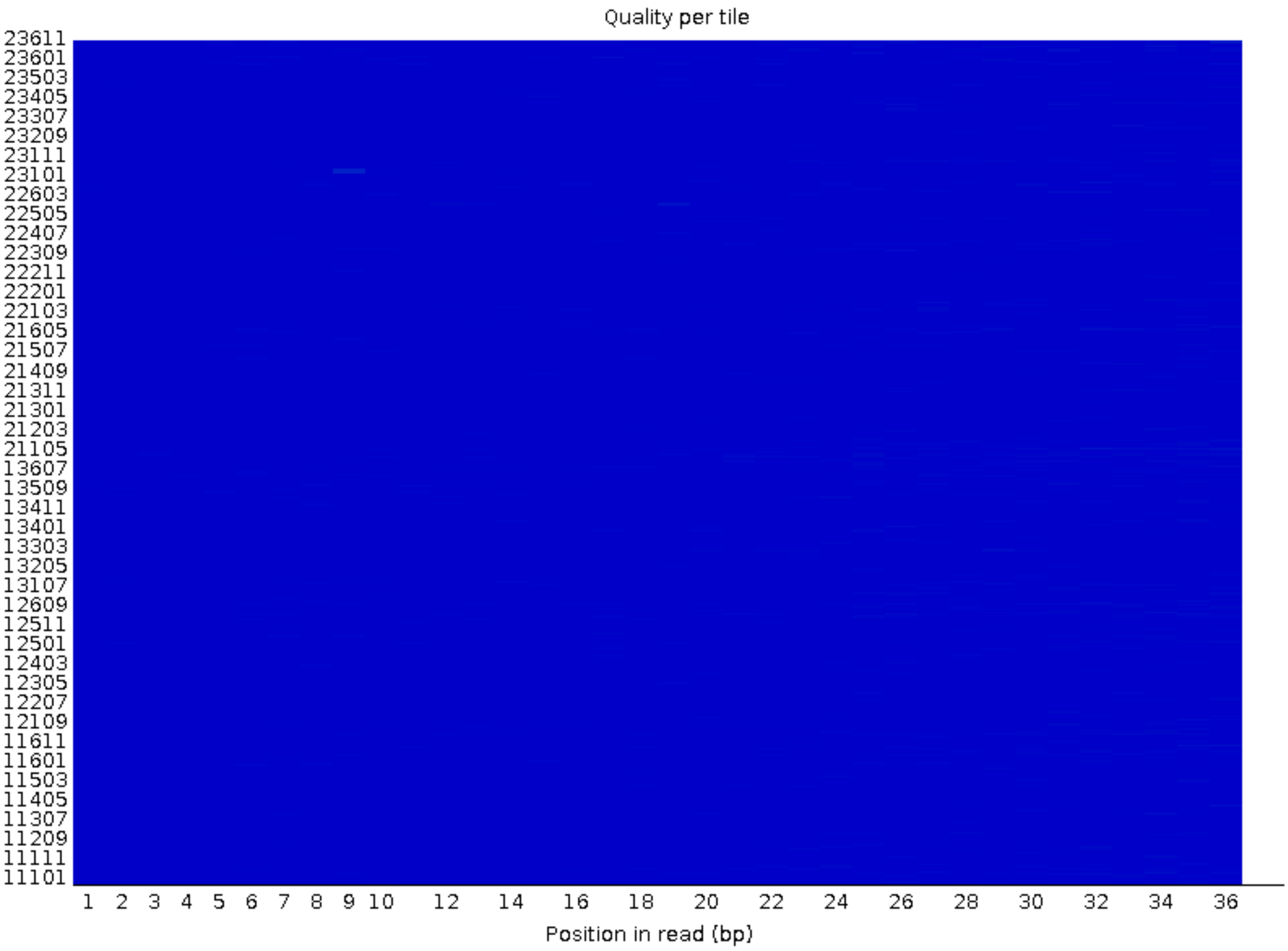

Per sequence quality scores

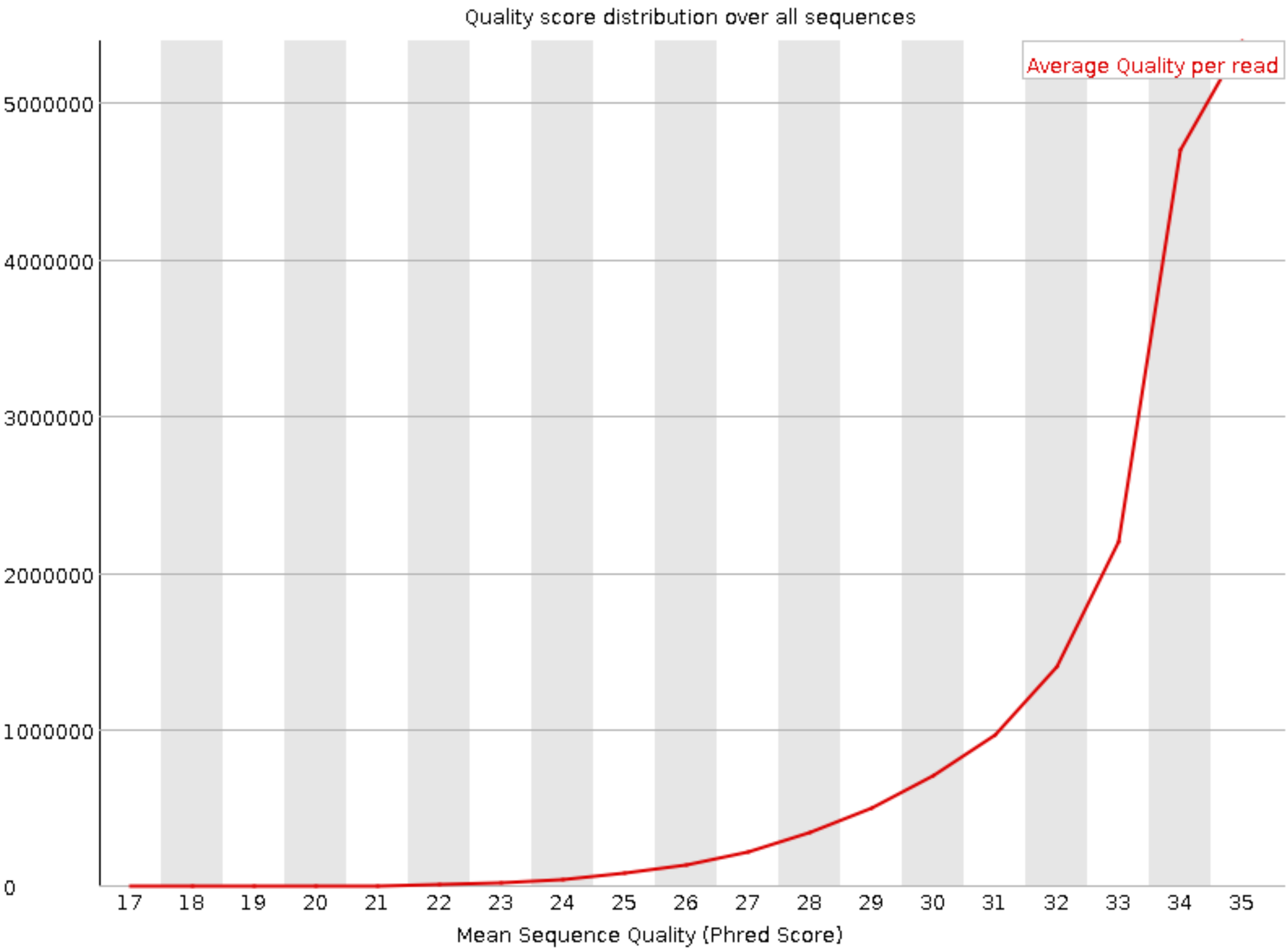

Per base sequence content

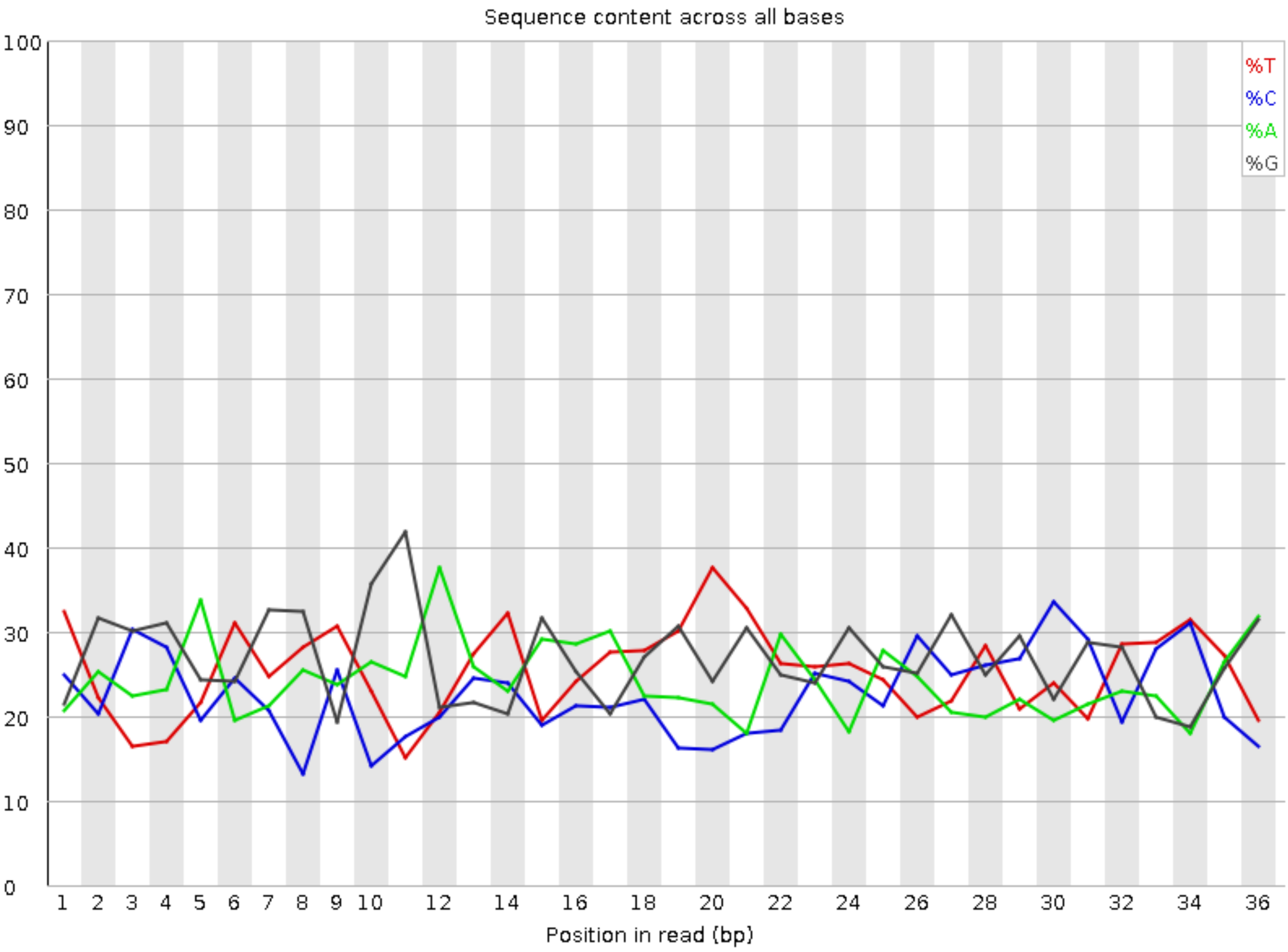

Per sequence GC content

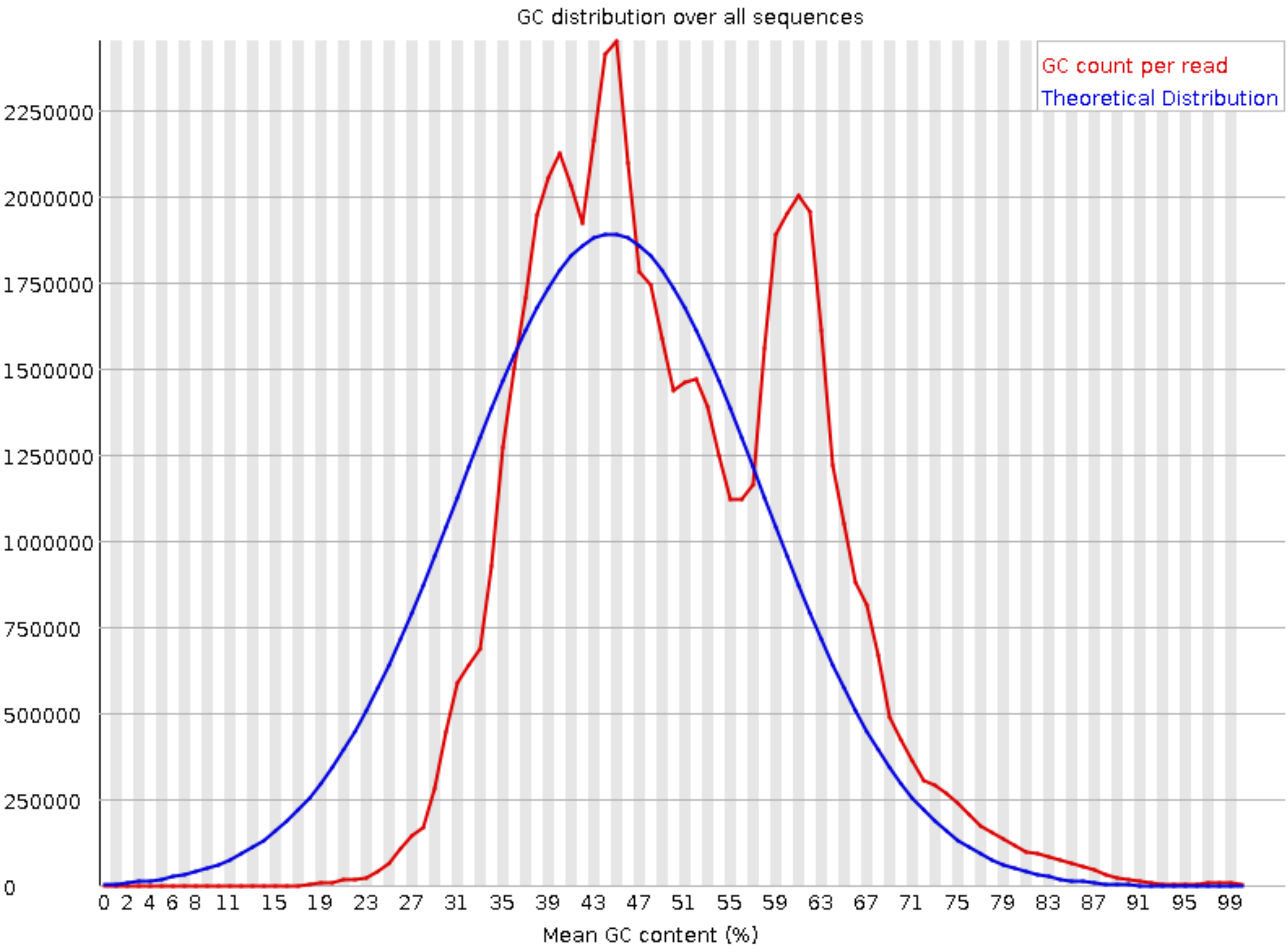

Per base N content

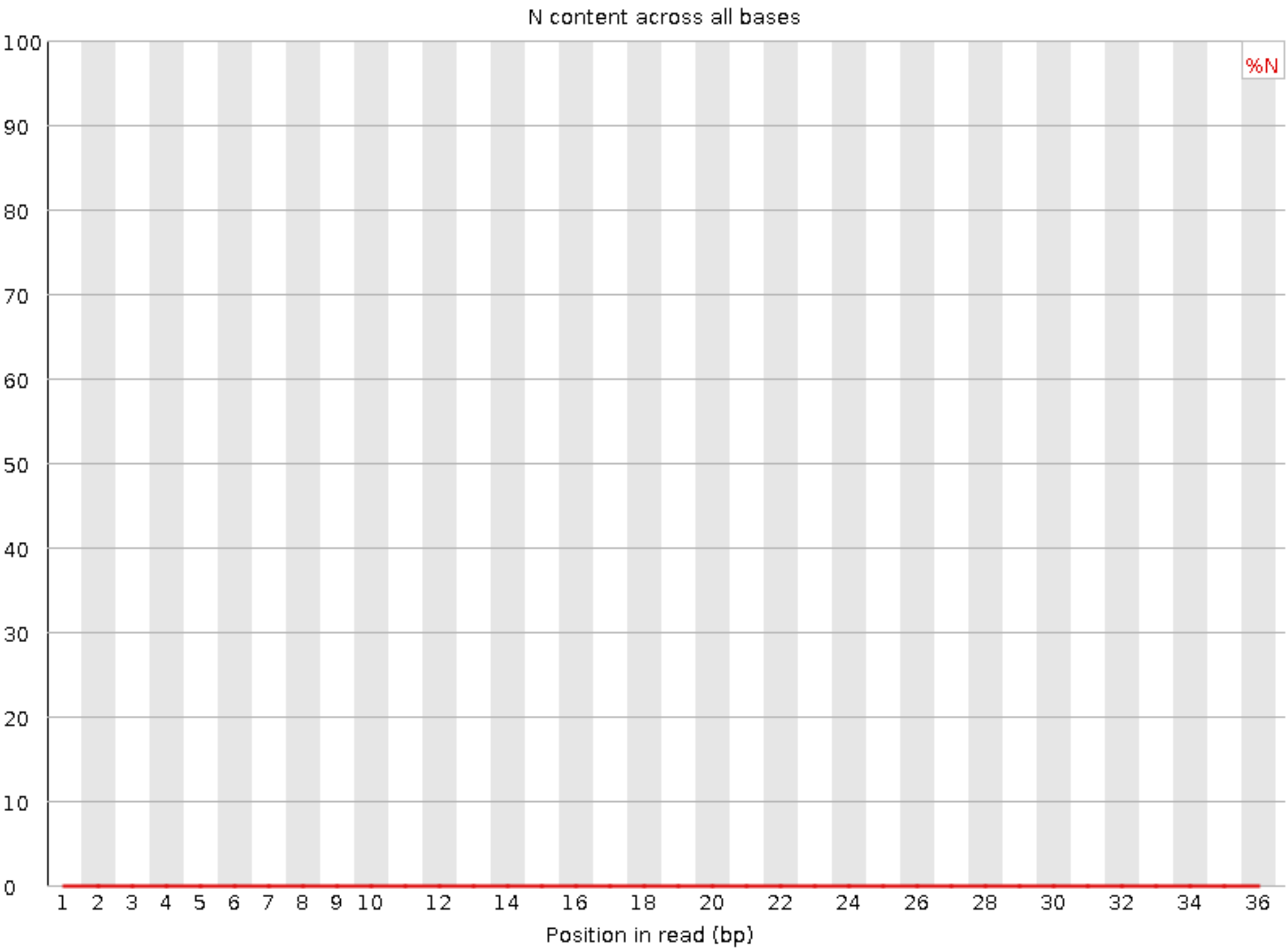

Sequence Length Distribution

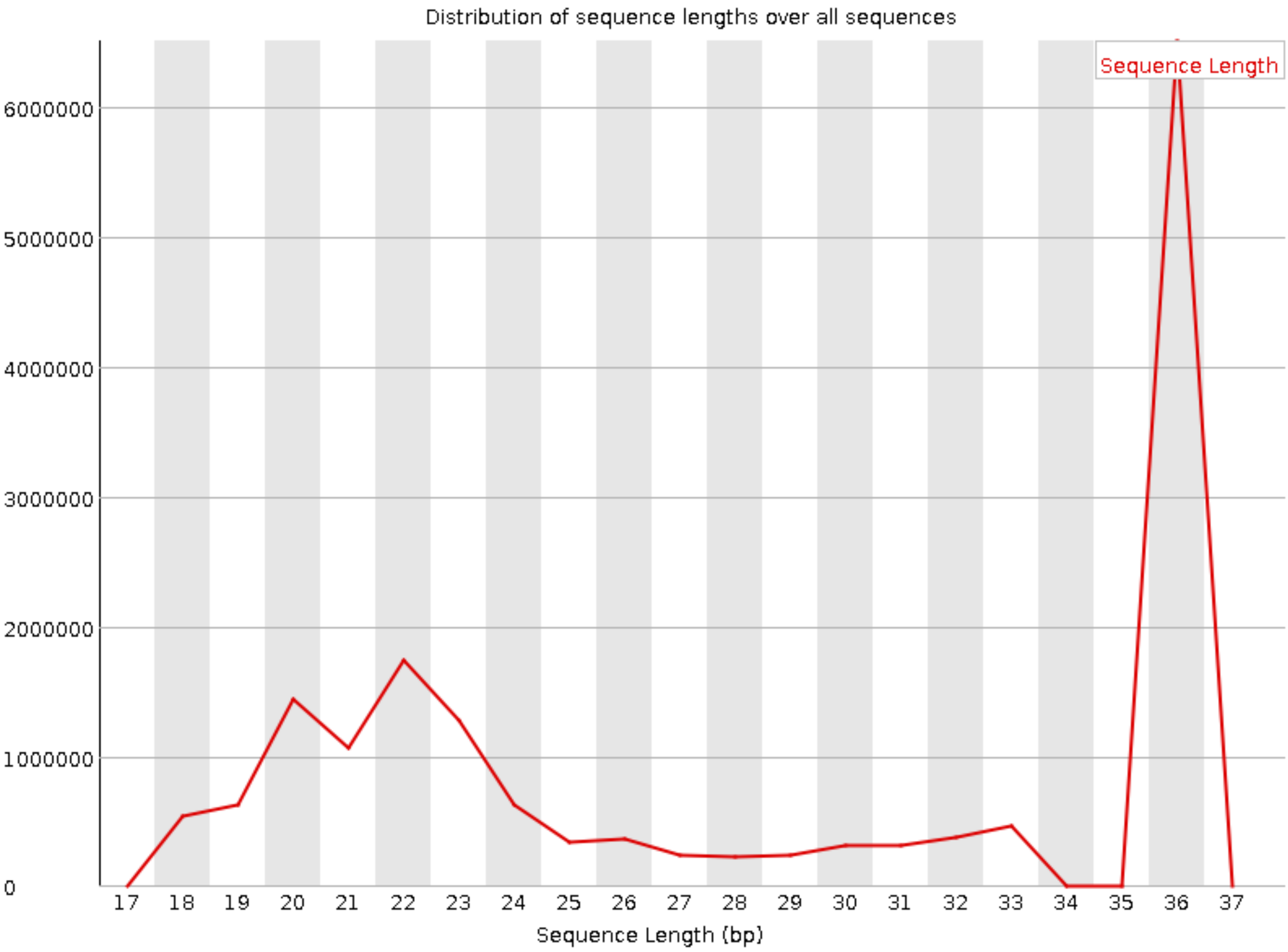

Sequence Duplication Levels

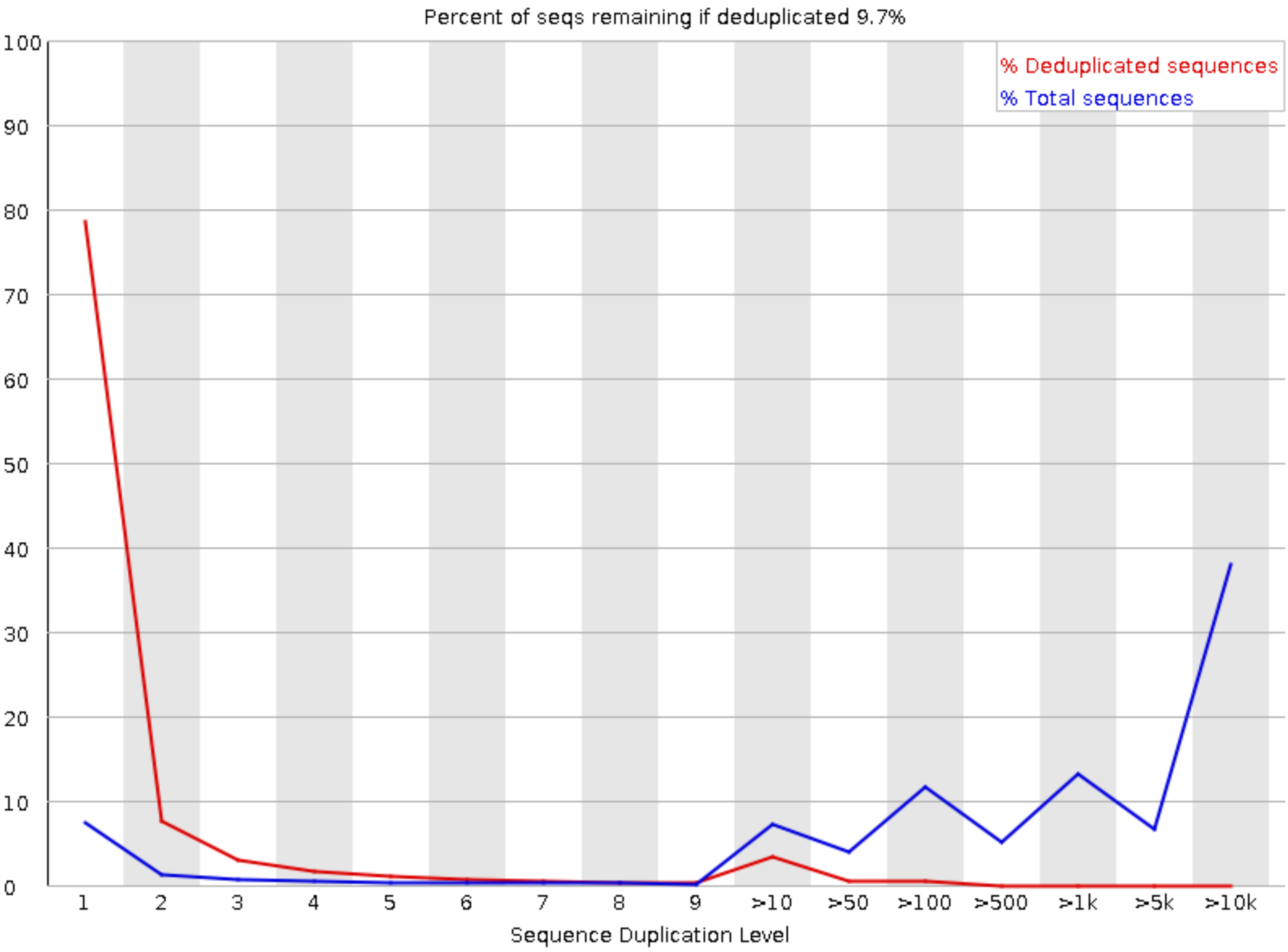

Overrepresented sequences

Sequence	Count	Percentage	Possible Source
CGCGACCTCAGATCAGACGT	633303	3.774017008203755	No Hit
TGCTCTGATGAAATCACTAATAGGAAGTGCCGTCAG	292619	1.7437925967879113	No Hit
TAGCTTATCAGACTGATGTTGAC	275610	1.6424315495600634	No Hit
TAGCTTATCAGACTGATGTTGA	219569	1.3084686800382919	No Hit
GTGAAATGATGGCAATCATCTTTCGGGACTGACCTG	165488	0.9861859594121977	No Hit
GTTTGTGATGACTTACATGGAATCTCGTTCGGCTGA	155734	0.9280593408772794	No Hit
GCCTCTGATGAAGCCTGTGTTGGTAGGGACATCTGA	125444	0.7475533663619339	No Hit
TTGAATGATGACTTTAATTGTCGGATACCCCTTCAC	113945	0.679027839754078	No Hit
AGTAGTGATGAAATTCCACTTCATTGGTCCGTGTTT	105641	0.6295421476981048	No Hit
GTGCAATGATGTATTTTATTCAACACATCATTCTGA	101828	0.6068194906882992	No Hit
CGCGACCTCAGATCAGACGC	95750	0.5705991105924172	No Hit
TTGGTACTAGCAACGCACTTT	90456	0.5390507900548062	No Hit

Sequence	Count	Percentage	Possible Source
GAGAAGACGGTCGAACTTGACTATCT	88733	0.528782985693963	No Hit
TATCTGTGATGATCTTATCCCGAACCTGAACTTCTG	84126	0.5013286765294798	No Hit
CGCGACCTCAGATCAGACGTGGCGACCCGCTGAATT	81197	0.4838740050420104	No Hit
CCTGGATGATGATAAGCAAATGCTGACTGAACATGA	77825	0.4637793815337323	No Hit
CGACTCTTAGCGGTGGATCACTCGGCTCGTGCGTCG	75445	0.4495963435889808	No Hit
TGGAAGACTAGTGATTTTGTTGTT	74974	0.44678953229823376	No Hit
TCGCTGCGATCTATTGAAAGTCAGCCCTCGACACAA	74505	0.4439946395267681	No Hit
TAGCTTATCAGACTGATGTTGAT	73742	0.43944772442095065	No Hit
TTTCTATGATGAATCAAACTAGCTCACTATGACCGA	72260	0.43061610163350456	No Hit
ATACATGATGATCTCAATCCAACTTGAACTCTCTCA	67855	0.4043655629164331	No Hit
ACCGGGTGCTGTAGGCTT	63855	0.38052852435382556	No Hit
TGAGGTAGTAGATTGTATAGTT	62859	0.3745931017517363	No Hit
ACCGGGTGCTGTAGGCTTT	60816	0.36241833430588455	No Hit
CTACGGGGATGATTTTACGAACTGAACTCTCTCTTT	57262	0.3412391255430078	No Hit
TGAGGTAGTAGTTTGTGCTGTT	57083	0.34017241806733106	No Hit
CGCTGCGATCTATTGAAAGTCAGCCCTCGACACAAG	53246	0.3173067388261498	No Hit
TGAAATGATGGCAATCATCTTTCGGGACTGACCTGA	52983	0.31573945354065835	No Hit
AGAAGACGGTCGAACTTGACTATCT	51171	0.3049412750717972	No Hit
CGCGACCTCAGATCAGACG	48674	0.29006100374908944	No Hit
ACAAATGATGAATAACAAAGGGACTTAATACTG	47622	0.28379186260712363	No Hit
ACTCCATGATGAACACAAAATGACAAGCATATGGCT	47522	0.2831959366430584	No Hit
CAGGACGGTGGCCATGGAAGTCGGAATCCGCTAAGG	47250	0.28157501802080115	No Hit
GATGGGAGACCGCCTGGGAATACCGGGTGCTGTAGG	45539	0.2713787247756458	No Hit
TAACACTGTCTGGTAACGATGTT	44926	0.2677256986159262	No Hit
TAGCTTATCAGACTGATGTTG	44586	0.26569955033810455	No Hit
TTTGAATGATGACTTTAATTGTCGGATACCCCTTCA	44293	0.2639534872633935	No Hit
CTCGCTGCGATCTATTGAAAGTCAGCCCTCGACACA	42951	0.2559561608256387	No Hit
TAGCTTATCAGACTGATGTTGACT	42836	0.25527084596696376	No Hit
TAGCTTATCAGACTGATGTTGACA	42786	0.2549728829849312	No Hit
TCAGTGCACTACAGAACTTTGT	42036	0.25050343825444227	No Hit
GTGAAATGATGGCAAATCATCTTTCGGGACTGACCT	42020	0.2504080901001919	No Hit
TTCAAGTAATCCAGGATAGGCT	40887	0.24365624892733326	No Hit
GCATTGGTGGTTCAGTGGTAGAATTCTCGCCT	40701	0.242547826634172	No Hit
TGCCTCTGATGAAGCCTGTGTTGGTAGGGACATCTG	39318	0.23430617055115047	No Hit
TACCCTGTAGATCCGAATTTGT	38426	0.22899051095168899	No Hit
GCAAATGATGATAAACTGGATCTGACTGACTGTGCT	37452	0.22318619206169407	No Hit
GACGTGGCGACCCGCTGAATTT	33862	0.20179244995175383	No Hit

Sequence	Count	Percentage	Possible Source
TTGGTACTAGCAACGCACTTTT	33467	0.19943854239369632	No Hit
CGCGACCTCAGATCAGACGA	32981	0.19654234220833952	No Hit
TGAAATGATGGCAAATCATCTTTCGGGACTGACCTG	32105	0.19132203076312848	No Hit
CTGCAGTGATGACTTTCTTAGGACACCTTTGGATTT	32079	0.19116709001247154	No Hit
TGGGAGACCGCCTGGGAATACCGGGTGCTGTAGGCT	30996	0.18471321182164555	No Hit
CTGAATGATGATATCCCACTAACTGAGCAGTCAGTA	30611	0.1824188968599946	No Hit
TGAGGTAGTAGTTTGTACAGTT	29743	0.17724625949190875	No Hit
CTCCTACTTGGATAACTGTGGTAATTCTAGAGCTAA	29677	0.17685294835562573	No Hit
CTGGATGATGATAAGCAAATGCTGACTGAACATGAA	29527	0.17595905940952797	No Hit
AGACGTGGCGACCCGCTGAATTT	29428	0.1753690927051034	No Hit
CTCACTGATGAGTACGTTCTGACTTTCGTTCTTCTG	29293	0.1745645926536154	No Hit
CTAGACTGAAGCTCCTTGAGG	28878	0.1720914999027449	No Hit
AATGGATTTTTGGAGCAGG	27728	0.16523835131599524	No Hit
ATATATGATGACTTAGCTTTTTTCCCCGAC	27151	0.16179985850333908	No Hit
CTGACCTATGAATTGACAGCC	27004	0.16092384733616327	No Hit
CGCGACCTCAGATCAGACGG	26834	0.15991077319725244	No Hit
AACTGTGATGAAAGATTTGGTCTGTATGTAAT	26436	0.157538987860273	No Hit
CACAGATGATGAACTTATTGACGGGCGGACAGAAAC	26315	0.15681791744375412	No Hit
TGAGGTAGTAGGTTGTATAGTT	25836	0.1539634320758819	No Hit
TGGAAGACTAGTGATTTTGTTGT	25827	0.153909798739116	No Hit
GACGTGGCGACCCGCTGAATT	25066	0.14937480215257995	No Hit
TAATACTGCCTGGTAATGATGAC	24649	0.1468897908824281	No Hit
GCAGCTGATGATACAGCTTCTTTCCCCATC	24539	0.14623427232195638	No Hit
TGTAACAGCAACTCCATGTGGA	24178	0.14408297959168107	No Hit
TACAATGATGATAACATAGTTCAGCAGACTAACGCT	23615	0.14072791641399407	No Hit
ACGGCCCTGGCGGAGCGCTGAGAAGACGGTCGAACT	23210	0.13831441625953006	No Hit
TAATACTGTCTGGTAAAACCGT	22740	0.13551356422842364	No Hit
TAGGGTGATGAAAAAGAATCCTTAGGCGTGGTTGTG	22500	0.1340833419146672	No Hit
TAATACTGCCTGGTAATGATGA	21916	0.13060313428452652	No Hit
TCTCCTACTTGGATAACTGTGGTAATTCTAGAGCTA	21553	0.12843992303496987	No Hit
GTTGAGGTCTATCCCGATGGGGCTTTTCCTGTAGCC	20865	0.1243399524022014	No Hit
CTGACCTATGAATTGACAGCT	20847	0.12423268572866966	No Hit
ATACATGATGATCTCACACAACTTGAACTCTCTCAC	20292	0.12092529662810786	No Hit
TTCACAGTGGCTAAGTTCTGC	20138	0.12000757064344748	No Hit
TAATACTGCCGGGTAATGATGGA	19928	0.1187561261189106	No Hit
GCATTGGTGGTTCAGTGGTAGAATTCTCGCC	19263	0.11479321845787709	No Hit
AGACGTGGCGACCCGCTGAATT	19124	0.11396488136782648	No Hit

Sequence	Count	Percentage	Possible Source
TTCCTATGATGAGGACCTTTTCACAGACCTGTACTG	19121	0.11394700358890453	No Hit
TTCACAGTGGCTAAGTTCCG	18988	0.11315442205669783	No Hit
GCAGCCGACTTAGAACTGGTGCGGACCAGGGGAATC	18558	0.11059194041121753	No Hit
TAACACTGTCTGGTAACGATGT	18088	0.10779108838011113	No Hit
CTTAATGATGACTGTTTTTTTTGATTGCTTGAAGCA	18039	0.1074990846577192	No Hit
TACCCTGTAGATCCGAATTTGTG	17971	0.10709385500215488	No Hit
TTGCATGATGACTTGAATTGTCGGATACCCCTTCAC	17908	0.1067184216447938	No Hit
GACTCTTAGCGGTGGATCACTCGGCTCGTGCGTCGA	17780	0.10595563641079037	No Hit
AAGCTATGATGAATTTGATTGCATTGATCGTCTGAC	17721	0.10560404009199191	No Hit
TAATACTGCCTGGTAATGATG	17063	0.10168284724844297	No Hit
GCTTAATGATGACTGTTTTTTTTGATTGCTTGAAGC	16851	0.10041948420462478	No Hit

Adapter Content

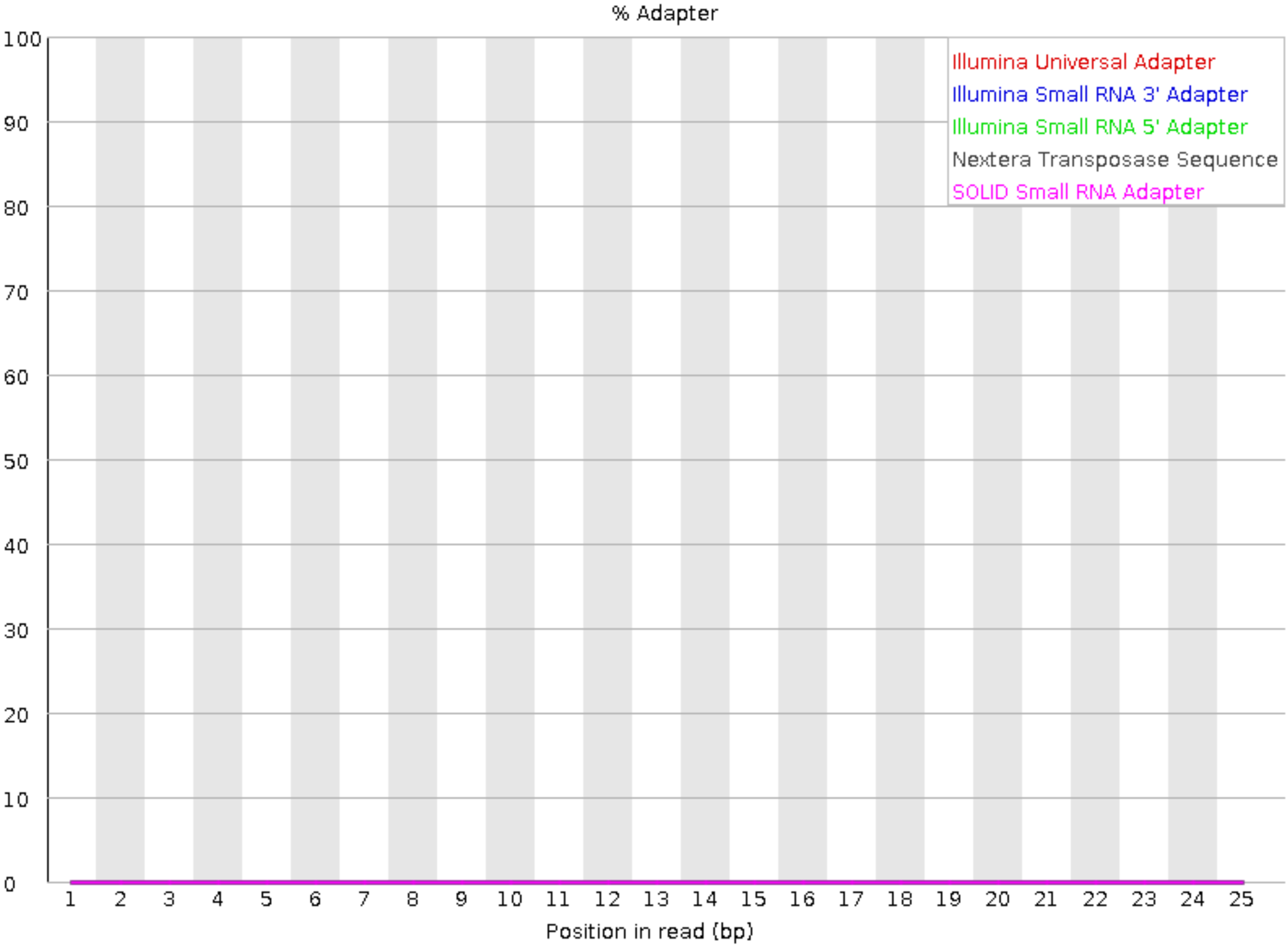

Supplement: Supplementary file 5 [file DataSheet5.zip › QC reports/shLUC_6.fastq.gz FastQC Report.pdf]
