# Supplementary material for: CD44 knockdown alters miRNA expression and their target genes in colon cancer
Source: Front Immunol. 2025 May 14;16:1552665. doi: 10.3389/fimmu.2025.1552665 (PMC12116639; doi:10.3389/fimmu.2025.1552665)
Supplement: Supplementary file 5 [file DataSheet5.zip › QC reports/shCD44_3.fastq.gz FastQC Report.pdf]

FastQC Report

Mon 31 Mar 2025
shCD44_3.fastq.gz

Summary

- Basic Statistics
- Per base sequence quality
- Per tile sequence quality
- Per sequence quality scores
- Per base sequence content
- Per sequence GC content
- Per base N content
- Sequence Length Distribution
- Sequence Duplication Levels
- Overrepresented sequences
- Adapter Content

Basic Statistics

Measure	Value
Filename	shCD44_3.fastq.gz
File type	Conventional base calls
Encoding	Sanger / Illumina 1.9
Total Sequences	21570447
Sequences flagged as poor quality	0
Sequence length	18-36
%GC	46

Per base sequence quality

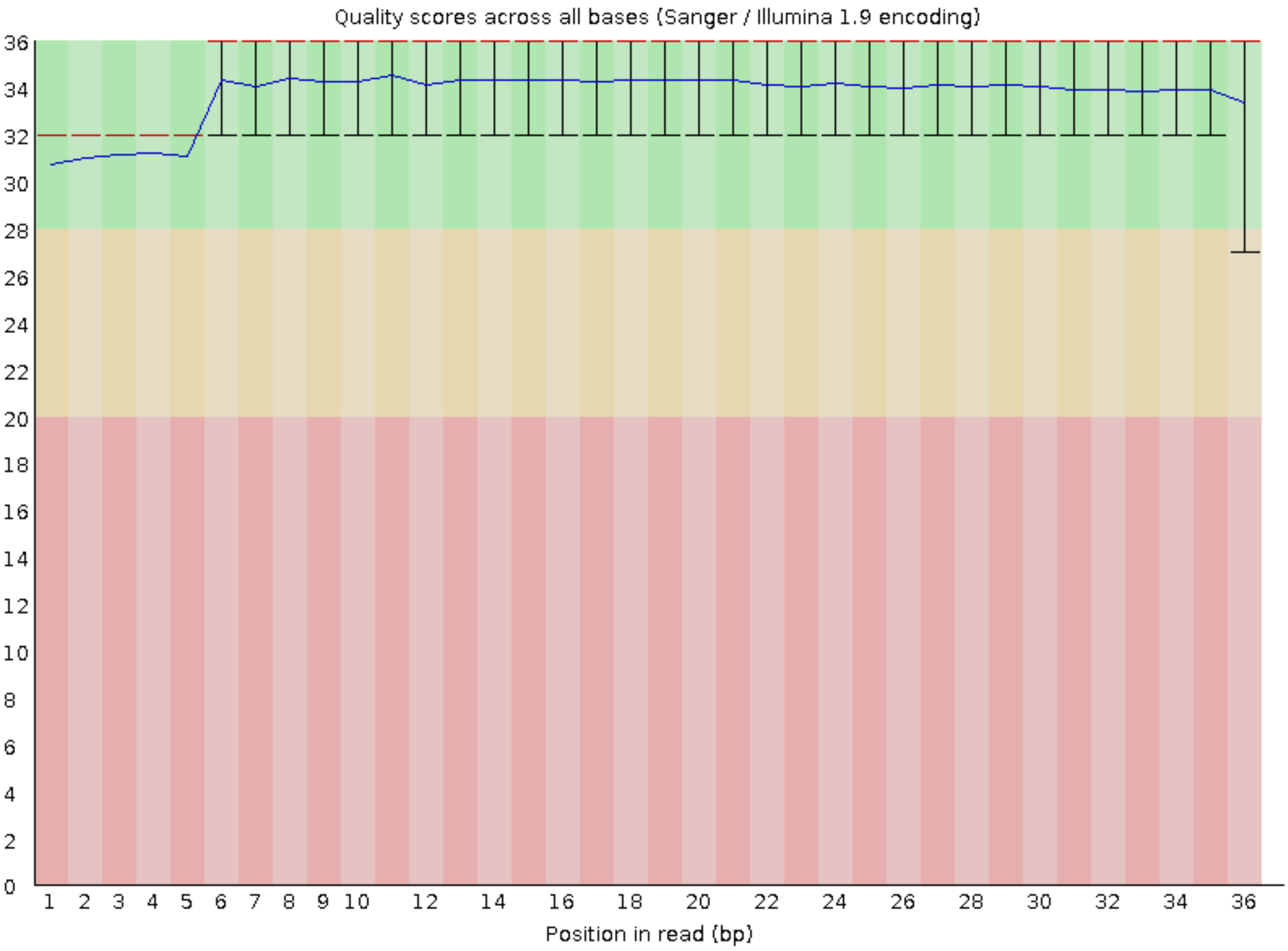

Per tile sequence quality

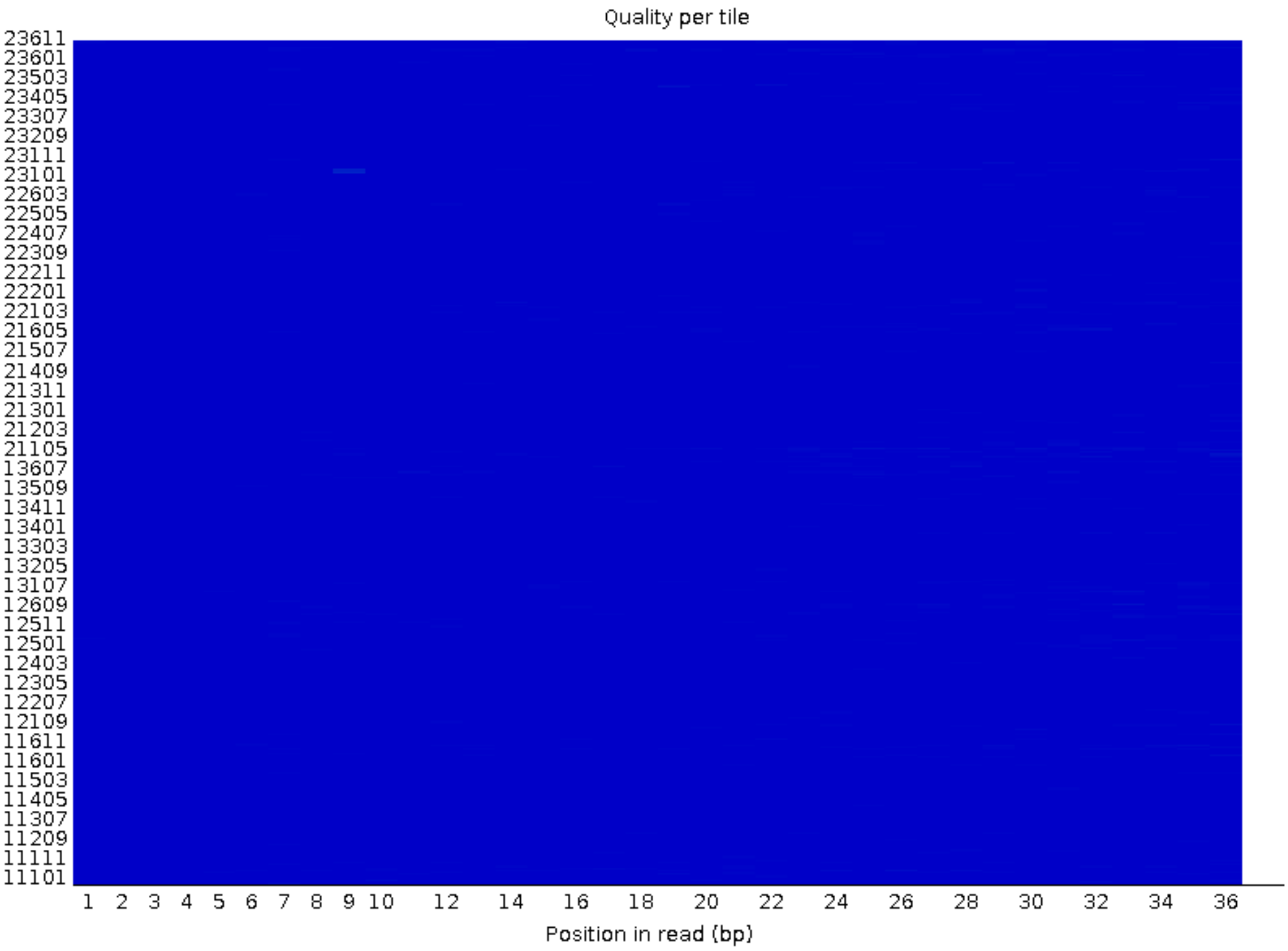

Per sequence quality scores

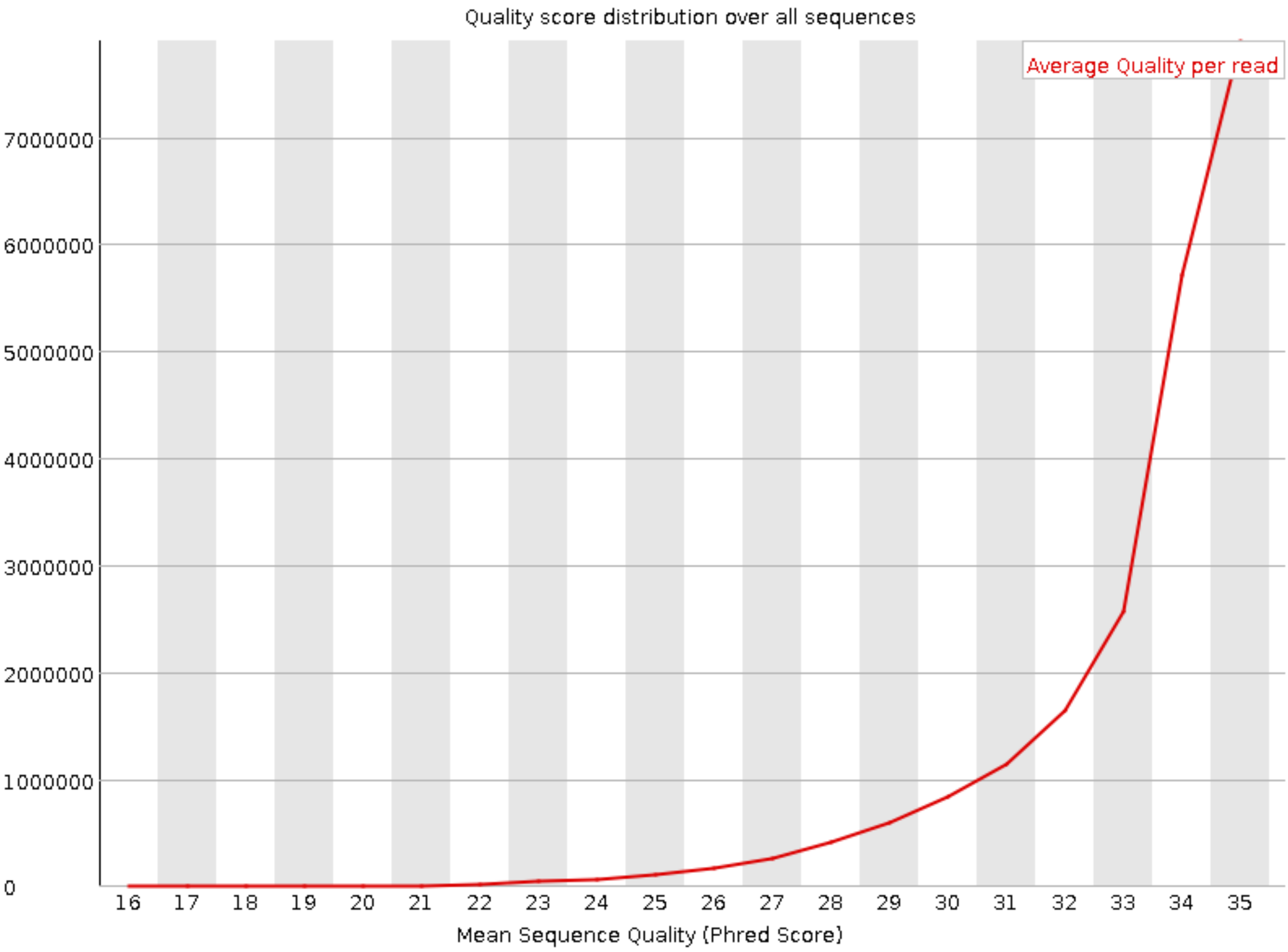

Per base sequence content

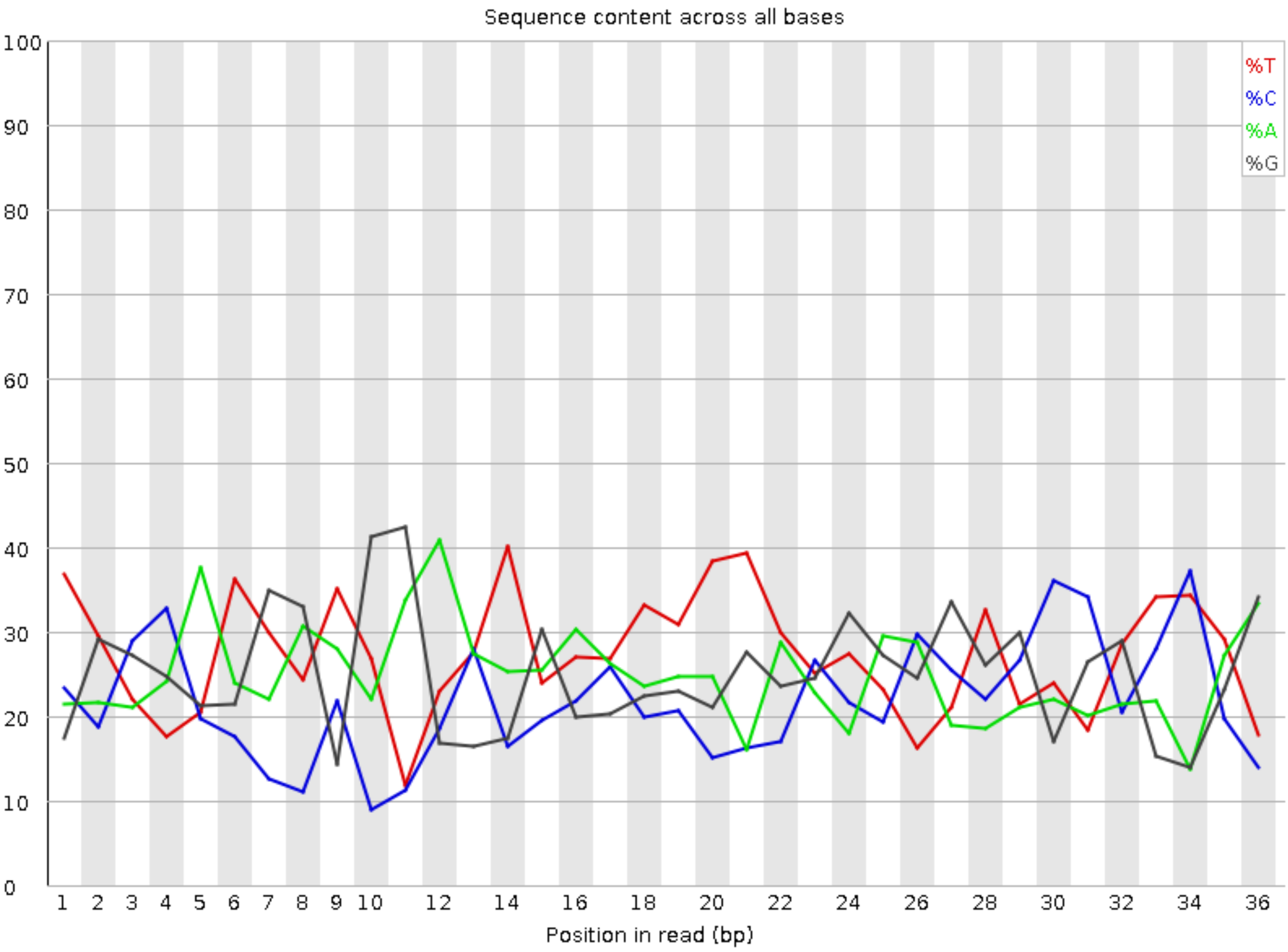

Per sequence GC content

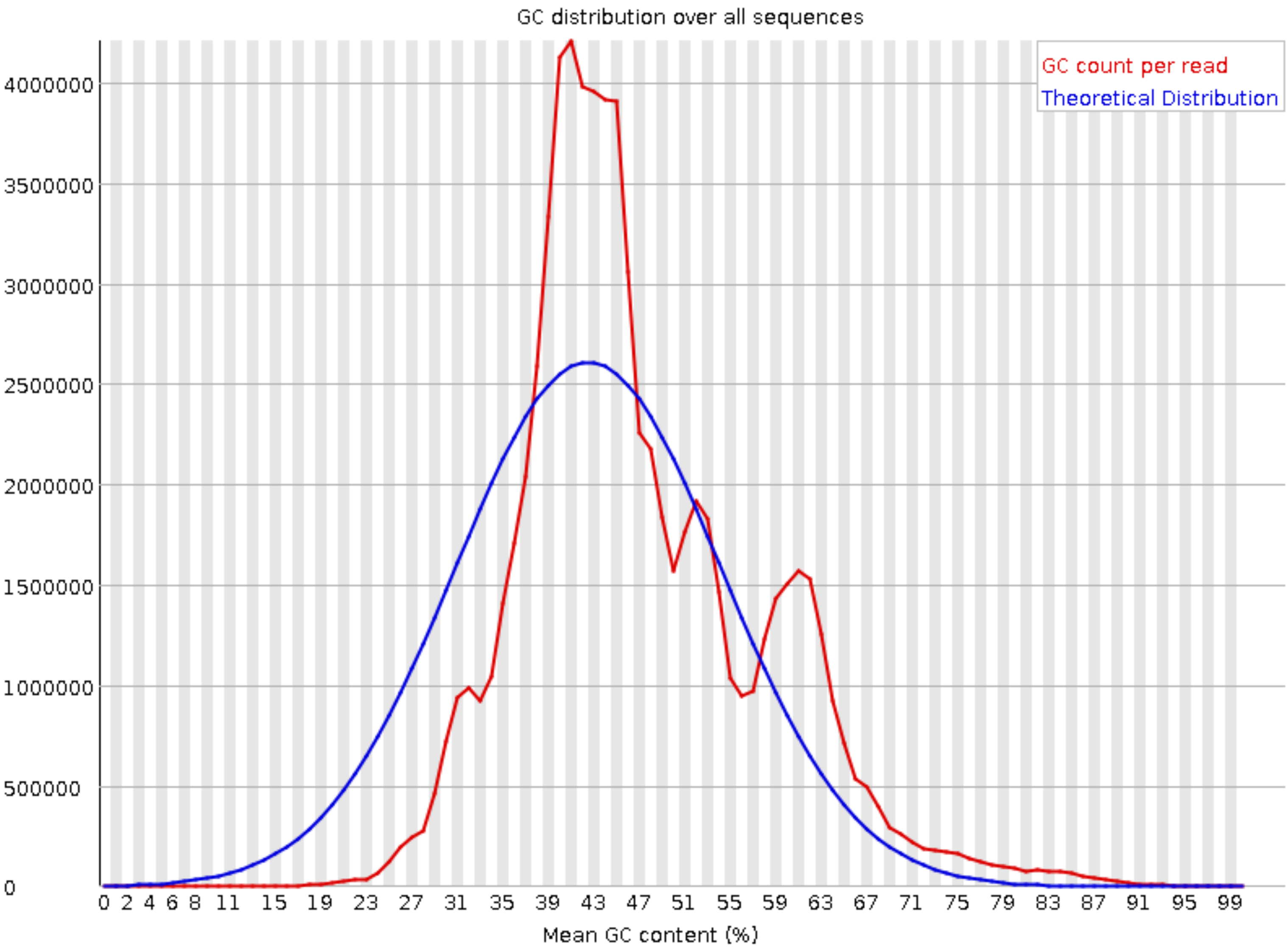

Per base N content

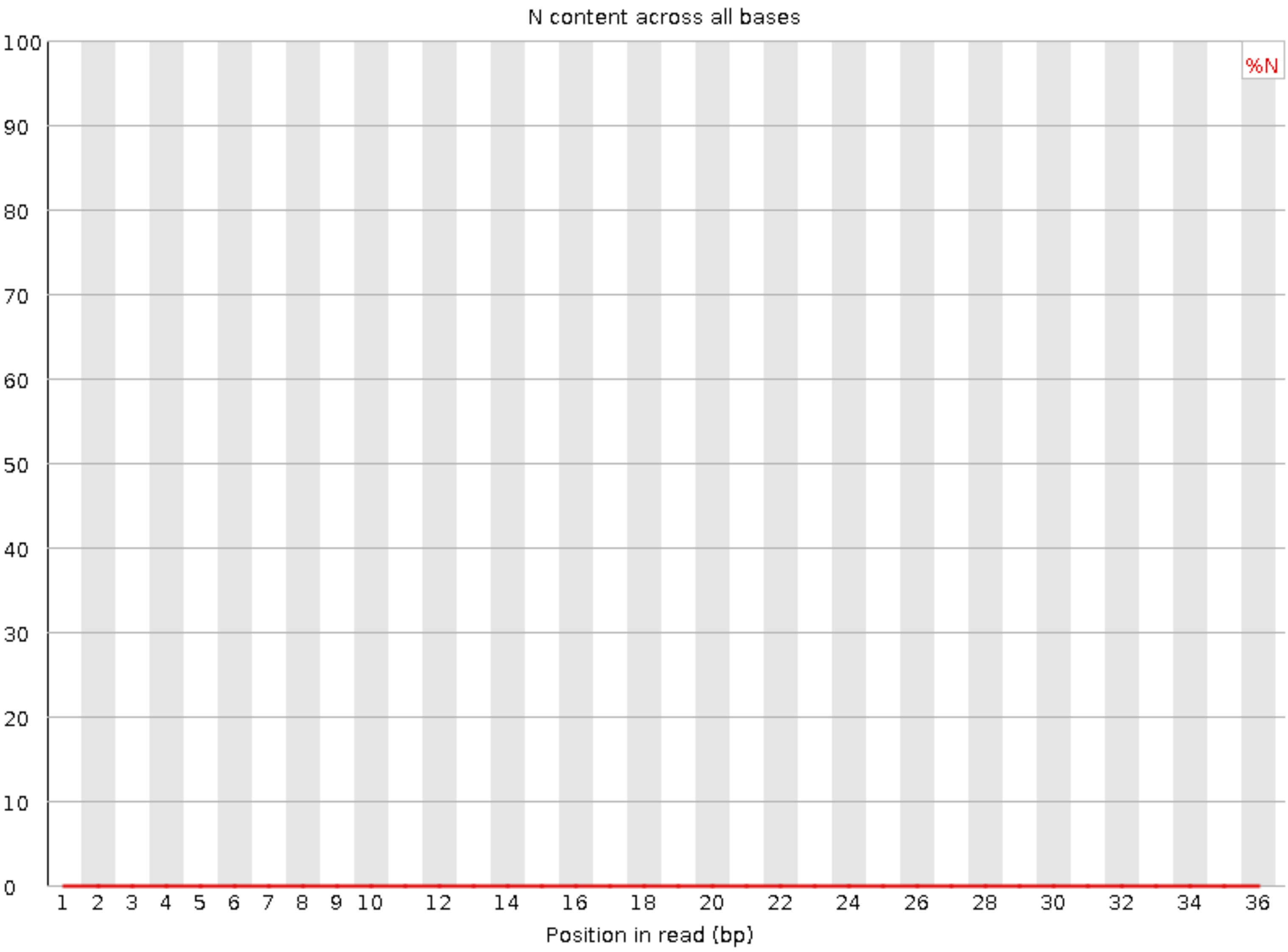

Sequence Length Distribution

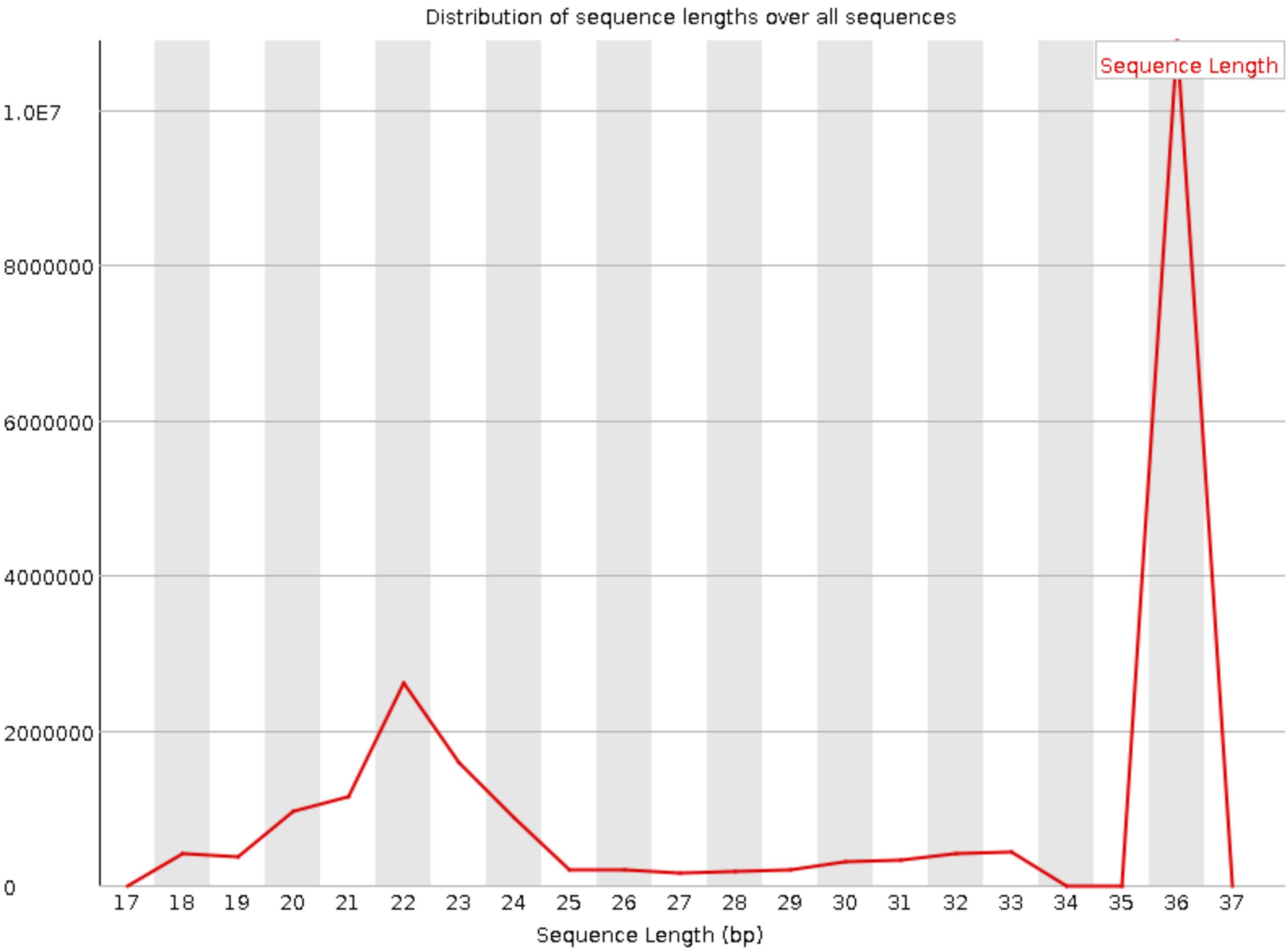

Sequence Duplication Levels

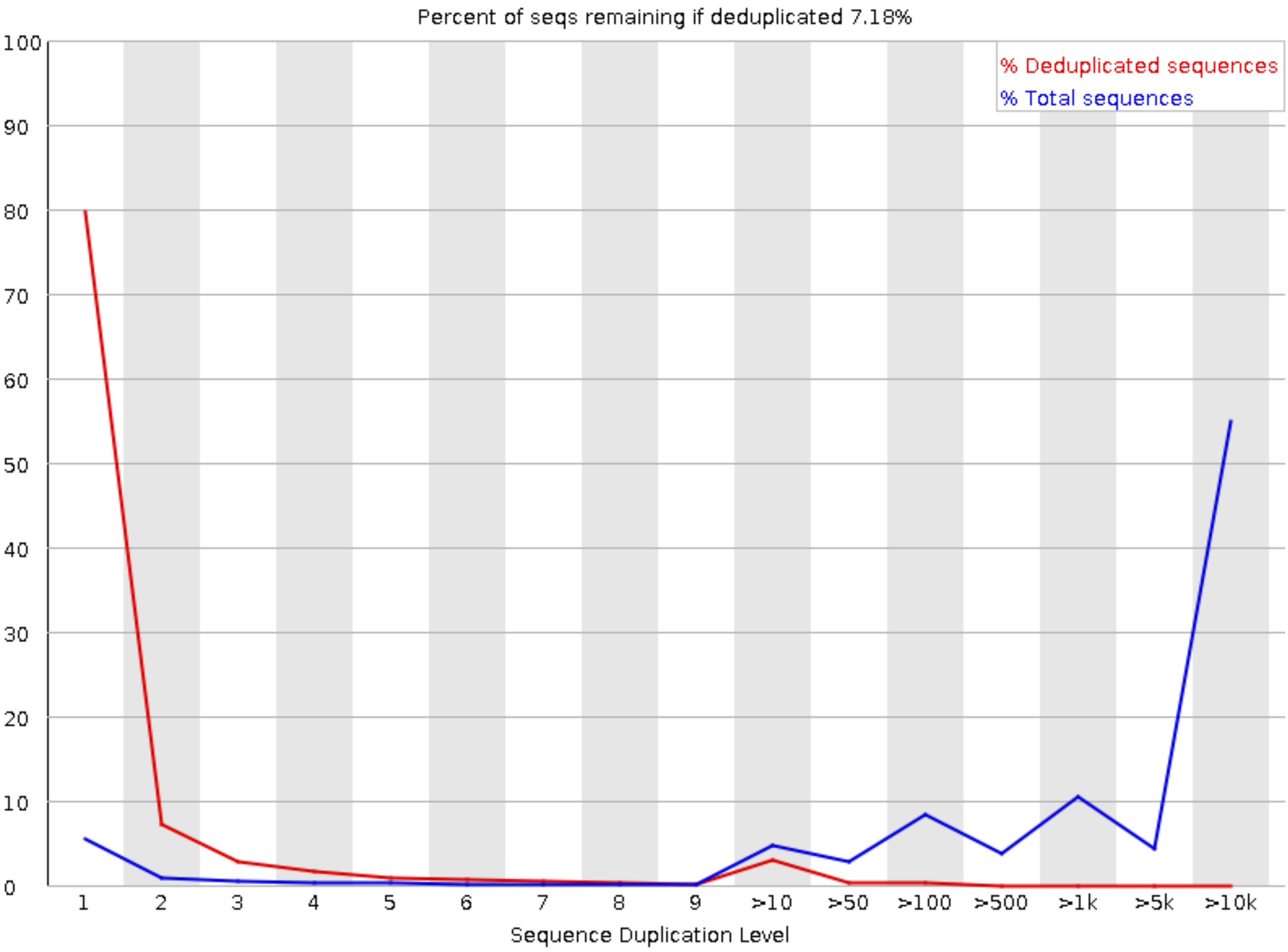

Overrepresented sequences

Sequence	Count	Percentage	Possible Source
TGCTCTGATGAAATCACTAATAGGAAGTGCCGTCAG	895942	4.153562510781534	No Hit
TAGCTTATCAGACTGATGTTGAC	543575	2.5199987742488603	No Hit
ATTCAAATCGATCTGCGCCTTT	437537	2.0284095178926984	No Hit
CGCGACCTCAGATCAGACGT	388801	1.8024707601098855	No Hit
CCTGGATGATGATAAGCAAATGCTGACTGAACATGA	385578	1.7875290206086132	No Hit
GTGAAATGATGGCAATCATCTTTCGGGACTGACCTG	354751	1.6446158950716228	No Hit
TATCTGTGATGATCTTATCCCGAACCTGAACTTCTG	324621	1.5049340423960615	No Hit
AGTAGTGATGAAATTCCACTTCATTGGTCCGTGTTT	264643	1.226877681301644	No Hit
TGGAAGACTAGTGATTTTGTTGTT	205963	0.9548388125661003	No Hit
GTGCAATGATGTATTTTATTCAACACATCATTCTGA	197860	0.917273527062281	No Hit
CTCGCTGCGATCTATTGAAAGTCAGCCCTCGACACA	186782	0.8659162232474831	No Hit
GCCTCTGATGAAGCCTGTGTTGGTAGGGACATCTGA	175795	0.814980792933962	No Hit

Sequence	Count	Percentage	Possible Source
ATTCAAATCGAACTGCGCCTTT	169022	0.7835813509103451	No Hit
GTTTGTGATGACTTACATGGAATCTCGTTCGGCTGA	167542	0.776720111548917	No Hit
TTGAATGATGACTTTAATTGTCGGATACCCCTTCAC	157936	0.7321869593152149	No Hit
CTACGGGGATGATTTTACGAACTGAACTCTCTCTTT	151252	0.7012001188477921	No Hit
TGAAATGATGGCAATCATCTTTCGGGACTGACCTGA	148288	0.6874590962347698	No Hit
TCAGTGCACTACAGAACTTTGT	142413	0.6602227575534249	No Hit
GTGAAATGATGGCAAATCATCTTTCGGGACTGACCT	142054	0.6585584434110243	No Hit
CTAGACTGAAGCTCCTTGAGG	137317	0.6365978414819128	No Hit
ATACATGATGATCTCAATCCAACTTGAACTCTCTCA	136339	0.6320638603363203	No Hit
TAGCTTATCAGACTGATGTTGA	121397	0.5627931586211449	No Hit
TGAAATGATGGCAAATCATCTTTCGGGACTGACCTG	120826	0.5601460182999454	No Hit
TGCCTCTGATGAAGCCTGTGTTGGTAGGGACATCTG	119595	0.5544391361013521	No Hit
GCAAATGATGATAAACTGGATCTGACTGACTGTGCT	111921	0.5188626828178386	No Hit
TCGCTGCGATCTATTGAAAGTCAGCCCTCGACACAA	111037	0.5147644830911479	No Hit
TTTGAATGATGACTTTAATTGTCGGATACCCCTTCA	104355	0.48378691456880796	No Hit
CTGGATGATGATAAGCAAATGCTGACTGAACATGAA	103885	0.4816080074743004	No Hit
TAGCTTATCAGACTGATGTTGACT	103003	0.47751907969269247	No Hit
CGACTCTTAGCGGTGGATCACTCGGCTCGTGCGTCG	100679	0.46674507950623373	No Hit
TTTCTATGATGAATCAAACTAGCTCACTATGACCGA	91475	0.4240755882342169	No Hit
TAGCTTATCAGACTGATGTTGACA	89305	0.41401552781914996	No Hit
ACCGGGTGCTGTAGGCTT	89058	0.41287044260139816	No Hit
TGAGGTAGTAGATTGTATAGTT	88441	0.41001004754328924	No Hit
ATACATGATGATCTCACACAACTTGAACTCTCTCAC	87066	0.4036355852987191	No Hit
AACTGTGATGAAAGATTTGGTCTGTATGTAAT	80788	0.3745309496831475	No Hit
CTGCAGTGATGACTTTCTTAGGACACCTTTGGATTT	78033	0.3617588453312998	No Hit
CTCACTGATGAGTACGTTCTGACTTTCGTTCTTCTG	77777	0.36057203636067436	No Hit
TGAGGTAGTAGTTTGTGCTGTT	76754	0.35582943645071424	No Hit
TACCCTGTAGATCCGAATTTGT	75468	0.3498675757623382	No Hit
CTTAATGATGACTGTTTTTTTTGATTGCTTGAAGCA	70516	0.32691023973680283	No Hit
CTCCTACTTGGATAACTGTGGTAATTCTAGAGCTAA	69899	0.32404984467869397	No Hit
GATGGGAGACCGCCTGGGAATACCGGGTGCTGTAGG	66383	0.30774976522276054	No Hit
CGCGACCTCAGATCAGACGTGGCGACCCGCTGAATT	66109	0.3064795087463881	No Hit
TACGGGGATGATTTTACGAACTGAACTCTCTCTTTC	60764	0.2817002355120411	No Hit
TTGCATGATGACTTGAATTGTCGGATACCCCTTCAC	58773	0.27247001418190364	No Hit
GGCTGGTCCGATGGTAGTGGGTTATCAGAACT	56402	0.261478123285994	No Hit
CTGACCTATGAATTGACAGCCAT	55729	0.2583581137655608	No Hit
CAGGACGGTGGCCATGGAAGTCGGAATCCGCTAAGG	55598	0.2577508013626236	No Hit

Sequence	Count	Percentage	Possible Source
TTCAAGTAATCCAGGATAGGCT	55548	0.2575190027355483	No Hit
CTGACCTATGAATTGACAGCC	53752	0.2491927960510044	No Hit
CACAGATGATGAACTTATTGACGGGCGGACAGAAAC	51396	0.2382704447432174	No Hit
ATTCAAATCGATCTGCGCCTTC	49828	0.23100123979813678	No Hit
ACCGGGTGCTGTAGGCTTT	49107	0.22765870359571128	No Hit
CGCTGCGATCTATTGAAAGTCAGCCCTCGACACAAG	48596	0.225289721627002	No Hit
ACAAATGATGAATAACAAAGGGACTTAATACTG	48423	0.22448769837732155	No Hit
TAGCTTATCAGACTGATGTTGAT	47915	0.22213262432623673	No Hit
TTGCTGTGATGACTATCTTAGGACACCTTTGGATTA	47613	0.22073256061870208	No Hit
CGCGACCTCAGATCAGACGC	47120	0.21844702615573985	No Hit
ATTCAAATCGATCTGCGCCTT	43754	0.2028423425810323	No Hit
GCATTGGTGGTTCAGTGGTAGAATTCTCGCCT	43381	0.20111312482305072	No Hit
CTGCTGTGATGACATTCCAATTAAAGCACGTGTTAG	42956	0.19914283649291087	No Hit
CTGCTCTGATGAAATCACTAATAGGAAGTGCCGTCA	42662	0.19777986056570823	No Hit
CACCAGTGATGAGTTGAATACCGCCCCAGTCTGATC	42640	0.19767786916979513	No Hit
TGGAAGACTAGTGATTTTGTTGT	41281	0.19137758248588915	No Hit
TCTCCTACTTGGATAACTGTGGTAATTCTAGAGCTA	41146	0.1907517261927859	No Hit
TACCCTGTAGATCCGAATTTGTG	40151	0.18613893351398791	No Hit
TTCAAATCGAACTGCGCCTTT	39858	0.1847805935593268	No Hit
TTCAAATCGATCTGCGCCTTT	39438	0.18283348509189448	No Hit
TTTGCATGATGACTTGAATTGTCGGATACCCCTTCA	37955	0.17595833781284181	No Hit
CACCCTGATGAACTGAATACCGCCCCAGTCTGATAG	37905	0.17572653918576653	No Hit
TAATACTGCCTGGTAATGATGAC	37486	0.17378406669087573	No Hit
GAGAAGACGGTCGAACTTGACTATCT	36800	0.17060378952740293	No Hit
TCGCGTGATGACATTCTCCGGAATCGCTGTACGGCC	36260	0.16810036435498996	No Hit
CCTCACTGATGAGTACGTTCTGACTTTCGTTCTTCT	35374	0.16399289268321607	No Hit
GCATTGGTGGTTCAGTGGTAGAATTCTCGCC	34678	0.1607662557943282	No Hit
TGGAATGATGACATTCTCCGGAATCGCTGTACTGAC	34591	0.16036292618321726	No Hit
TGGGAGACCGCCTGGGAATACCGGGTGCTGTAGGCT	34321	0.15911121359701075	No Hit
TGTAAACATCCCCGACTGGAAGC	33011	0.15303808956763854	No Hit
AGAAATGAAGAAACTAAAATTGGTCTTAGTATTGAA	32752	0.1518373726793886	No Hit
TCGTACGACTCTTAGCGGTGGATCACTCGGCTCGTG	32199	0.14927367986393605	No Hit
GCAGCTGATGATACAGCTTCTTTCCCCATC	32124	0.14892598192332315	No Hit
ACGGCCCTGGCGGAGCGCTGAGAAGACGGTCGAACT	31721	0.1470576849890964	No Hit
TGCTGTGATGAGATGACTAAGTAGGAAGTGCCGTCA	31604	0.14651527620174026	No Hit
TGAGATGAAGCACTGTAGCTC	31546	0.14624638979433294	No Hit
TGAGGTAGTAGGTTGTATAGTT	31448	0.14579206448526544	No Hit

Sequence	Count	Percentage	Possible Source
GACTCTTAGCGGTGGATCACTCGGCTCGTGCGTCGA	31445	0.1457781565676409	No Hit
TGGAATGTAAAGAAGTATGTAT	31172	0.1445125360638099	No Hit
GCATATGATGGAAAAGTTTTAATCTCCTGACACTTG	30960	0.14352970988501074	No Hit
TGTAAACATCCCCGACTGGAAGCT	30859	0.14306147665831867	No Hit
CGACTCTTAGCGGTGGATCACTCGGCTCGTG	30834	0.14294557734478103	No Hit
ATCTGTGATGATCTTATCCCGAACCTGAACTTCTGT	29753	0.13793409102741358	No Hit
CTGCGATGATGGCATTTCTTAGGACACCTTTGGATT	29631	0.1373685023773499	No Hit
CTCCCATGATGTCCAGCACTGGGCTCTGATCACCCC	29220	0.13546311766279112	No Hit
ACCCTGTAGATCCGAATTTGTG	28851	0.13375244379497558	No Hit
TGAGGTAGTAGTTTGTACAGTT	28279	0.13110066750123445	No Hit
GCTAATGATGGAAAAATCATTATTGGAAAAGAATGA	27338	0.12673821733967774	No Hit
GCTTAATGATGACTGTTTTTTTTGATTGCTTGAAGC	27271	0.12642760717939688	No Hit
GAGAAGACGGTCGAACTTGACTATCTAGAGGAAGTA	27271	0.12642760717939688	No Hit
GTGTATGATGACAACTCGGTAATGCTGCATACTCCC	27260	0.1263766114814403	No Hit
ACAGATGATGAACTTATTGACGGGCGGACAGAAACT	27064	0.12546796086330525	No Hit
AAGCTATGATGAATTTGATTGCATTGATCGTCTGAC	26892	0.12467057358616629	No Hit
TGGAAGACTAGTGATTTTGTTGTC	25605	0.11870407692524869	No Hit
ATGTTATGATGATGGGCGAAATGTTCAACTGCTCTG	25161	0.11664570511682026	No Hit
CTGAATGATGATATCCCACTAACTGAGCAGTCAGTA	25007	0.1159317653454284	No Hit
TTGCTGTGATGACTATCTTAGGACACCTTTGGAATA	24542	0.11377603811362834	No Hit
TGCTATGATGAAGGCTATGTTGGTAGGGACAACTGA	24464	0.11341443225539091	No Hit
AGCAGCATTGTACAGGGCTATGA	24447	0.11333562072218531	No Hit
CCACAATGATGACAGTTTATTTGCTACTCTTGAGTG	24306	0.11268194859383304	No Hit
CTTCTATGATGATTTTATCAAAATGACTTTCGTTCT	24037	0.11143487198016805	No Hit
AGCAAATGATGATAAACTGGATCTGACTGACTGTGC	24026	0.1113838762822115	No Hit
CGCGACCTCAGATCAGACG	23608	0.10944603975986218	No Hit
AATACATGATGATCTCAATCCAACTTGAACTCTCTC	23608	0.10944603975986218	No Hit
TTCACAGTGGCTAAGTTCTGC	23310	0.10806451994249354	No Hit
TAACACTGTCTGGTAACGATGTT	23197	0.10754065504530341	No Hit
TCCTACTTGGATAACTGTGGTAATTCTAGAGCTAAT	23119	0.10717904918706599	No Hit
TAGCTTATCAGACTGATGTTG	23112	0.10714659737927545	No Hit
GGCTAATGATGGAAAAATCATTATTGGAAAAGAATG	23033	0.1067803555484965	No Hit
TGTAAACATCCCCGACTGGAAG	22936	0.10633066621197047	No Hit
TTCAAATCGATCTGCGCCTTTT	22653	0.10501868598272442	No Hit
TAGGGTGATGAAAAAGAATCCTTAGGCGTGGTTGTG	22440	0.10403122383138375	No Hit
ATATATGATGACTTAGCTTTTTTCCCCGAC	22402	0.10385505687480655	No Hit
TTCAAATCGAACTGCGCCTTTT	22398	0.10383651298464051	No Hit

Adapter Content

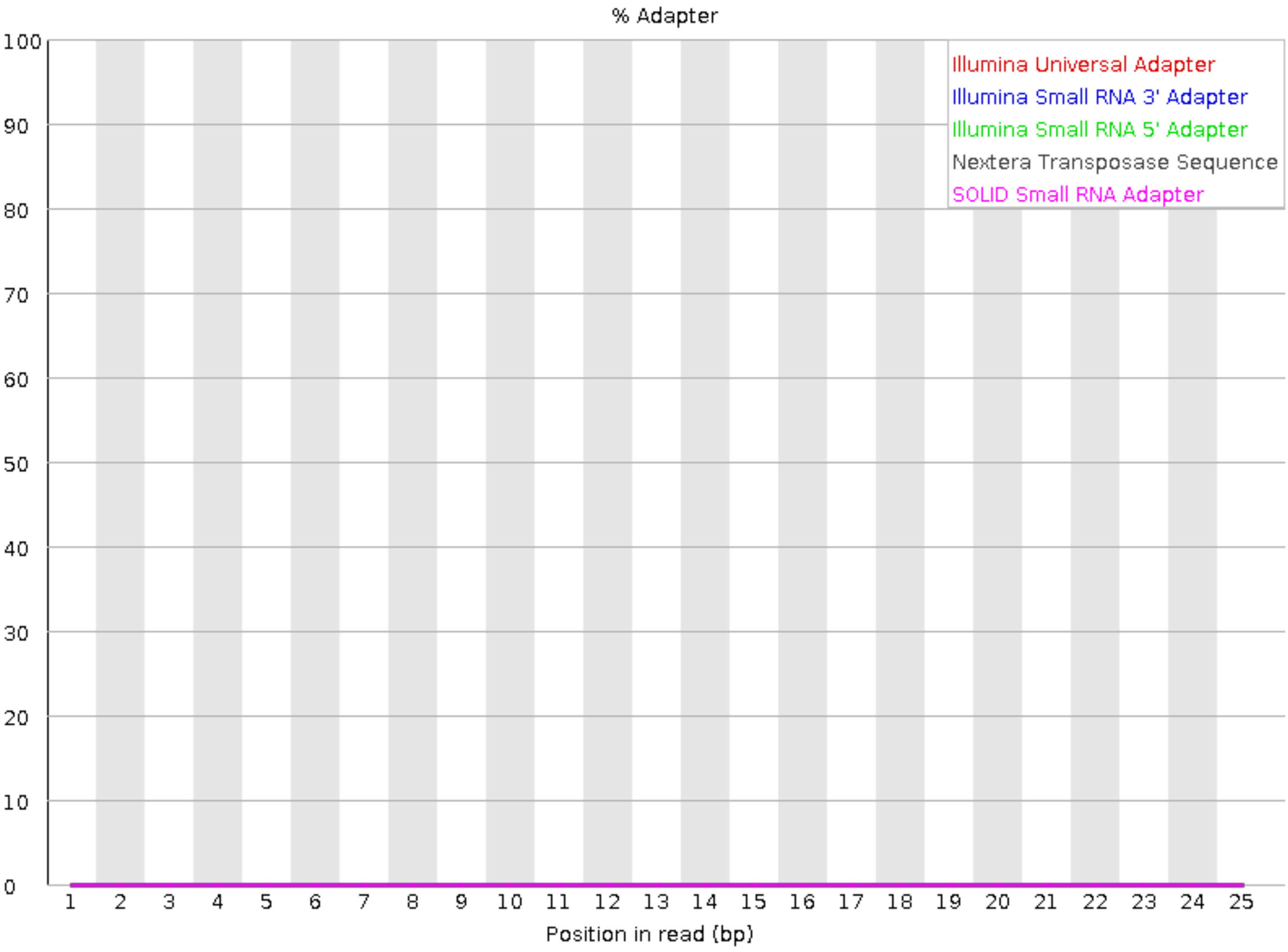

Produced by FastQC (version 0.11.9)
