# Supplementary material for: CD44 knockdown alters miRNA expression and their target genes in colon cancer
Source: Front Immunol. 2025 May 14;16:1552665. doi: 10.3389/fimmu.2025.1552665 (PMC12116639; doi:10.3389/fimmu.2025.1552665)
Supplement: Supplementary file 5 [file DataSheet5.zip › QC reports/shLUC_7.fastq.gz FastQC Report.pdf]

FastQC Report

Mon 31 Mar 2025
shLUC_7.fastq.gz

Summary

- [Basic Statistics]
- [Per base sequence quality]
- [Per tile sequence quality]
- [Per sequence quality scores]
- [Per base sequence content]
- [Per sequence GC content]
- [Per base N content]
- [Sequence Length Distribution]
- [Sequence Duplication Levels]
- [Overrepresented sequences]
- [Adapter Content]

Basic Statistics

Measure	Value
Filename	shLUC_7.fastq.gz
File type	Conventional base calls
Encoding	Sanger / Illumina 1.9
Total Sequences	25066271
Sequences flagged as poor quality	0
Sequence length	18-36
%GC	48

Per base sequence quality

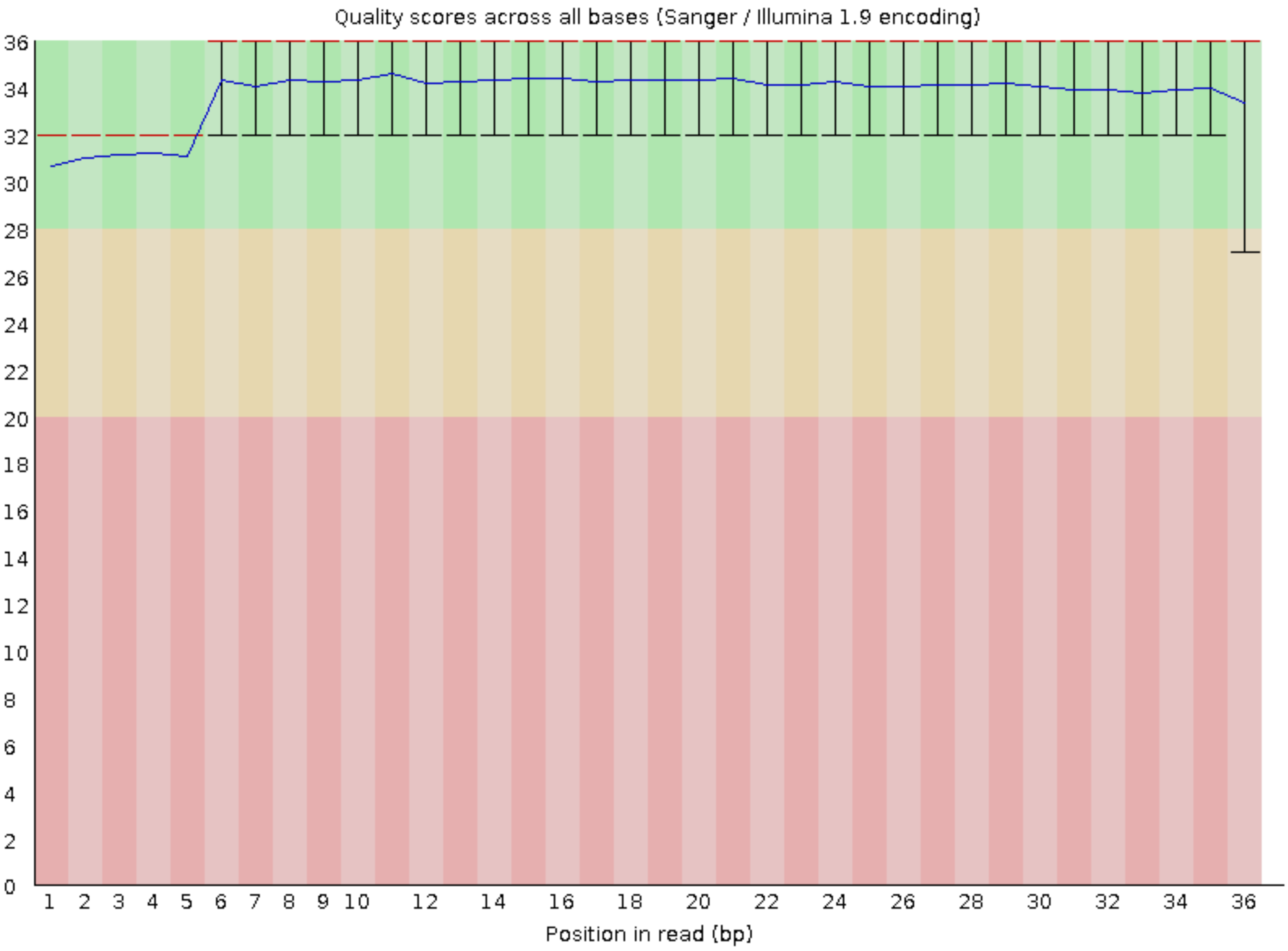

Per tile sequence quality

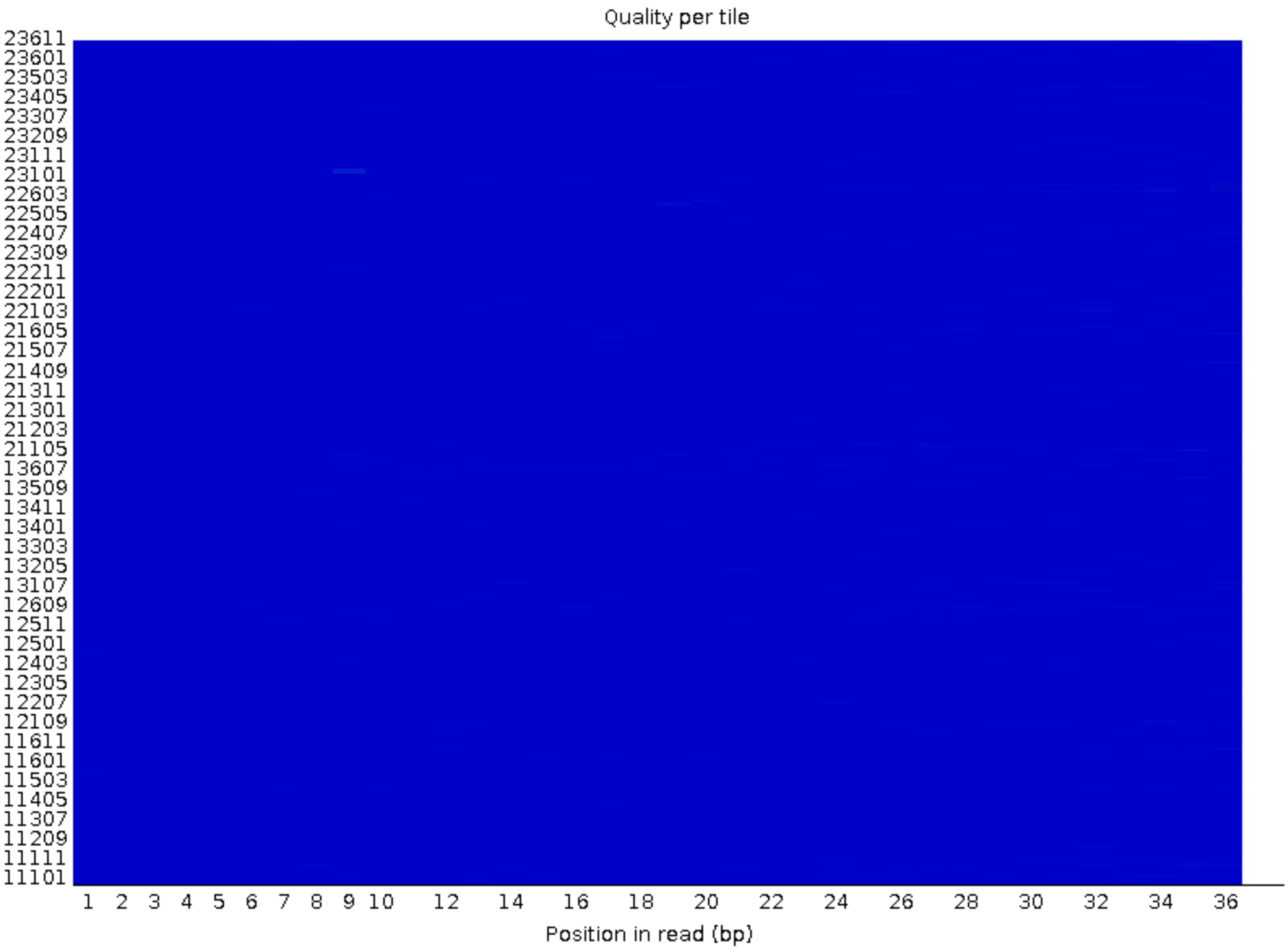

Per sequence quality scores

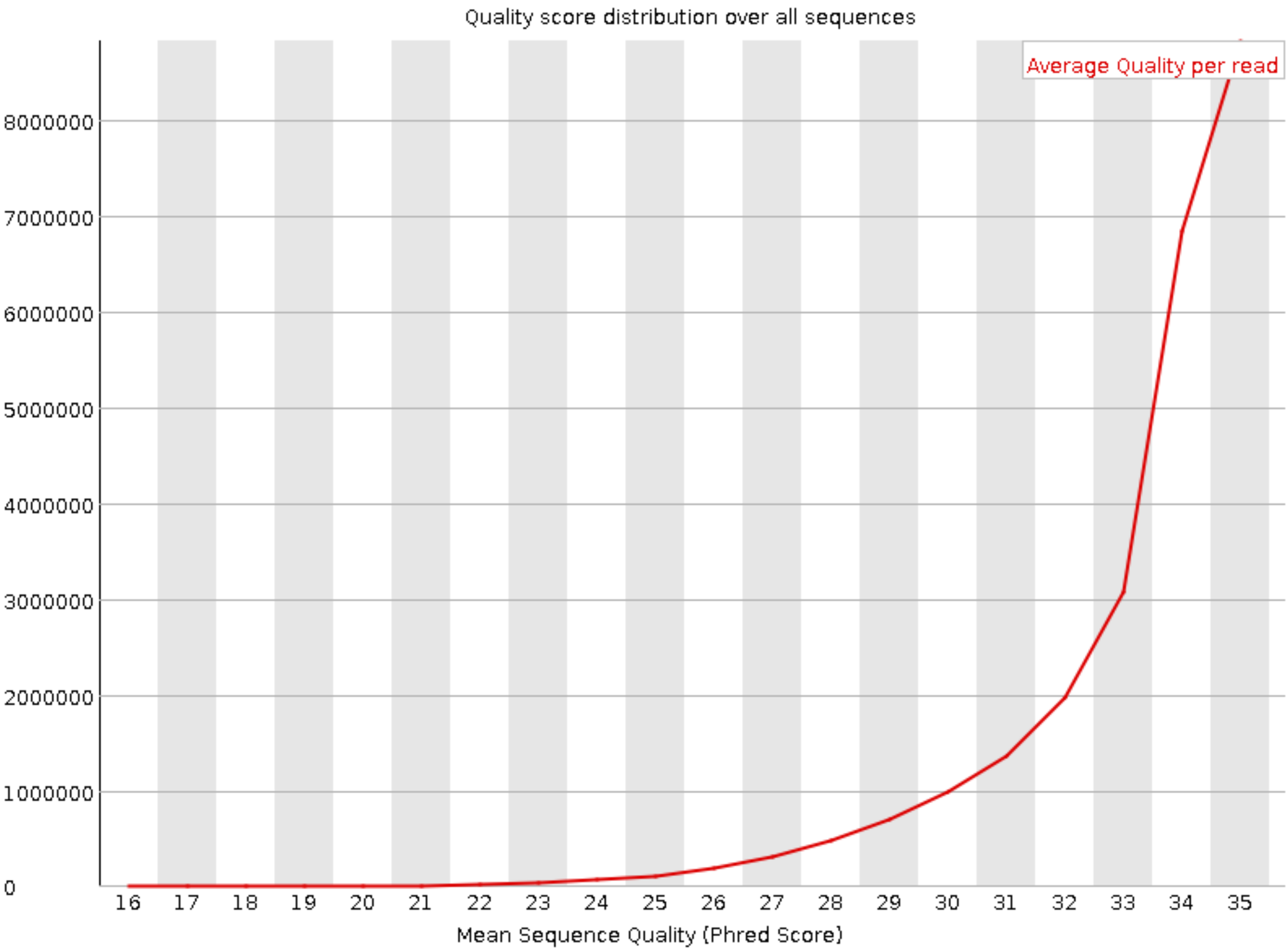

Per base sequence content

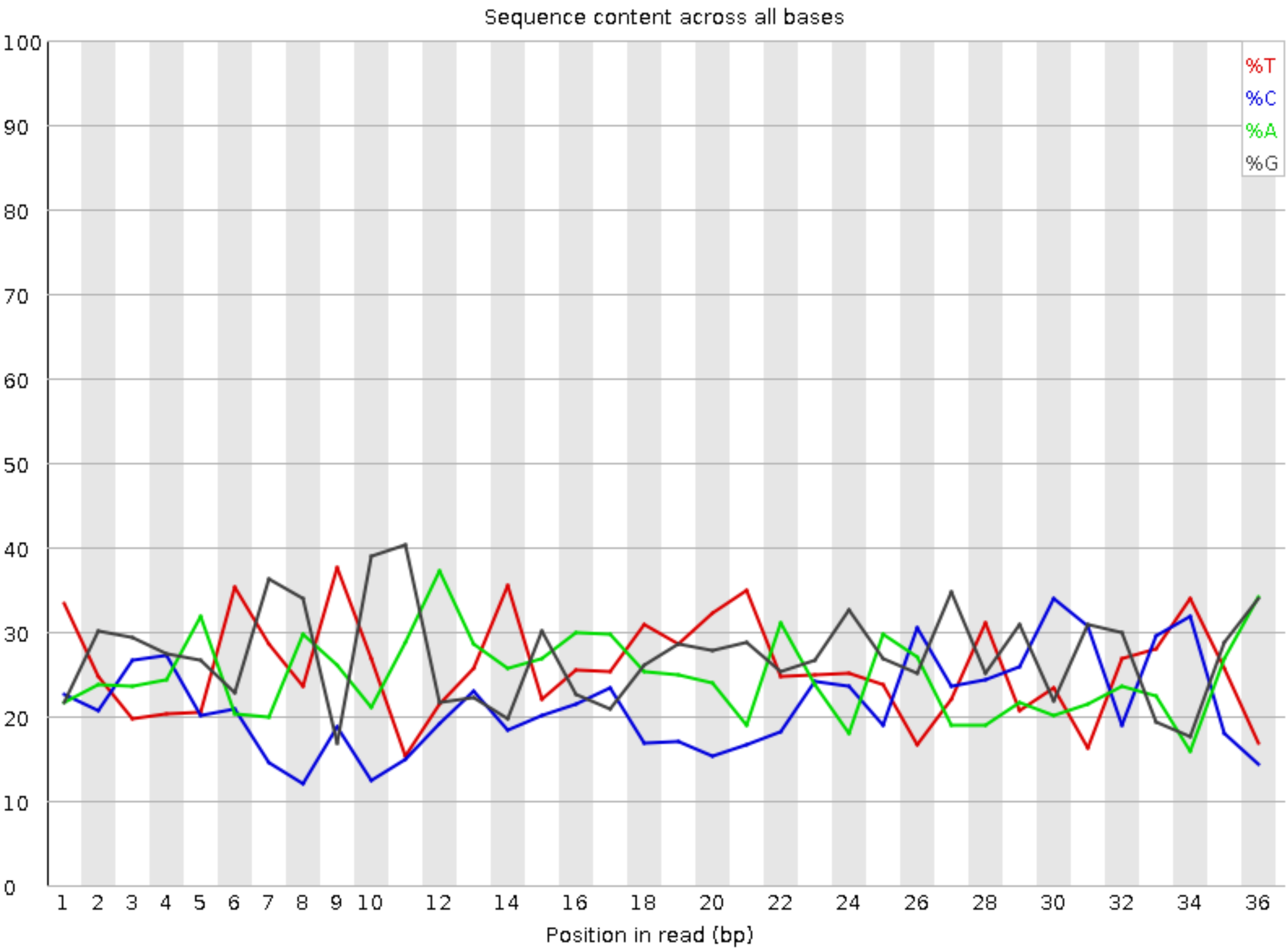

Per sequence GC content

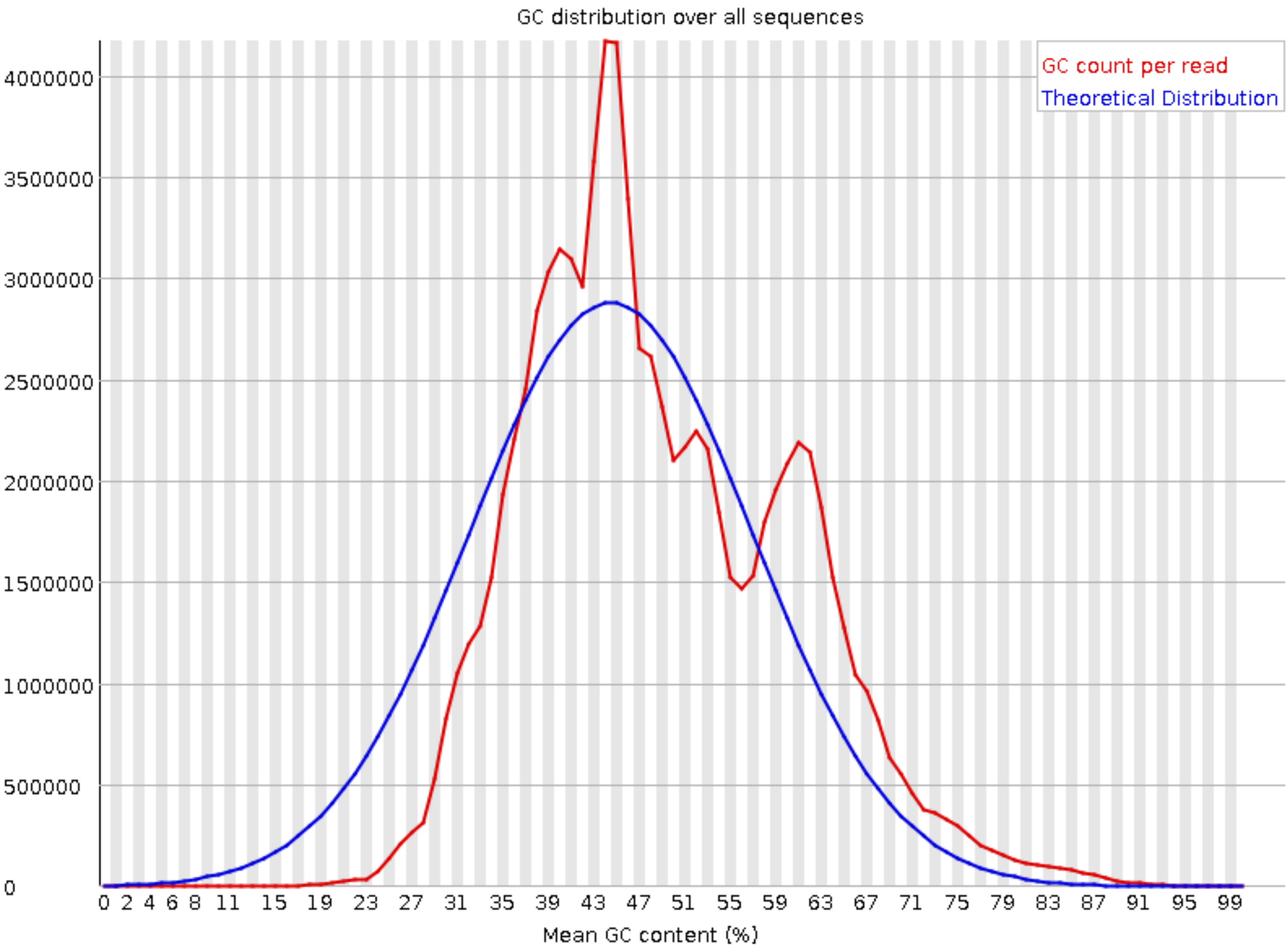

Per base N content

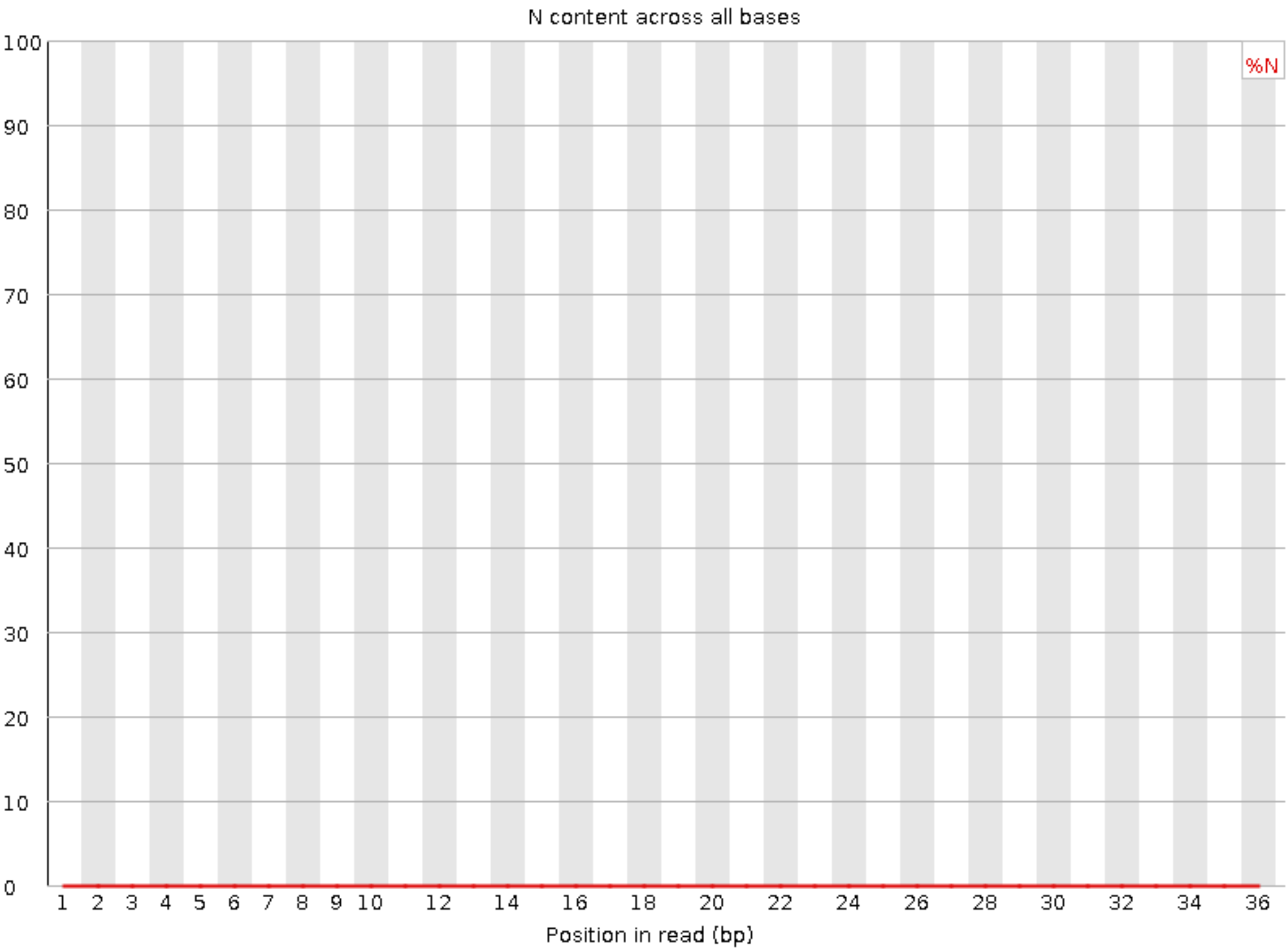

Sequence Length Distribution

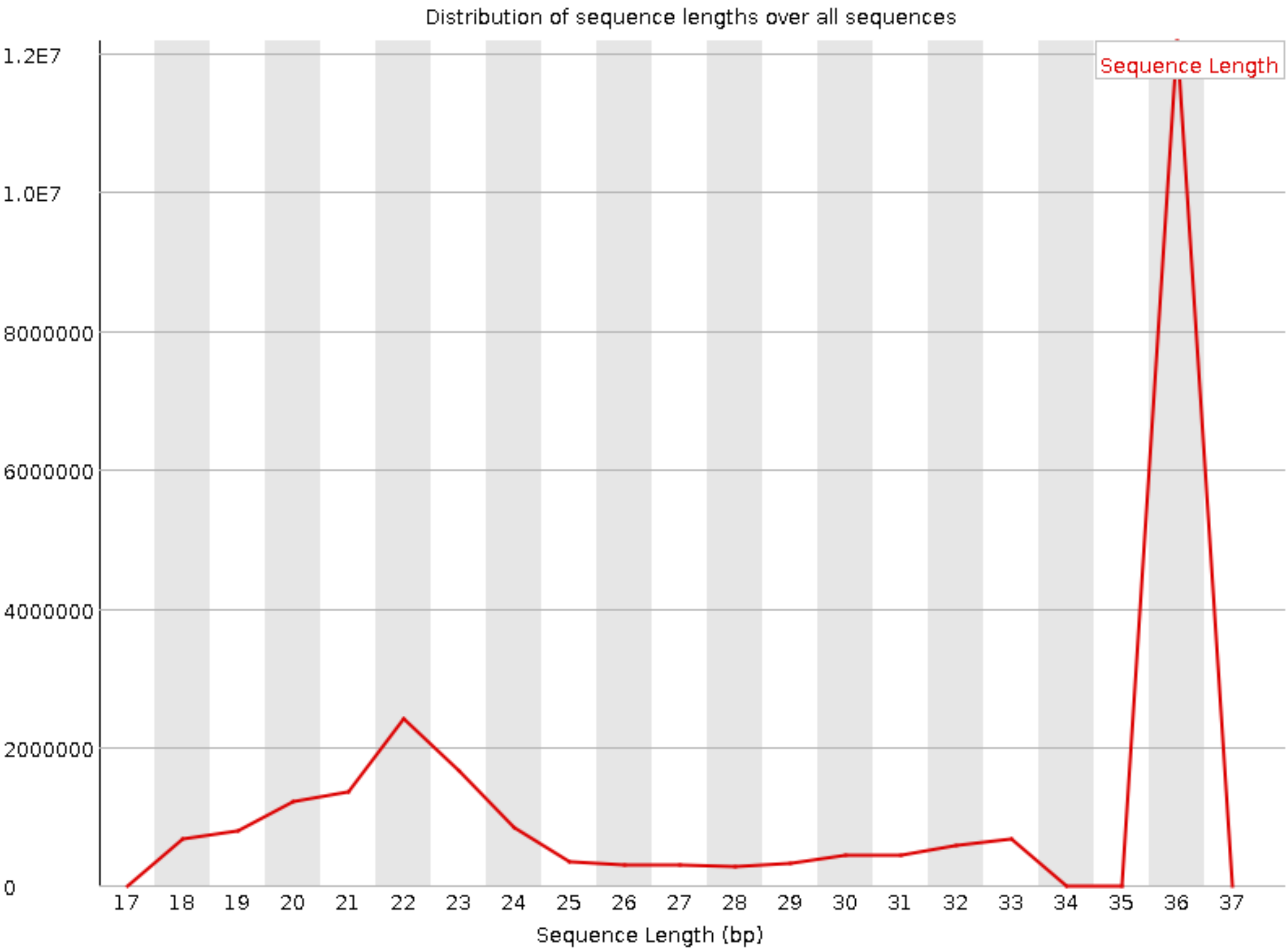

Sequence Duplication Levels

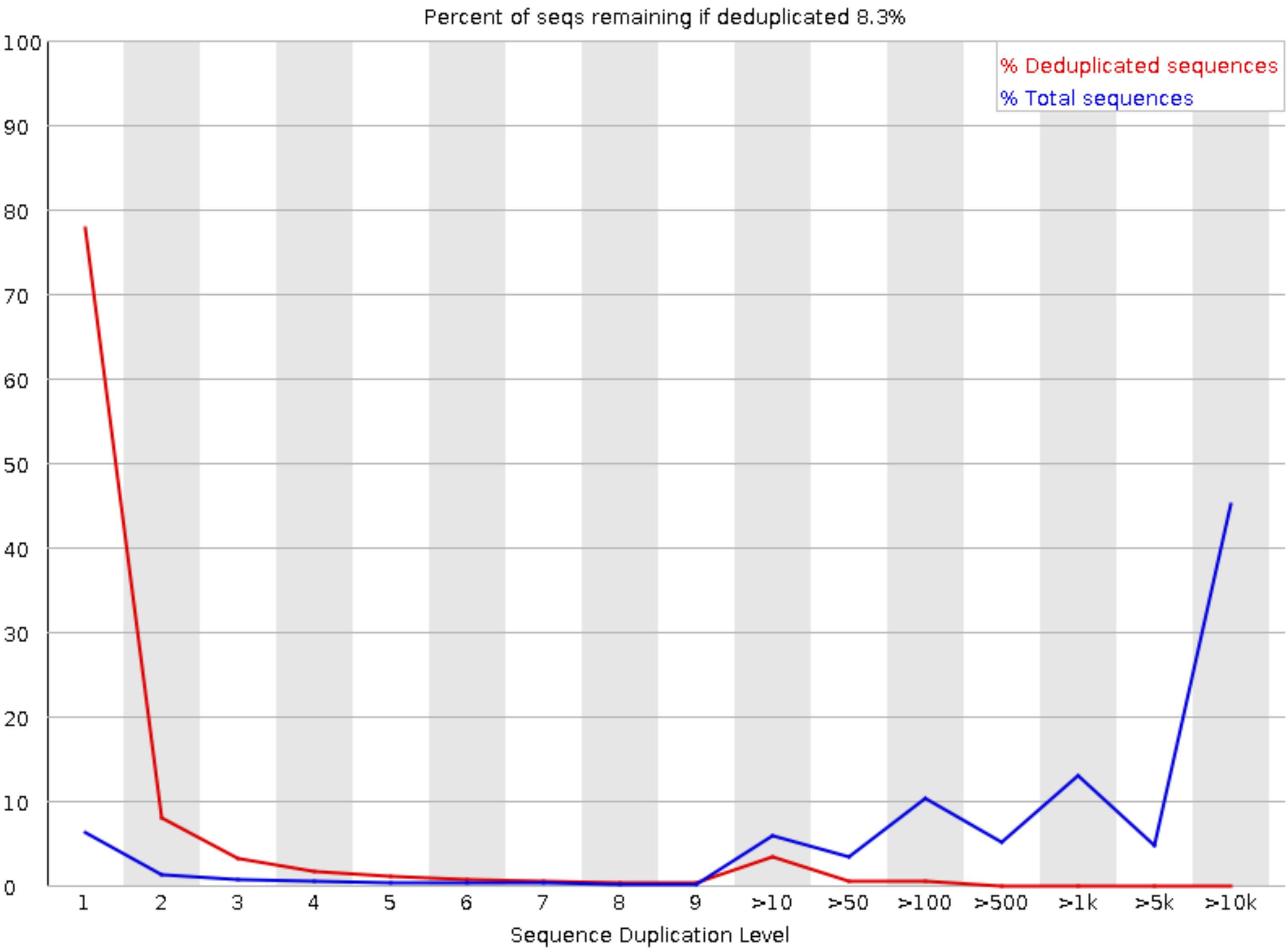

Overrepresented sequences

Sequence	Count	Percentage	Possible Source
TGCTCTGATGAAATCACTAATAGGAAGTGCCGTCAG	759301	3.029174144012087	No Hit
GTTTGTGATGACTTACATGGAATCTCGTTCGGCTGA	475206	1.8957985413945297	No Hit
GTGAAATGATGGCAATCATCTTTCGGGACTGACCTG	354674	1.4149452066484083	No Hit
TAGCTTATCAGACTGATGTTGAC	309129	1.2332468598939188	No Hit
CGCGACCTCAGATCAGACGT	303750	1.2117877445751704	No Hit
TAGCTTATCAGACTGATGTTGA	282247	1.1260031458209319	No Hit
GCCTCTGATGAAGCCTGTGTTGGTAGGGACATCTGA	247384	0.9869198334287538	No Hit
CCTGGATGATGATAAGCAAATGCTGACTGAACATGA	246710	0.9842309611988157	No Hit
AGTAGTGATGAAATTCCACTTCATTGGTCCGTGTTT	220260	0.8787106785847802	No Hit
CGACTCTTAGCGGTGGATCACTCGGCTCGTGCGTCG	196959	0.7857530942675917	No Hit
TATCTGTGATGATCTTATCCCGAACCTGAACTTCTG	182180	0.7267933870179574	No Hit
TTGAATGATGACTTTAATTGTCGGATACCCCTTCAC	177327	0.707432709077469	No Hit

Sequence	Count	Percentage	Possible Source
GTGCAATGATGTATTTTATTCAACACATCATTCTGA	173674	0.6928593407451791	No Hit
TGAGGTAGTAGATTGTATAGTT	152125	0.6068912284559598	No Hit
CAGGACGGTGGCCATGGAAGTCGGAATCCGCTAAGG	150526	0.6005121384030356	No Hit
TCGCTGCGATCTATTGAAAGTCAGCCCTCGACACAA	150420	0.6000892593876449	No Hit
TTTCTATGATGAATCAAACTAGCTCACTATGACCGA	148743	0.5933989942101879	No Hit
TGAAATGATGGCAATCATCTTTCGGGACTGACCTGA	132036	0.5267476761900484	No Hit
ATACATGATGATCTCAATCCAACTTGAACTCTCTCA	123043	0.4908707801012763	No Hit
TGGAAGACTAGTGATTTTGTTGTT	122625	0.489203200587754	No Hit
CTACGGGGATGATTTTACGAACTGAACTCTCTCTTT	112773	0.44989938870444673	No Hit
CTGGATGATGATAAGCAAATGCTGACTGAACATGAA	109133	0.4353778828929121	No Hit
CGCTGCGATCTATTGAAAGTCAGCCCTCGACACAAG	92983	0.37094867441591134	No Hit
TGCCTCTGATGAAGCCTGTGTTGGTAGGGACATCTG	92000	0.3670270699618623	No Hit
ACTCCATGATGAACACAAAATGACAAGCATATGGCT	86647	0.3456716796846248	No Hit
ACCGGGTGCTGTAGGCTTT	85455	0.3409162854738146	No Hit
TTGGTACTAGCAACGCACTTT	83706	0.3339387817198657	No Hit
TGAGGTAGTAGTTTGTGCTGTT	82258	0.32816209479263986	No Hit
CTCGCTGCGATCTATTGAAAGTCAGCCCTCGACACA	79627	0.3176659184766653	No Hit
ACCGGGTGCTGTAGGCTT	77531	0.3093040843610124	No Hit
GCATTGGTGGTTCAGTGGTAGAATTCTCGCCT	74272	0.2963025493500808	No Hit
GCAAATGATGATAAACTGGATCTGACTGACTGTGCT	73290	0.29238493432070534	No Hit
TCAGATGATGAATTTAACTGTTCAACTGCTGAATGA	71412	0.28489279478387514	No Hit
TTTGAATGATGACTTTAATTGTCGGATACCCCTTCA	71125	0.283747829902581	No Hit
GTGAAATGATGGCAAATCATCTTTCGGGACTGACCT	63219	0.2522074384339019	No Hit
ACAAATGATGAATAACAAAGGGACTTAATACTG	61917	0.24701320750900682	No Hit
TGGAAGACTAGTGATTTTGTTGT	61472	0.24523791352930002	No Hit
AATGGATTTTTGGAGCAGG	60247	0.24035086830426433	No Hit
CTGCAGTGATGACTTTCTTAGGACACCTTTGGATTT	57956	0.23121109637727924	No Hit
CTAGACTGAAGCTCCTTGAGG	57226	0.22829881636562535	No Hit
CTGACCTATGAATTGACAGCC	57137	0.22794375756968396	No Hit
TGTAACAGCAACTCCATGTGGA	55787	0.222558034260461	No Hit
CTCCTACTTGGATAACTGTGGTAATTCTAGAGCTAA	53823	0.21472280420170997	No Hit
CACAGATGATGAACTTATTGACGGGCGGACAGAAAC	53678	0.21414433762405263	No Hit
CGCGACCTCAGATCAGACGTGGCGACCCGCTGAATT	52245	0.20842749206692932	No Hit
TAGCTTATCAGACTGATGTTGACT	51997	0.20743811474790166	No Hit
ATATATGATGACTTAGCTTTTTTCCCCGAC	51577	0.20576255638503232	No Hit
TAAAGTGCTTATAGTGCAGGTAG	51241	0.20442210969473681	No Hit
TGTAATGATGTTGATCAAATGTCTGACCTGAAATGA	48739	0.19444056916164354	No Hit

Sequence	Count	Percentage	Possible Source
TGAAATGATGGCAAATCATCTTTCGGGACTGACCTG	48317	0.19275703194942717	No Hit
TAGCTTATCAGACTGATGTTGACA	46967	0.18737130864020418	No Hit
TAGCTTATCAGACTGATGTTGAT	46206	0.18433535646367186	No Hit
ACGGCCCTGGCGGAGCGCTGAGAAGACGGTCGAACT	46100	0.18391247744828101	No Hit
AGCAGCATTGTACAGGGCTATGA	45173	0.1802142807759479	No Hit
CTCACTGATGAGTACGTTCTGACTTTCGTTCTTCTG	45066	0.17978741233588355	No Hit
GACTCTTAGCGGTGGATCACTCGGCTCGTGCGTCGA	42923	0.17123807526057624	No Hit
TAATACTGCCTGGTAATGATGAC	42627	0.1700572055572207	No Hit
TAGCTTATCAGACTGATGTTG	42524	0.16964629481585033	No Hit
GCAGCCGACTTAGAACTGGTGCGGACCAGGGGAATC	42302	0.16876064253833367	No Hit
TAATACTGCCTGGTAATGATGA	41376	0.16506643529067408	No Hit
TAGGGTGATGAAAAAGAATCCTTAGGCGTGGTTGTG	40507	0.16159962524940386	No Hit
TACCCTGTAGATCCGAATTTGT	40332	0.16090147593154164	No Hit
TAACACTGTCTGGTAACGATGTT	39993	0.15954906096722565	No Hit
TGAGGTAGTAGTTTGTACAGTT	39945	0.1593575685828977	No Hit
AACTGTGATGAAAGATTTGGTCTGTATGTAAT	39614	0.1580370690159697	No Hit
TCTCCTACTTGGATAACTGTGGTAATTCTAGAGCTA	39148	0.1561779971181194	No Hit
CGCGACCTCAGATCAGACGG	38937	0.1553362285120112	No Hit
TGAGGTAGTAGGTTGTATAGTT	38803	0.1548016456057624	No Hit
TCGTACGACTCTTAGCGGTGGATCACTCGGCTCGTG	37588	0.14995449462746174	No Hit
CTGAATGATGATATCCCACTAACTGAGCAGTCAGTA	37413	0.1492563453095995	No Hit
TAATACTGCCGGGTAATGATGGA	37041	0.14777227933105805	No Hit
TTCAAGTAATCCAGGATAGGCT	36628	0.14612464694090319	No Hit
TTCCTATGATGAGGACCTTTTCACAGACCTGTACTG	36417	0.14528287833479497	No Hit
TAGCTTATCAGACTGATGTTGG	35080	0.1399490175463275	No Hit
TCAGTGCACTACAGAACTTTGT	34732	0.13856069775995003	No Hit
CTTAATGATGACTGTTTTTTTTGATTGCTTGAAGCA	34017	0.13570825911839857	No Hit
GCATATGATGGAAAAGTTTTAATCTCCTGACACTTG	33896	0.13522553873290527	No Hit
CGCGACCTCAGATCAGACG	33690	0.13440371725016456	No Hit
CTCCATGATGAACACAAAATGACAAGCATATGGCTG	33373	0.13313906962866556	No Hit
GATGGGAGACCGCCTGGGAATACCGGGTGCTGTAGG	33320	0.13292763012097011	No Hit
GCATTGGTGGTTCAGTGGTAGAATTCTCGCC	33213	0.13250076168090577	No Hit
GCTTAATGATGACTGTTTTTTTTGATTGCTTGAAGC	33058	0.13188240085651354	No Hit
CGCGACCTCAGATCAGACGC	32937	0.13139968047102019	No Hit
GCATGGGTGGTTCAGTGGTAGAATTCTCGCCT	32738	0.13060578496099398	No Hit
TGCTATGATGAAGGCTATGTTGGTAGGGACAACTGA	32655	0.13027466271309363	No Hit
ATACATGATGATCTCACACAACTTGAACTCTCTCAC	32166	0.12832383404775285	No Hit

Sequence	Count	Percentage	Possible Source
CTGCGATGATGGCATTTCTTAGGACACCTTTGGATT	32133	0.1281921830335274	No Hit
TAATACTGTCTGGTAAAACCGT	31620	0.12614560817602266	No Hit
ATGGATTTTTGGAGCAGG	31605	0.12608576680592018	No Hit
TACGGGGATGATTTTACGAACTGAACTCTCTCTTTC	31119	0.12414690641459993	No Hit
ATGGATTTTTGGAAATAGGA	30914	0.12332907435653273	No Hit
AGACGTGGCGACCCGCTGAATTT	30644	0.12225192969468814	No Hit
TGGGAGACCGCCTGGGAATACCGGGTGCTGTAGGCT	30603	0.12208836328307468	No Hit
ATGACCTATGAATTGACAGACA	29818	0.11895666491437837	No Hit
TCGCGTGATGACATTCTCCGGAATCGCTGTACGGCC	29591	0.1180510655134942	No Hit
TTGGTACTAGCAACGCACTTTT	28845	0.11507495470706433	No Hit
ACAGATGATGAACTTATTGACGGGCGGACAGAAACT	28530	0.1138182859349123	No Hit
GCAGCTGATGATACAGCTTCTTTCCCCATC	28458	0.1135310473584204	No Hit
AGTCTGTGATGAATTGCTTTGACTTCTGACACCTCG	28021	0.11178766877610155	No Hit
TCAAATGATGAAATCACCCAAAATAGCTGGAATTAC	27914	0.11136080033603722	No Hit
CGACTCTTAGCGGTGGATCACTCGGCTCG	27678	0.11041929611309156	No Hit
ATGGATTTTTGGAAATAGG	27668	0.11037940186635659	No Hit
CAAAGTGCTTACAGTGCAGGTAG	27498	0.10970119967186184	No Hit
ACGACTCTTAGCGGTGGATCACTCGGCTCGTGCGTC	27452	0.10951768613688091	No Hit
TACCCTGTAGATCCGAATTTGTG	27197	0.1085003828451388	No Hit
AGAAATGAAGAAACTAAAATTGGTCTTAGTATTGAA	27174	0.10840862607764833	No Hit
CGGCCCTGGCGGAGCGCTGAGAAGACGGTCGAACTT	26880	0.10723573522363977	No Hit
TCGCGAAGGCCCGCGGCGGGTGTTGACGCGATGTGA	26818	0.10698839089388285	No Hit
TCTCGTGATGAAAACTCTGTCCAGTTCTGCTACTGA	26708	0.10654955417979803	No Hit
GGCTGGTCCGATGGTAGTGGGTTATCAGAACT	26653	0.1063301358227556	No Hit
TTCACAGTGGCTAAGTTCTGC	26613	0.10617055883581566	No Hit
CTGCTGTGATGACATTCCAATTAAAGCACGTGTTAG	26278	0.10483410157019367	No Hit
CTGACCTATGAATTGACAGCT	26128	0.1042356878691689	No Hit
TGTAAACATCCCCGACTGGAAG	25851	0.10313061723460981	No Hit
GAGAAGACGGTCGAACTTGACTATCTAGAGGAAGTA	25818	0.10299896622038436	No Hit
TGTTTGTGATGACTTACATGGAATCTCGTTCGGCTG	25378	0.10124361936404501	No Hit

Adapter Content

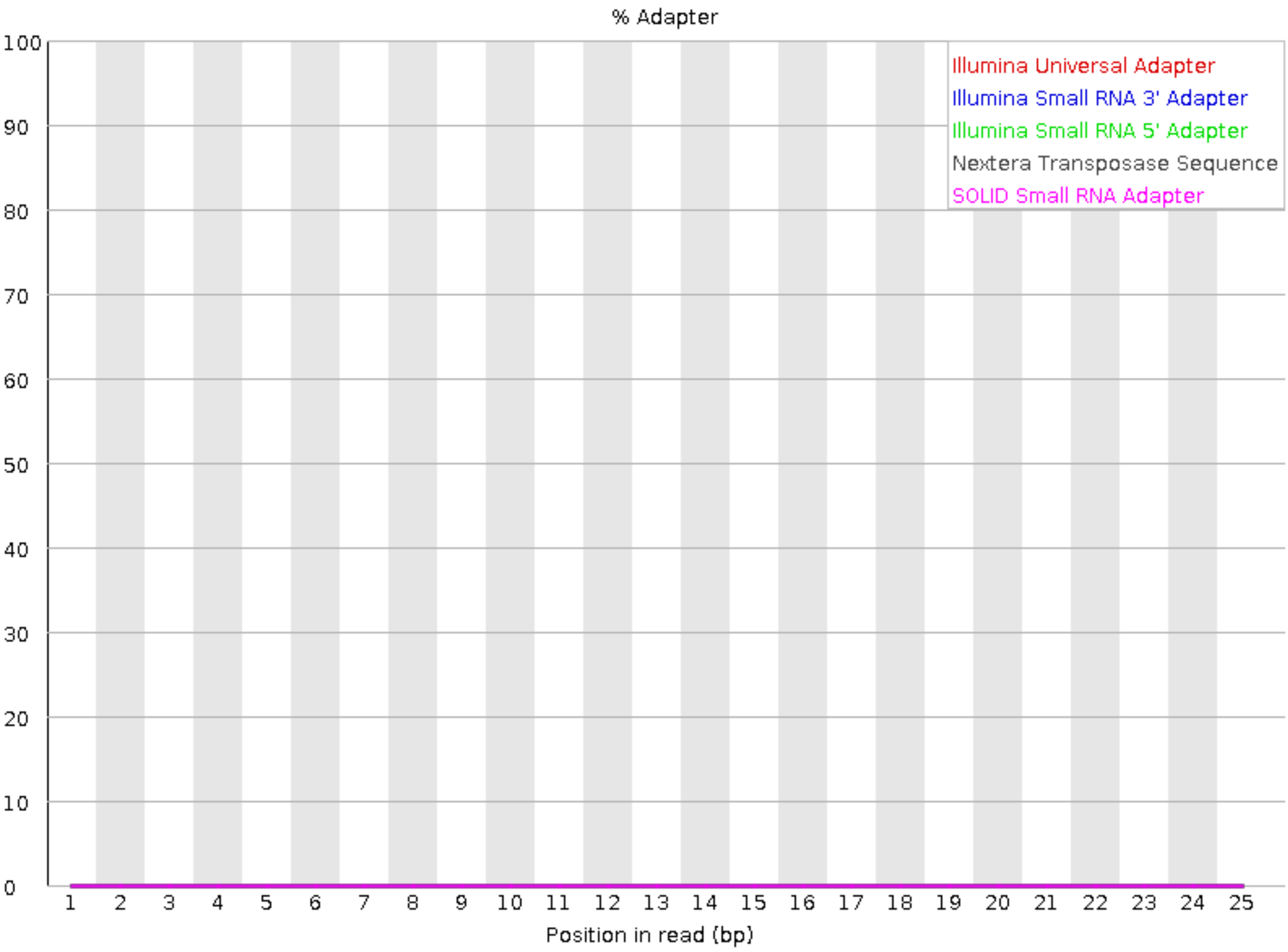
% Adapter
Illumina Universal Adapter
Illumina Small RNA 3' Adapter
Illumina Small RNA 5' Adapter
Nextera Transposase Sequence
SOLID Small RNA Adapter
100
90
80
70
60
50
40
30
20
10
0
1
2
3
4
5
6
7
8
9
10
11
12
13
14
15
16
17
18
19
20
21
22
23
24
25
Position in read (bp)
