# Supplementary material for: CD44 knockdown alters miRNA expression and their target genes in colon cancer
Source: Front Immunol. 2025 May 14;16:1552665. doi: 10.3389/fimmu.2025.1552665 (PMC12116639; doi:10.3389/fimmu.2025.1552665)

FastQC Report

Mon 31 Mar 2025
shCD44_4.fastq.gz

Summary

- Basic Statistics
- Per base sequence quality
- Per tile sequence quality
- Per sequence quality scores
- Per base sequence content
- Per sequence GC content
- Per base N content
- Sequence Length Distribution
- Sequence Duplication Levels
- Overrepresented sequences
- Adapter Content

Basic Statistics

Measure	Value
Filename	shCD44_4.fastq.gz
File type	Conventional base calls
Encoding	Sanger / Illumina 1.9
Total Sequences	18944590
Sequences flagged as poor quality	0
Sequence length	18-36
%GC	45

Per base sequence quality

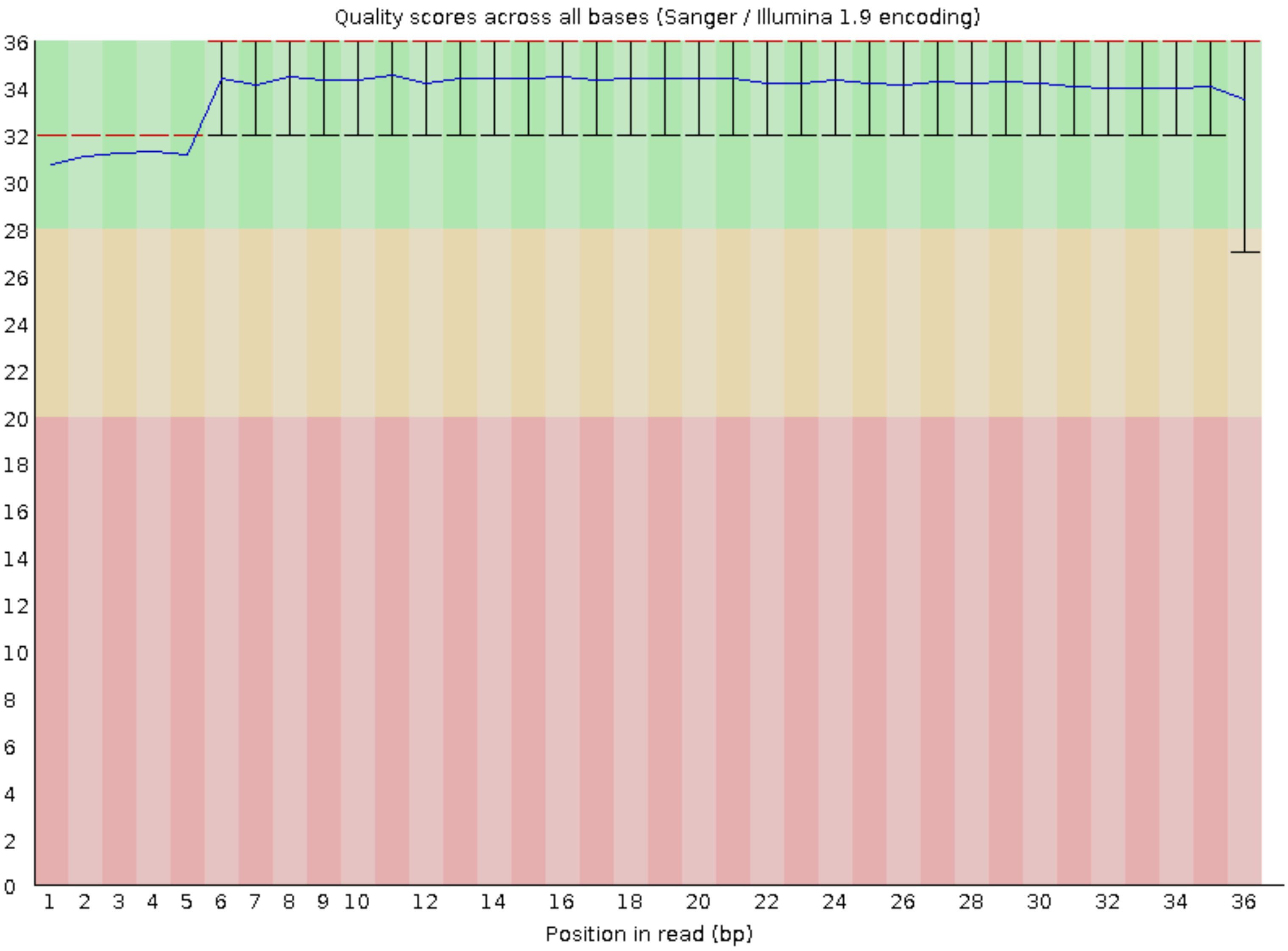

Per tile sequence quality

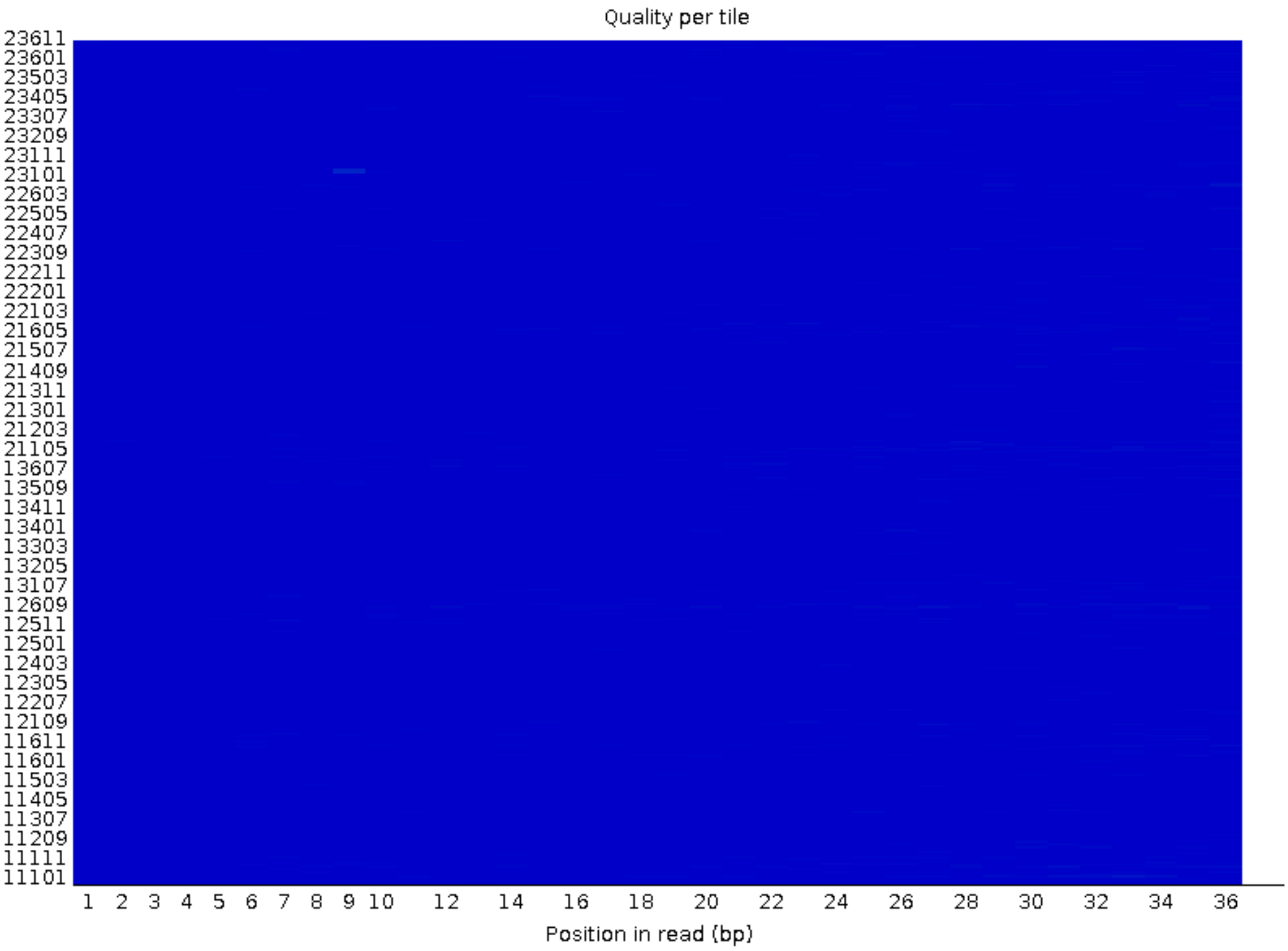

Per sequence quality scores

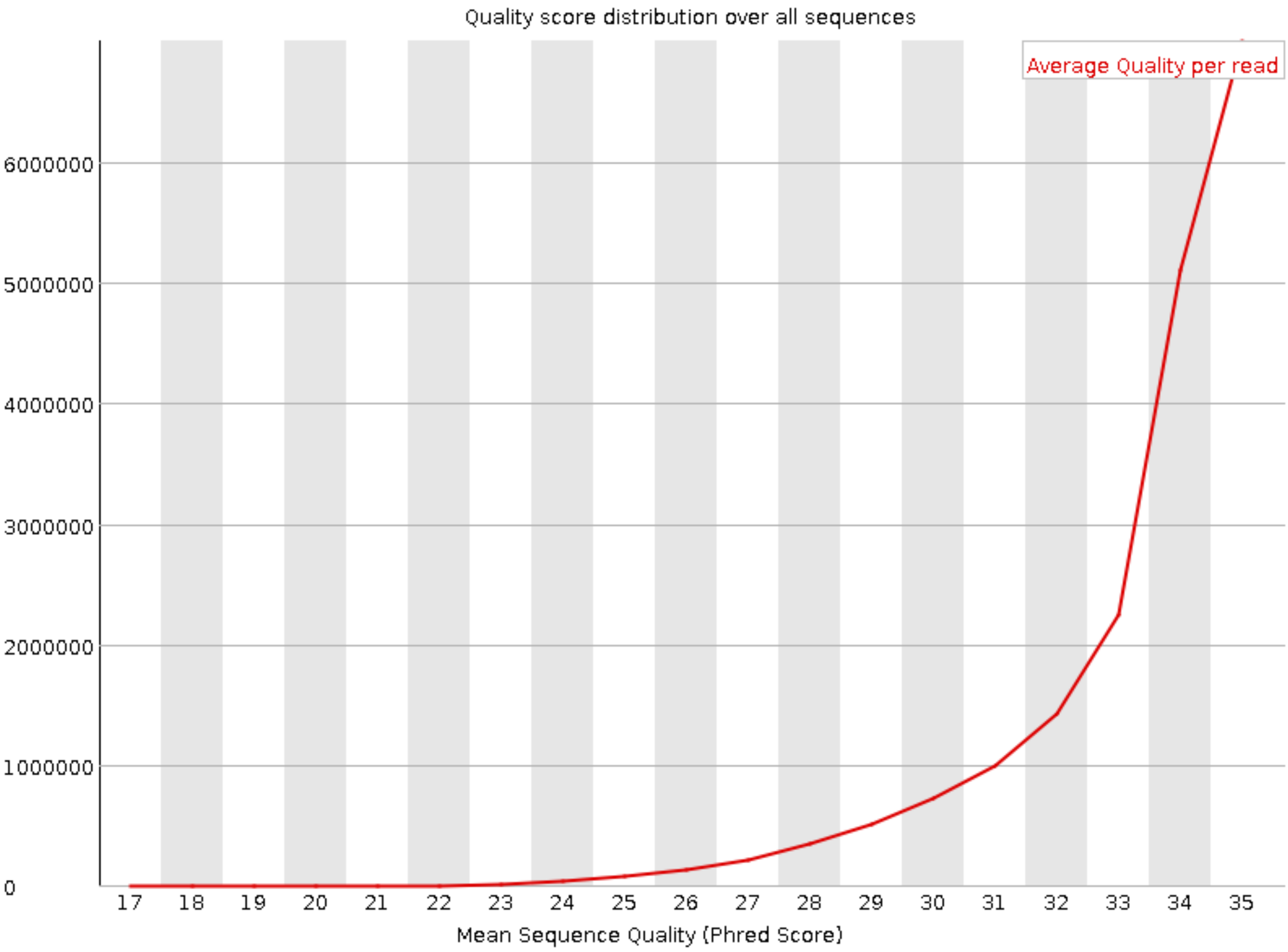

Per base sequence content

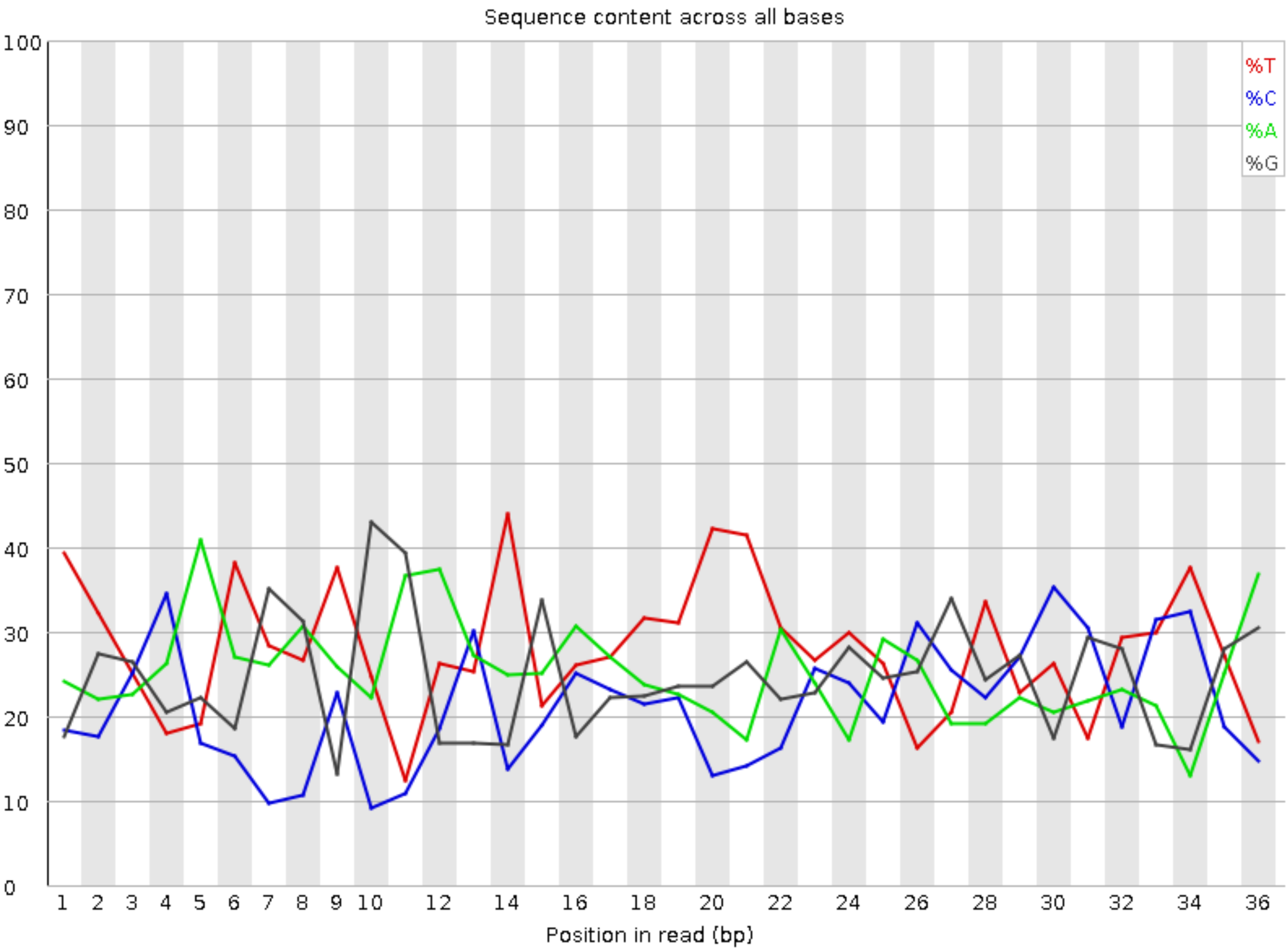

Per sequence GC content

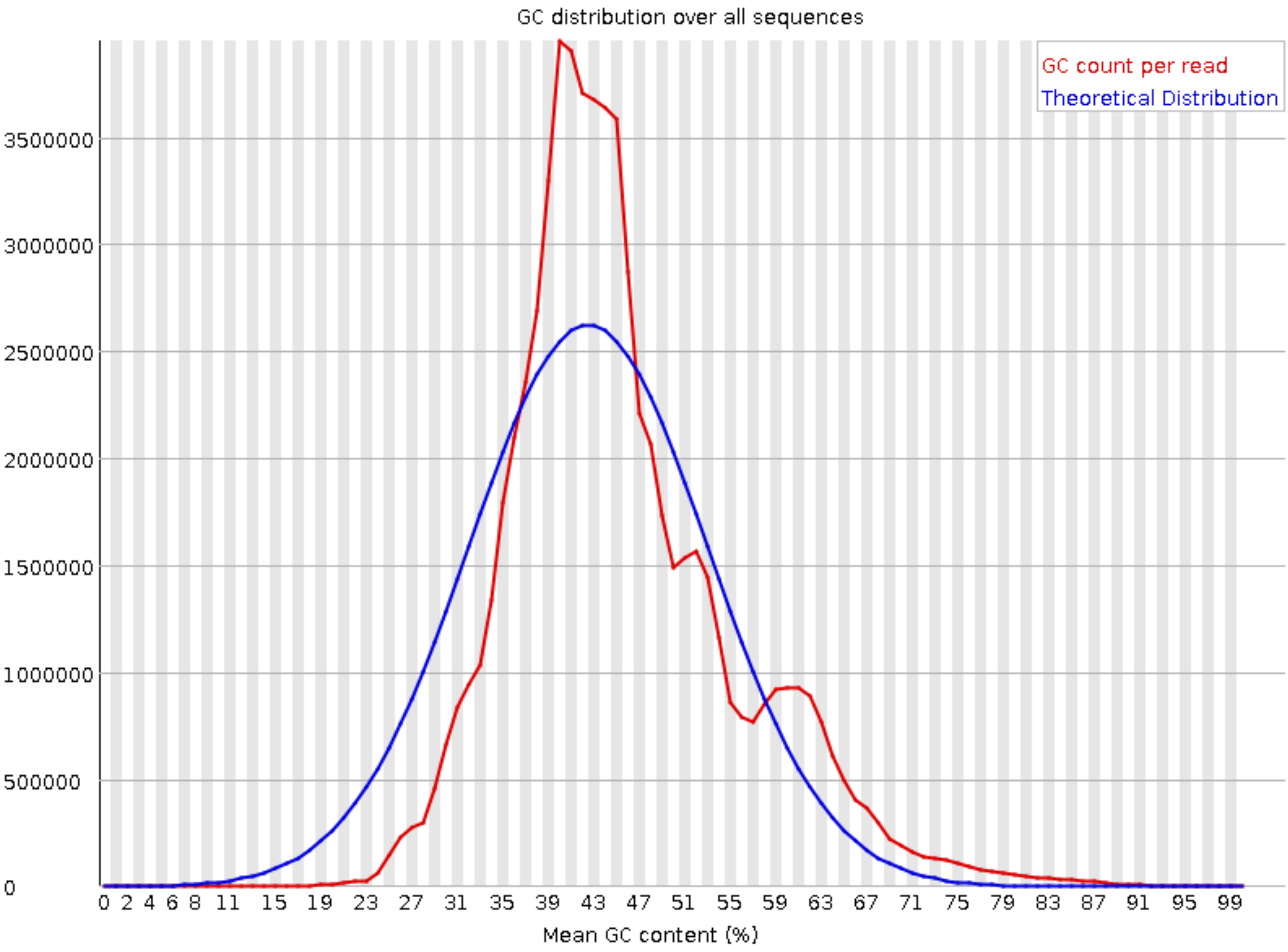

Per base N content

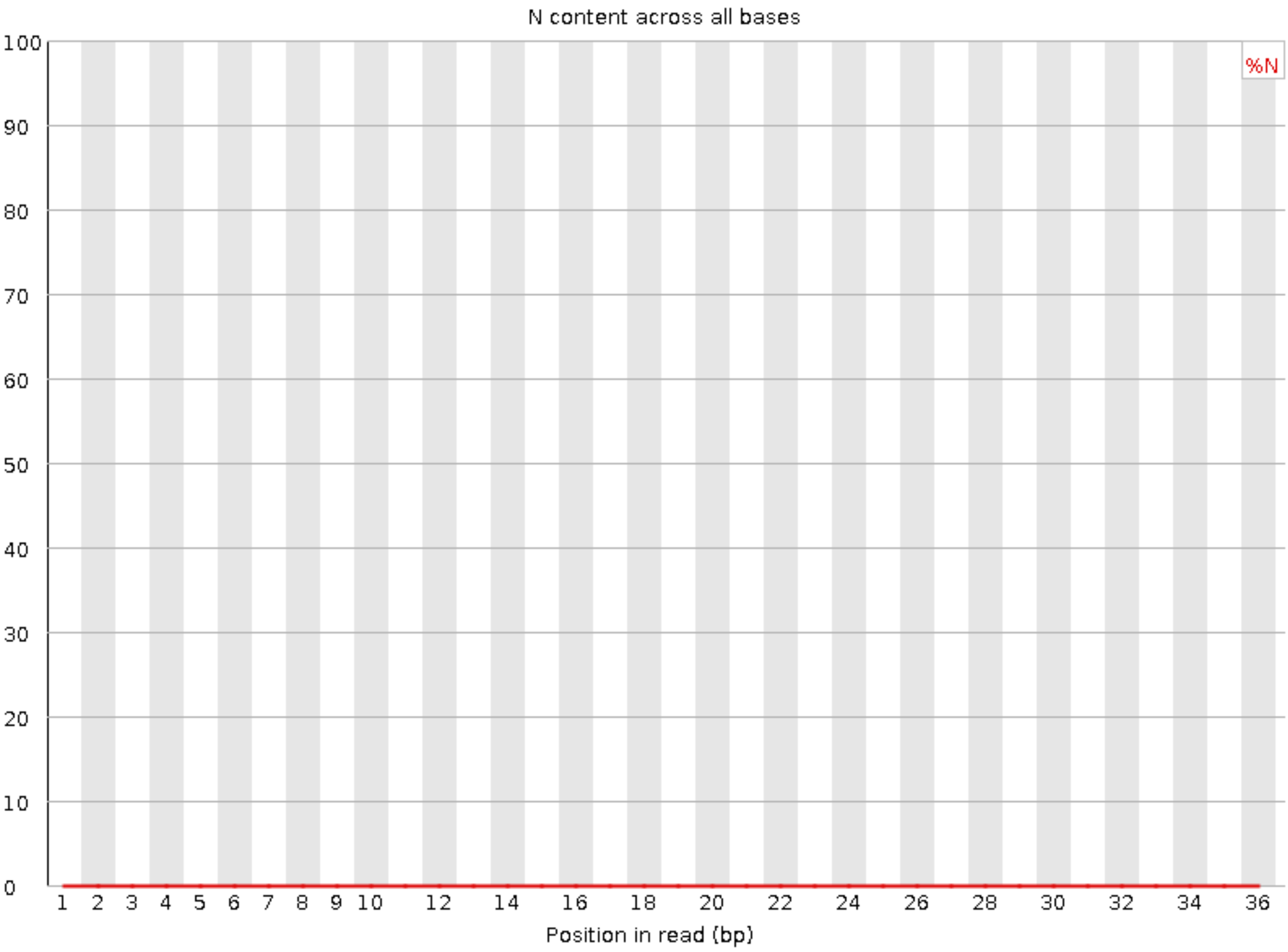

Sequence Length Distribution

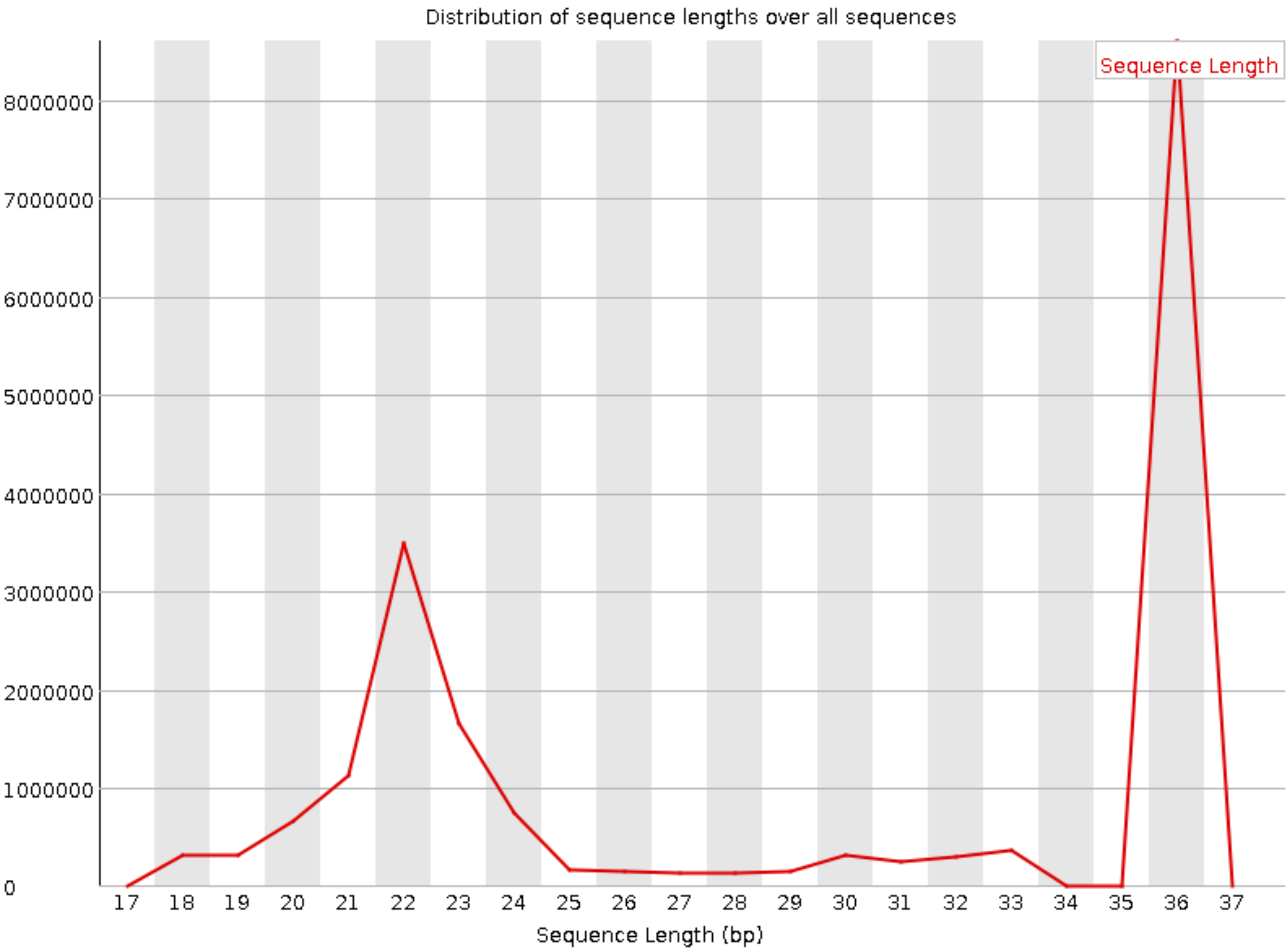

Sequence Duplication Levels

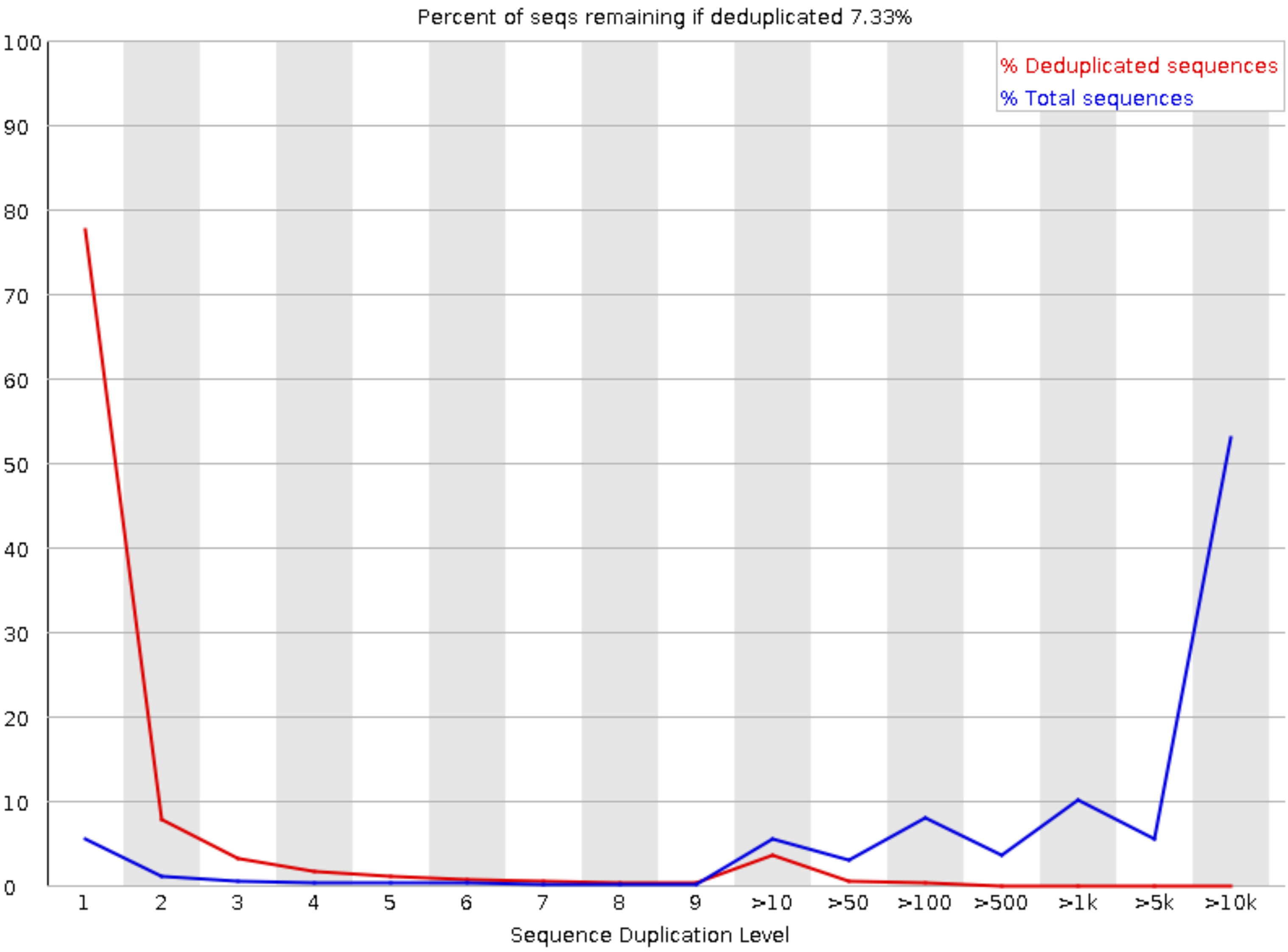

Overrepresented sequences

Sequence	Count	Percentage	Possible Source
ATTCAAATCGATCTGCGCCTTT	625100	3.299622741901514	No Hit
TGCTCTGATGAAATCACTAATAGGAAGTGCCGTCAG	523793	2.7648684927992635	No Hit
TAGCTTATCAGACTGATGTTGAC	378784	1.9994309721139385	No Hit
GTTTGTGATGACTTACATGGAATCTCGTTCGGCTGA	367070	1.9375980161090842	No Hit
GTGAAATGATGGCAATCATCTTTCGGGACTGACCTG	276968	1.4619899401359437	No Hit
TAGCTTATCAGACTGATGTTGA	254218	1.341902886259349	No Hit
CCTGGATGATGATAAGCAAATGCTGACTGAACATGA	244393	1.2900411146401163	No Hit
AGTAGTGATGAAATTCCACTTCATTGGTCCGTGTTT	215895	1.1396129449093382	No Hit
GCCTCTGATGAAGCCTGTGTTGGTAGGGACATCTGA	179970	0.9499809708206933	No Hit
ATTCAAATCGAACTGCGCCTTT	172172	0.9088188237380699	No Hit
ATACATGATGATCTCAATCCAACTTGAACTCTCTCA	162481	0.8576643780625498	No Hit
GTGCAATGATGTATTTTATTCAACACATCATTCTGA	157436	0.8310340841369489	No Hit

Sequence	Count	Percentage	Possible Source
CGCGACCTCAGATCAGACGT	150870	0.796375112895027	No Hit
TATCTGTGATGATCTTATCCCGAACCTGAACTTCTG	140863	0.7435526448447816	No Hit
TGGAAGACTAGTGATTTTGTTGTT	138888	0.7331275050027475	No Hit
TTTCTATGATGAATCAAACTAGCTCACTATGACCGA	138833	0.7328371846527162	No Hit
TTGAATGATGACTTTAATTGTCGGATACCCCTTCAC	132131	0.6974603303634441	No Hit
TGAAATGATGGCAATCATCTTTCGGGACTGACCTGA	100176	0.5287842069952424	No Hit
TCGCTGCGATCTATTGAAAGTCAGCCCTCGACACAA	99365	0.524503301470235	No Hit
CTGGATGATGATAAGCAAATGCTGACTGAACATGAA	99130	0.5232628417928284	No Hit
ATTCAAATCGATCTGCGCCTTC	95813	0.50575388541003	No Hit
CTCGCTGCGATCTATTGAAAGTCAGCCCTCGACACA	90917	0.479910095705423	No Hit
CTACGGGGATGATTTTACGAACTGAACTCTCTCTTT	88663	0.4680122399059573	No Hit
TGAGGTAGTAGATTGTATAGTT	87391	0.4612979219925055	No Hit
TGAGGTAGTAGTTTGTGCTGTT	86624	0.4572492727475232	No Hit
GCAAATGATGATAAACTGGATCTGACTGACTGTGCT	85283	0.4501707347585775	No Hit
TAGCTTATCAGACTGATGTTGAT	80948	0.4272882126242901	No Hit
CGACTCTTAGCGGTGGATCACTCGGCTCGTGCGTCG	75439	0.39820867065478854	No Hit
TGGAATGTAAAGAAGTATGTAT	75102	0.3964297986918693	No Hit
TAGCTTATCAGACTGATGTTGACA	72369	0.3820035165712216	No Hit
TGCCTCTGATGAAGCCTGTGTTGGTAGGGACATCTG	70704	0.3732147277930005	No Hit
GTGAAATGATGGCAAATCATCTTTCGGGACTGACCT	68822	0.36328049327011036	No Hit
TGGAAGACTAGTGATTTTGTTGT	68097	0.35945354320151557	No Hit
ACTCCATGATGAACACAAAATGACAAGCATATGGCT	66585	0.3514723728515634	No Hit
TAGCTTATCAGACTGATGTTGACT	60248	0.3180221899761357	No Hit
ACAAATGATGAATAACAAAGGGACTTAATACTG	58995	0.31140816454724013	No Hit
TCAGTGCACTACAGAACTTTGT	57605	0.30407097751917567	No Hit
CTGCAGTGATGACTTTCTTAGGACACCTTTGGATTT	56333	0.2973566596057239	No Hit
TACCCTGTAGATCCGAATTTGT	55941	0.2952874672927733	No Hit
CTAGACTGAAGCTCCTTGAGG	55761	0.2943373279653981	No Hit
CGCTGCGATCTATTGAAAGTCAGCCCTCGACACAAG	54653	0.28848869255022147	No Hit
TGAAATGATGGCAAATCATCTTTCGGGACTGACCTG	54200	0.28609750857632704	No Hit
TGAGGTAGTAGGTTGTATAGTT	53992	0.28499956979802676	No Hit
TTCAAGTAATCCAGGATAGGCT	53552	0.28267700699777615	No Hit
CTCACTGATGAGTACGTTCTGACTTTCGTTCTTCTG	53405	0.2819010598804197	No Hit
TTTGAATGATGACTTTAATTGTCGGATACCCCTTCA	52763	0.27851222961278127	No Hit
CAGGACGGTGGCCATGGAAGTCGGAATCCGCTAAGG	52366	0.27641664454073694	No Hit
TTCAAATCGATCTGCGCCTTT	50810	0.26820321791075974	No Hit
TGAGGTAGTAGTTTGTACAGTT	47243	0.24937462357327345	No Hit

Sequence	Count	Percentage	Possible Source
CTGAATGATGATATCCCACTAACTGAGCAGTCAGTA	46013	0.24288200483620917	No Hit
ATTCAAATCGATCTGCGCCTT	44311	0.23389790964069423	No Hit
ATACATGATGATCTCACACAACTTGAACTCTCTCAC	43660	0.2304615724066871	No Hit
TAATACTGCCTGGTAATGATGAC	43280	0.22845572271556155	No Hit
CACAGATGATGAACTTATTGACGGGCGGACAGAAAC	42930	0.22660822957899854	No Hit
ATATATGATGACTTAGCTTTTTTCCCCGAC	42850	0.22618594543349843	No Hit
TCGCGTGATGACATTCTCCGGAATCGCTGTACGGCC	42405	0.22383698987415404	No Hit
TGCTATGATGAAGGCTATGTTGGTAGGGACAACTGA	42332	0.2234516555913852	No Hit
TAGGGTGATGAAAAAGAATCCTTAGGCGTGGTTGTG	41716	0.2202000676710343	No Hit
GCAGCTGATGATACAGCTTCTTTCCCCATC	41647	0.21983584759554045	No Hit
ACCGGGTGCTGTAGGCTT	41479	0.21894905088999023	No Hit
TTCACAGTGGCTAAGTTCTGC	41122	0.21706460789069595	No Hit
TAATACTGCCGGGTAATGATGGA	40768	0.21519600054685797	No Hit
CTTAATGATGACTGTTTTTTTTGATTGCTTGAAGCA	40685	0.2147578807459016	No Hit
TAACACTGTCTGGTAACGATGTT	39475	0.20837083304521237	No Hit
AGCAGCATTGTACAGGGCTATGA	39332	0.20761600013513093	No Hit
AACTGTGATGAAAGATTTGGTCTGTATGTAAT	39076	0.20626469086953056	No Hit
GCATTGGTGGTTCAGTGGTAGAATTCTCGCCT	38568	0.20358318654560484	No Hit
ACCGGGTGCTGTAGGCTTT	37870	0.19989875737611634	No Hit
TTCAAATCGAACTGCGCCTTT	36737	0.193918158165471	No Hit
TTCCTATGATGAGGACCTTTTCACAGACCTGTACTG	36465	0.1924823920707706	No Hit
AGAAATGAAGAAACTAAAATTGGTCTTAGTATTGAA	35921	0.18961085988136983	No Hit
GCATATGATGGAAAAGTTTTAATCTCCTGACACTTG	35272	0.18618507975100015	No Hit
TAGCTTATCAGACTGATGTTG	34905	0.1842478512335184	No Hit
CTGCTGTGATGACATTCCAATTAAAGCACGTGTTAG	34727	0.18330826900978064	No Hit
ATTCAAATCGATCTGCGCCTTA	34444	0.18181443884507398	No Hit
TTCAAATCGATCTGCGCCTTTT	34226	0.18066371454858618	No Hit
TACCCTGTAGATCCGAATTTGTG	34087	0.1799299958457797	No Hit
TAATACTGCCTGGTAATGATGA	33344	0.1760080318444474	No Hit
TACGGGGATGATTTTACGAACTGAACTCTCTCTTTC	33123	0.17484147189250335	No Hit
TAAAGTGCTTATAGTGCAGGTAG	33083	0.1746303298197533	No Hit
TGTAACAGCAACTCCATGTGGA	31860	0.1681746609454203	No Hit
TCAGATGATGAATTTAACTGTTCAACTGCTGAATGA	31837	0.16805325425358902	No Hit
CTGACCTATGAATTGACAGCC	30859	0.16289083057485013	No Hit
TGTAAACATCCCCGACTGGAAG	30821	0.16269024560573758	No Hit
GGCTGGTCCGATGGTAGTGGGTTATCAGAACT	30399	0.16046269673822447	No Hit
AAGCTATGATGAATTTGATTGCATTGATCGTCTGAC	30022	0.15847268270255518	No Hit

Sequence	Count	Percentage	Possible Source
TACAATGATGATAACATAGTTCAGCAGACTAACGCT	29859	0.1576122787560987	No Hit
TGTAATGATGTTGATCAAATGTCTGACCTGAAATGA	29848	0.15755421468609243	No Hit
CTCCATGATGAACACAAAATGACAAGCATATGGCTG	29752	0.1570474737114923	No Hit
TTCAAATCGAACTGCGCCTTTT	28588	0.15090323939446565	No Hit
TAATACTGTCTGGTAAAACCGT	28464	0.15024869896894047	No Hit
AGTCTGTGATGAATTGCTTTGACTTCTGACACCTCG	28154	0.14861234790512753	No Hit
CACCAGTGATGAGTTGAATACCGCCCCAGTCTGATC	28079	0.14821645651872117	No Hit
GCTTAATGATGACTGTTTTTTTTGATTGCTTGAAGC	27571	0.14553495219479545	No Hit
TGTAAACATCCCCGACTGGAAGCT	27227	0.14371913036914497	No Hit
ACGGCCCTGGCGGAGCGCTGAGAAGACGGTCGAACT	26699	0.1409320550088442	No Hit
TGGAATGATGACATTCTCCGGAATCGCTGTACTGAC	26027	0.13738486818664328	No Hit
TGTAAACATCCCCGACTGGAAGC	25934	0.13689396286749939	No Hit
ACCCTGTAGATCCGAATTTGTG	25472	0.13445527192723622	No Hit
CTGCGATGATGGCATTTCTTAGGACACCTTTGGATT	25326	0.13368460336169852	No Hit
TCAAATGATGAAATCACCCAAAATAGCTGGAATTAC	25173	0.13287698493342953	No Hit
ATTCAAATCGAACTGCGCCTTC	24936	0.13162596815238548	No Hit
CTCCTACTTGGATAACTGTGGTAATTCTAGAGCTAA	24528	0.12947231901033487	No Hit
TCTCGTGATGAAAACTCTGTCCAGTTCTGCTACTGA	24249	0.12799960305290323	No Hit
AATACATGATGATCTCAATCCAACTTGAACTCTCTC	23390	0.12346532704059576	No Hit
CGCGACCTCAGATCAGACGC	23171	0.12230932419228921	No Hit
GCATTGGTGGTTCAGTGGTAGAATTCTCGCC	22665	0.11963837697200098	No Hit
TGGAAGACTAGTGATTTTGTTGTC	22591	0.11924776413741336	No Hit
TTGCATGATGACTTGAATTGTCGGATACCCCTTCAC	22579	0.11918442151558836	No Hit
GGTCCAGGATGAAACCTAATTTGAGTGGACATCCAT	22140	0.11686713726715649	No Hit
TTGCTGTGATGACTATCTTAGGACACCTTTGGATTA	22132	0.11682490885260646	No Hit
GTTGAGGTCTATCCCGATGGGGCTTTTCCTGTAGCC	21996	0.11610702580525628	No Hit
ACAGATGATGAACTTATTGACGGGCGGACAGAAACT	21587	0.11394809811138694	No Hit
TTCAAGTAATTCAGGATAGGTT	21454	0.113246050719493	No Hit
TCGTACCGTGAGTAATAATGCG	21231	0.11206893366391144	No Hit
GACTCTTAGCGGTGGATCACTCGGCTCGTGCGTCGA	20746	0.10950883603181699	No Hit
AGCAAATGATGATAAACTGGATCTGACTGACTGTGC	20728	0.10941382209907949	No Hit
CTGACCTATGAATTGACAGCCAT	20617	0.10882790284719805	No Hit
CTGACCTATGAATTGACAGCT	20250	0.10689067432971629	No Hit
GTAGGGTGATGAAAAAGAATCCTTAGGCGTGGTTGT	20147	0.1063469834923849	No Hit
CTTAATGATGAACAATGTAAATGACAAGCATATGGC	20143	0.10632586928510988	No Hit
TGTTTGTGATGACTTACATGGAATCTCGTTCGGCTG	19794	0.10448365470036565	No Hit
TGCATATGATGGAAAAGTTTTAATCTCCTGACACTT	19794	0.10448365470036565	No Hit

Sequence	Count	Percentage	Possible Source
TGCTGTGATGAGATGACTAAGTAGGAAGTGCCGTCA	19718	0.10408248476214055	No Hit
TAACACTGTCTGGTAACGATGT	19708	0.10402969924395303	No Hit
GATGGGAGACCGCCTGGGAATACCGGGTGCTGTAGG	19589	0.10340155157752161	No Hit
TGAGATGAAGCACTGTAGCTCT	19529	0.10308483846839653	No Hit
TAGCTTATCAGACTGATGTTGAA	19474	0.10279451811836521	No Hit
CGCGACCTCAGATCAGACGTGGCGACCCGCTGAATT	19297	0.10186021444644619	No Hit
TTCACAGTGGCTAAGTTCCG	19093	0.10078338987542089	No Hit
TTTGCATGATGACTTGAATTGTCGGATACCCCTTCA	18973	0.10014996365717073	No Hit

Adapter Content

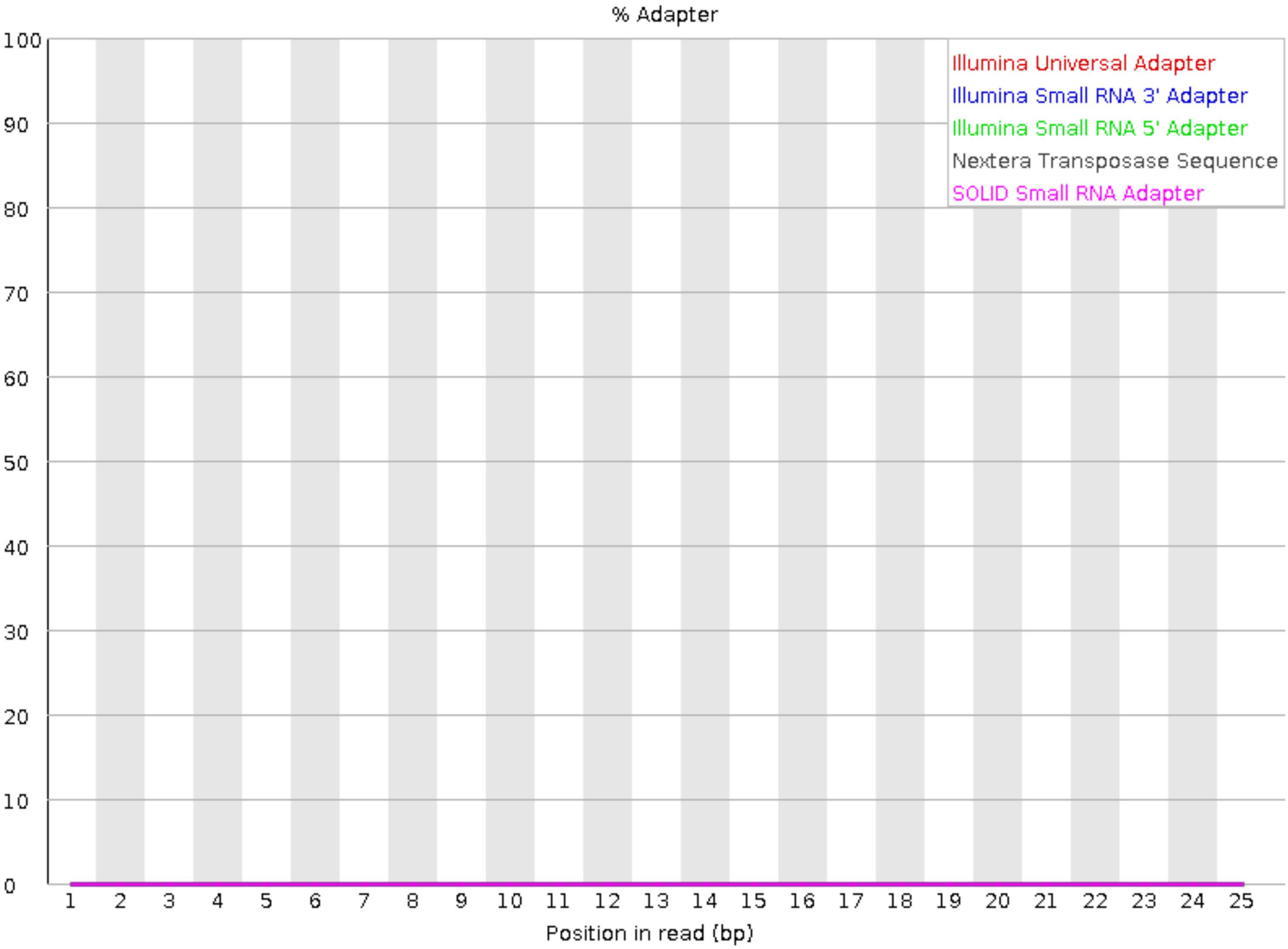

Supplement: Supplementary file 5 [file DataSheet5.zip › QC reports/shCD44_4.fastq.gz FastQC Report.pdf]
