# Supplementary material for: CD44 knockdown alters miRNA expression and their target genes in colon cancer
Source: Front Immunol. 2025 May 14;16:1552665. doi: 10.3389/fimmu.2025.1552665 (PMC12116639; doi:10.3389/fimmu.2025.1552665)
Supplement: Supplementary file 5 [file DataSheet5.zip › QC reports/shCD44_8.fastq.gz FastQC Report.pdf]

FastQC Report

Mon 31 Mar 2025
shCD44_8.fastq.gz

Summary

- Basic Statistics
- Per base sequence quality
- Per tile sequence quality
- Per sequence quality scores
- Per base sequence content
- Per sequence GC content
- Per base N content
- Sequence Length Distribution
- Sequence Duplication Levels
- Overrepresented sequences
- Adapter Content

Basic Statistics

Measure	Value
Filename	shCD44_8.fastq.gz
File type	Conventional base calls
Encoding	Sanger / Illumina 1.9
Total Sequences	21517273
Sequences flagged as poor quality	0
Sequence length	18-36
%GC	47

Per base sequence quality

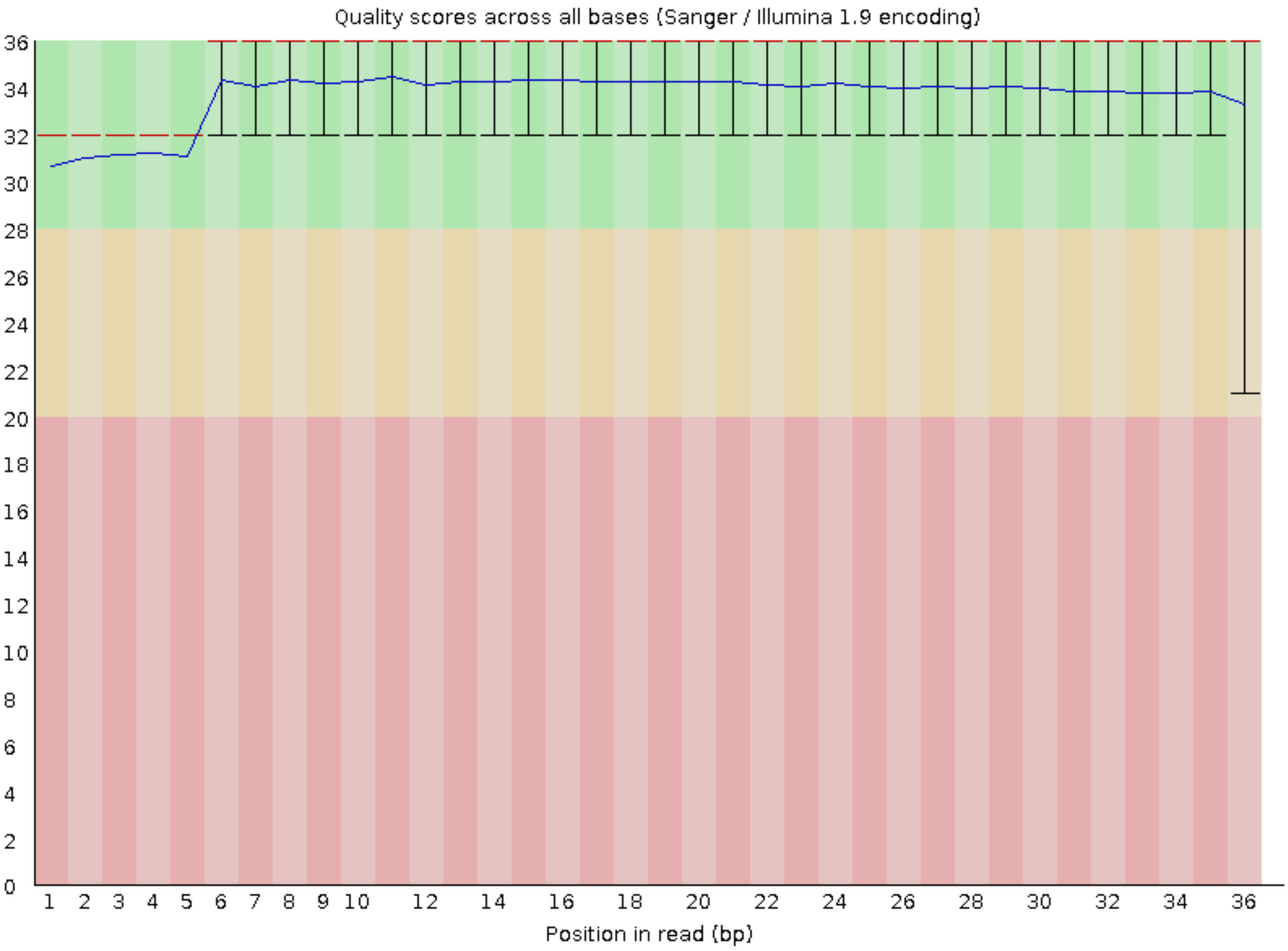

Per tile sequence quality

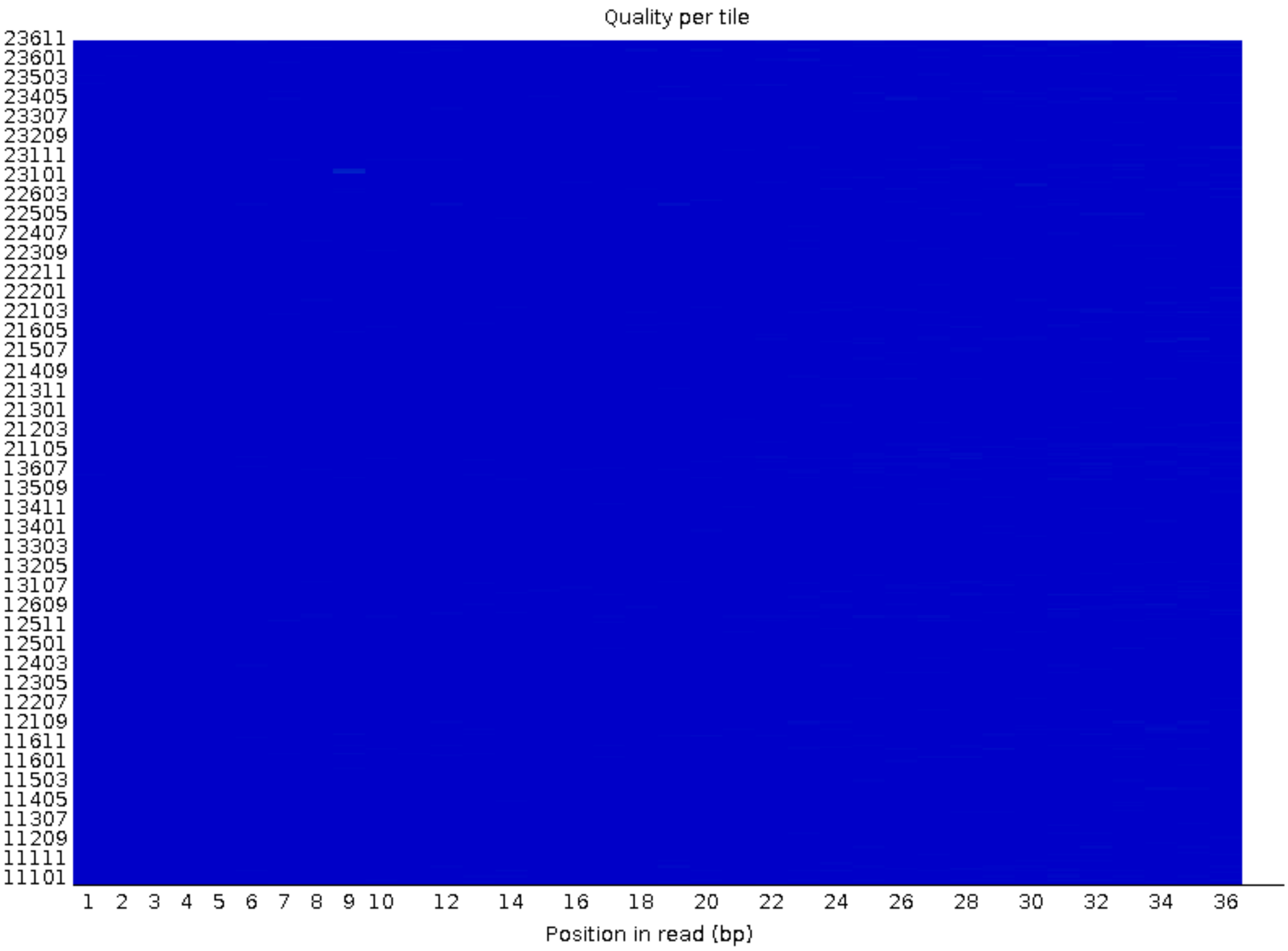

Per sequence quality scores

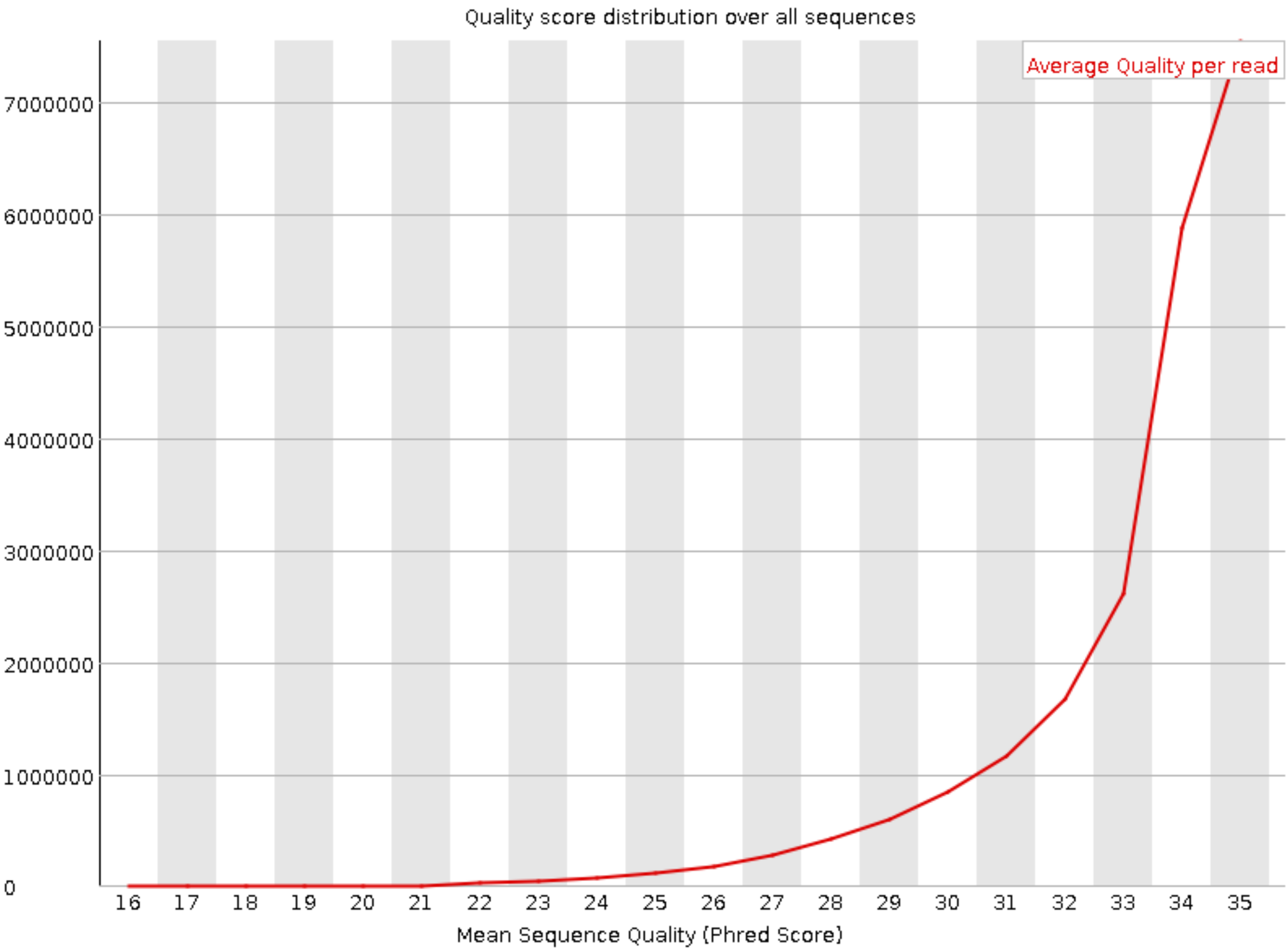

Per base sequence content

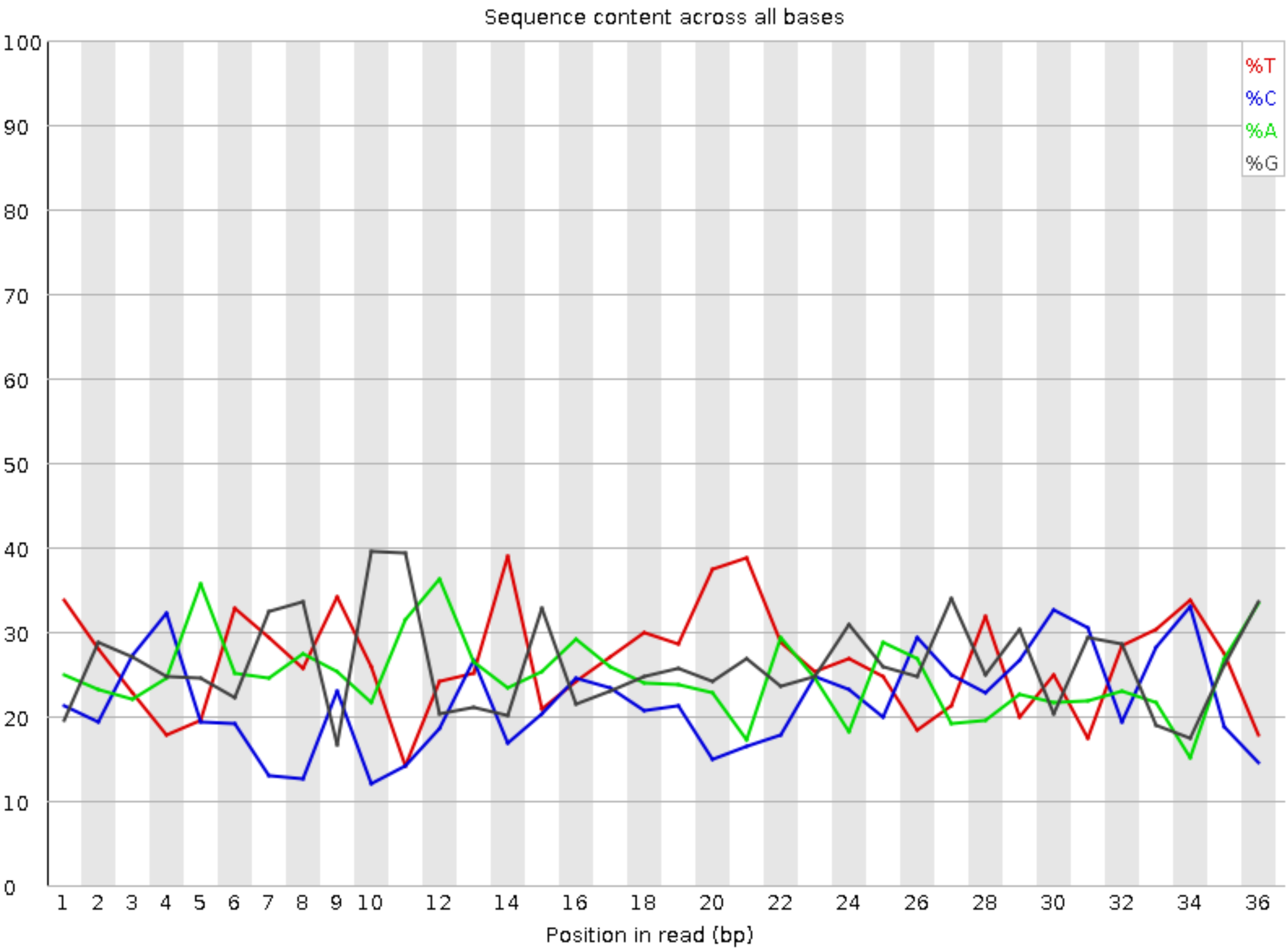

Per sequence GC content

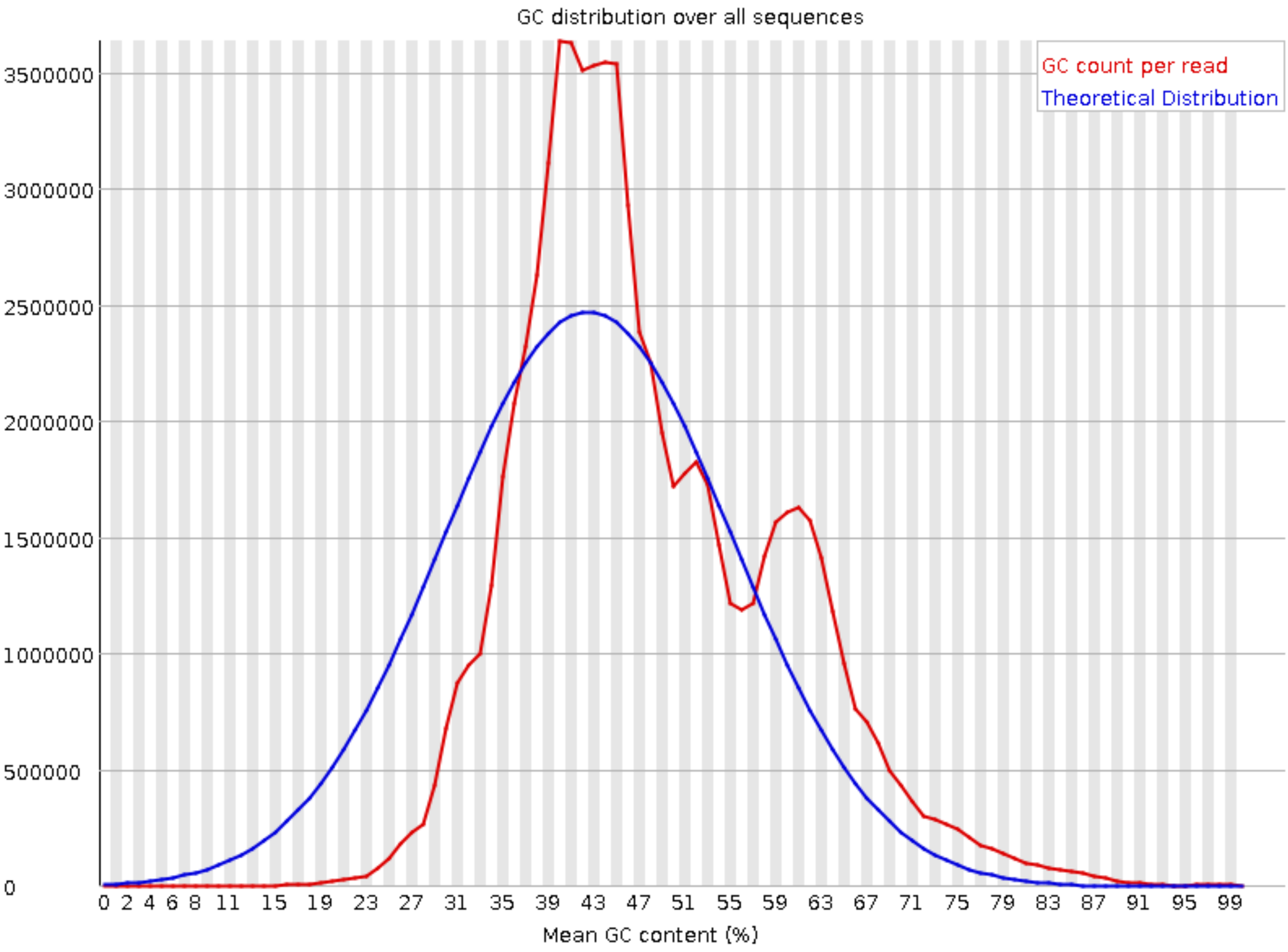

Per base N content

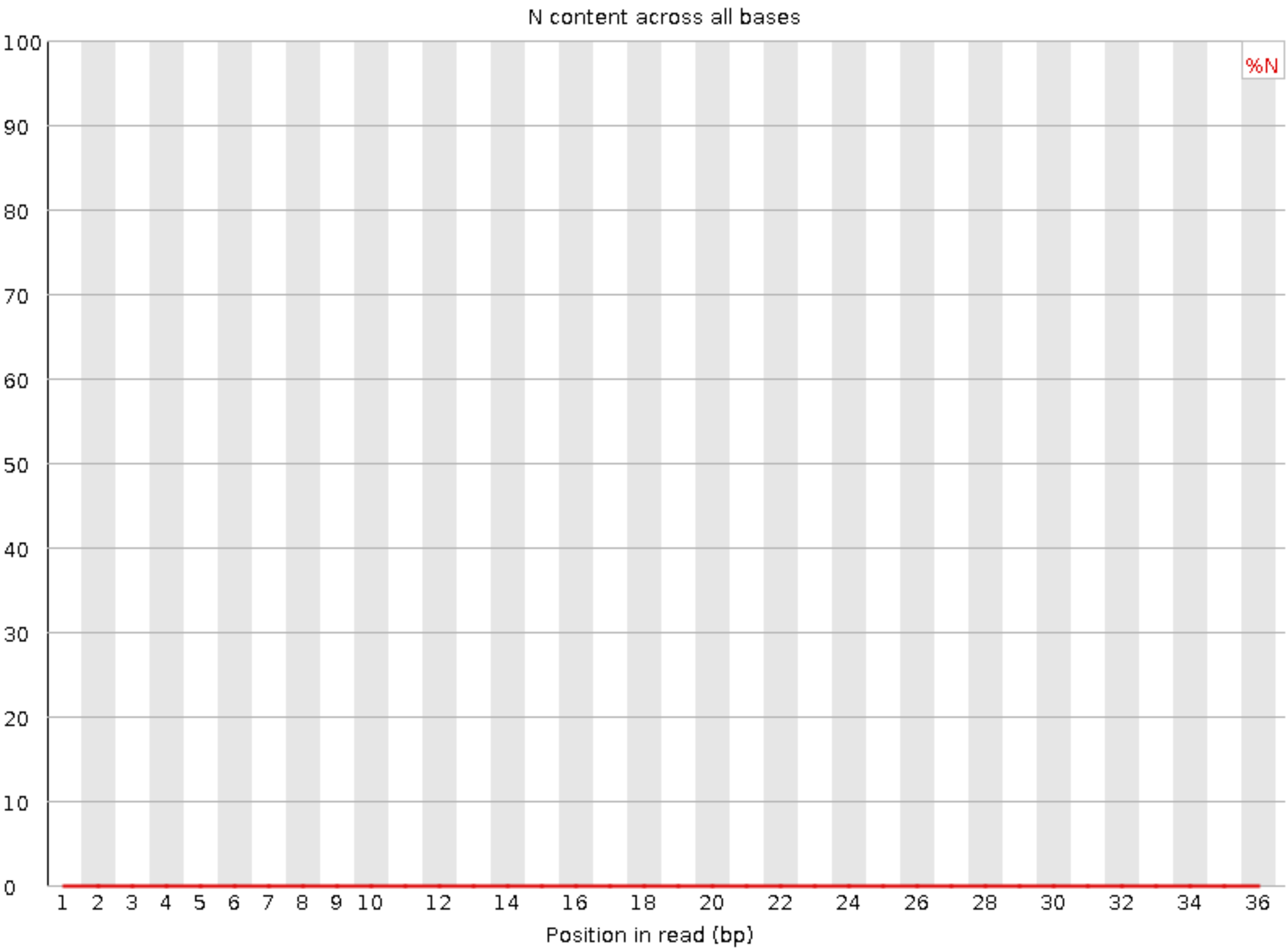

Sequence Length Distribution

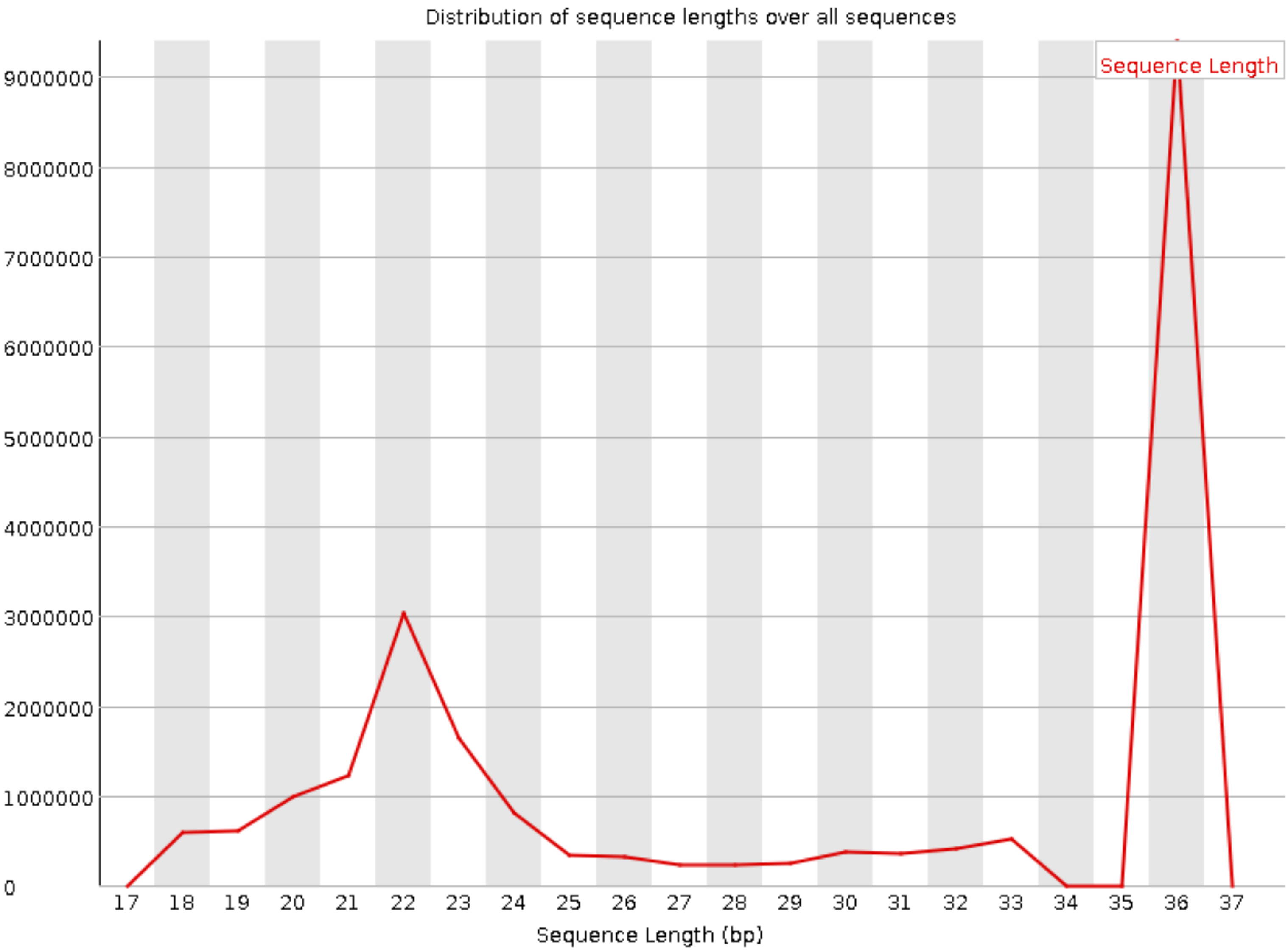

Sequence Duplication Levels

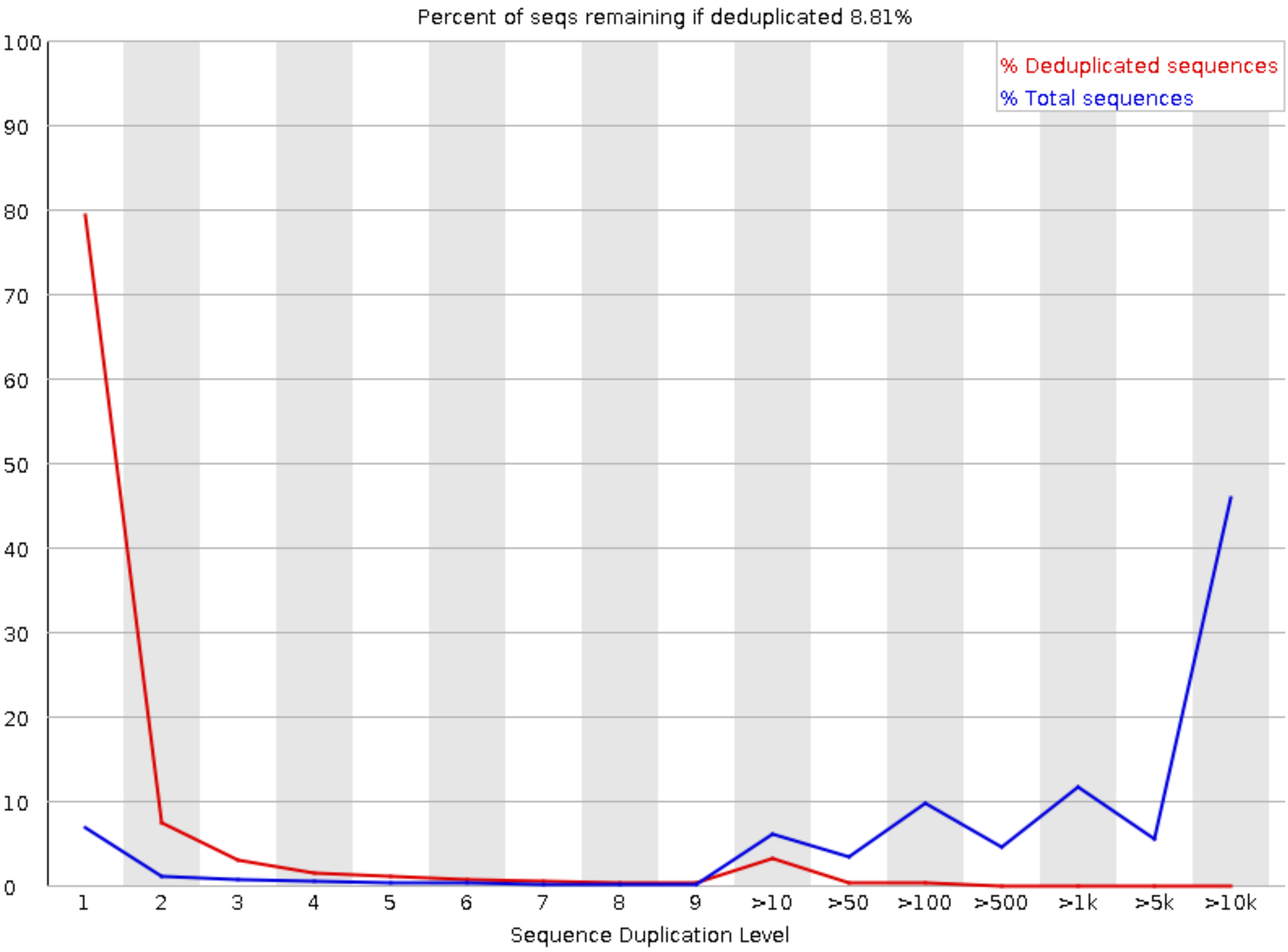

Overrepresented sequences

Sequence	Count	Percentage	Possible Source
TGCTCTGATGAAATCACTAATAGGAAGTGCCGTCAG	599659	2.7868726673682116	No Hit
ATTCAAATCGATCTGCGCCTTT	476887	2.2162985058562024	No Hit
TAGCTTATCAGACTGATGTTGAC	326935	1.5194072222813737	No Hit
GTGAAATGATGGCAATCATCTTTCGGGACTGACCTG	281403	1.307800481966279	No Hit
CGCGACCTCAGATCAGACGT	256889	1.193873405798216	No Hit
GTTTGTGATGACTTACATGGAATCTCGTTCGGCTGA	239901	1.1149228807944203	No Hit
CCTGGATGATGATAAGCAAATGCTGACTGAACATGA	226054	1.0505699304925862	No Hit
TAGCTTATCAGACTGATGTTGA	214880	0.9986395580889827	No Hit
AGTAGTGATGAAATTCCACTTCATTGGTCCGTGTTT	205953	0.9571519588007272	No Hit
GCCTCTGATGAAGCCTGTGTTGGTAGGGACATCTGA	192904	0.8965076568949978	No Hit
TATCTGTGATGATCTTATCCCGAACCTGAACTTCTG	176767	0.8215120940279004	No Hit
GTGCAATGATGTATTTTATTCAACACATCATTCTGA	163428	0.7595200376925088	No Hit

Sequence	Count	Percentage	Possible Source
ATTCAAATCGAACTGCGCCTTT	140372	0.6523689131052991	No Hit
TTGAATGATGACTTTAATTGTCGGATACCCCTTCAC	138829	0.6451979300536829	No Hit
ATACATGATGATCTCAATCCAACTTGAACTCTCTCA	131812	0.6125869202849265	No Hit
TTTCTATGATGAATCAAACTAGCTCACTATGACCGA	128974	0.5993975165905084	No Hit
CGACTCTTAGCGGTGGATCACTCGGCTCGTGCGTCG	128406	0.5967577768799978	No Hit
TGGAAGACTAGTGATTTTGTTGTT	123038	0.5718103776440444	No Hit
TCGCTGCGATCTATTGAAAGTCAGCCCTCGACACAA	116718	0.5424386259355449	No Hit
TGCCTCTGATGAAGCCTGTGTTGGTAGGGACATCTG	93603	0.43501330303333513	No Hit
ACCGGGTGCTGTAGGCTT	92895	0.4317229232533323	No Hit
TGAGGTAGTAGATTGTATAGTT	92654	0.4306028928479924	No Hit
CTACGGGGATGATTTTACGAACTGAACTCTCTCTTT	83386	0.3875305202476169	No Hit
TAGCTTATCAGACTGATGTTGAT	80159	0.3725332666458245	No Hit
GCAAATGATGATAAACTGGATCTGACTGACTGTGCT	79876	0.37121804421963694	No Hit
ATTCAAATCGATCTGCGCCTTC	76298	0.3545895430150465	No Hit
ACCGGGTGCTGTAGGCTTT	75845	0.3524842576473329	No Hit
GAGAAGACGGTCGAACTTGACTATCT	70078	0.32568253421332716	No Hit
CTGGATGATGATAAGCAAATGCTGACTGAACATGAA	68834	0.31990113245298324	No Hit
CTCGCTGCGATCTATTGAAAGTCAGCCCTCGACACA	68810	0.31978959415535607	No Hit
TGAAATGATGGCAATCATCTTTCGGGACTGACCTGA	68809	0.3197849467262882	No Hit
TAGCTTATCAGACTGATGTTGACA	67943	0.3157602731535729	No Hit
CGCTGCGATCTATTGAAAGTCAGCCCTCGACACAAG	67141	0.3120330350411969	No Hit
CTCCTACTTGGATAACTGTGGTAATTCTAGAGCTAA	65100	0.302547632313816	No Hit
TTTGAATGATGACTTTAATTGTCGGATACCCCTTCA	61210	0.28446913324007184	No Hit
TGAGGTAGTAGTTTGTGCTGTT	60929	0.28316320567201986	No Hit
CTGCAGTGATGACTTTCTTAGGACACCTTTGGATTT	59257	0.2753927042706573	No Hit
TGAGGTAGTAGGTTGTATAGTT	57886	0.2690210790187028	No Hit
ACAAATGATGAATAACAAAGGGACTTAATACTG	55866	0.2596332723017457	No Hit
CTAGACTGAAGCTCCTTGAGG	55177	0.2564311936740311	No Hit
ACTCCATGATGAACACAAAATGACAAGCATATGGCT	55157	0.25633824509267505	No Hit
CAGGACGGTGGCCATGGAAGTCGGAATCCGCTAAGG	53291	0.24766614245215926	No Hit
TACCCTGTAGATCCGAATTTGT	52908	0.24588617711919164	No Hit
GATGGGAGACCGCCTGGGAATACCGGGTGCTGTAGG	52717	0.24499851816724172	No Hit
TAGCTTATCAGACTGATGTTGACT	50742	0.23581984575833564	No Hit
CACAGATGATGAACTTATTGACGGGCGGACAGAAAC	50627	0.2352853914155386	No Hit
TTCAAGTAATCCAGGATAGGCT	50380	0.23413747643579183	No Hit
TAATACTGCCGGGTAATGATGGA	48369	0.22479149658044492	No Hit
TGGAAGACTAGTGATTTTGTTGT	48296	0.22445223425849548	No Hit

Sequence	Count	Percentage	Possible Source
CTGAATGATGATATCCCACTAACTGAGCAGTCAGTA	47457	0.22055304127061082	No Hit
TAACACTGTCTGGTAACGATGTT	46337	0.21534792071467423	No Hit
AATGGATTTTTGGAGCAGG	46286	0.21511090183221637	No Hit
GCAGCTGATGATACAGCTTCTTTCCCCATC	43741	0.20328319485466398	No Hit
CTCACTGATGAGTACGTTCTGACTTTCGTTCTTCTG	43727	0.20321813084771476	No Hit
AGAAGACGGTCGAACTTGACTATCT	43405	0.201721658687883	No Hit
TGAGGTAGTAGTTTGTACAGTT	42433	0.19720435763398084	No Hit
AACTGTGATGAAAGATTTGGTCTGTATGTAAT	41056	0.19080484780761947	No Hit
CGCGACCTCAGATCAGACGTGGCGACCCGCTGAATT	40953	0.19032616261363602	No Hit
CGCGACCTCAGATCAGACGC	40438	0.18793273664371876	No Hit
TAATACTGCCTGGTAATGATGAC	39656	0.18429844711269872	No Hit
ATTCAAATCGATCTGCGCCTT	39571	0.18390341564193566	No Hit
TCTCCTACTTGGATAACTGTGGTAATTCTAGAGCTA	39268	0.1824952446343921	No Hit
TTCAAATCGATCTGCGCCTTT	39234	0.18233723204608687	No Hit
TGGGAGACCGCCTGGGAATACCGGGTGCTGTAGGCT	39098	0.181705181692866	No Hit
TAGCTTATCAGACTGATGTTG	37690	0.17516160156540284	No Hit
ATGACCTATGAATTGACAGACA	36428	0.16929654608183853	No Hit
GTGAAATGATGGCAAATCATCTTTCGGGACTGACCT	36259	0.16851113056938022	No Hit
TAATACTGTCTGGTAAAACCGT	36199	0.1682322848253122	No Hit
GCATTGGTGGTTCAGTGGTAGAATTCTCGCCT	35057	0.16292492082988397	No Hit
TAGGGTGATGAAAAAGAATCCTTAGGCGTGGTTGTG	34736	0.16143309609912	No Hit
TGTAACAGCAACTCCATGTGGA	34290	0.15936034273488095	No Hit
ATATATGATGACTTAGCTTTTTTCCCCGAC	34186	0.1588770101118297	No Hit
CTGACCTATGAATTGACAGCC	33875	0.15743165967174372	No Hit
TCAGTGCACTACAGAACTTTGT	33758	0.15688791047081105	No Hit
TTCCTATGATGAGGACCTTTTCACAGACCTGTACTG	33744	0.15682284646386185	No Hit
CTTAATGATGACTGTTTTTTTTGATTGCTTGAAGCA	33638	0.156330218982675	No Hit
CGACTCTTAGCGGTGGATCACTCGGCTCGTG	33624	0.1562651549757258	No Hit
CTGCTGTGATGACATTCCAATTAAAGCACGTGTTAG	33219	0.15438294620326656	No Hit
AGCAGCATTGTACAGGGCTATGA	32320	0.15020490747131385	No Hit
TTCAAATCGAACTGCGCCTTT	32265	0.14994929887258482	No Hit
GGCTGGTCCGATGGTAGTGGGTTATCAGAACT	31578	0.14675651510300586	No Hit
TCGCGTGATGACATTCTCCGGAATCGCTGTACGGCC	30692	0.14263889294893456	No Hit
TAATACTGCCTGGTAATGATGA	30030	0.1395622949060506	No Hit
TTCAAATCGATCTGCGCCTTTT	29967	0.13926950687477915	No Hit
GCATATGATGGAAAAGTTTTAATCTCCTGACACTTG	29922	0.13906037256672812	No Hit
GACTCTTAGCGGTGGATCACTCGGCTCGTGCGTCGA	29573	0.13743841982206573	No Hit

Sequence	Count	Percentage	Possible Source
TACAATGATGATAACATAGTTCAGCAGACTAACGCT	29537	0.13727111237562492	No Hit
TTCACAGTGGCTAAGTTCTGC	29448	0.13685749118859067	No Hit
TGTAAACATCCCCGACTGGAAG	28614	0.132981535346045	No Hit
TTCAAATCGAACTGCGCCTTTT	28553	0.13269804217290918	No Hit
CTGACCTATGAATTGACAGCCAT	27414	0.12740462046468434	No Hit
TGTAAACATCCCCGACTGGAAGC	27347	0.12709324271714173	No Hit
TACCCTGTAGATCCGAATTTGTG	27316	0.1269491724160399	No Hit
AATACATGATGATCTCAATCCAACTTGAACTCTCTC	27208	0.12644725007671742	No Hit
TCCTACTTGGATAACTGTGGTAATTCTAGAGCTAAT	26902	0.12502513678197047	No Hit
TCGCGAAGGCCCGCGGCGGGTGTTGACGCGATGTGA	26864	0.12484853447739404	No Hit
TCGTACGACTCTTAGCGGTGGATCACTCGGCTCGTG	26760	0.12436520185434279	No Hit
AGAAATGAAGAAACTAAAATTGGTCTTAGTATTGAA	26486	0.12309180628976545	No Hit
TGTAAACATCCCCGACTGGAAGCT	26407	0.1227246593934092	No Hit
AGTCTGTGATGAATTGCTTTGACTTCTGACACCTCG	26065	0.1211352386522214	No Hit
CTGACCTATGAATTGACAGCT	25864	0.1202011054095935	No Hit
GCTTAATGATGACTGTTTTTTTTGATTGCTTGAAGC	25780	0.11981072136789823	No Hit
TCAGATGATGAATTTAACTGTTCAACTGCTGAATGA	25488	0.11845367208010048	No Hit
AAGCTATGATGAATTTGATTGCATTGATCGTCTGAC	25262	0.11740335311077757	No Hit
CTGCGATGATGGCATTTCTTAGGACACCTTTGGATT	24766	0.11509822829314849	No Hit
AGACGTGGCGACCCGCTGAATTT	24145	0.11221217484204435	No Hit
ATTCAAATCGATCTGCGCCTTA	24095	0.1119798033886543	No Hit
TACGGGGATGATTTTACGAACTGAACTCTCTCTTTC	23799	0.11060416438458534	No Hit
TGAAATGATGGCAAATCATCTTTCGGGACTGACCTG	23729	0.11027884434983931	No Hit
GTAGGGTGATGAAAAAGAATCCTTAGGCGTGGTTGT	23324	0.10839663557738008	No Hit
AGCAAATGATGATAAACTGGATCTGACTGACTGTGC	22893	0.10639359364915804	No Hit
ACCCTGTAGATCCGAATTTGTG	22340	0.10382356537466433	No Hit
GACGTGGCGACCCGCTGAATTT	22245	0.10338205961322329	No Hit
TCAAATGATGAAATCACCCAAAATAGCTGGAATTAC	22140	0.10289407956110425	No Hit
TAACACTGTCTGGTAACGATGT	22124	0.10281972069601943	No Hit
ATGGATTTTTGGAGCAGG	21736	0.10101651821771282	No Hit

Adapter Content

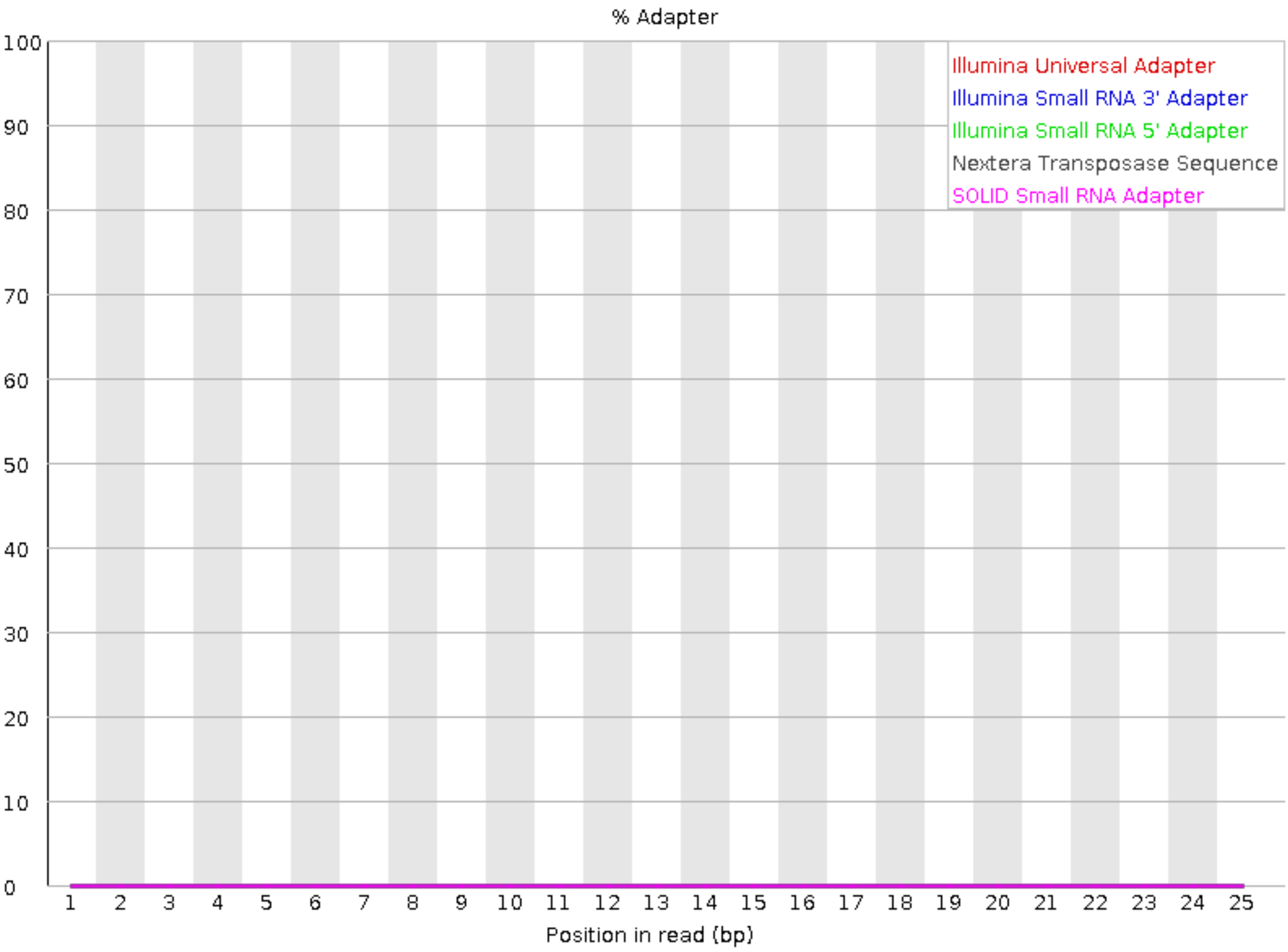

Produced by [FastQC](http://www.bioinformatics.babraham.ac.uk/projects/fastqc/) (version 0.11.9)
