# Supplementary material for: CD44 knockdown alters miRNA expression and their target genes in colon cancer
Source: Front Immunol. 2025 May 14;16:1552665. doi: 10.3389/fimmu.2025.1552665 (PMC12116639; doi:10.3389/fimmu.2025.1552665)

FastQC Report

Mon 31 Mar 2025
shLUC_5.fastq.gz

Summary

- Basic Statistics
- Per base sequence quality
- Per tile sequence quality
- Per sequence quality scores
- Per base sequence content
- Per sequence GC content
- Per base N content
- Sequence Length Distribution
- Sequence Duplication Levels
- Overrepresented sequences
- Adapter Content

Basic Statistics

Measure	Value
Filename	shLUC_5.fastq.gz
File type	Conventional base calls
Encoding	Sanger / Illumina 1.9
Total Sequences	14316749
Sequences flagged as poor quality	0
Sequence length	18-36
%GC	50

Per base sequence quality

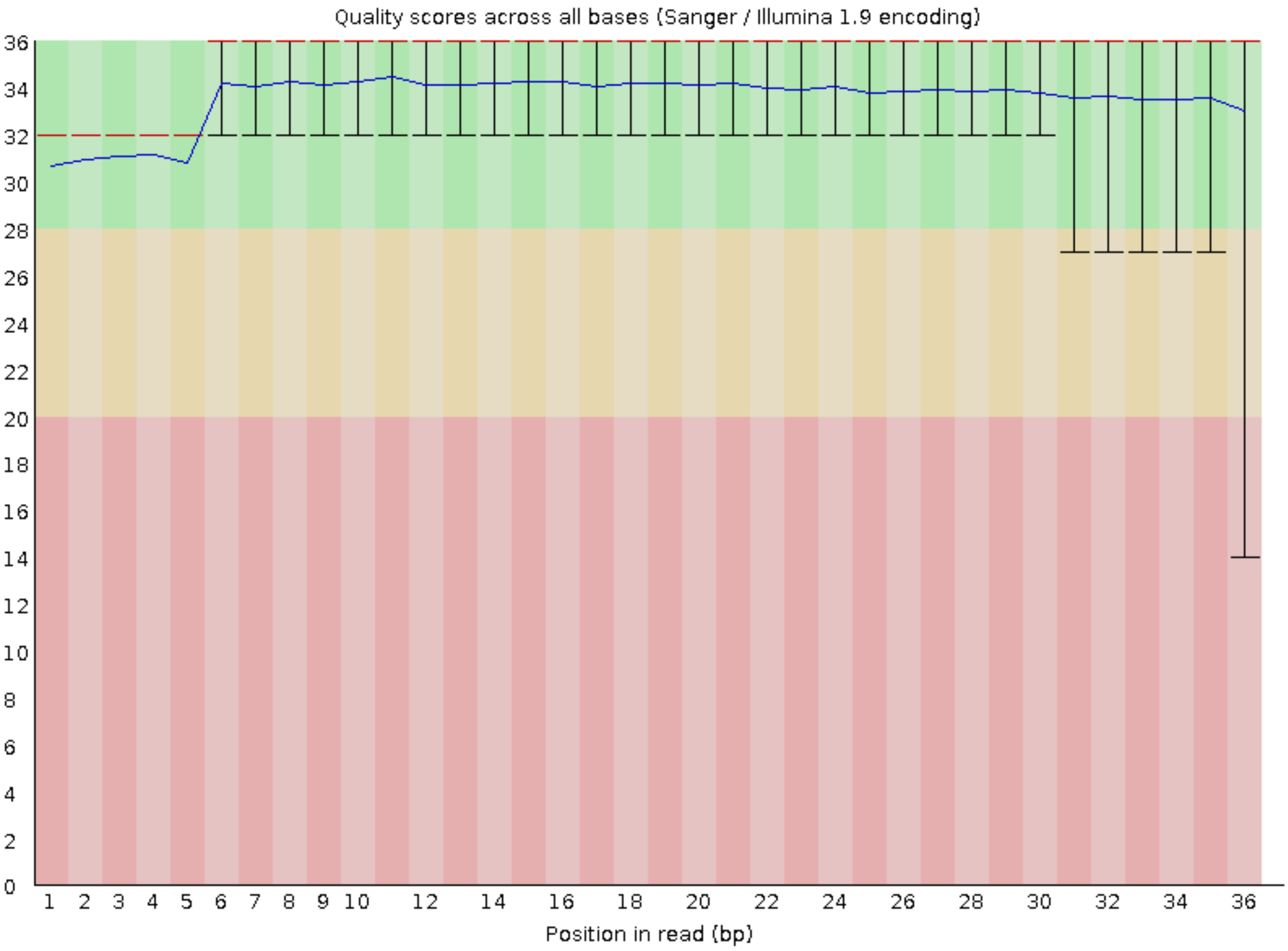

Per tile sequence quality

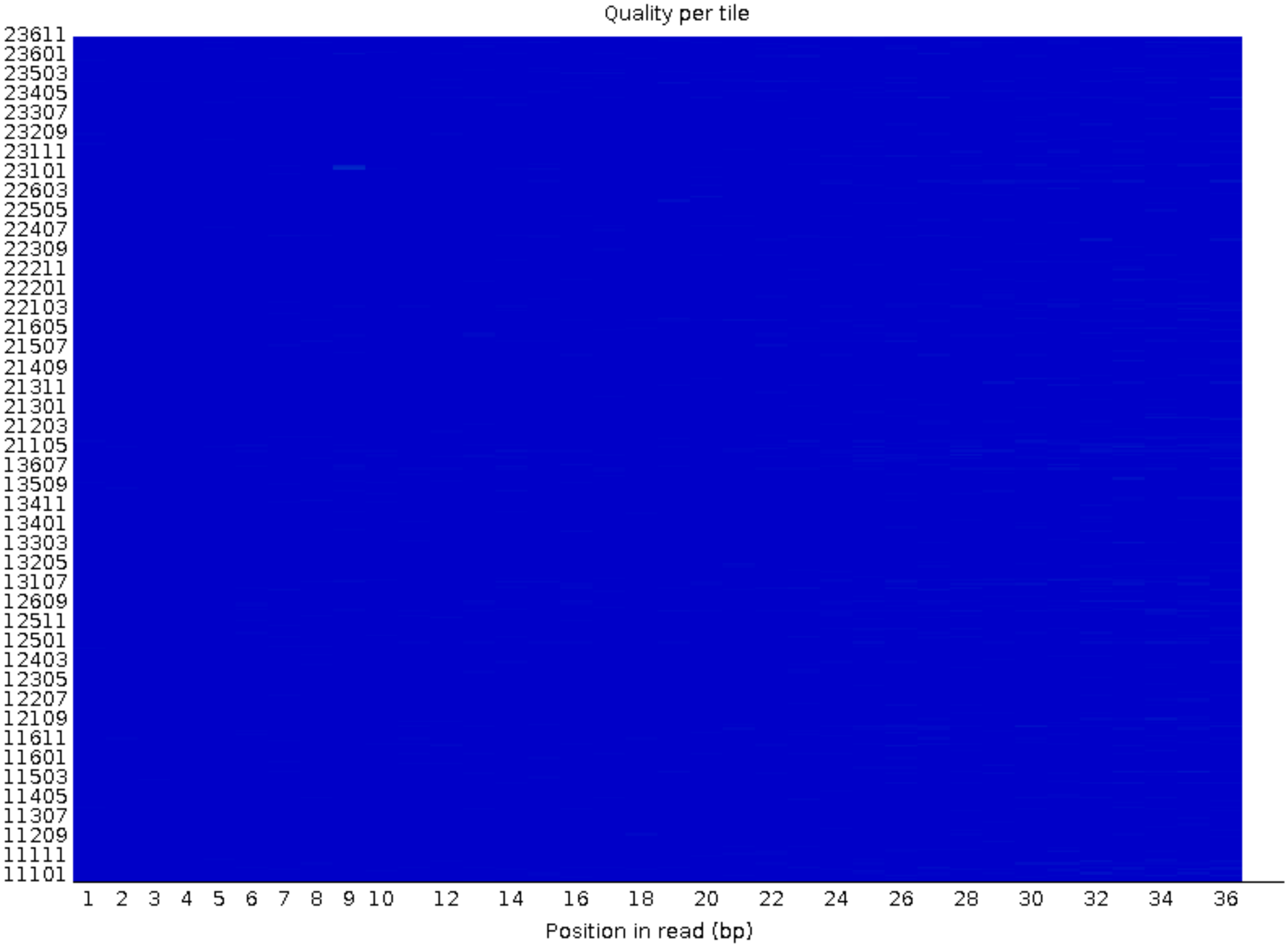

Per sequence quality scores

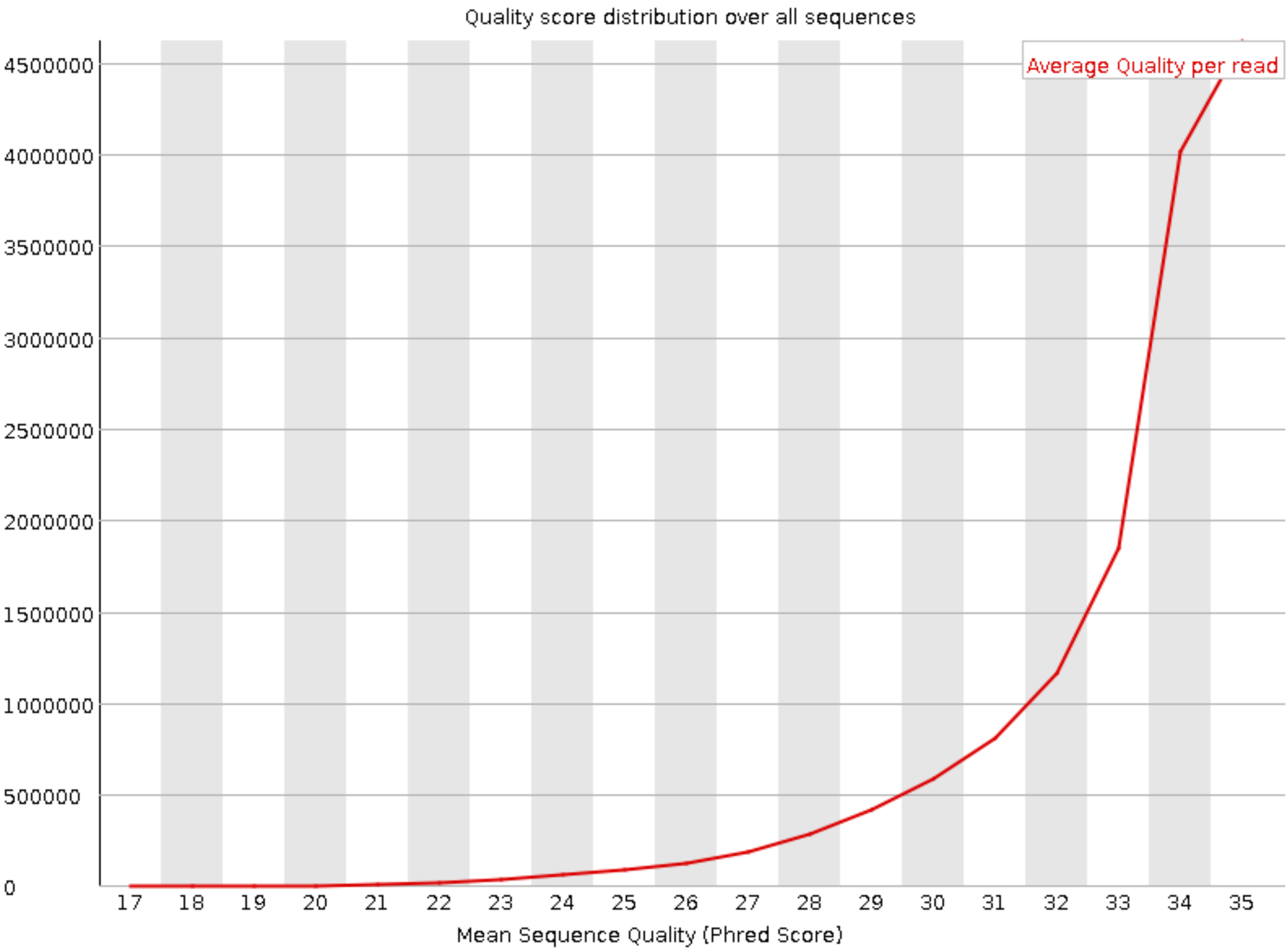

Per base sequence content

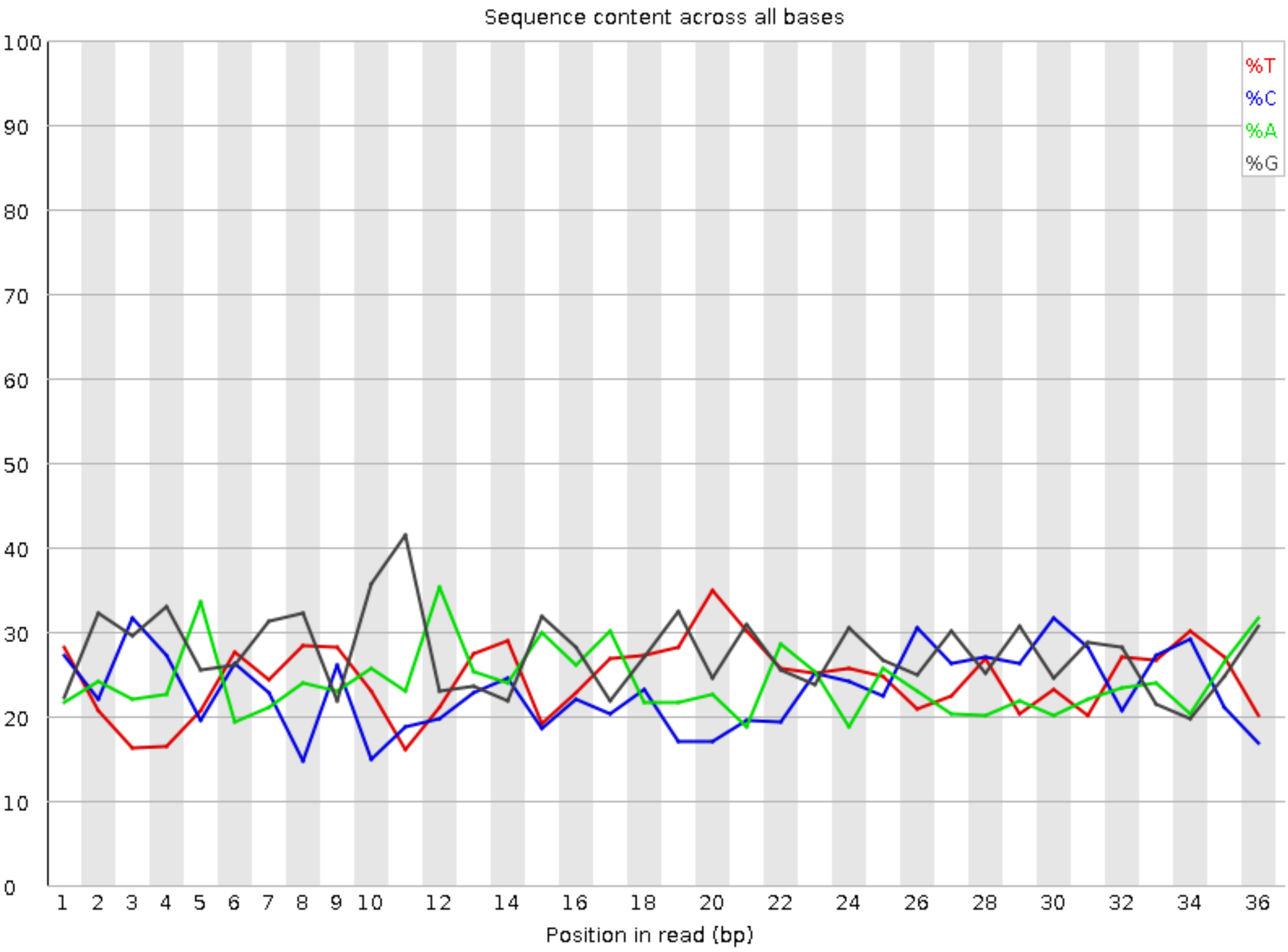

Per sequence GC content

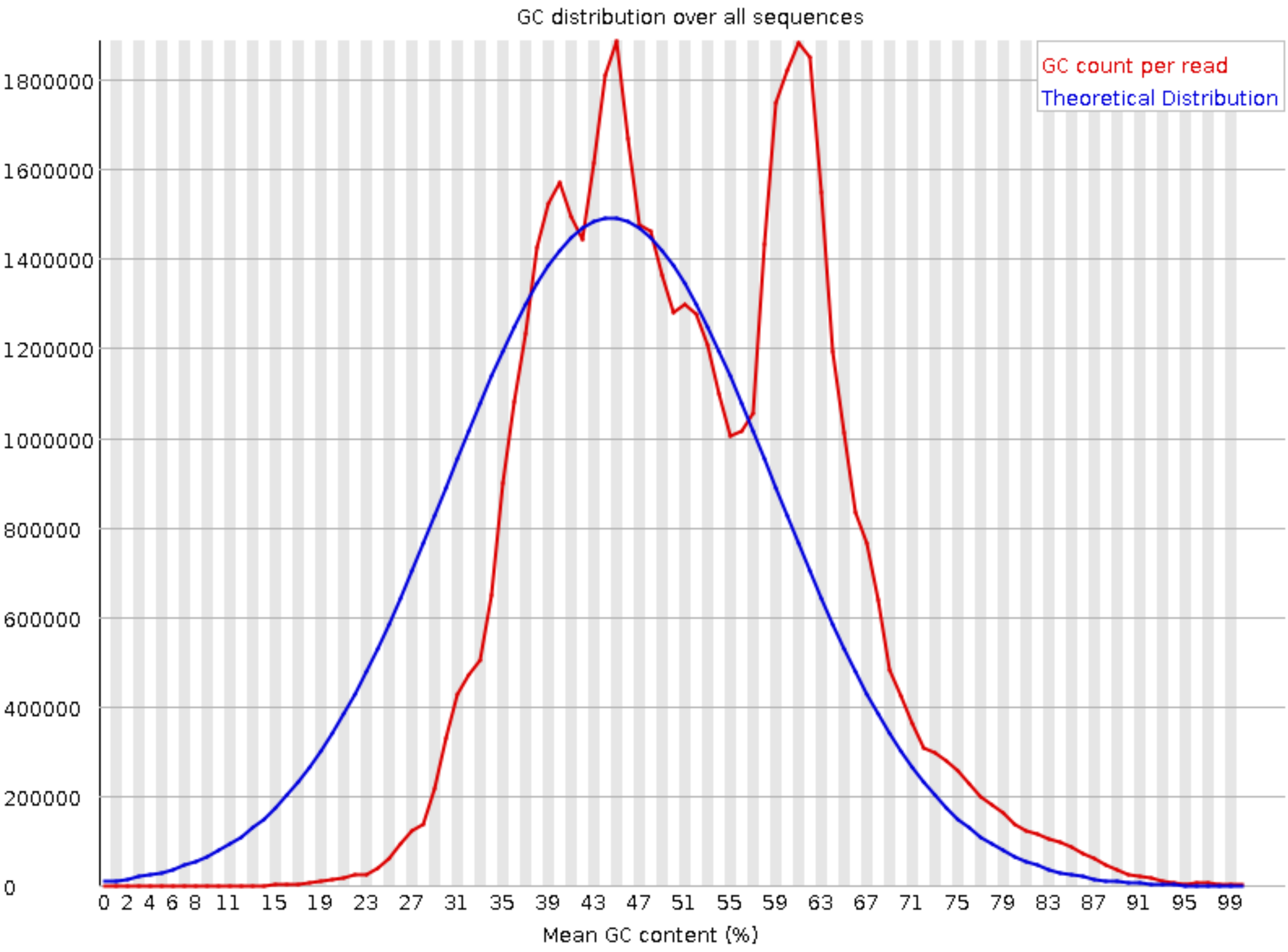

Per base N content

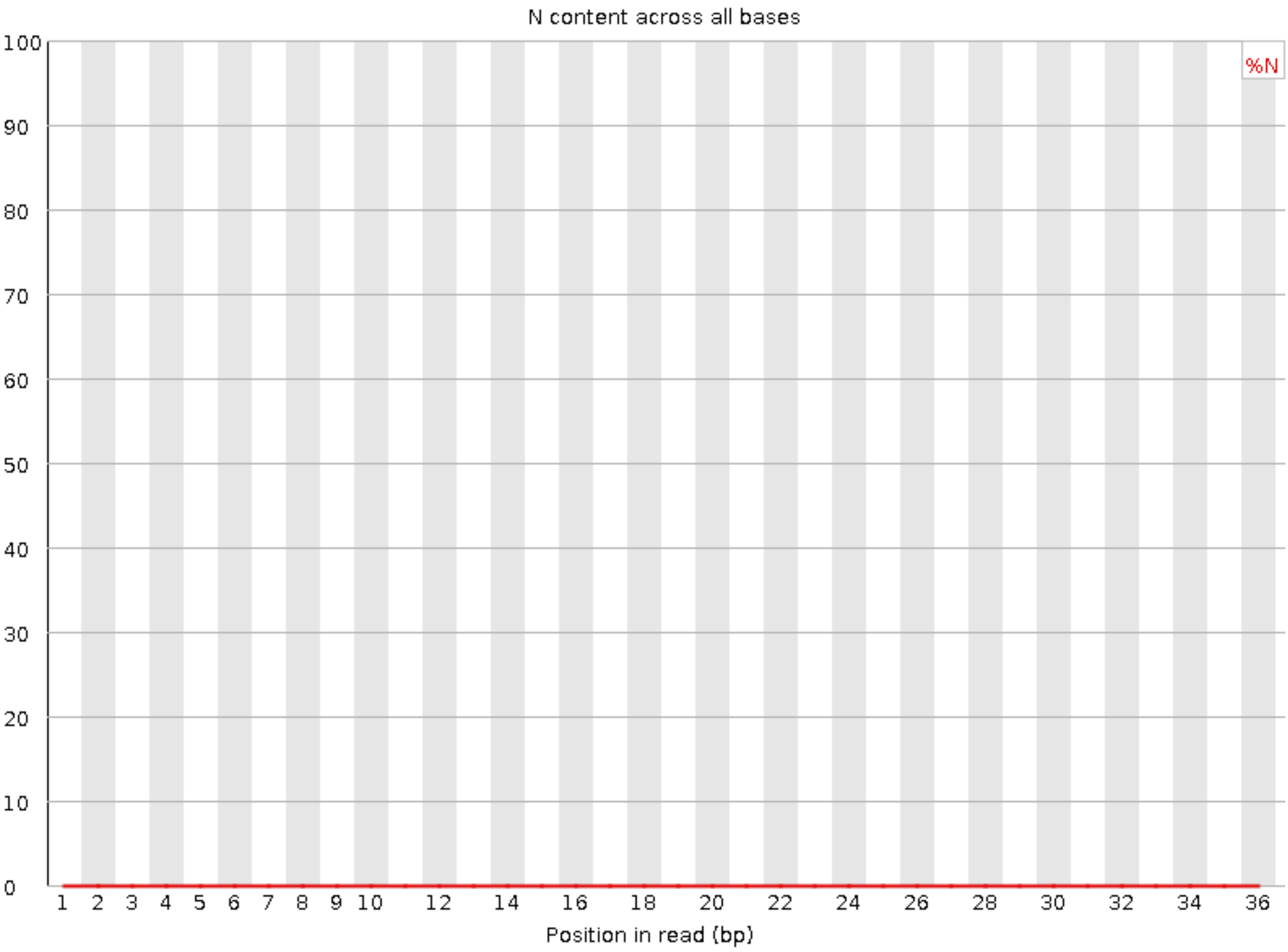

Sequence Length Distribution

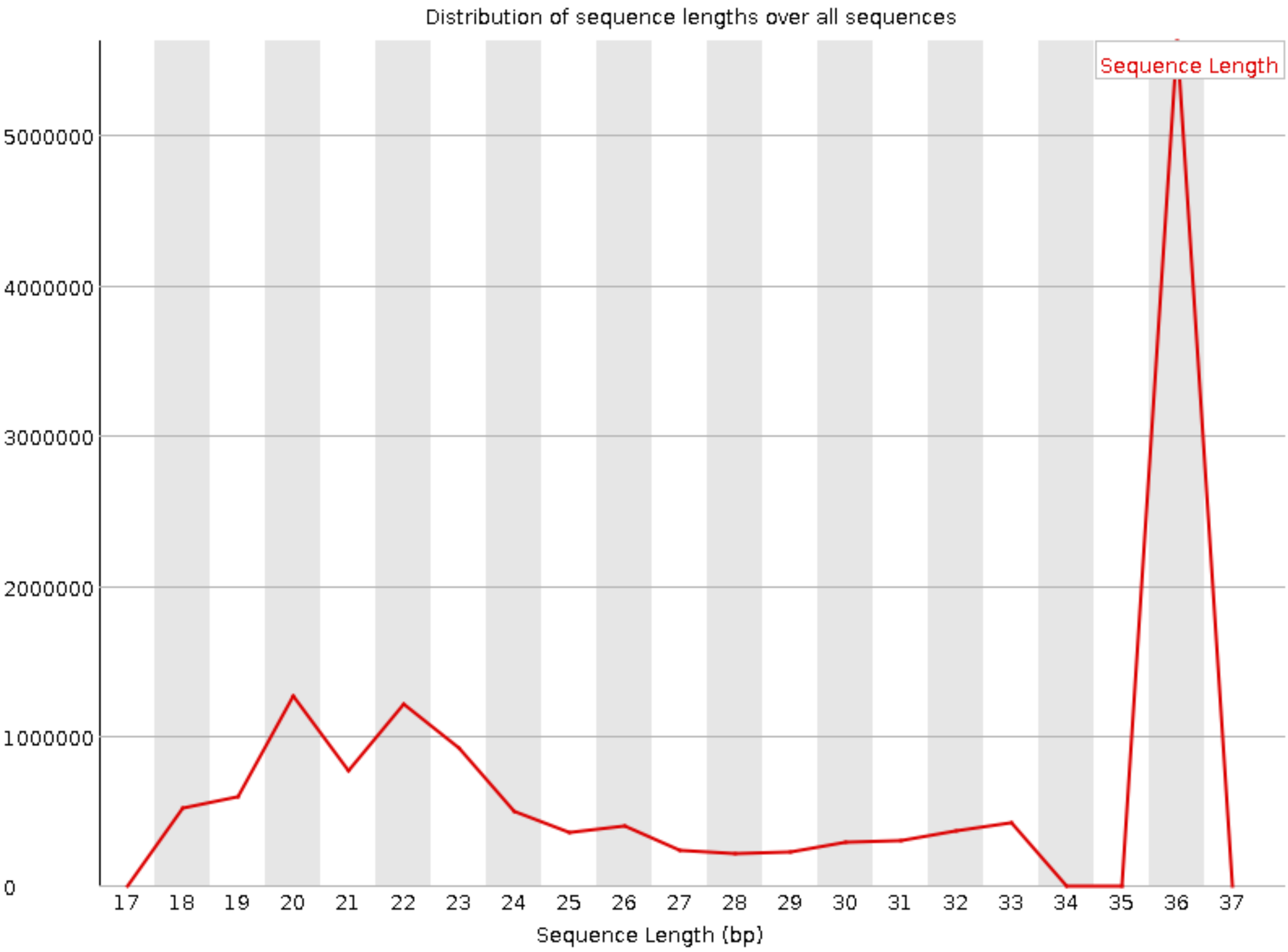

Sequence Duplication Levels

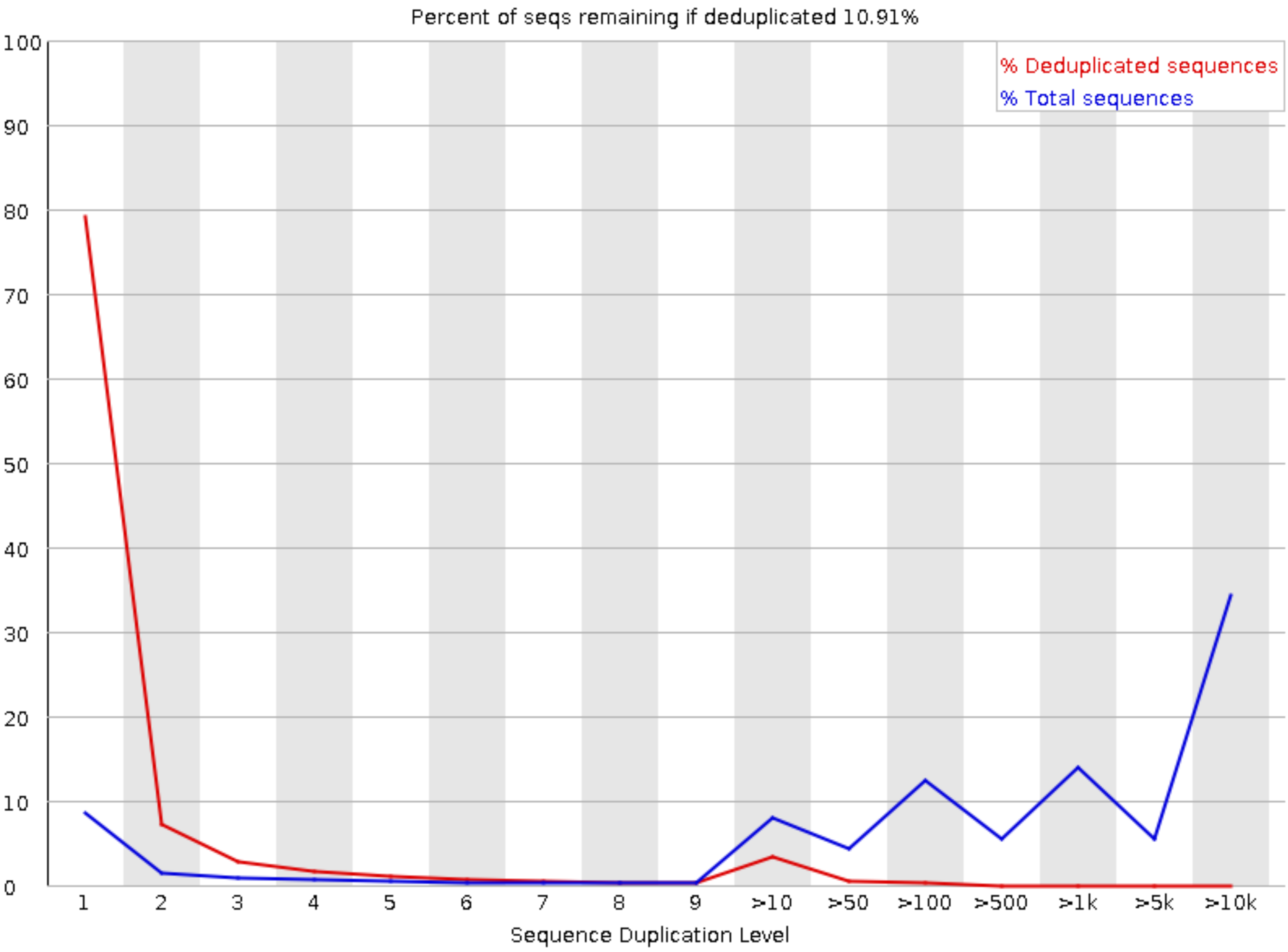

Overrepresented sequences

Sequence	Count	Percentage	Possible Source
CGCGACCTCAGATCAGACGT	578220	4.038766063440799	No Hit
TAGCTTATCAGACTGATGTTGAC	200710	1.4019244173380423	No Hit
TGCTCTGATGAAATCACTAATAGGAAGTGCCGTCAG	166114	1.1602773786143767	No Hit
TAGCTTATCAGACTGATGTTGA	135558	0.9468490367470994	No Hit
GAGAAGACGGTCGAACTTGACTATCT	130186	0.9093265517192485	No Hit
GTGAAATGATGGCAATCATCTTTCGGGACTGACCTG	123199	0.8605235727747969	No Hit
GTTTGTGATGACTTACATGGAATCTCGTTCGGCTGA	104912	0.7327920605439125	No Hit
CGCGACCTCAGATCAGACGC	91071	0.6361150845069645	No Hit
CGCGACCTCAGATCAGACGTGGCGACCCGCTGAATT	85192	0.5950512927201559	No Hit
ACCGGGTGCTGTAGGCTT	84383	0.5894005685229238	No Hit
CGACTCTTAGCGGTGGATCACTCGGCTCGTGCGTCG	79899	0.5580806089427146	No Hit
GCCTCTGATGAAGCCTGTGTTGGTAGGGACATCTGA	78207	0.5462622834276134	No Hit

Sequence	Count	Percentage	Possible Source
TCGCTGCGATCTATTGAAAGTCAGCCCTCGACACAA	77748	0.5430562483144742	No Hit
AGTAGTGATGAAATTCCACTTCATTGGTCCGTGTTT	76473	0.534150595222421	No Hit
TTGAATGATGACTTTAATTGTCGGATACCCCTTCAC	76418	0.5337664297949206	No Hit
AGAAGACGGTCGAACTTGACTATCT	73199	0.5112822750472191	No Hit
ACCGGGTGCTGTAGGCTTT	68890	0.4811846600090566	No Hit
CGCGACCTCAGATCAGACG	66816	0.46669813097931656	No Hit
CCTGGATGATGATAAGCAAATGCTGACTGAACATGA	64116	0.4478391009020274	No Hit
GTGCAATGATGTATTTTATTCAACACATCATTCTGA	61301	0.42817681583996475	No Hit
ATACATGATGATCTCAATCCAACTTGAACTCTCTCA	58678	0.4098556173611761	No Hit
TTGGTACTAGCAACGCACTTT	57715	0.4031292299669429	No Hit
TATCTGTGATGATCTTATCCCGAACCTGAACTTCTG	56064	0.3915972823159783	No Hit
GATGGGAGACCGCCTGGGAATACCGGGTGCTGTAGG	55492	0.3876019618699748	No Hit
TGGAAGACTAGTGATTTTGTTGTT	54496	0.38064507521924146	No Hit
CGCTGCGATCTATTGAAAGTCAGCCCTCGACACAAG	52965	0.369951306682823	No Hit
CTCCTACTTGGATAACTGTGGTAATTCTAGAGCTAA	51969	0.3629944200320897	No Hit
TTTCTATGATGAATCAAACTAGCTCACTATGACCGA	46667	0.32596087282105735	No Hit
TGAGGTAGTAGATTGTATAGTT	45424	0.31727873415954977	No Hit
TGAAATGATGGCAATCATCTTTCGGGACTGACCTGA	43484	0.30372817180771977	No Hit
CAGGACGGTGGCCATGGAAGTCGGAATCCGCTAAGG	41871	0.2924616475430281	No Hit
CTACGGGGATGATTTTACGAACTGAACTCTCTCTTT	41256	0.2881659795809789	No Hit
GCATTGGTGGTTCAGTGGTAGAATTCTCGCCT	40993	0.2863289703549318	No Hit
TAGCTTATCAGACTGATGTTGAT	39447	0.2755304294291951	No Hit
TGGGAGACCGCCTGGGAATACCGGGTGCTGTAGGCT	37643	0.26292980340718414	No Hit
TGAGGTAGTAGTTTGTGCTGTT	37357	0.2609321431841824	No Hit
TTTGAATGATGACTTTAATTGTCGGATACCCCTTCA	34054	0.2378612630562986	No Hit
TCTCCTACTTGGATAACTGTGGTAATTCTAGAGCTA	34047	0.23781236927461674	No Hit
CTCGCTGCGATCTATTGAAAGTCAGCCCTCGACACA	33249	0.23223847816288462	No Hit
TTCAAGTAATCCAGGATAGGCT	31855	0.22250163078223975	No Hit
ACAAATGATGAATAACAAAGGGACTTAATACTG	31298	0.21861108272555452	No Hit
ACGGCCCTGGCGGAGCGCTGAGAAGACGGTCGAACT	30892	0.21577524338800658	No Hit
GCAAATGATGATAAACTGGATCTGACTGACTGTGCT	30810	0.21520248765973335	No Hit
TAGCTTATCAGACTGATGTTG	30565	0.2134912053008682	No Hit
TAGCTTATCAGACTGATGTTGACT	28762	0.2008975641048118	No Hit
TAACACTGTCTGGTAACGATGTT	27868	0.1946531297014427	No Hit
ACTCCATGATGAACACAAAATGACAAGCATATGGCT	27736	0.19373113267544187	No Hit
TACCCTGTAGATCCGAATTTGT	27452	0.19174744210434927	No Hit
TAGCTTATCAGACTGATGTTGACA	25955	0.18129115765038556	No Hit

Sequence	Count	Percentage	Possible Source
TGCCTCTGATGAAGCCTGTGTTGGTAGGGACATCTG	25666	0.17927254294952016	No Hit
CGCGACCTCAGATCAGACGA	25291	0.17665323321656334	No Hit
AATGGATTTTTGGAGCAGG	24901	0.17392915109428825	No Hit
GCAGCTGATGATACAGCTTCTTTCCCCATC	24843	0.17352403118892423	No Hit
CTGGATGATGATAAGCAAATGCTGACTGAACATGAA	24253	0.16940298387573882	No Hit
TCCTACTTGGATAACTGTGGTAATTCTAGAGCTAAT	24157	0.1687324405841019	No Hit
TCAGTGCACTACAGAACTTTGT	24061	0.16806189729246493	No Hit
CGCGACCTCAGATCAGACGG	23678	0.16538670895187169	No Hit
CTGCAGTGATGACTTTCTTAGGACACCTTTGGATTT	23466	0.16390592584950675	No Hit
GAGAAGACGGTCGAACTTGACTATCC	23429	0.16364748728918835	No Hit
CTAGACTGAAGCTCCTTGAGG	22525	0.15733320462627373	No Hit
TTGGTACTAGCAACGCACTTTT	22135	0.15460912250399864	No Hit
CTGAATGATGATATCCCACTAACTGAGCAGTCAGTA	22123	0.15452530459254402	No Hit
AGCGCTGAGAAGACGGTCGAACTTGACTATCT	21986	0.15356838343677046	No Hit
TCGCGAAGGCCCGCGGCGGGTGTTGACGCGATGTGA	21949	0.15330994487645205	No Hit
GTGAAATGATGGCAAATCATCTTTCGGGACTGACCT	21296	0.1487488535281299	No Hit
GACTCTTAGCGGTGGATCACTCGGCTCGTGCGTCGA	20955	0.14636702787762781	No Hit
CACAGATGATGAACTTATTGACGGGCGGACAGAAAC	20858	0.1456894997600363	No Hit
CTCACTGATGAGTACGTTCTGACTTTCGTTCTTCTG	20726	0.14476750273403552	No Hit
GCCGCCGGTGAAATACCACTACTCTGATCGTTTTTT	20652	0.1442506256133987	No Hit
CGGCCCTGGCGGAGCGCTGAGAAGACGGTCGAACTT	20370	0.14228090469421514	No Hit
ATATATGATGACTTAGCTTTTTTCCCCGAC	19160	0.13382926528920777	No Hit
TAATACTGCCTGGTAATGATGAC	18386	0.12842301000038486	No Hit
GCATTGGTGGTTCAGTGGTAGAATTCTCGCC	18348	0.1281575866141119	No Hit
TGAGGTAGTAGGTTGTATAGTT	18316	0.12793407218356626	No Hit
CTGACCTATGAATTGACAGCC	18180	0.12698413585374724	No Hit
TGAGGTAGTAGTTTGTACAGTT	18149	0.12676760624915615	No Hit
TAATACTGCCGGGTAATGATGGA	17966	0.12548938309947322	No Hit
TAGGGTGATGAAAAAGAATCCTTAGGCGTGGTTGTG	17964	0.1254754134475641	No Hit
TGAAATGATGGCAAATCATCTTTCGGGACTGACCTG	17718	0.12375714626274442	No Hit
GGCTGGTCCGATGGTAGTGGGTTATCAGAACT	17516	0.12234621141992501	No Hit
GGGAGACCGCCTGGGAATACCGGGTGCTGTAGGCTT	16838	0.11761049942273906	No Hit
CGCGACCTCAGATCAGACA	16129	0.112658257820962	No Hit
GCAGCCGACTTAGAACTGGTGCGGACCAGGGGAATC	16025	0.11193183592168865	No Hit
TCGTACGACTCTTAGCGGTGGATCACTCGGCTCGTG	15982	0.11163148840564294	No Hit
TGGAAGACTAGTGATTTTGTTGT	15921	0.1112054140224153	No Hit
TCCCTGGTGGTCTAGTGGTTAGGATTCGGCGCT	15765	0.11011578117350523	No Hit

Sequence	Count	Percentage	Possible Source
TACAATGATGATAACATAGTTCAGCAGACTAACGCT	15176	0.10600171868627437	No Hit
TAATACTGTCTGGTAAAACCGT	14989	0.10469555623277324	No Hit
TTCCTATGATGAGGACCTTTTCACAGACCTGTACTG	14840	0.10365481716554505	No Hit
CGACTCTTAGCGGTGGATCACTCGGCTCG	14840	0.10365481716554505	No Hit
AACTGTGATGAAAGATTTGGTCTGTATGTAAT	14438	0.10084691713181533	No Hit
GACGTGGCGACCCGCTGAATTT	14375	0.10040687309667859	No Hit
ATACATGATGATCTCACACAACTTGAACTCTCTCAC	14372	0.10038591861881493	No Hit
CTTAATGATGACTGTTTTTTTTGATTGCTTGAAGCA	14326	0.10006461662490555	No Hit

Adapter Content

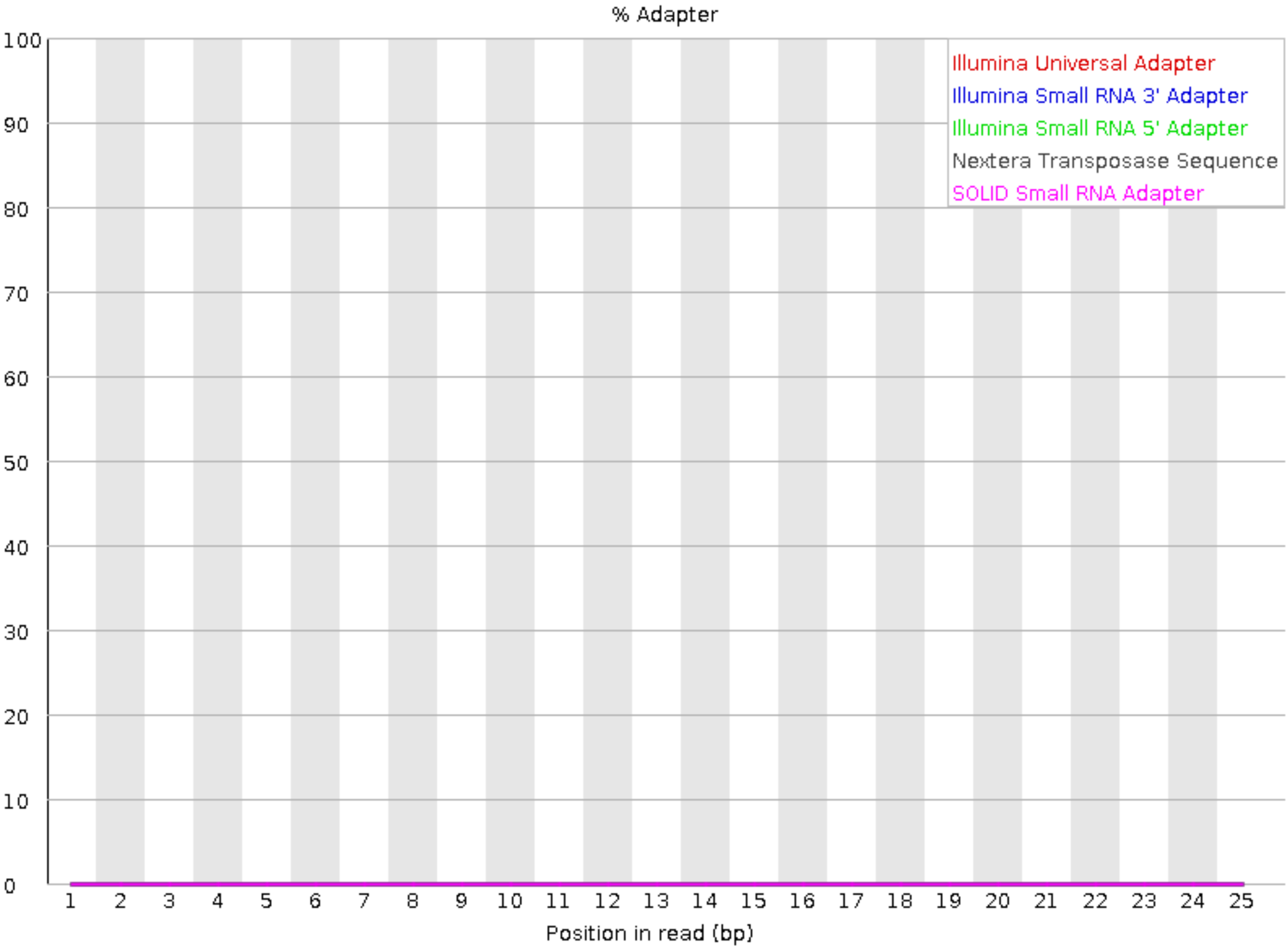

Supplement: Supplementary file 5 [file DataSheet5.zip › QC reports/shLUC_5.fastq.gz FastQC Report.pdf]
