# Supplementary material for: CD44 knockdown alters miRNA expression and their target genes in colon cancer
Source: Front Immunol. 2025 May 14;16:1552665. doi: 10.3389/fimmu.2025.1552665 (PMC12116639; doi:10.3389/fimmu.2025.1552665)
Supplement: Supplementary file 5 [file DataSheet5.zip › QC reports/shCD44_1.fastq.gz FastQC Report.pdf]

FastQC Report

Mon 31 Mar 2025
shCD44_1.fastq.gz

Summary

- Basic Statistics
- Per base sequence quality
- Per tile sequence quality
- Per sequence quality scores
- Per base sequence content
- Per sequence GC content
- Per base N content
- Sequence Length Distribution
- Sequence Duplication Levels
- Overrepresented sequences
- Adapter Content

Basic Statistics

Measure	Value
Filename	shCD44_1.fastq.gz
File type	Conventional base calls
Encoding	Sanger / Illumina 1.9
Total Sequences	15995560
Sequences flagged as poor quality	0
Sequence length	18-36
%GC	47

Per base sequence quality

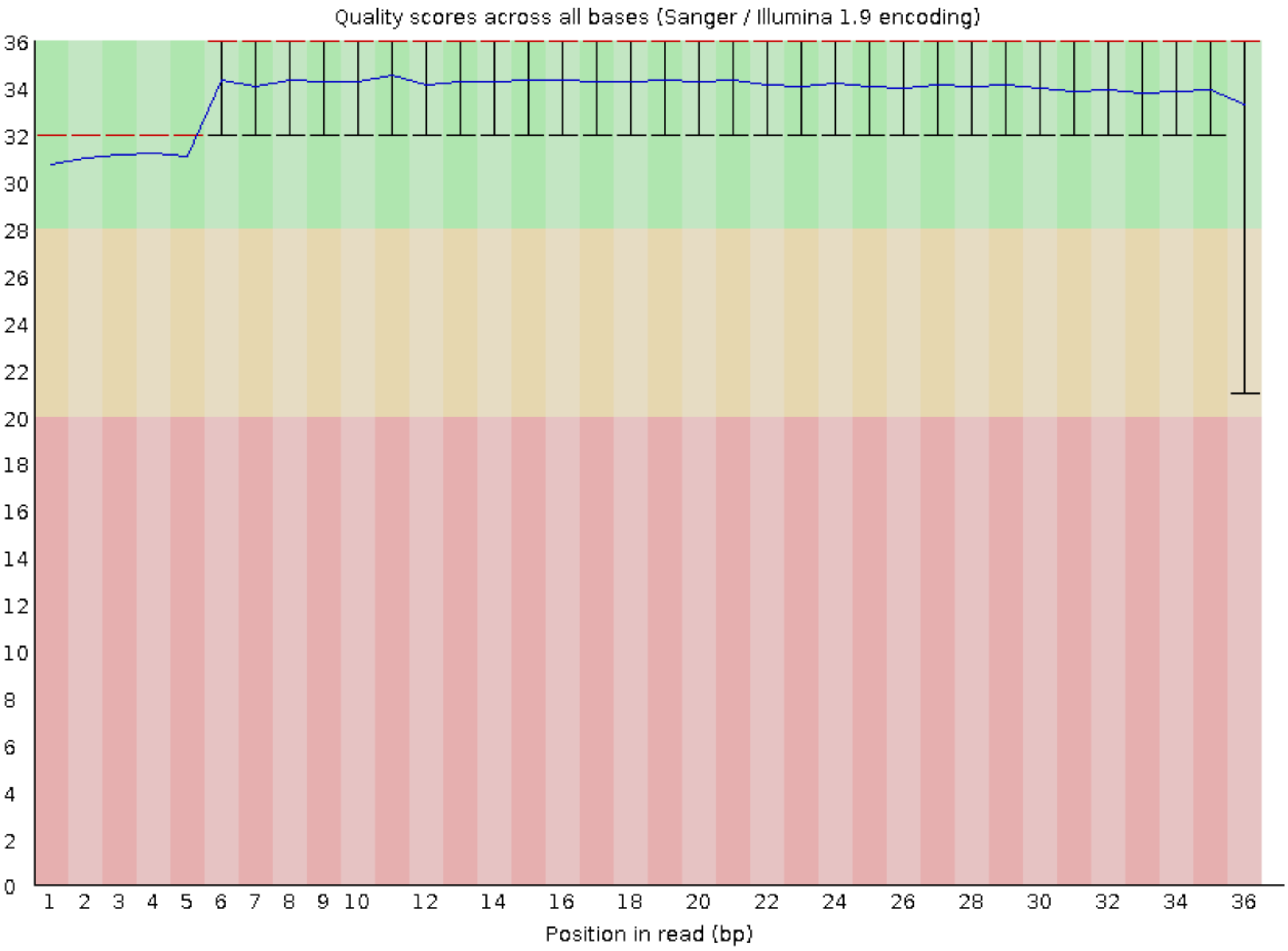

Per tile sequence quality

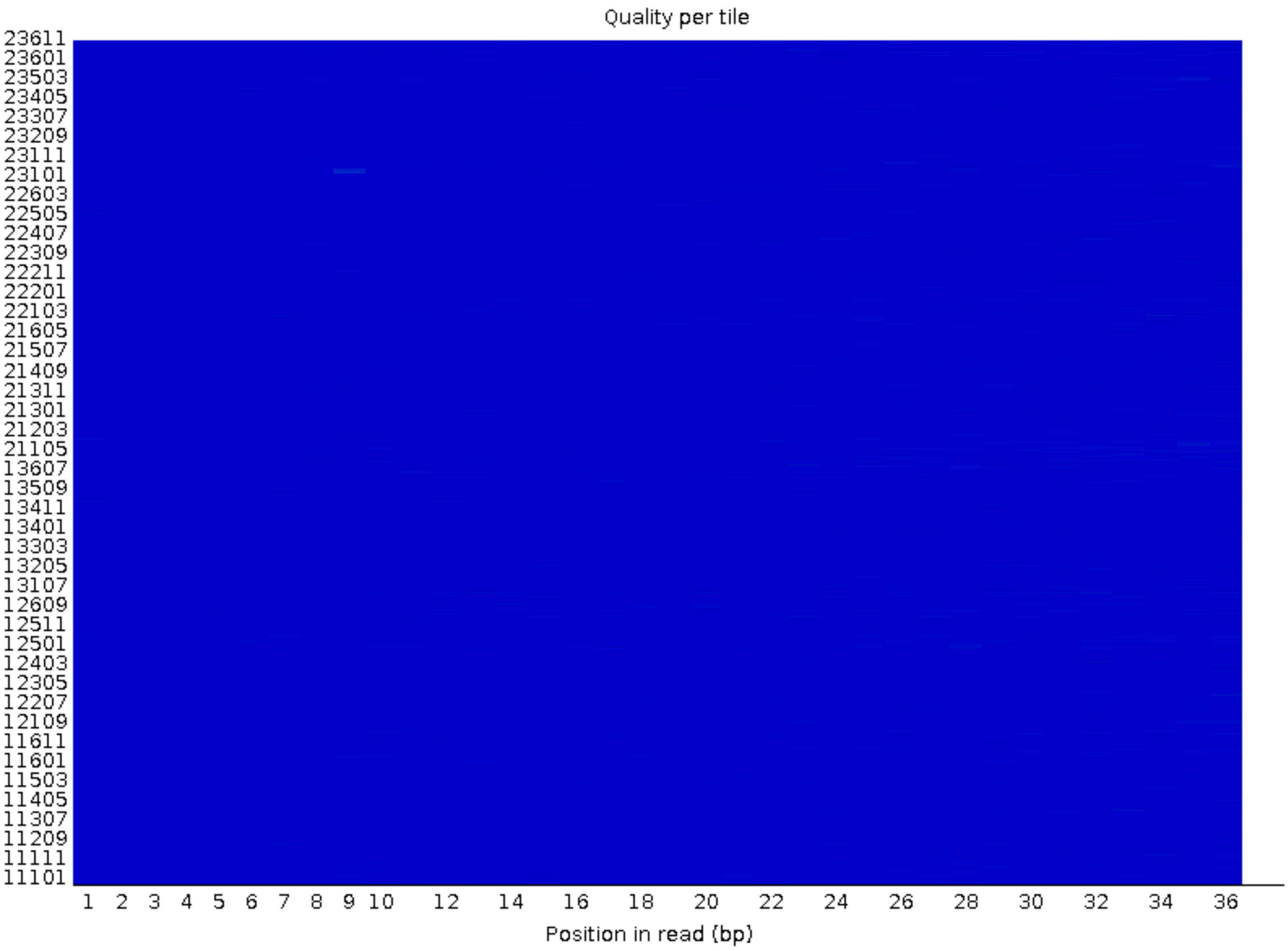

Per sequence quality scores

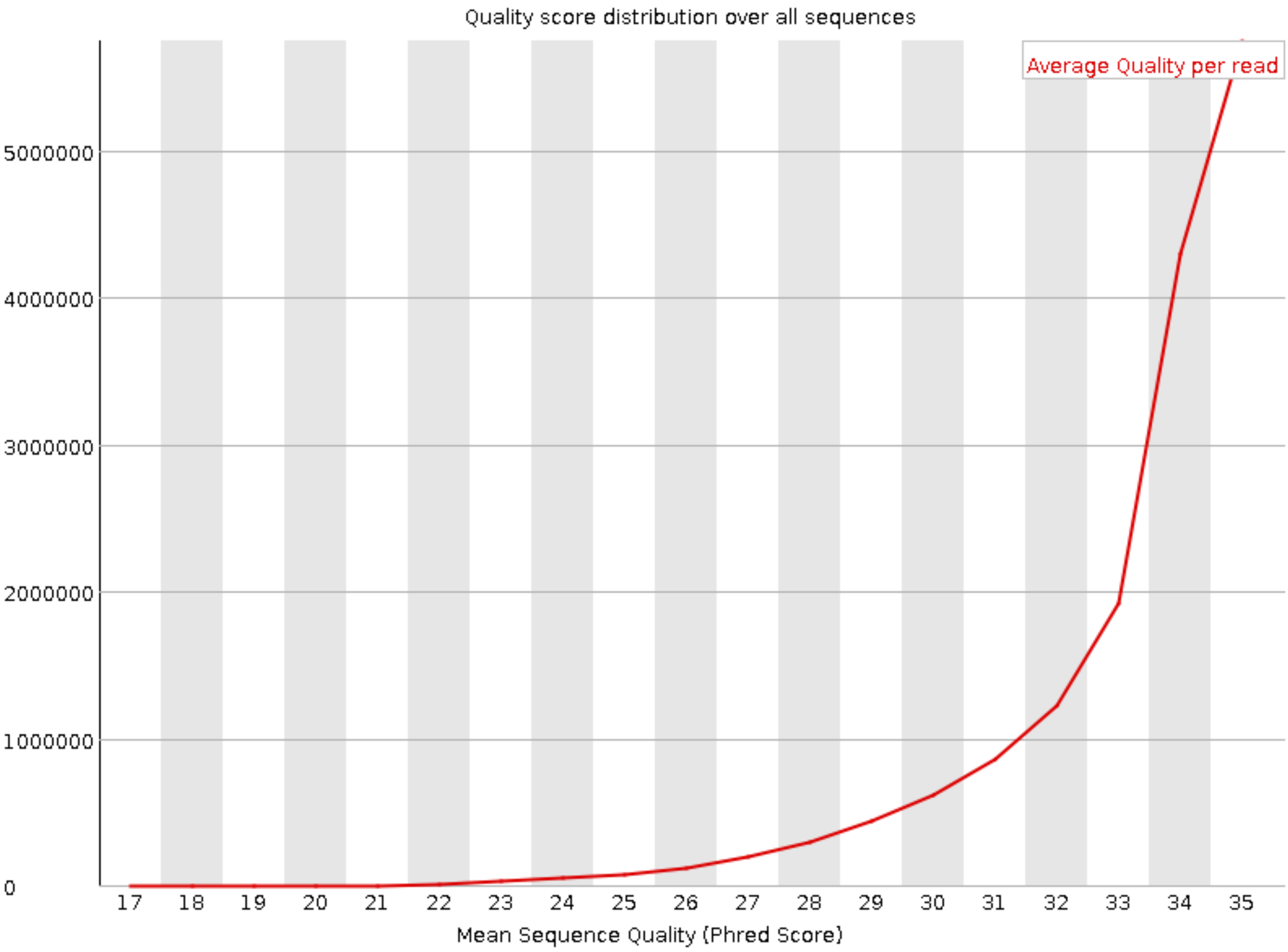

Per base sequence content

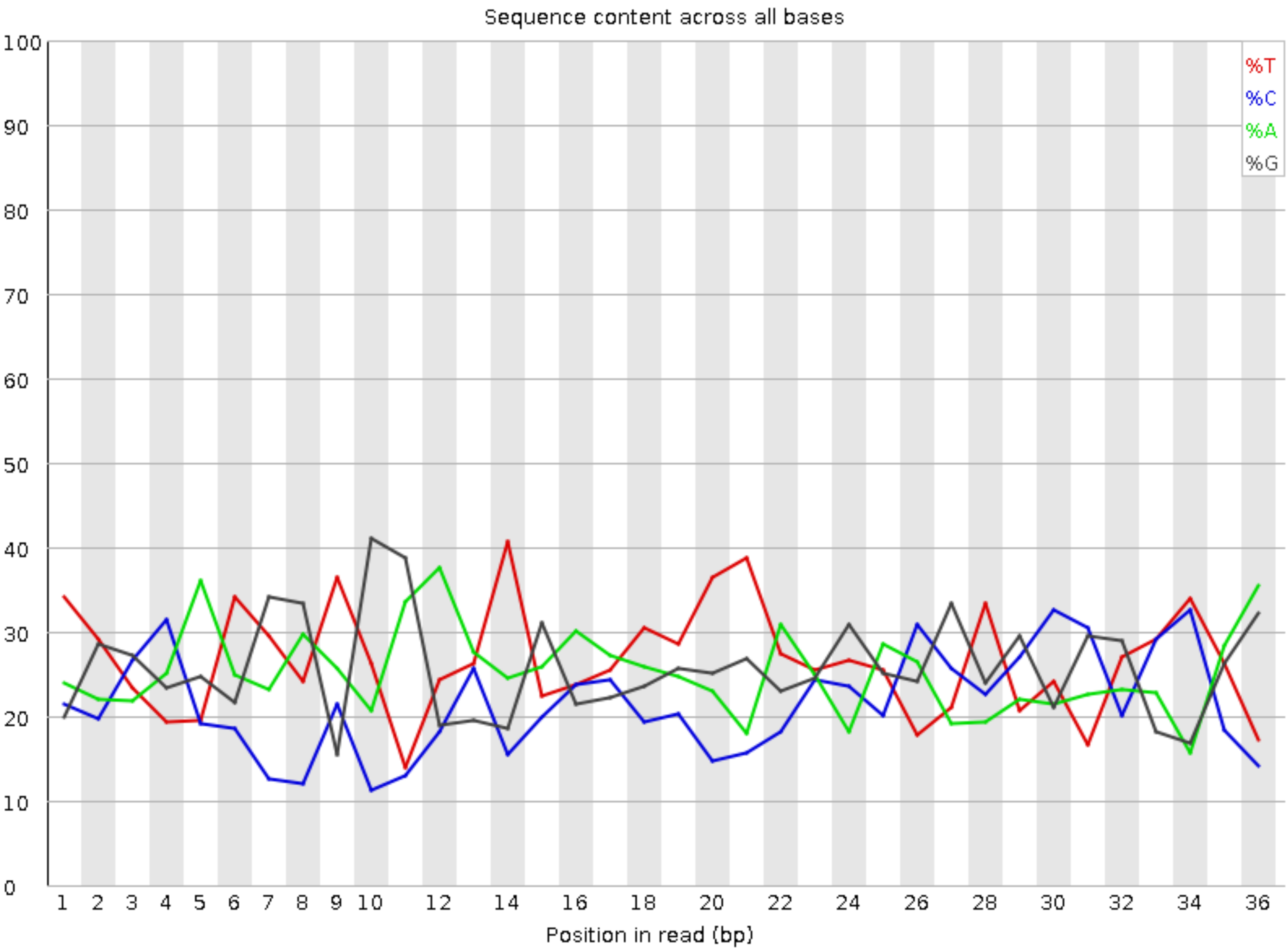

Per sequence GC content

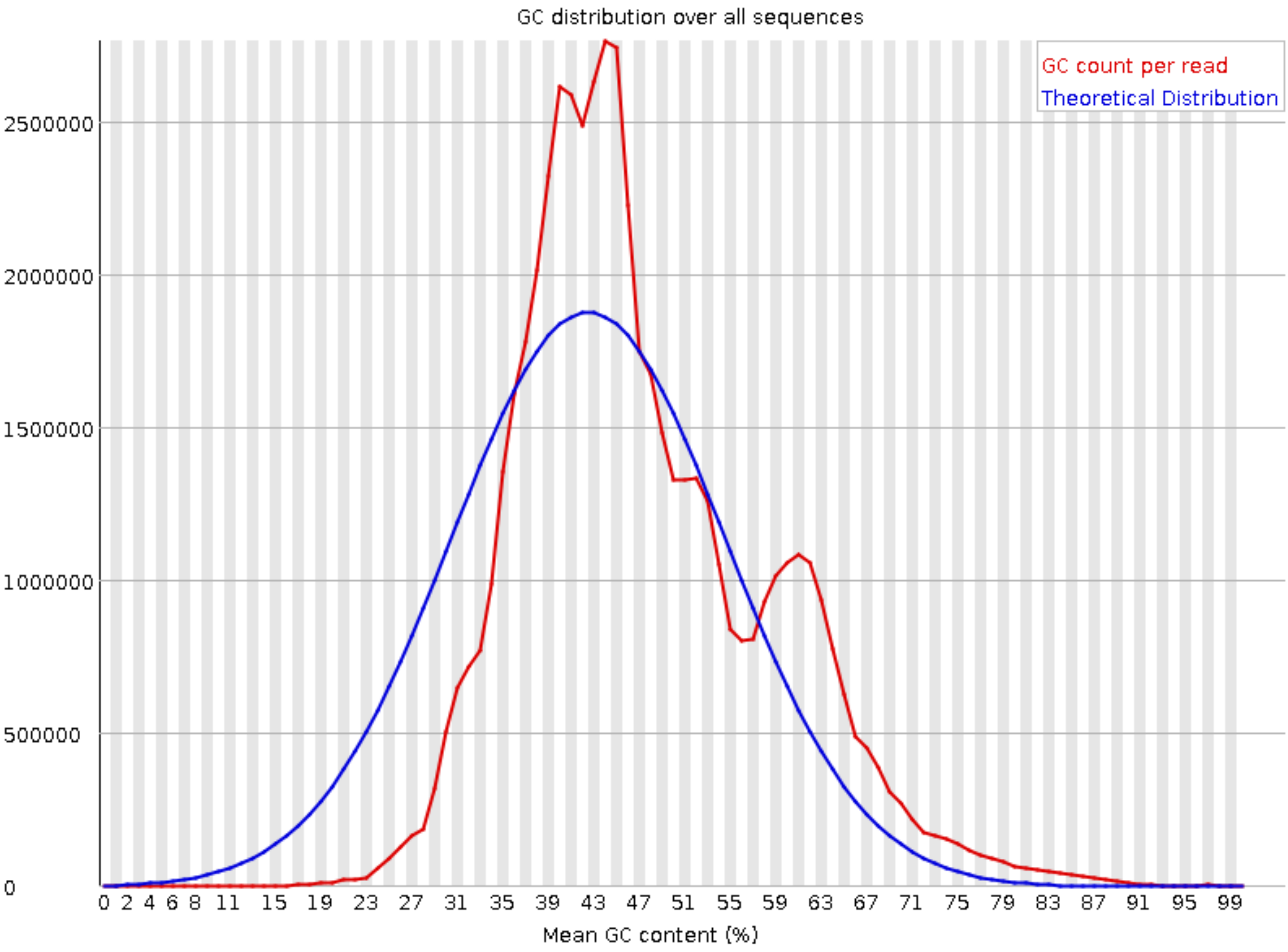

Per base N content

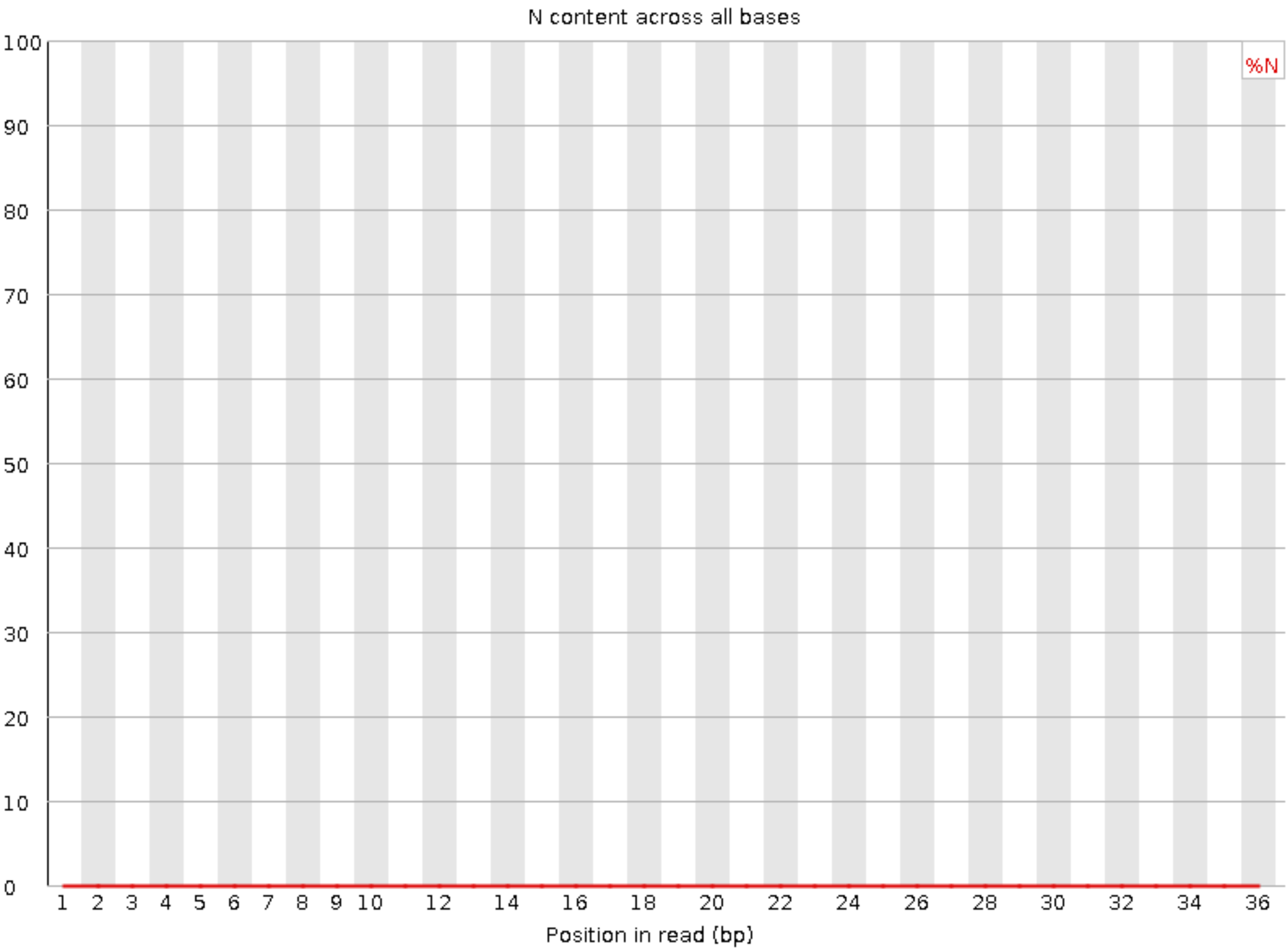

Sequence Length Distribution

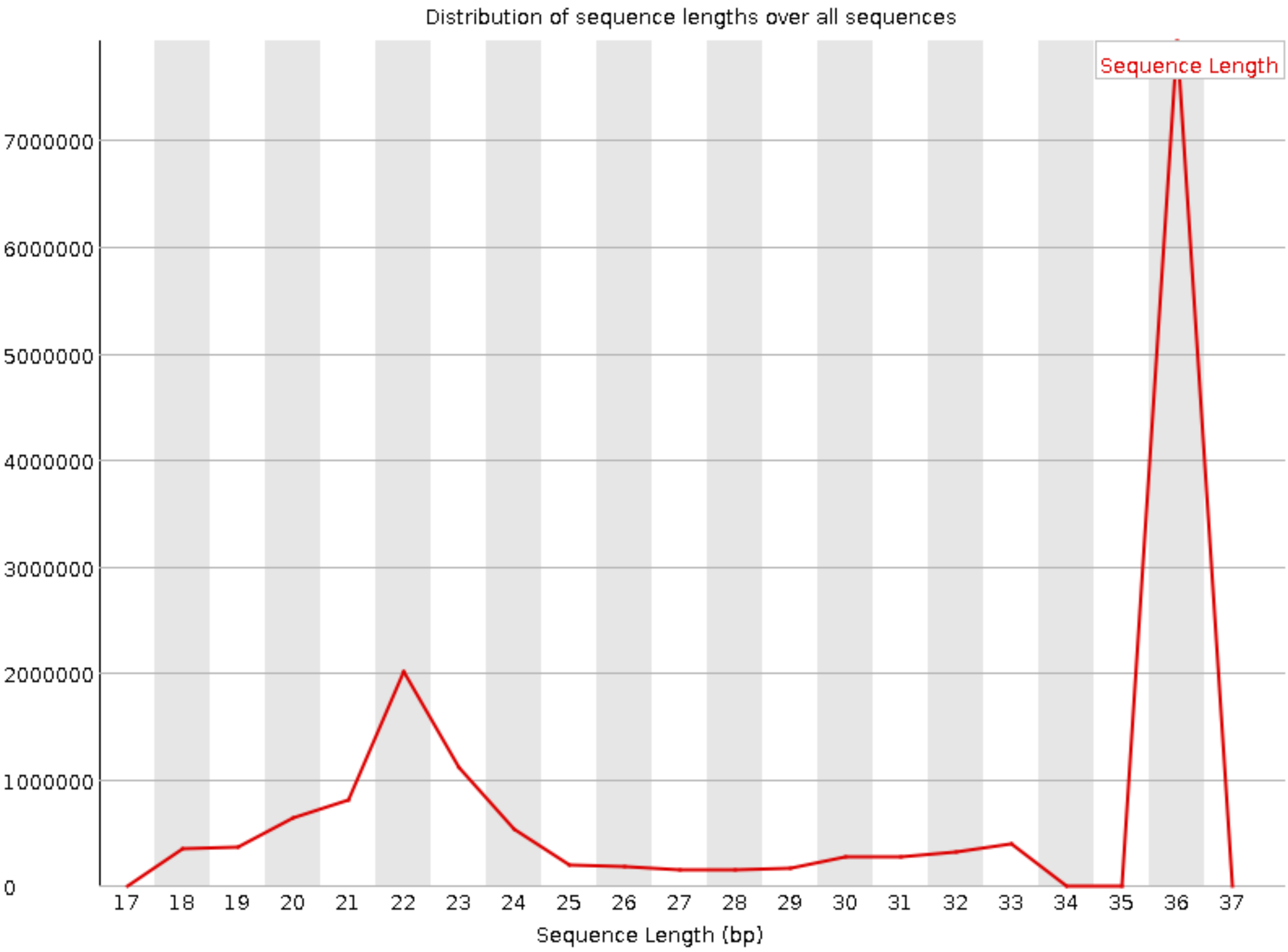

Sequence Duplication Levels

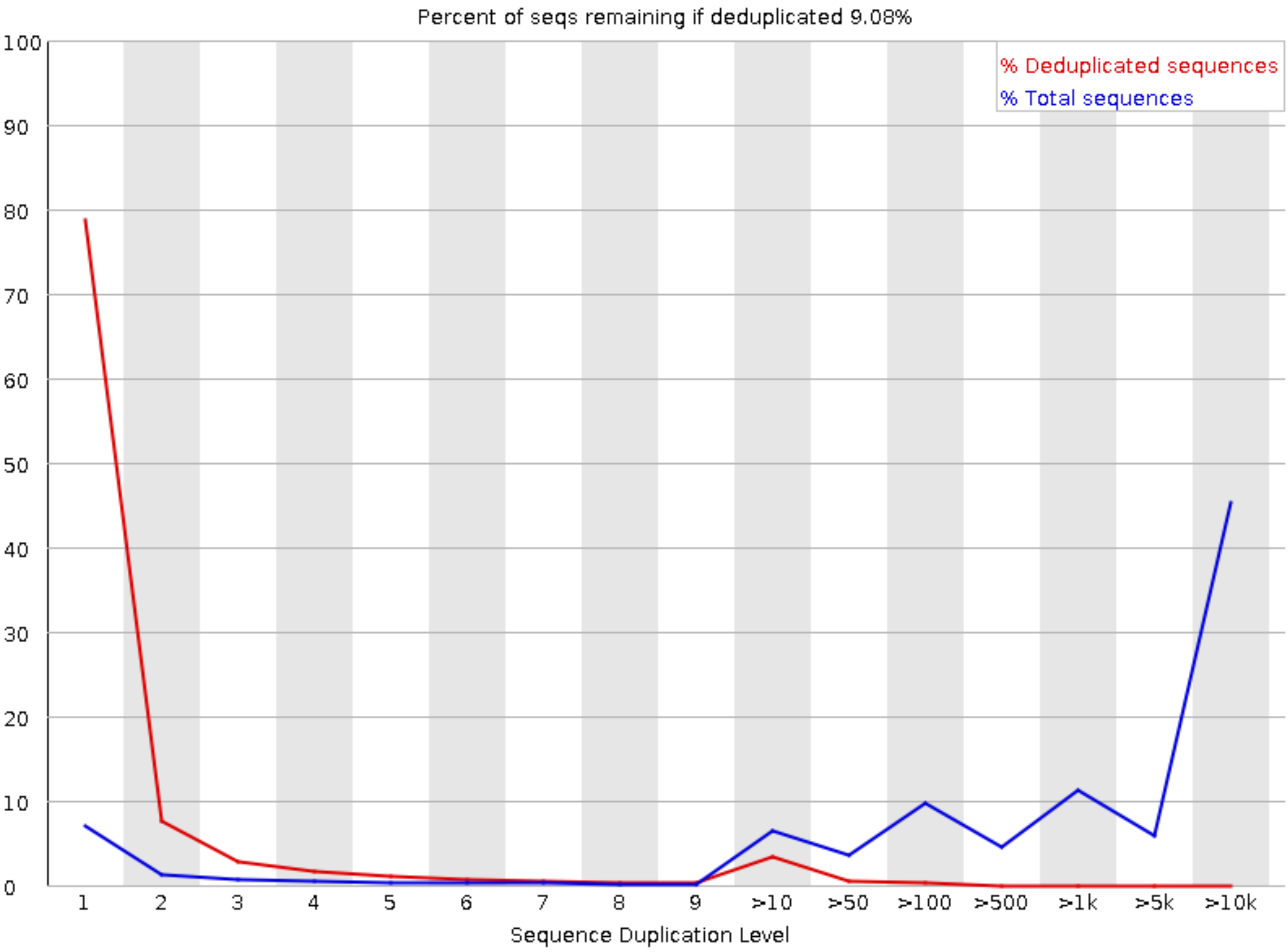

Overrepresented sequences

Sequence	Count	Percentage	Possible Source
TGCTCTGATGAAATCACTAATAGGAAGTGCCGTCAG	492840	3.0811050066393424	No Hit
ATTCAAATCGATCTGCGCCTTT	309696	1.9361372780946713	No Hit
GTTTGTGATGACTTACATGGAATCTCGTTCGGCTGA	266596	1.666687505782855	No Hit
TAGCTTATCAGACTGATGTTGAC	234513	1.4661130963842466	No Hit
GTGAAATGATGGCAATCATCTTTCGGGACTGACCTG	234261	1.464537659200428	No Hit
CCTGGATGATGATAAGCAAATGCTGACTGAACATGA	199580	1.247721242644834	No Hit
TCGCTGCGATCTATTGAAAGTCAGCCCTCGACACAA	171091	1.0696155683202089	No Hit
AGTAGTGATGAAATTCCACTTCATTGGTCCGTGTTT	170737	1.0674024541810352	No Hit
CGCGACCTCAGATCAGACGT	164025	1.0254408098247265	No Hit
TAGCTTATCAGACTGATGTTGA	156292	0.97709614418001	No Hit
GTGCAATGATGTATTTTATTCAACACATCATTCTGA	125041	0.7817231781819456	No Hit
GCCTCTGATGAAGCCTGTGTTGGTAGGGACATCTGA	123284	0.7707388800392109	No Hit

Sequence	Count	Percentage	Possible Source
ATACATGATGATCTCAATCCAACTTGAACTCTCTCA	116542	0.7285896836372093	No Hit
TATCTGTGATGATCTTATCCCGAACCTGAACTTCTG	115040	0.7191995778828625	No Hit
TTTCTATGATGAATCAAACTAGCTCACTATGACCGA	109556	0.6849150639302406	No Hit
TTGAATGATGACTTTAATTGTCGGATACCCCTTCAC	107790	0.6738745001737982	No Hit
CGACTCTTAGCGGTGGATCACTCGGCTCGTGCGTCG	95513	0.5971219513414973	No Hit
ATTCAAATCGAACTGCGCCTTT	94369	0.5899699666657497	No Hit
TGGAAGACTAGTGATTTTGTTGTT	84919	0.5308910722725556	No Hit
CGCTGCGATCTATTGAAAGTCAGCCCTCGACACAAG	84225	0.5265523682821983	No Hit
TGAGGTAGTAGATTGTATAGTT	75782	0.4737689708894218	No Hit
CTCCTACTTGGATAACTGTGGTAATTCTAGAGCTAA	72639	0.45411976823568534	No Hit
CTGGATGATGATAAGCAAATGCTGACTGAACATGAA	71446	0.4466614485519732	No Hit
TGAAATGATGGCAATCATCTTTCGGGACTGACCTGA	67922	0.4246303349179397	No Hit
CTACGGGGATGATTTTACGAACTGAACTCTCTCTTT	65446	0.4091510394134372	No Hit
GCAAATGATGATAAACTGGATCTGACTGACTGTGCT	62563	0.39112728782237066	No Hit
CTCGCTGCGATCTATTGAAAGTCAGCCCTCGACACA	62507	0.390777190670411	No Hit
CAGGACGGTGGCCATGGAAGTCGGAATCCGCTAAGG	57574	0.3599373826236781	No Hit
TGCCTCTGATGAAGCCTGTGTTGGTAGGGACATCTG	54636	0.3415697856155083	No Hit
ACCGGGTGCTGTAGGCTT	50863	0.31798199000222566	No Hit
TTTGAATGATGACTTTAATTGTCGGATACCCCTTCA	50039	0.31283056048053337	No Hit
ATTCAAATCGATCTGCGCCTTC	49049	0.3066413429726749	No Hit
ACAAATGATGAATAACAAAGGGACTTAATACTG	47994	0.300045762699149	No Hit
ACTCCATGATGAACACAAAATGACAAGCATATGGCT	47893	0.2994143374786503	No Hit
TAGCTTATCAGACTGATGTTGAT	47806	0.29887043654614154	No Hit
CTGCAGTGATGACTTTCTTAGGACACCTTTGGATTT	47197	0.29506313001858014	No Hit
TAGCTTATCAGACTGATGTTGACA	44288	0.27687683332124663	No Hit
CTCACTGATGAGTACGTTCTGACTTTCGTTCTTCTG	44270	0.276764302093831	No Hit
ACCGGGTGCTGTAGGCTTT	43723	0.27334460312736786	No Hit
TGGAAGACTAGTGATTTTGTTGT	42804	0.2675992587943154	No Hit
ATGACCTATGAATTGACAGACA	42098	0.26318553398568106	No Hit
CACAGATGATGAACTTATTGACGGGCGGACAGAAAC	41773	0.26115372015734367	No Hit
TAGCTTATCAGACTGATGTTGACT	37028	0.23148923826361817	No Hit
CTGAATGATGATATCCCACTAACTGAGCAGTCAGTA	36508	0.22823833613827837	No Hit
TGAGGTAGTAGTTTGTGCTGTT	36368	0.2273630932583792	No Hit
TGAGGTAGTAGGTTGTATAGTT	36078	0.22555009015001662	No Hit
GCAGCTGATGATACAGCTTCTTTCCCCATC	35959	0.22480613370210234	No Hit
TCTCCTACTTGGATAACTGTGGTAATTCTAGAGCTA	35586	0.22247423660065666	No Hit
GGCTGGTCCGATGGTAGTGGGTTATCAGAACT	34679	0.21680391308588134	No Hit

Sequence	Count	Percentage	Possible Source
CTGACCTATGAATTGACAGCC	33965	0.21234017439839556	No Hit
GTGAAATGATGGCAAATCATCTTTCGGGACTGACCT	33574	0.2098957460695343	No Hit
TAATACTGCCGGGTAATGATGGA	33198	0.20754509376351937	No Hit
TCCTACTTGGATAACTGTGGTAATTCTAGAGCTAAT	33188	0.20748257641495516	No Hit
ATATATGATGACTTAGCTTTTTTCCCCGAC	33168	0.2073575417178267	No Hit
GATGGGAGACCGCCTGGGAATACCGGGTGCTGTAGG	33112	0.20700744456586703	No Hit
CGCGACCTCAGATCAGACGTGGCGACCCGCTGAATT	32560	0.2035564869251217	No Hit
GCATTGGTGGTTCAGTGGTAGAATTCTCGCCT	32472	0.20300633425775652	No Hit
TGTAACAGCAACTCCATGTGGA	31829	0.19898646874507678	No Hit
TAATACTGCCTGGTAATGATGAC	31569	0.19736101768240685	No Hit
CTAGACTGAAGCTCCTTGAGG	31231	0.195247931300936	No Hit
TGAGGTAGTAGTTTGTACAGTT	30909	0.1932348726771679	No Hit
CTTAATGATGACTGTTTTTTTTGATTGCTTGAAGCA	29938	0.1871644381315815	No Hit
GCATATGATGGAAAAGTTTTAATCTCCTGACACTTG	29339	0.18341964895258434	No Hit
TAGGGTGATGAAAAAGAATCCTTAGGCGTGGTTGTG	29185	0.18245688178469524	No Hit
TCAGATGATGAATTTAACTGTTCAACTGCTGAATGA	29115	0.18201926034474566	No Hit
GAGAAGACGGTCGAACTTGACTATCT	29078	0.18178794615505803	No Hit
AACTGTGATGAAAGATTTGGTCTGTATGTAAT	28381	0.17743048696013144	No Hit
ATTCAAATCGATCTGCGCCTT	28325	0.17708038980817178	No Hit
GCTTAATGATGACTGTTTTTTTTGATTGCTTGAAGC	28170	0.17611137090542625	No Hit
TGGGAGACCGCCTGGGAATACCGGGTGCTGTAGGCT	28081	0.17555496650320465	No Hit
TAACACTGTCTGGTAACGATGTT	27834	0.17401078799366823	No Hit
CGCGACCTCAGATCAGACGC	27607	0.1725916441812603	No Hit
TTCAAATCGATCTGCGCCTTT	27585	0.172454106014419	No Hit
TGAAATGATGGCAAATCATCTTTCGGGACTGACCTG	27428	0.17147258364196064	No Hit
TTCAAGTAATCCAGGATAGGCT	27307	0.1707161237243335	No Hit
TACCCTGTAGATCCGAATTTGT	27222	0.17018472626153758	No Hit
AGCAGCATTGTACAGGGCTATGA	26673	0.16675252382536154	No Hit
AGAAATGAAGAAACTAAAATTGGTCTTAGTATTGAA	26598	0.16628364371112983	No Hit
TCAGTGCACTACAGAACTTTGT	26569	0.1661023434002936	No Hit
TTCCTATGATGAGGACCTTTTCACAGACCTGTACTG	25968	0.16234505075158356	No Hit
TAATACTGCCTGGTAATGATGA	25907	0.1619636949253418	No Hit
GACTCTTAGCGGTGGATCACTCGGCTCGTGCGTCGA	25848	0.16159484256881285	No Hit
TCGCGTGATGACATTCTCCGGAATCGCTGTACGGCC	25820	0.16141979399283302	No Hit
AATGGATTTTTGGAGCAGG	24936	0.1558932603797554	No Hit
AAGCTATGATGAATTTGATTGCATTGATCGTCTGAC	24313	0.15199842956420406	No Hit
TAGCTTATCAGACTGATGTTG	22943	0.14343355281090503	No Hit

Sequence	Count	Percentage	Possible Source
CTGCTGTGATGACATTCCAATTAAAGCACGTGTTAG	22894	0.14312721780294033	No Hit
GGCTGGTCCGATGGTAGTGGGTTATCAGAAC	22841	0.14279587585554993	No Hit
AGTCTGTGATGAATTGCTTTGACTTCTGACACCTCG	22803	0.14255830993100585	No Hit
TTCAAATCGAACTGCGCCTTT	22495	0.14063277559522766	No Hit
CTGACCTATGAATTGACAGCT	22112	0.13823836114521781	No Hit
TACGGGGATGATTTTACGAACTGAACTCTCTCTTTC	22092	0.13811332644808935	No Hit
CTGACCTATGAATTGACAGCCAT	21599	0.13503122116387298	No Hit
ACGGCCCTGGCGGAGCGCTGAGAAGACGGTCGAACT	21282	0.133049421214387	No Hit
CTGCGATGATGGCATTTCTTAGGACACCTTTGGATT	21146	0.13219918527391353	No Hit
TAAAGTGCTTATAGTGCAGGTAG	21057	0.1316427808716919	No Hit
TCGCGAAGGCCCGCGGCGGGTGTTGACGCGATGTGA	21019	0.13140521494714785	No Hit
TGTAATGATGTTGATCAAATGTCTGACCTGAAATGA	20881	0.1305424755369615	No Hit
TCAAATGATGAAATCACCCAAAATAGCTGGAATTAC	20811	0.13010485409701192	No Hit
TACAATGATGATAACATAGTTCAGCAGACTAACGCT	20184	0.1261850163420349	No Hit
TAATACTGTCTGGTAAAACCGT	20113	0.1257411431672289	No Hit
TCGTACGACTCTTAGCGGTGGATCACTCGGCTCGTG	20071	0.12547857030325915	No Hit
GCATTGGTGGTTCAGTGGTAGAATTCTCGCC	19718	0.12327170789894196	No Hit
TGCATATGATGGAAAAGTTTTAATCTCCTGACACTT	19415	0.12137743223744589	No Hit
AATACATGATGATCTCAATCCAACTTGAACTCTCTC	19051	0.11910180074970805	No Hit
ACAGATGATGAACTTATTGACGGGCGGACAGAAACT	18910	0.11822030613495246	No Hit
CTCCATGATGAACACAAAATGACAAGCATATGGCTG	18630	0.1164698203751541	No Hit
TTCAAATCGATCTGCGCCTTTT	17967	0.11232492016534588	No Hit
TTCACAGTGGCTAAGTTCTGC	17951	0.11222489240764312	No Hit
TGTAAACATCCCCGACTGGAAG	17897	0.1118872987253963	No Hit
TTCAAATCGAACTGCGCCTTTT	17659	0.11039938582956771	No Hit
GTAGGGTGATGAAAAAGAATCCTTAGGCGTGGTTGT	17296	0.10813000607668628	No Hit
AGCAAATGATGATAAACTGGATCTGACTGACTGTGC	16523	0.10329741503267156	No Hit
CCTACTTGGATAACTGTGGTAATTCTAGAGCTAATA	16498	0.103141121661261	No Hit
AGAAGACGGTCGAACTTGACTATCT	16340	0.10215334755394623	No Hit
TGTAAACATCCCCGACTGGAAGCT	16120	0.10077796588553324	No Hit

Adapter Content

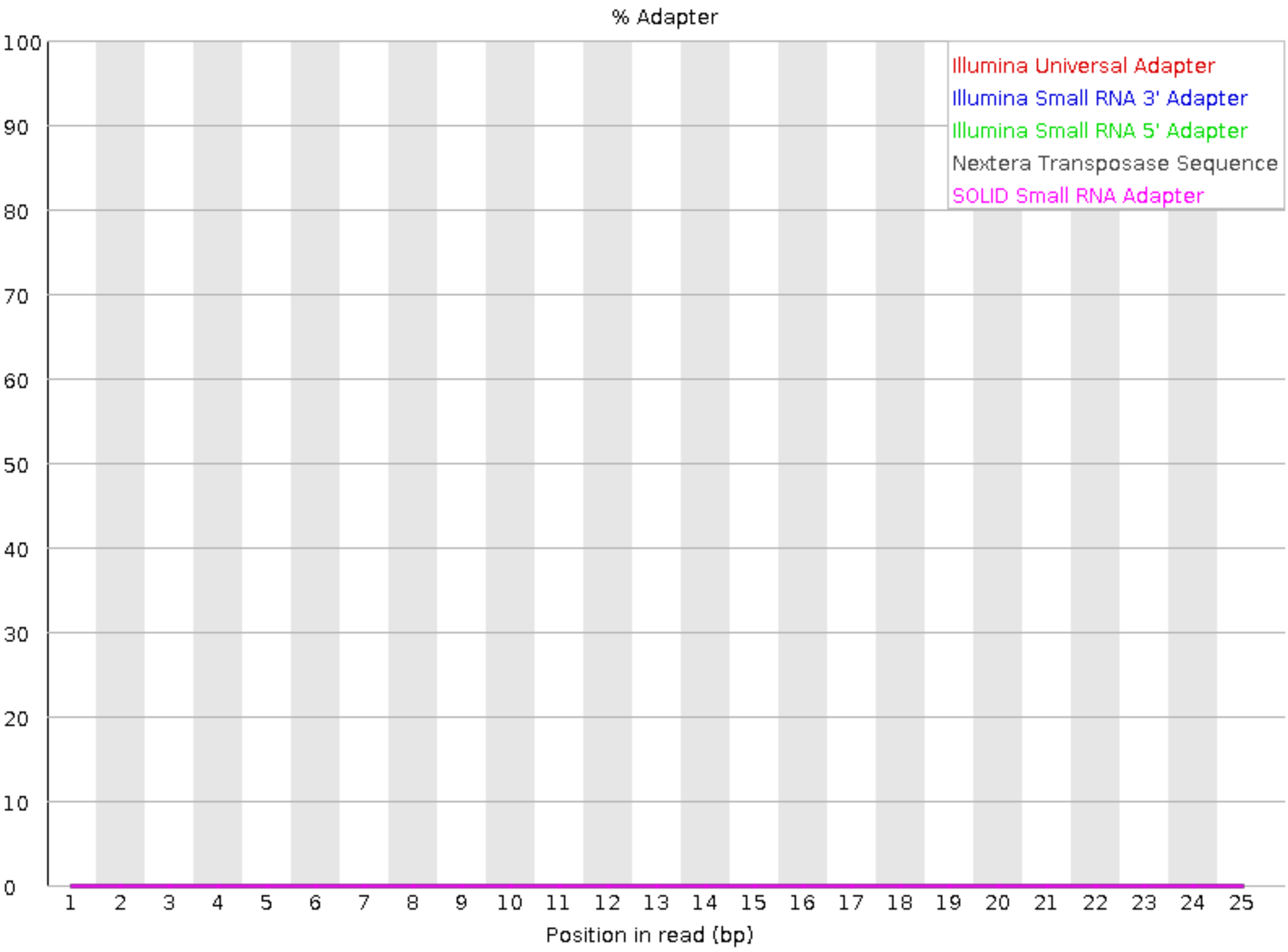
% Adapter
Illumina Universal Adapter
Illumina Small RNA 3' Adapter
Illumina Small RNA 5' Adapter
Nextera Transposase Sequence
SOLID Small RNA Adapter
100
90
80
70
60
50
40
30
20
10
0
1 2 3 4 5 6 7 8 9 10 11 12 13 14 15 16 17 18 19 20 21 22 23 24 25
Position in read (bp)
