# Supplementary material for: CD44 knockdown alters miRNA expression and their target genes in colon cancer
Source: Front Immunol. 2025 May 14;16:1552665. doi: 10.3389/fimmu.2025.1552665 (PMC12116639; doi:10.3389/fimmu.2025.1552665)

FastQC Report

Summary

- Basic Statistics
- Per base sequence quality
- Per tile sequence quality
- Per sequence quality scores
- Per base sequence content
- Per sequence GC content
- Per base N content
- Sequence Length Distribution
- Sequence Duplication Levels
- Overrepresented sequences
- Adapter Content

Basic Statistics

Measure	Value
Filename	shCD44_6.fastq.gz
File type	Conventional base calls
Encoding	Sanger / Illumina 1.9
Total Sequences	19901198
Sequences flagged as poor quality	0
Sequence length	18-36
%GC	47

Per base sequence quality

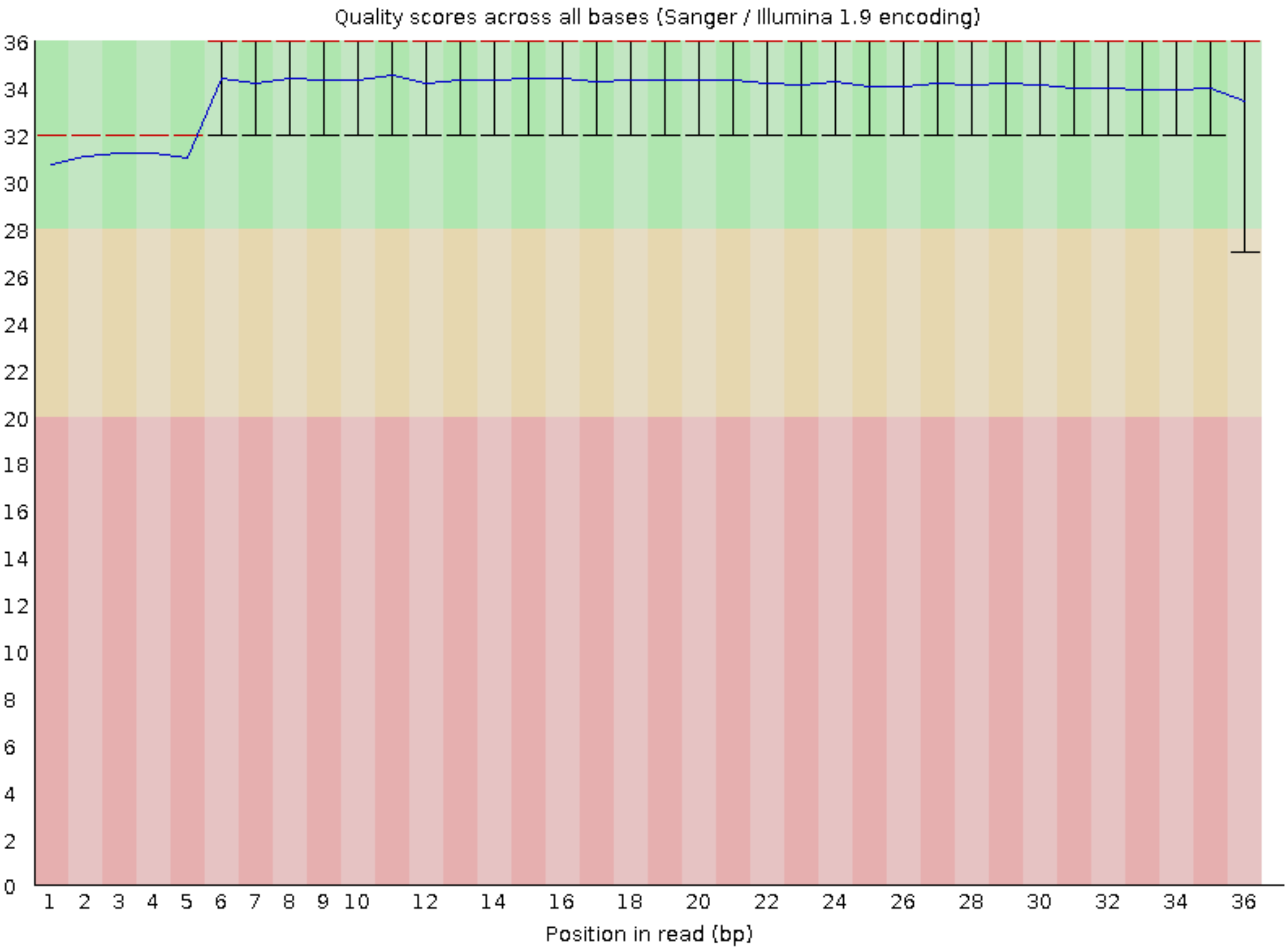

Per tile sequence quality

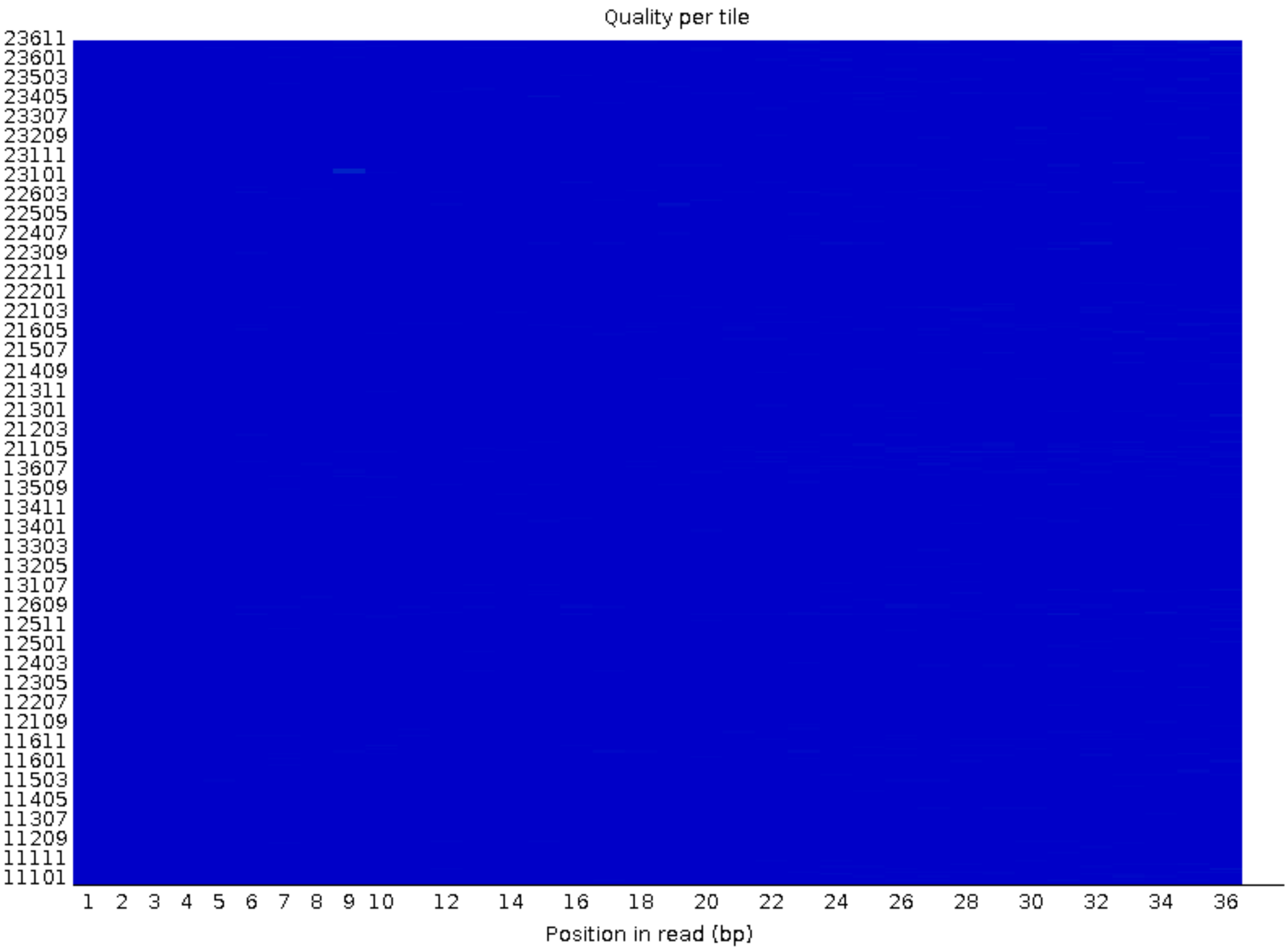

Per sequence quality scores

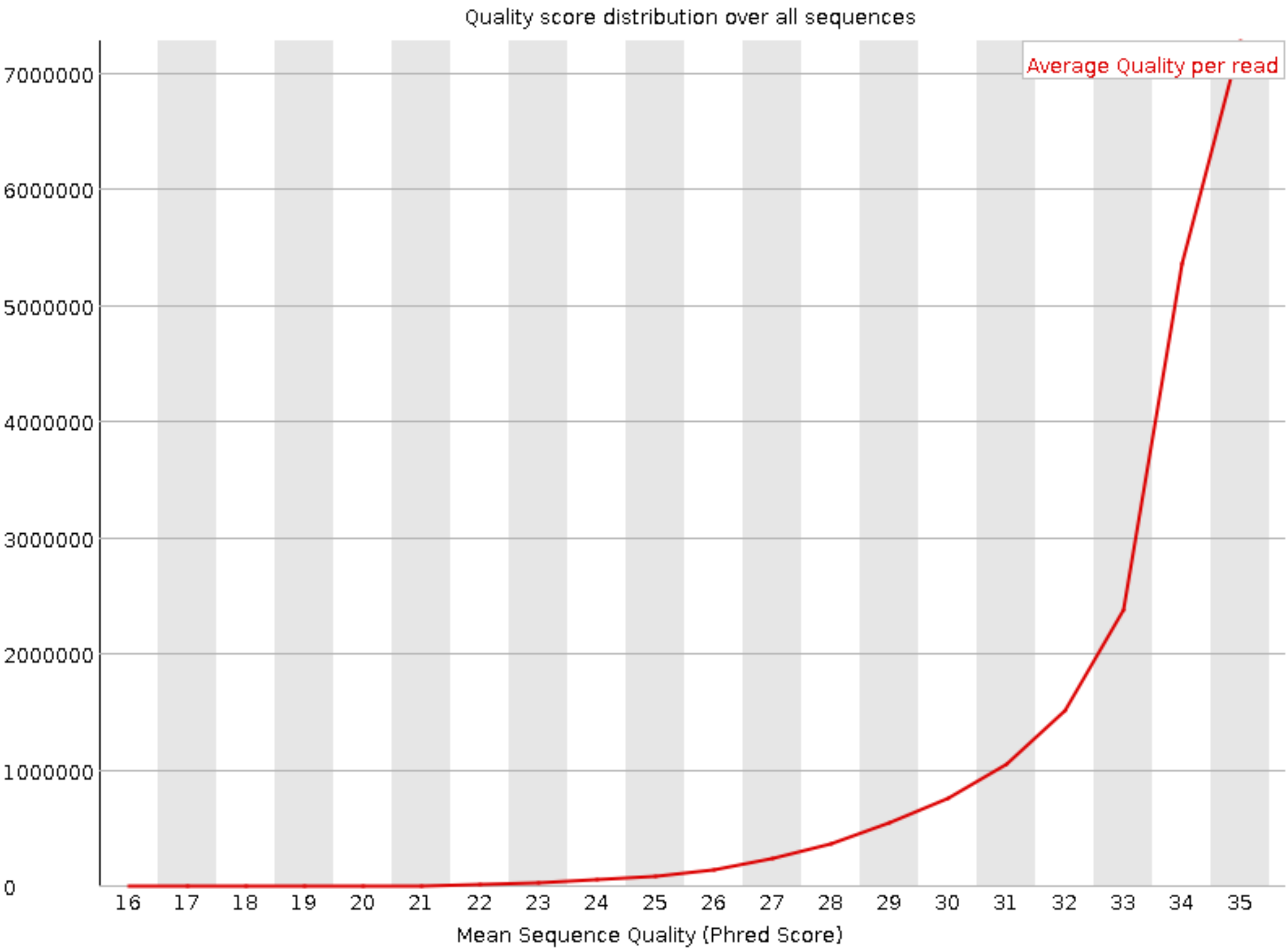

Per base sequence content

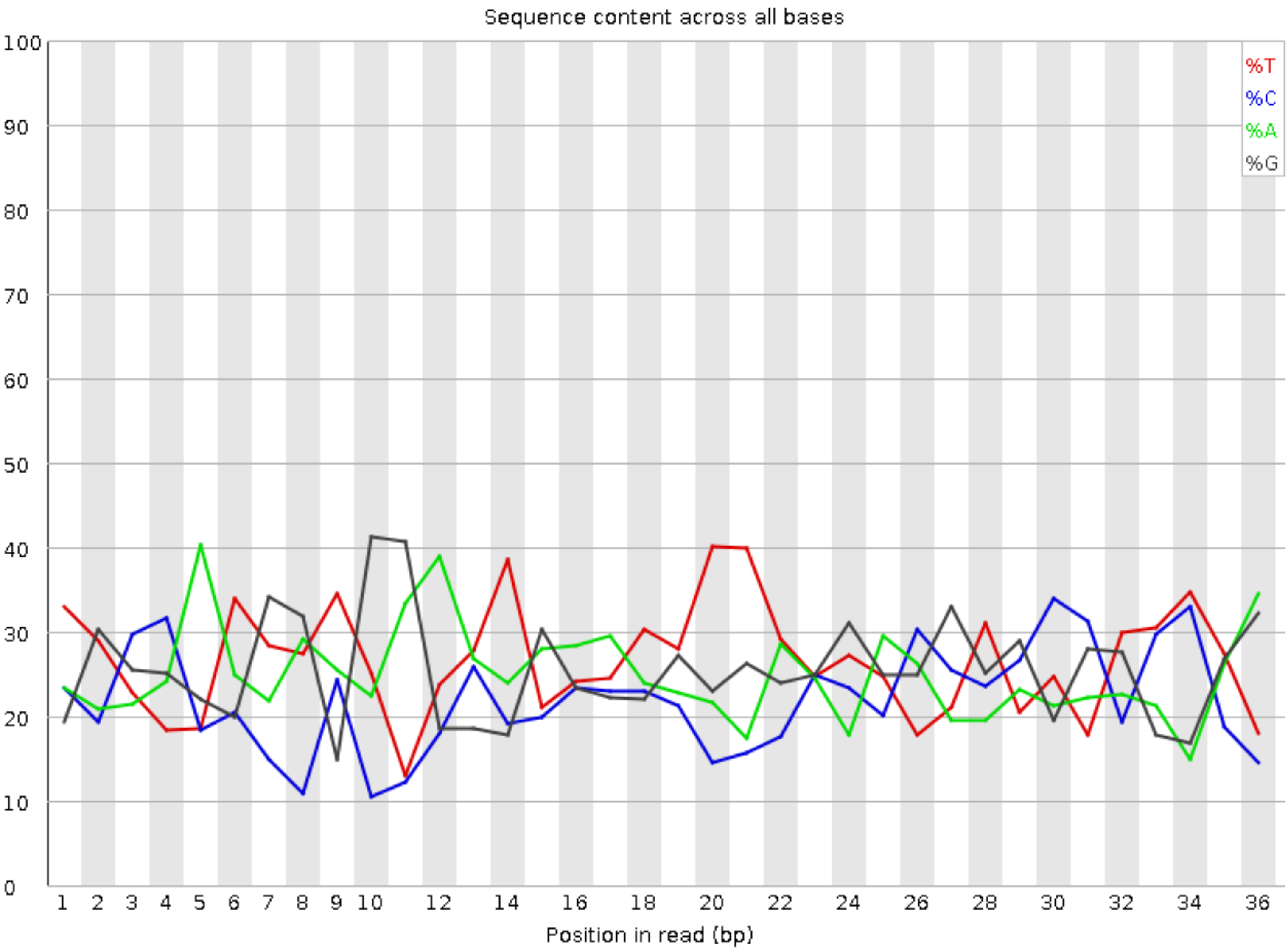

Per sequence GC content

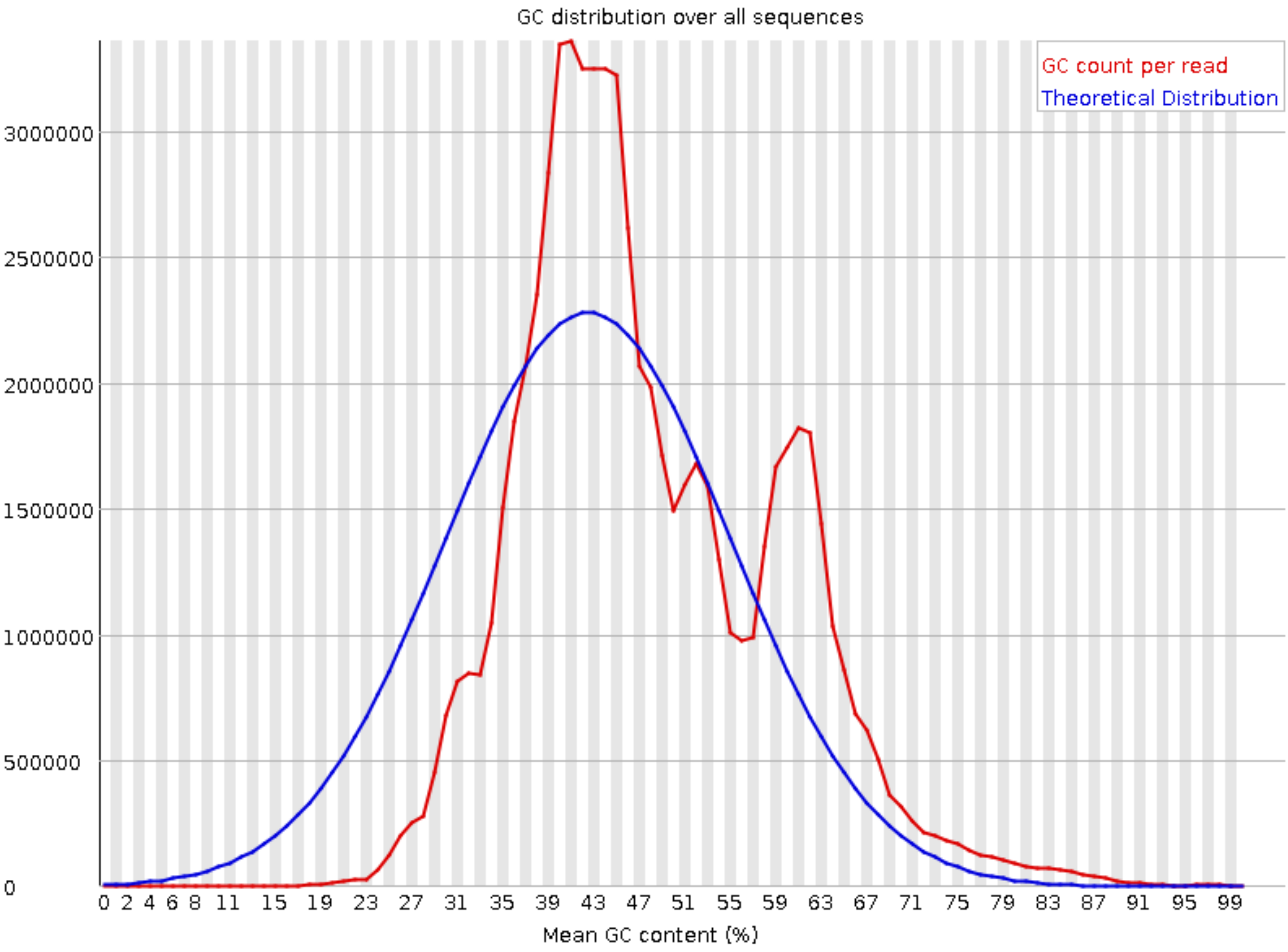

Per base N content

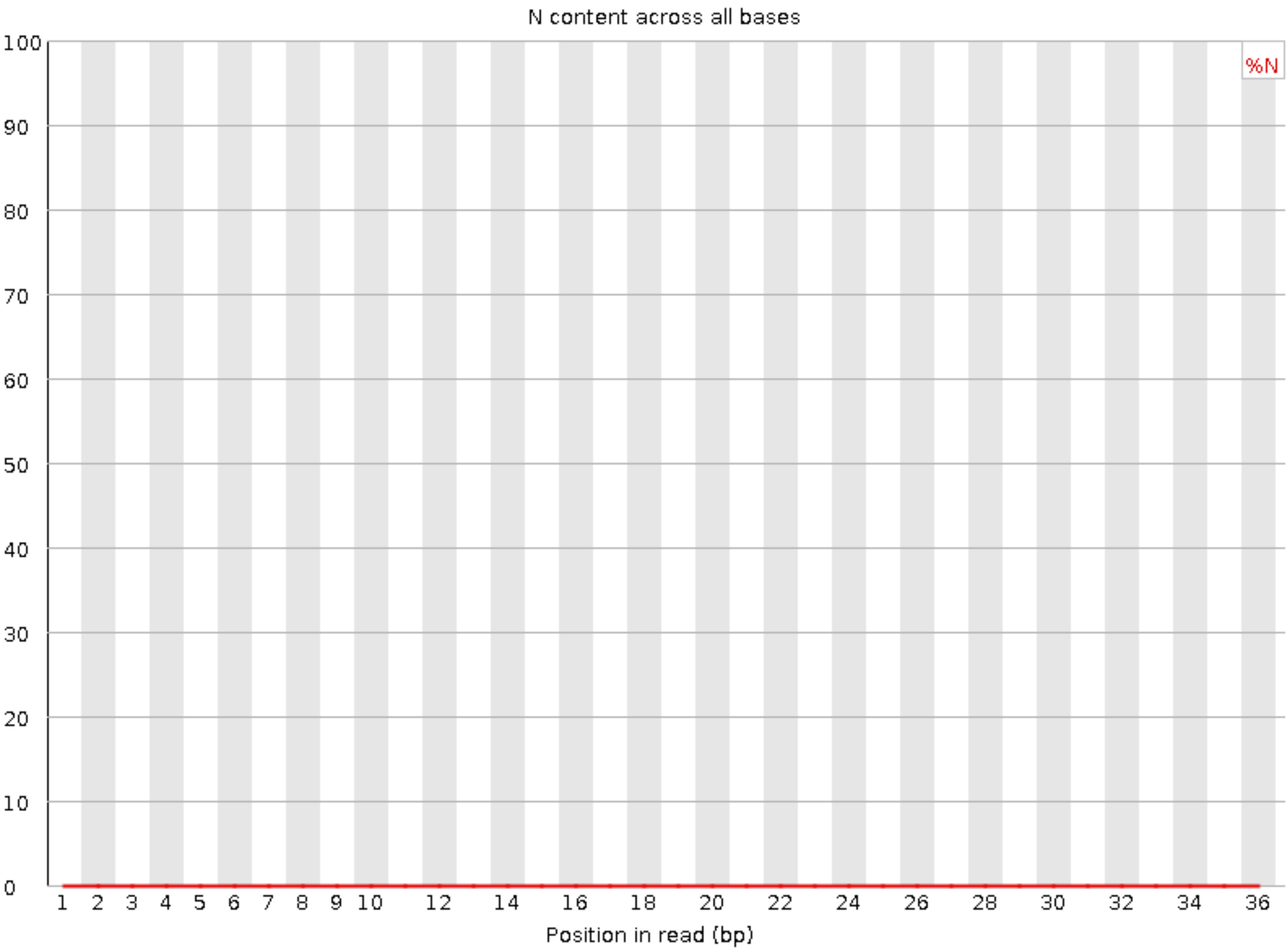

Sequence Length Distribution

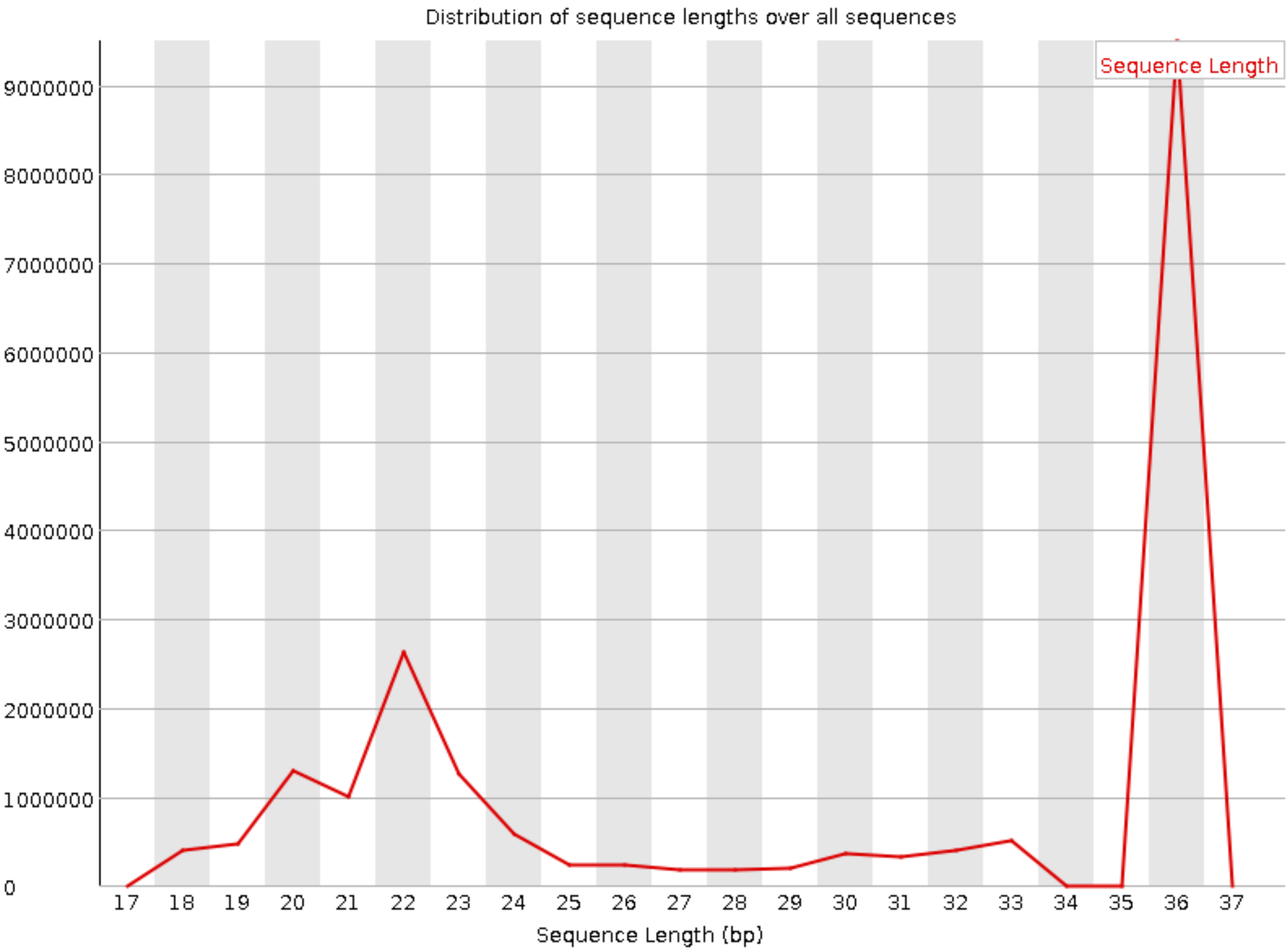

Sequence Duplication Levels

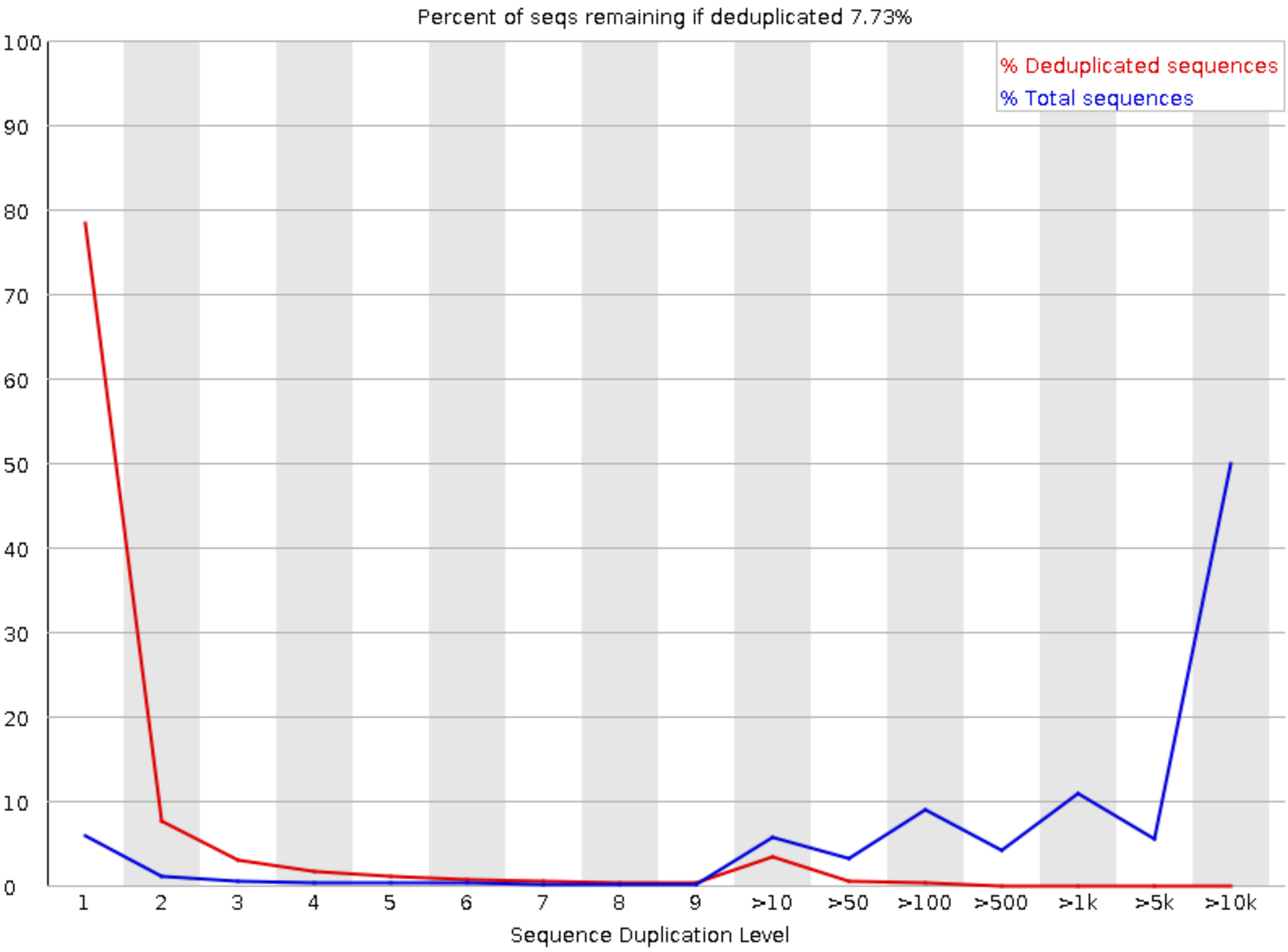

Overrepresented sequences

Sequence	Count	Percentage	Possible Source
CGCGACCTCAGATCAGACGT	611257	3.071458311203175	No Hit
TGCTCTGATGAAATCACTAATAGGAAGTGCCGTCAG	578456	2.9066390877574304	No Hit
ATTCAAATCGATCTGCGCCTTT	505204	2.538560743931094	No Hit
GTGAAATGATGGCAATCATCTTTCGGGACTGACCTG	294008	1.477338198434084	No Hit
TAGCTTATCAGACTGATGTTGAC	263476	1.323920298667447	No Hit
GCCTCTGATGAAGCCTGTGTTGGTAGGGACATCTGA	241696	1.2144796509235274	No Hit
GTTTGTGATGACTTACATGGAATCTCGTTCGGCTGA	234123	1.1764266653695923	No Hit
CCTGGATGATGATAAGCAAATGCTGACTGAACATGA	229371	1.152548705861828	No Hit
AGTAGTGATGAAATTCCACTTCATTGGTCCGTGTTT	201152	1.0107532219919624	No Hit
TAGCTTATCAGACTGATGTTGA	195437	0.9820363578112232	No Hit
TATCTGTGATGATCTTATCCCGAACCTGAACTTCTG	183534	0.9222258881098514	No Hit
GTGCAATGATGTATTTTATTCAACACATCATTCTGA	168683	0.8476022398249593	No Hit

Sequence	Count	Percentage	Possible Source
TTGAATGATGACTTTAATTGTCGGATACCCCTTCAC	157805	0.7929422138305442	No Hit
ATACATGATGATCTCAATCCAACTTGAACTCTCTCA	156445	0.7861084543754603	No Hit
TTTCTATGATGAATCAAACTAGCTCACTATGACCGA	149565	0.7515376712497408	No Hit
TCGCTGCGATCTATTGAAAGTCAGCCCTCGACACAA	122208	0.6140735849168477	No Hit
CGACTCTTAGCGGTGGATCACTCGGCTCGTGCGTCG	116849	0.5871455577699393	No Hit
ATTCAAATCGAACTGCGCCTTT	114424	0.5749603616827489	No Hit
TGAAATGATGGCAATCATCTTTCGGGACTGACCTGA	112272	0.5641469423097042	No Hit
GCAAATGATGATAAACTGGATCTGACTGACTGTGCT	86908	0.4366973284723864	No Hit
TGGAAGACTAGTGATTTTGTTGTT	85038	0.4273009092216459	No Hit
TGCCTCTGATGAAGCCTGTGTTGGTAGGGACATCTG	84257	0.42337652235810125	No Hit
CGCGACCTCAGATCAGACGC	83202	0.41807533395728236	No Hit
CTACGGGGATGATTTTACGAACTGAACTCTCTCTTT	82564	0.4148694968011474	No Hit
CTGGATGATGATAAGCAAATGCTGACTGAACATGAA	81938	0.4117239575225572	No Hit
ACAAATGATGAATAACAAAGGGACTTAATACTG	80830	0.40615645349591517	No Hit
CGCGACCTCAGATCAGACGTGGCGACCCGCTGAATT	77941	0.39163973947699027	No Hit
TGAGGTAGTAGATTGTATAGTT	74876	0.37623865658740746	No Hit
CAGGACGGTGGCCATGGAAGTCGGAATCCGCTAAGG	74193	0.3728067023904792	No Hit
CTCGCTGCGATCTATTGAAAGTCAGCCCTCGACACA	72491	0.36425445342536666	No Hit
CGCTGCGATCTATTGAAAGTCAGCCCTCGACACAAG	71422	0.3588829175007454	No Hit
ATTCAAATCGATCTGCGCCTTC	68872	0.3460696185224628	No Hit
ACTCCATGATGAACACAAAATGACAAGCATATGGCT	66270	0.33299502874148584	No Hit
ACCGGGTGCTGTAGGCTT	64570	0.32445282942263076	No Hit
CTCCTACTTGGATAACTGTGGTAATTCTAGAGCTAA	61951	0.3112928176484652	No Hit
CGCGACCTCAGATCAGACG	61216	0.3075995726488426	No Hit
TTTGAATGATGACTTTAATTGTCGGATACCCCTTCA	55212	0.27743053458389794	No Hit
GCAGCTGATGATACAGCTTCTTTCCCCATC	55076	0.2767471586383895	No Hit
CTGCAGTGATGACTTTCTTAGGACACCTTTGGATTT	54620	0.2744558392916849	No Hit
TGAGGTAGTAGTTTGTGCTGTT	53482	0.2687375905711807	No Hit
TAGCTTATCAGACTGATGTTGACA	53077	0.2667025372040417	No Hit
ATATATGATGACTTAGCTTTTTTCCCCGAC	52671	0.26466245901377394	No Hit
CTCACTGATGAGTACGTTCTGACTTTCGTTCTTCTG	52138	0.2619842282861564	No Hit
GTGAAATGATGGCAAATCATCTTTCGGGACTGACCT	50449	0.2534973020217175	No Hit
ACCGGGTGCTGTAGGCTTT	49845	0.2504623088519596	No Hit
TTCAAGTAATCCAGGATAGGCT	47754	0.23995540368976784	No Hit
CTAGACTGAAGCTCCTTGAGG	47665	0.2395081944313101	No Hit
CTGAATGATGATATCCCACTAACTGAGCAGTCAGTA	47288	0.2376138361117758	No Hit
GCATTGGTGGTTCAGTGGTAGAATTCTCGCCT	45903	0.23065445607847326	No Hit

Sequence	Count	Percentage	Possible Source
TAATACTGCCGGGTAATGATGGA	45501	0.22863447718072047	No Hit
GAGAAGACGGTCGAACTTGACTATCT	45424	0.22824756579980762	No Hit
AACTGTGATGAAAGATTTGGTCTGTATGTAAT	45335	0.22780035654134992	No Hit
TTCAAATCGATCTGCGCCTTT	45096	0.22659942381358147	No Hit
ATTCAAATCGATCTGCGCCTT	44933	0.22578037764359712	No Hit
TAGCTTATCAGACTGATGTTGAT	44205	0.22212230640587566	No Hit
TGAGGTAGTAGGTTGTATAGTT	42028	0.21118326645461244	No Hit
GGCTGGTCCGATGGTAGTGGGTTATCAGAACT	40990	0.20596750004698208	No Hit
TAGCTTATCAGACTGATGTTGACT	40787	0.2049474609518482	No Hit
TGGAATGTAAAGAAGTATGTAT	40729	0.2046560212103814	No Hit
CTTAATGATGACTGTTTTTTTTGATTGCTTGAAGCA	40606	0.20403796796554663	No Hit
CACAGATGATGAACTTATTGACGGGCGGACAGAAAC	40051	0.20124919112909684	No Hit
TGAAATGATGGCAAATCATCTTTCGGGACTGACCTG	38705	0.19448577919781512	No Hit
TACCCTGTAGATCCGAATTTGT	38588	0.19389787489175275	No Hit
GATGGGAGACCGCCTGGGAATACCGGGTGCTGTAGG	38435	0.1931290769530558	No Hit
TCAGTGCACTACAGAACTTTGT	38063	0.19125984274916516	No Hit
TAGCTTATCAGACTGATGTTG	37579	0.18882782835485584	No Hit
TAATACTGCCTGGTAATGATGAC	35661	0.1791902175939358	No Hit
GCATATGATGGAAAAGTTTTAATCTCCTGACACTTG	35258	0.17716521387305428	No Hit
TAACACTGTCTGGTAACGATGTT	35196	0.1768536748390725	No Hit
TAGGGTGATGAAAAAGAATCCTTAGGCGTGGTTGTG	35174	0.17674312873024026	No Hit
TACAATGATGATAACATAGTTCAGCAGACTAACGCT	35054	0.17614014995479166	No Hit
CTGCTGTGATGACATTCCAATTAAAGCACGTGTTAG	35019	0.17596428114528584	No Hit
TGGAAGACTAGTGATTTTGTTGT	33976	0.1707233906220118	No Hit
TCTCCTACTTGGATAACTGTGGTAATTCTAGAGCTA	33032	0.16597995758848286	No Hit
GACTCTTAGCGGTGGATCACTCGGCTCGTGCGTCGA	32635	0.16398510280637377	No Hit
AGAAATGAAGAAACTAAAATTGGTCTTAGTATTGAA	32115	0.16137219477942985	No Hit
TGAGGTAGTAGTTTGTACAGTT	31881	0.1601963861673051	No Hit
TACGGGGATGATTTTACGAACTGAACTCTCTCTTTC	31272	0.1571362688819035	No Hit
TAATACTGCCTGGTAATGATGA	30834	0.15493539635151612	No Hit
ACGGCCCTGGCGGAGCGCTGAGAAGACGGTCGAACT	30215	0.15182503083482712	No Hit
GCATTGGTGGTTCAGTGGTAGAATTCTCGCC	30076	0.15112658041993252	No Hit
CGCGACCTCAGATCAGACGG	30027	0.15088036408662434	No Hit
CTGACCTATGAATTGACAGCC	29958	0.1505336512907414	No Hit
TCGCGTGATGACATTCTCCGGAATCGCTGTACGGCC	29207	0.14676000912105897	No Hit
CGCGACCTCAGATCAGACGA	28584	0.14362954431185498	No Hit
TTGCATGATGACTTGAATTGTCGGATACCCCTTCAC	28582	0.1436194946655975	No Hit

Sequence	Count	Percentage	Possible Source
TGTAACAGCAACTCCATGTGGA	28491	0.14316223576088236	No Hit
TCCTACTTGGATAACTGTGGTAATTCTAGAGCTAAT	28350	0.14245373569973024	No Hit
TTCAAATCGAACTGCGCCTTT	27204	0.13669528839419617	No Hit
ATACATGATGATCTCACACAACTTGAACTCTCTCAC	27171	0.1365294692309478	No Hit
TTCAAATCGATCTGCGCCTTTT	26675	0.13403715695909363	No Hit
TCAGATGATGAATTTAACTGTTCAACTGCTGAATGA	25903	0.13015799350370766	No Hit
CTCCATGATGAACACAAAATGACAAGCATATGGCTG	25752	0.1293992452112682	No Hit
TTCCTATGATGAGGACCTTTTCACAGACCTGTACTG	25514	0.12820333730662847	No Hit
AATGGATTTTTGGAGCAGG	25361	0.1274345393679315	No Hit
AGAAGACGGTCGAACTTGACTATCT	24654	0.12388198941591355	No Hit
GCTTAATGATGACTGTTTTTTTTGATTGCTTGAAGC	24635	0.12378651777646753	No Hit
TGGGAGACCGCCTGGGAATACCGGGTGCTGTAGGCT	23946	0.12032441464076686	No Hit
AAGCTATGATGAATTTGATTGCATTGATCGTCTGAC	23918	0.12018371959316217	No Hit
GTGTATGATGACAACTCGGTAATGCTGCATACTCCC	23799	0.11958576564084233	No Hit
CTGCGATGATGGCATTTCTTAGGACACCTTTGGATT	23410	0.11763110944376313	No Hit
TACCCTGTAGATCCGAATTTGTG	23298	0.11706832925334446	No Hit
ATTCAAATCGATCTGCGCCTTA	23255	0.1168522618588087	No Hit
TGTAAACATCCCCGACTGGAAGC	23204	0.11659599587924305	No Hit
AATACATGATGATCTCAATCCAACTTGAACTCTCTC	23150	0.11632465543029119	No Hit
GGCTGGTCCGATGGTAGTGGGTTATCAGAAC	22728	0.11420418006996362	No Hit
AGCAGCATTGTACAGGGCTATGA	22483	0.11297309840342273	No Hit
TGCTATGATGAAGGCTATGTTGGTAGGGACAACTGA	22474	0.1129278749952641	No Hit
TTCACAGTGGCTAAGTTCTGC	22344	0.11227464798852813	No Hit
TCGCGAAGGCCCGCGGCGGGTGTTGACGCGATGTGA	22293	0.11201838200896247	No Hit
CGACTCTTAGCGGTGGATCACTCGGCTCG	22187	0.11148575075731622	No Hit
TCGTACGACTCTTAGCGGTGGATCACTCGGCTCGTG	22091	0.11100336773695735	No Hit
AGTCTGTGATGAATTGCTTTGACTTCTGACACCTCG	22010	0.11059635706352955	No Hit
TGTAAACATCCCCGACTGGAAG	21999	0.11054108400911343	No Hit
TAATACTGTCTGGTAAAACCGT	21981	0.11045063719279614	No Hit
CGACTCTTAGCGGTGGATCACTCGGCTCGTG	21383	0.10744579296181064	No Hit
GTTGAGGTCTATCCCGATGGGGCTTTTCCTGTAGCC	21049	0.10576750203681205	No Hit
TTCAAATCGAACTGCGCCTTTT	20706	0.10404398770365482	No Hit
TGCATATGATGGAAAAGTTTTAATCTCCTGACACTT	20675	0.10388821818666394	No Hit
TTTGCATGATGACTTGAATTGTCGGATACCCCTTCA	20617	0.10359677844519712	No Hit
ACAGATGATGAACTTATTGACGGGCGGACAGAAACT	20475	0.10288325356091628	No Hit
TCAAATGATGAAATCACCCAAAATAGCTGGAATTAC	20461	0.10281290603711396	No Hit
TAAAGTGCTTATAGTGCAGGTAG	20339	0.10219987761540789	No Hit

Sequence	Count	Percentage	Possible Source
CTGACCTATGAATTGACAGCCAT	20272	0.10186321446578242	No Hit

Adapter Content

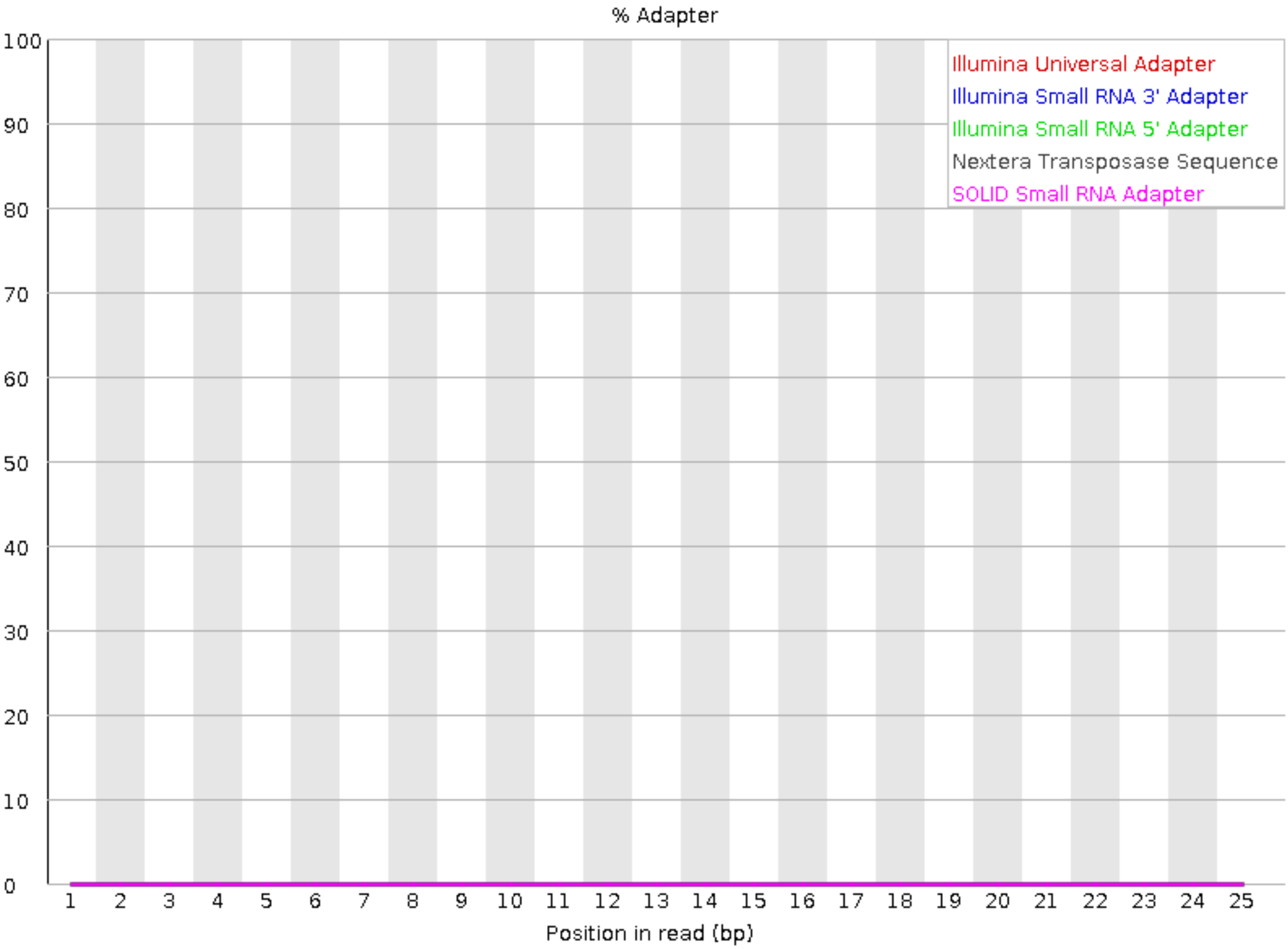

Supplement: Supplementary file 5 [file DataSheet5.zip › QC reports/shCD44_6.fastq.gz FastQC Report.pdf]
