# Supplementary material for: CD44 knockdown alters miRNA expression and their target genes in colon cancer
Source: Front Immunol. 2025 May 14;16:1552665. doi: 10.3389/fimmu.2025.1552665 (PMC12116639; doi:10.3389/fimmu.2025.1552665)
Supplement: Supplementary file 5 [file DataSheet5.zip › QC reports/shLUC_2.fastq.gz FastQC Report.pdf]

FastQC Report

Mon 31 Mar 2025
shLUC_2.fastq.gz

Summary

- Basic Statistics
- Per base sequence quality
- Per tile sequence quality
- Per sequence quality scores
- Per base sequence content
- Per sequence GC content
- Per base N content
- Sequence Length Distribution
- Sequence Duplication Levels
- Overrepresented sequences
- Adapter Content

Basic Statistics

Measure	Value
Filename	shLUC_2.fastq.gz
File type	Conventional base calls
Encoding	Sanger / Illumina 1.9
Total Sequences	26115616
Sequences flagged as poor quality	0
Sequence length	18-36
%GC	51

Per base sequence quality

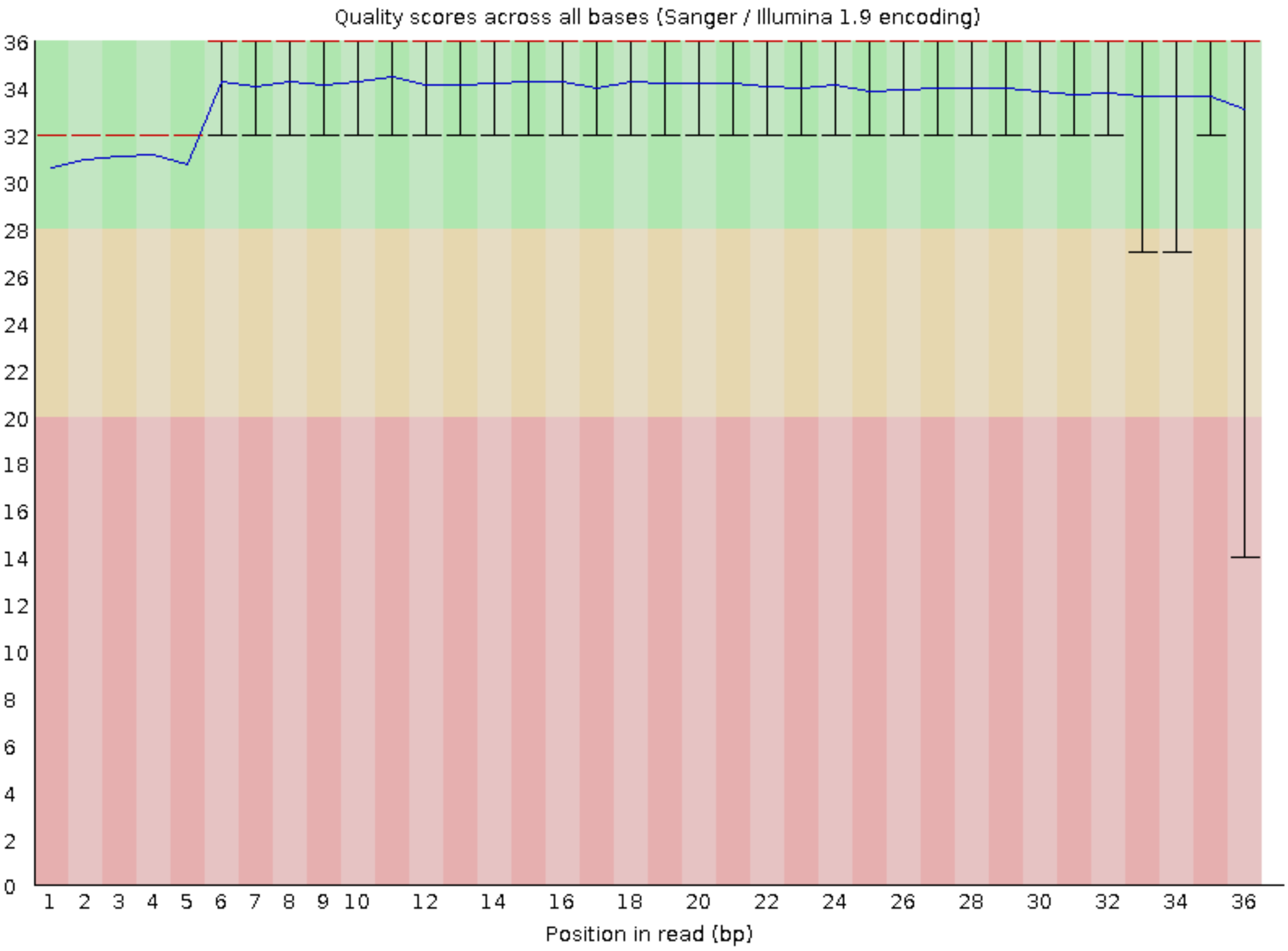

Per tile sequence quality

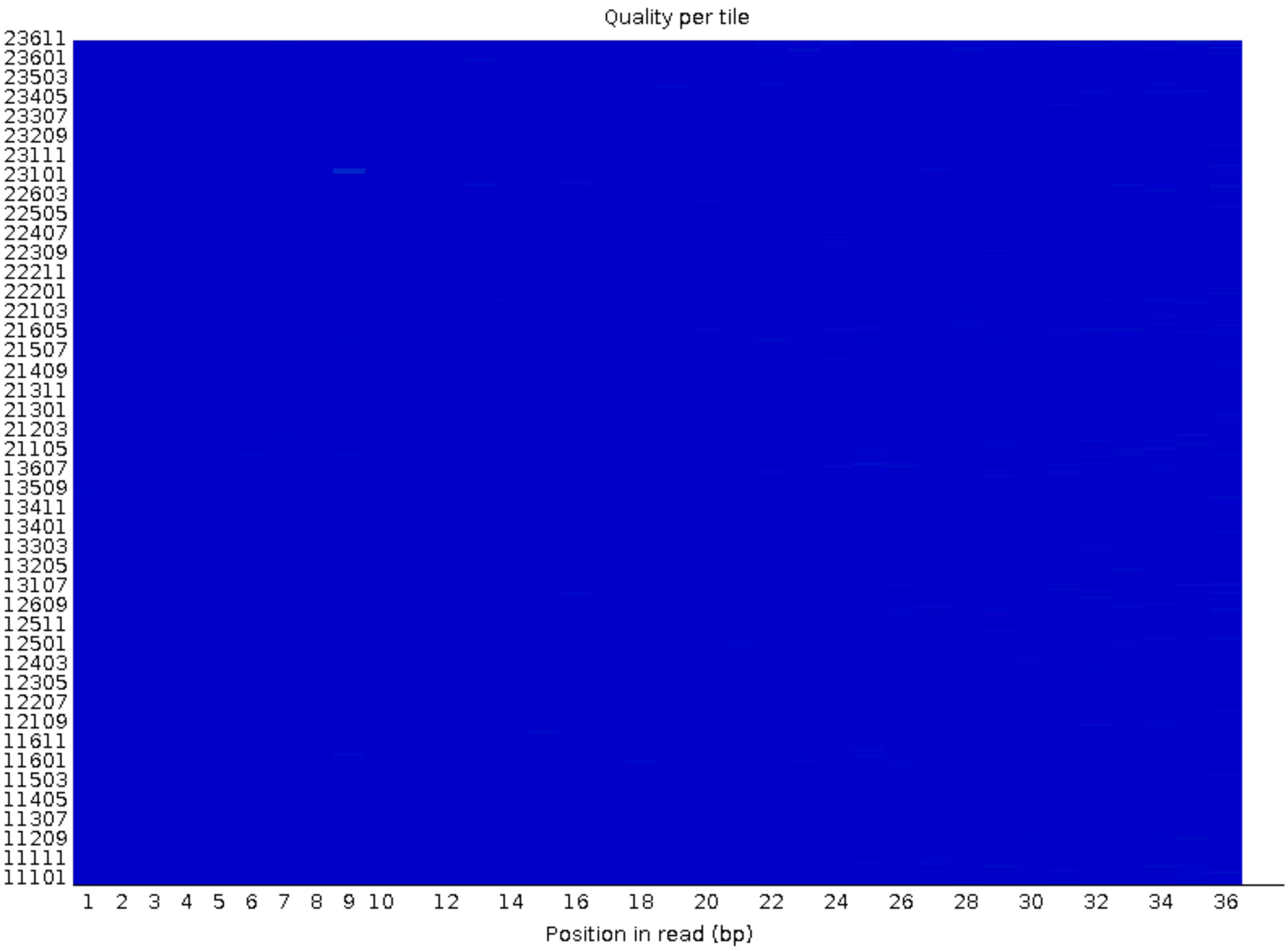

Per sequence quality scores

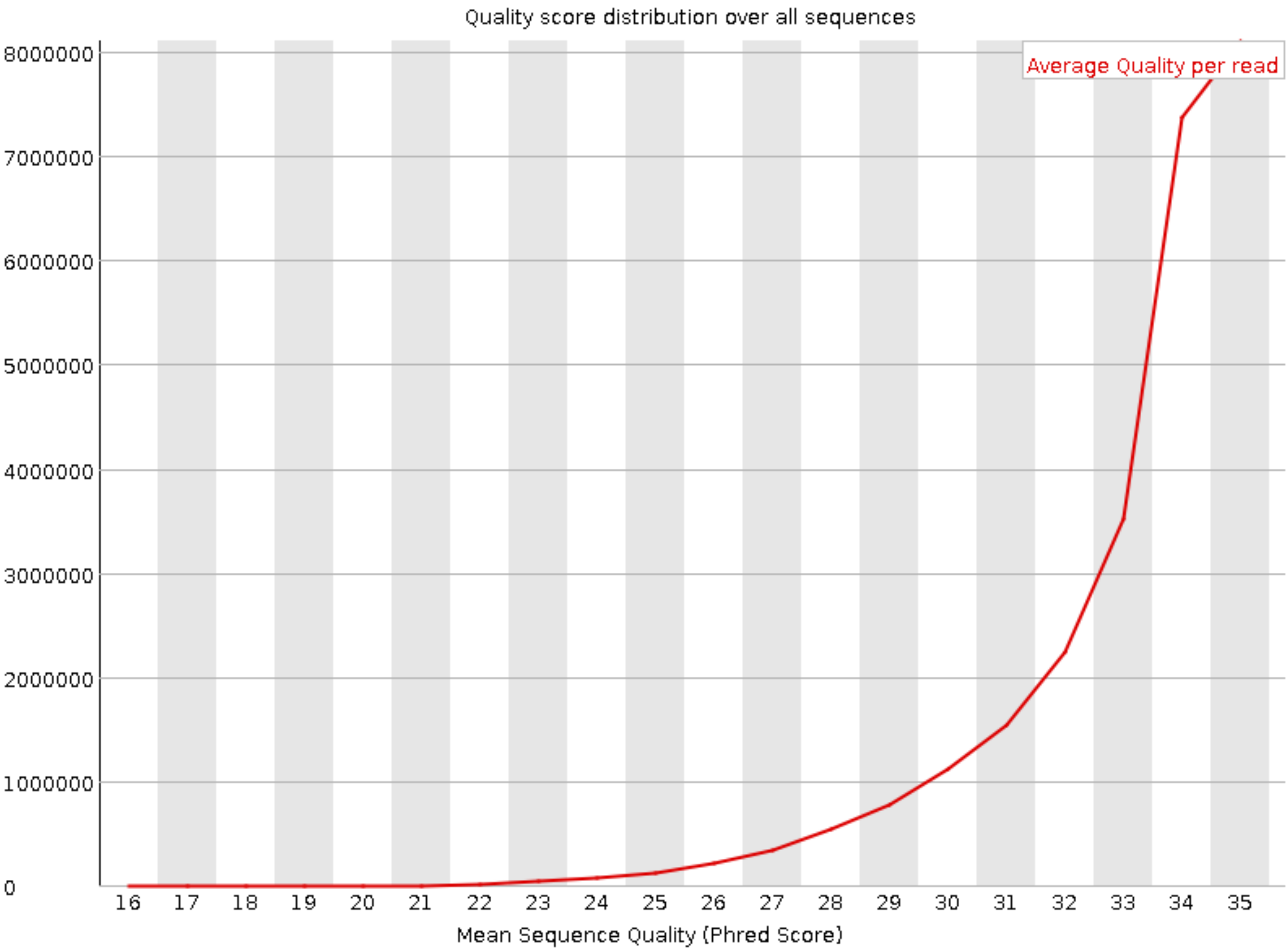

Per base sequence content

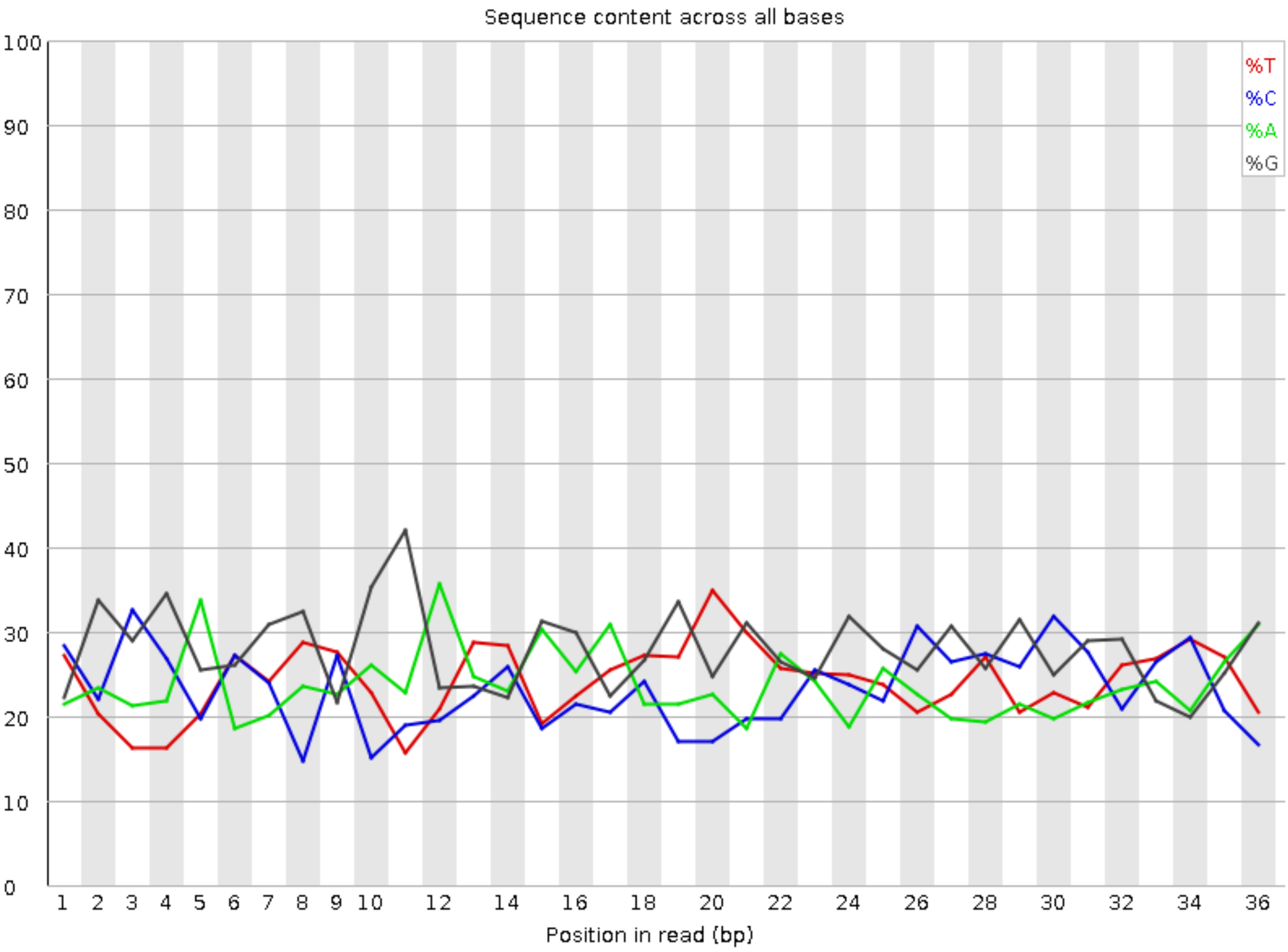

Per sequence GC content

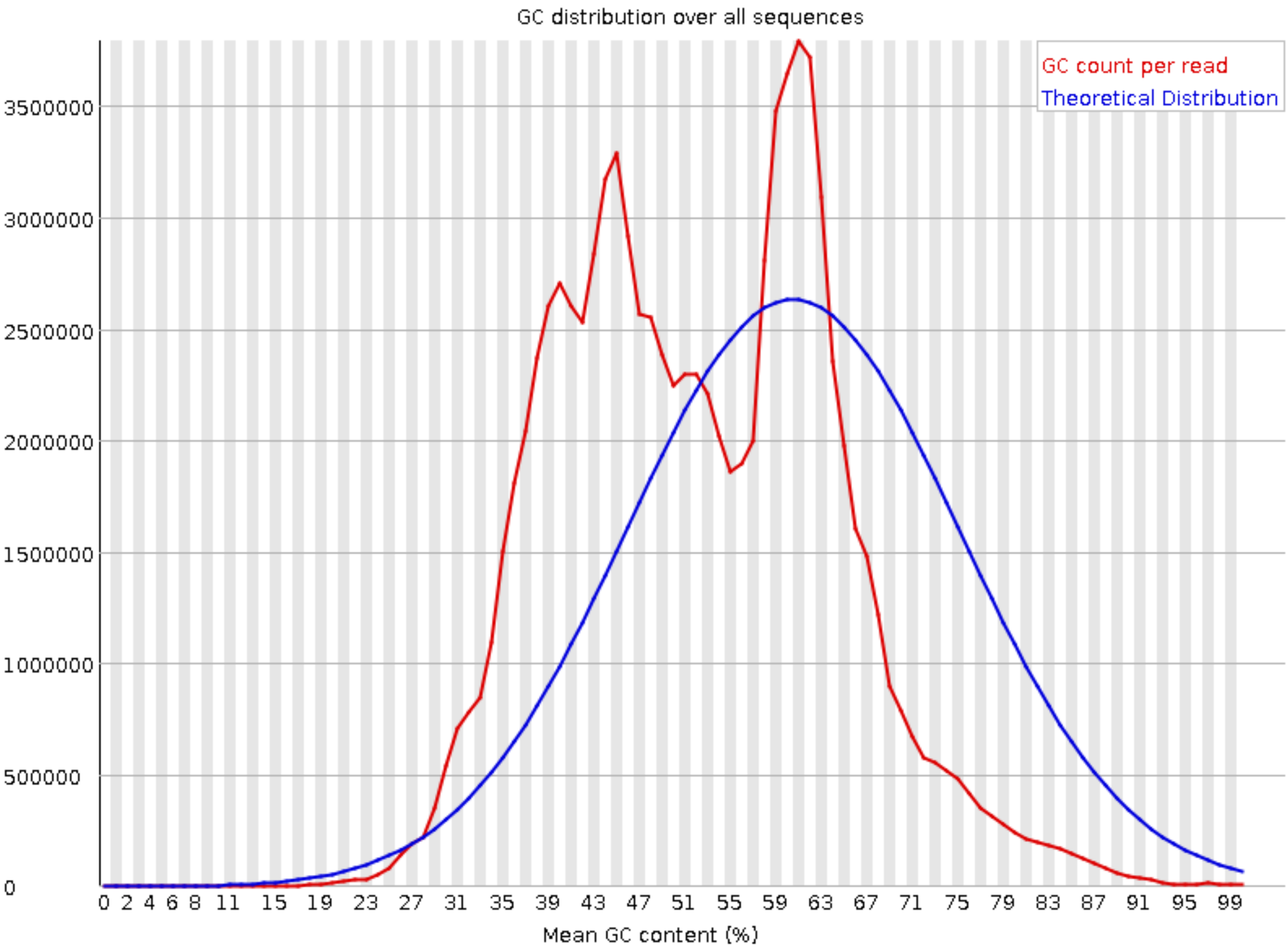

Per base N content

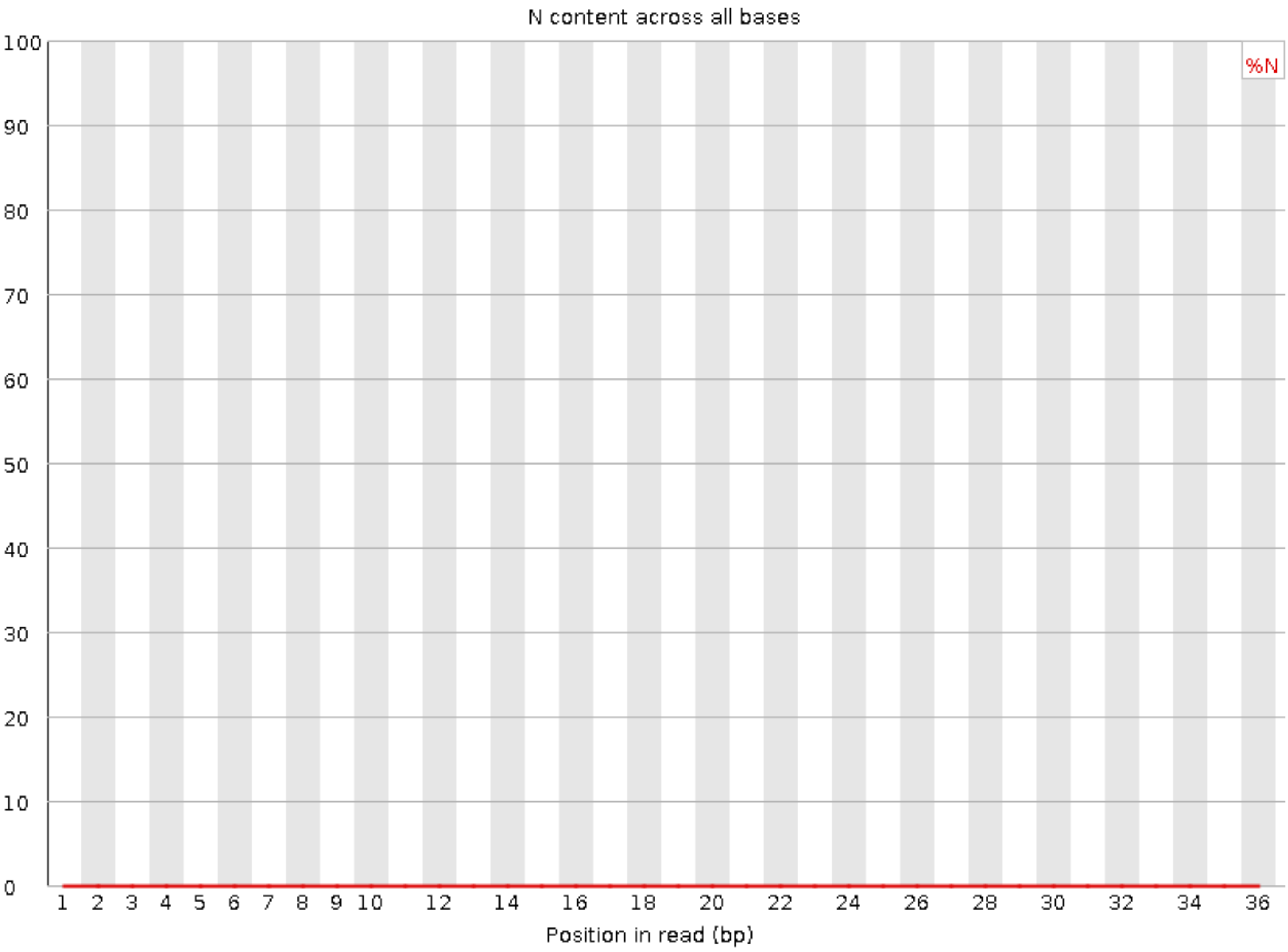

Sequence Length Distribution

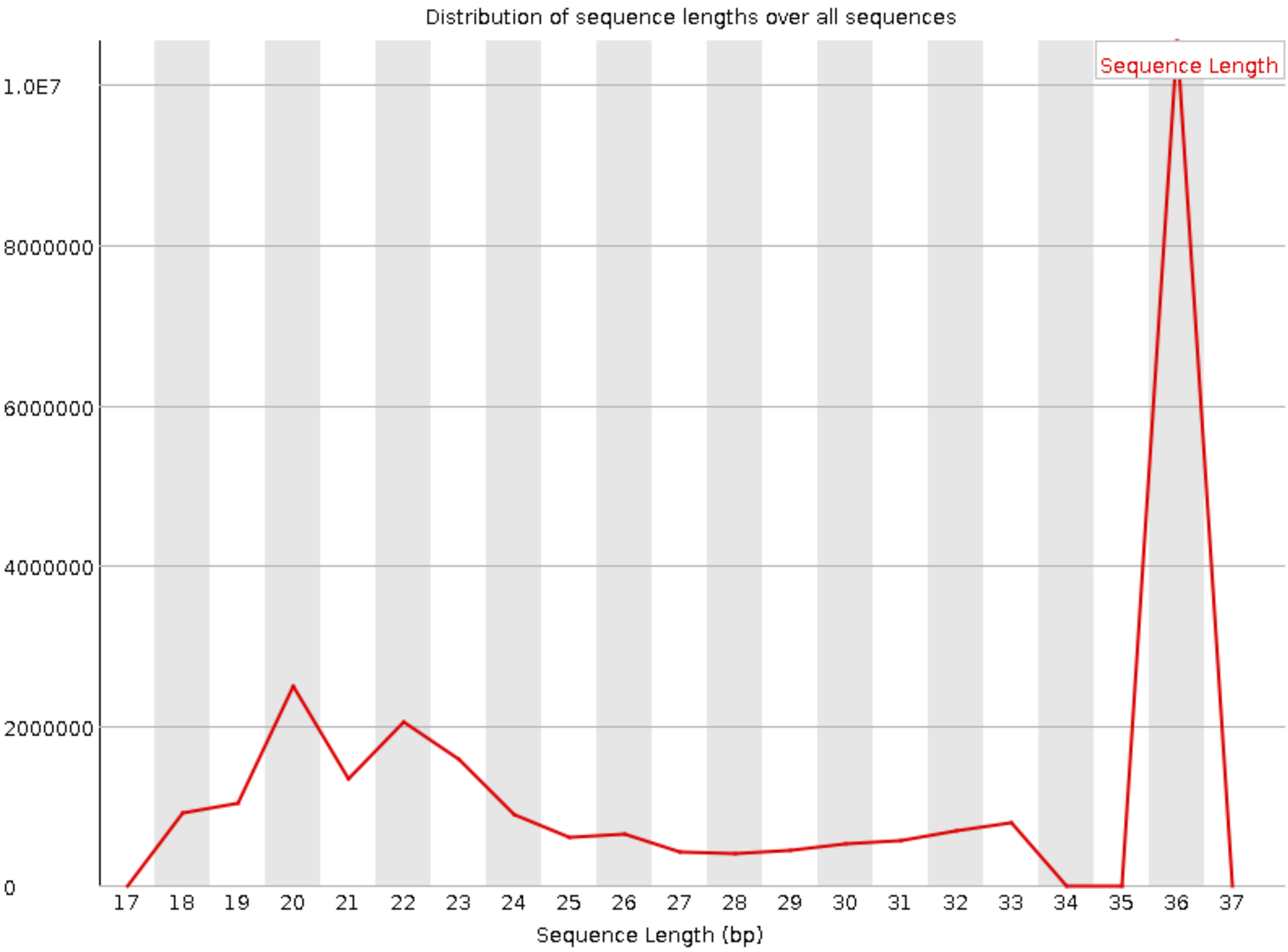

Sequence Duplication Levels

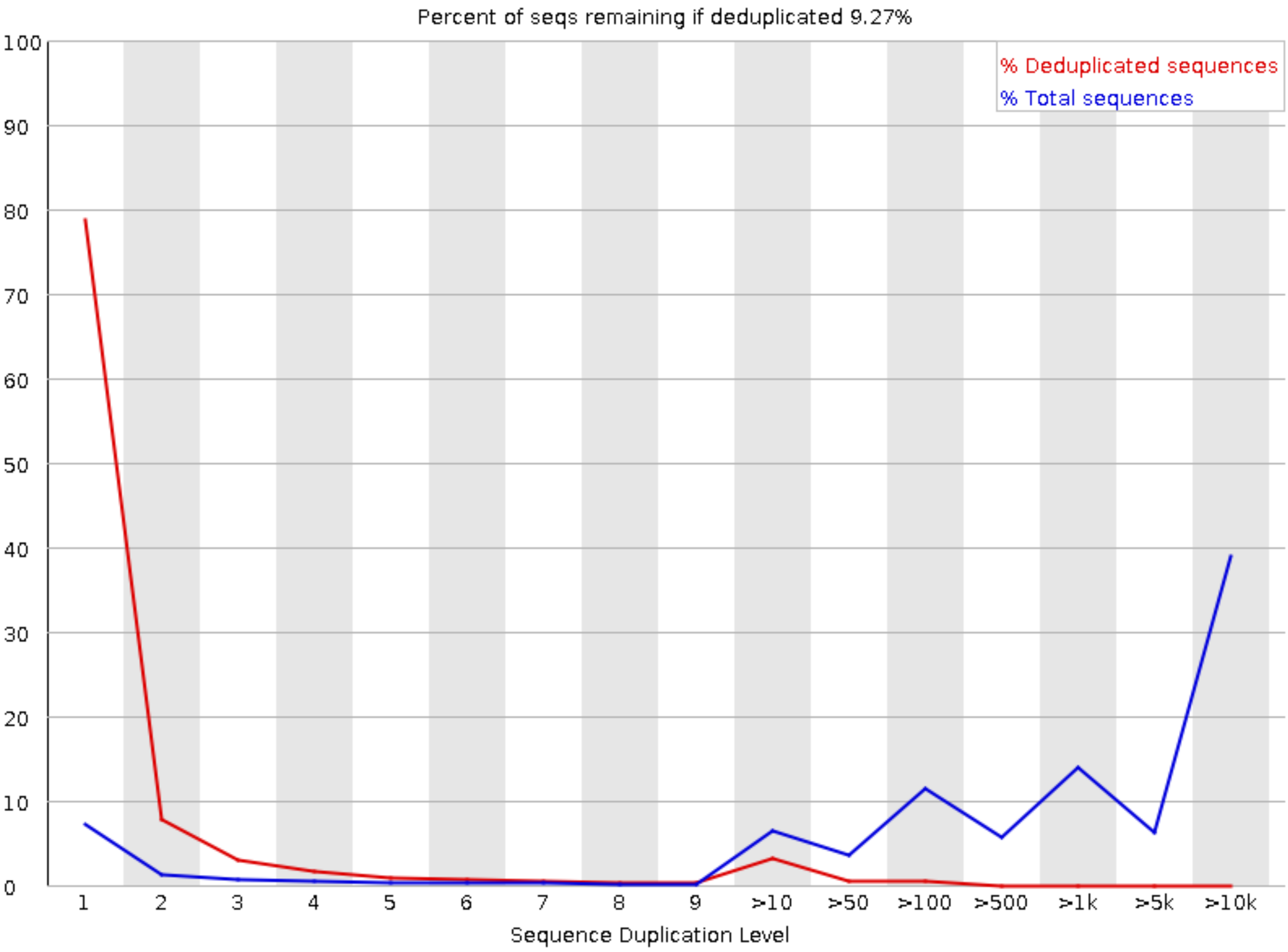

Overrepresented sequences

Sequence	Count	Percentage	Possible Source
CGCGACCTCAGATCAGACGT	1237460	4.73839100712769	No Hit
TGCTCTGATGAAATCACTAATAGGAAGTGCCGTCAG	315515	1.2081468803952393	No Hit
TAGCTTATCAGACTGATGTTGAC	308176	1.1800449202500145	No Hit
GTGAAATGATGGCAATCATCTTTCGGGACTGACCTG	217173	0.831582911925187	No Hit
TAGCTTATCAGACTGATGTTGA	202878	0.776845547123989	No Hit
CGCGACCTCAGATCAGACGTGGCGACCCGCTGAATT	188209	0.7206760889729731	No Hit
GAGAAGACGGTCGAACTTGACTATCT	178240	0.6825035258597767	No Hit
CGCGACCTCAGATCAGACGC	170417	0.6525482684383167	No Hit
GTTTGTGATGACTTACATGGAATCTCGTTCGGCTGA	167780	0.6424508615841189	No Hit
TCGCTGCGATCTATTGAAAGTCAGCCCTCGACACAA	161105	0.6168914415038114	No Hit
CGACTCTTAGCGGTGGATCACTCGGCTCGTGCGTCG	154247	0.59063129125501	No Hit
ACCGGGTGCTGTAGGCTT	153240	0.586775360764992	No Hit

Sequence	Count	Percentage	Possible Source
GCCTCTGATGAAGCCTGTGTTGGTAGGGACATCTGA	152168	0.5826705370457277	No Hit
GATGGGAGACCGCCTGGGAATACCGGGTGCTGTAGG	140199	0.5368397207249487	No Hit
TTGAATGATGACTTTAATTGTCGGATACCCCTTCAC	134037	0.5132446425923861	No Hit
AGTAGTGATGAAATTCCACTTCATTGGTCCGTGTTT	133608	0.5116019472793596	No Hit
ACCGGGTGCTGTAGGCTTT	126059	0.48269587054733837	No Hit
CCTGGATGATGATAAGCAAATGCTGACTGAACATGA	120761	0.4624091578004517	No Hit
CGCGACCTCAGATCAGACG	115556	0.4424785538277175	No Hit
CGCTGCGATCTATTGAAAGTCAGCCCTCGACACAAG	103964	0.39809131823656774	No Hit
GTGCAATGATGTATTTTATTCAACACATCATTCTGA	103084	0.3947216868252313	No Hit
AGAAGACGGTCGAACTTGACTATCT	102255	0.39154734087068827	No Hit
TATCTGTGATGATCTTATCCCGAACCTGAACTTCTG	101829	0.3899161329374731	No Hit
TGGAAGACTAGTGATTTTGTTGTT	93162	0.3567290926624132	No Hit
TTGGTACTAGCAACGCACTTT	91362	0.34983666477558867	No Hit
ATACATGATGATCTCAATCCAACTTGAACTCTCTCA	89312	0.34198695523781636	No Hit
TGGGAGACCGCCTGGGAATACCGGGTGCTGTAGGCT	87514	0.3351021856041994	No Hit
CTCCTACTTGGATAACTGTGGTAATTCTAGAGCTAA	85737	0.32829782762926213	No Hit
TTTCTATGATGAATCAAACTAGCTCACTATGACCGA	83407	0.3193759626424282	No Hit
TAGCTTATCAGACTGATGTTGAT	82412	0.3155659816716558	No Hit
TGAAATGATGGCAATCATCTTTCGGGACTGACCTGA	76209	0.2918139093483378	No Hit
TCTCCTACTTGGATAACTGTGGTAATTCTAGAGCTA	74771	0.2863076252920858	No Hit
CAGGACGGTGGCCATGGAAGTCGGAATCCGCTAAGG	74472	0.28516271643755214	No Hit
CTACGGGGATGATTTTACGAACTGAACTCTCTCTTT	72573	0.2778912050169523	No Hit
TGAGGTAGTAGATTGTATAGTT	71612	0.2742114143507088	No Hit
TGAGGTAGTAGTTTGTGCTGTT	70598	0.27032867997446436	No Hit
CTCGCTGCGATCTATTGAAAGTCAGCCCTCGACACA	69226	0.26507511827406255	No Hit
CGCGACCTCAGATCAGACGA	63797	0.24428678994207909	No Hit
GCATTGGTGGTTCAGTGGTAGAATTCTCGCCT	61306	0.23474843557203476	No Hit
TCAGTGCACTACAGAACTTTGT	59571	0.22810490091445668	No Hit
ACGGCCCTGGCGGAGCGCTGAGAAGACGGTCGAACT	58630	0.224501692780289	No Hit
ACAAATGATGAATAACAAAGGGACTTAATACTG	58052	0.22228845760329757	No Hit
TTTGAATGATGACTTTAATTGTCGGATACCCCTTCA	57381	0.21971911365215357	No Hit
TAGCTTATCAGACTGATGTTGACT	55430	0.21224848764815657	No Hit
GTGAAATGATGGCAAATCATCTTTCGGGACTGACCT	54819	0.20990889129324006	No Hit
CGCGACCTCAGATCAGACGG	53128	0.20343383820622876	No Hit
GCAAATGATGATAAACTGGATCTGACTGACTGTGCT	52365	0.20051221460753596	No Hit
TTCAAGTAATCCAGGATAGGCT	52189	0.19983828832526868	No Hit
TAGCTTATCAGACTGATGTTG	47579	0.1821860146817904	No Hit

Sequence	Count	Percentage	Possible Source
TGAAATGATGGCAAATCATCTTTCGGGACTGACCTG	45780	0.1752974159215697	No Hit
TGCCTCTGATGAAGCCTGTGTTGGTAGGGACATCTG	45662	0.1748455789823223	No Hit
TAGCTTATCAGACTGATGTTGACA	44361	0.16986388527078972	No Hit
TACCCTGTAGATCCGAATTTGT	43917	0.16816375305870634	No Hit
CTGCAGTGATGACTTTCTTAGGACACCTTTGGATTT	43865	0.16796463847530918	No Hit
TAACACTGTCTGGTAACGATGTT	43013	0.1647022226088789	No Hit
CTGGATGATGATAAGCAAATGCTGACTGAACATGAA	42006	0.160846292118861	No Hit
GGCTGGTCCGATGGTAGTGGGTTATCAGAACT	41590	0.15925337545168378	No Hit
ACTCCATGATGAACACAAAATGACAAGCATATGGCT	41205	0.1577791617092241	No Hit
GACTCTTAGCGGTGGATCACTCGGCTCGTGCGTCGA	41024	0.15708608979393784	No Hit
CTAGACTGAAGCTCCTTGAGG	40654	0.15566931295053502	No Hit
CACAGATGATGAACTTATTGACGGGCGGACAGAAAC	39936	0.15292000004901282	No Hit
CGGCCCTGGCGGAGCGCTGAGAAGACGGTCGAACTT	39912	0.15282810101052183	No Hit
AATGGATTTTTGGAGCAGG	39661	0.15186699023297018	No Hit
CGACTCTTAGCGGTGGATCACTCGGCTCG	37245	0.1426158203582102	No Hit
GCAGCTGATGATACAGCTTCTTTCCCCATC	37212	0.14248945918028508	No Hit
GGGAGACCGCCTGGGAATACCGGGTGCTGTAGGCTT	36975	0.14158195617518654	No Hit
TCCTACTTGGATAACTGTGGTAATTCTAGAGCTAAT	36325	0.13909302388272213	No Hit
ATACATGATGATCTCACACAACTTGAACTCTCTCAC	36282	0.1389283714387591	No Hit
TCGCGAAGGCCCGCGGCGGGTGTTGACGCGATGTGA	35331	0.13528687203855347	No Hit
CTGAATGATGATATCCCACTAACTGAGCAGTCAGTA	35176	0.13469335741496583	No Hit
GCCGCCGGTGAAATACCACTACTCTGATCGTTTTTT	34616	0.13254904651684266	No Hit
TTGGTACTAGCAACGCACTTTT	34227	0.1310595162679678	No Hit
TCGTACGACTCTTAGCGGTGGATCACTCGGCTCGTG	33662	0.12889605973682566	No Hit
TGAGGTAGTAGTTTGTACAGTT	33606	0.12868162864701335	No Hit
AGCGCTGAGAAGACGGTCGAACTTGACTATCT	33057	0.12657943814153189	No Hit
TCCCTGGTGGTCTAGTGGTTAGGATTCGGCGCT	32380	0.1239871194307651	No Hit
CTCACTGATGAGTACGTTCTGACTTTCGTTCTTCTG	32207	0.12332468052830918	No Hit
TTGCATGATGACTTGAATTGTCGGATACCCCTTCAC	30994	0.11867994995791023	No Hit
AGACGTGGCGACCCGCTGAATTT	30989	0.11866080432489129	No Hit
CTGACCTATGAATTGACAGCC	29992	0.11484316510091126	No Hit
TAGGGTGATGAAAAAGAATCCTTAGGCGTGGTTGTG	29522	0.11304347559712932	No Hit
GCATTGGTGGTTCAGTGGTAGAATTCTCGCC	29250	0.11200195316089806	No Hit
TGAGGTAGTAGGTTGTATAGTT	28647	0.10969298981881184	No Hit
GAGAAGACGGTCGAACTTGACTATCC	28453	0.10895013925767633	No Hit
GACGTGGCGACCCGCTGAATTT	28404	0.10876251205409054	No Hit
AACTGTGATGAAAGATTTGGTCTGTATGTAAT	28357	0.10858254310371236	No Hit

Sequence	Count	Percentage	Possible Source
TAATACTGCCTGGTAATGATGAC	27972	0.10710832936125267	No Hit
CGCGACCTCAGATCAGACGTGGCGACCCGCTGAATA	27782	0.1063807953065323	No Hit
GAGAAGACGGTCGAACTTGACTATCTAGAGGAAGTA	26662	0.10209217351028596	No Hit
GCAGCCGACTTAGAACTGGTGCGGACCAGGGGAATC	26604	0.10187008416726606	No Hit

Adapter Content

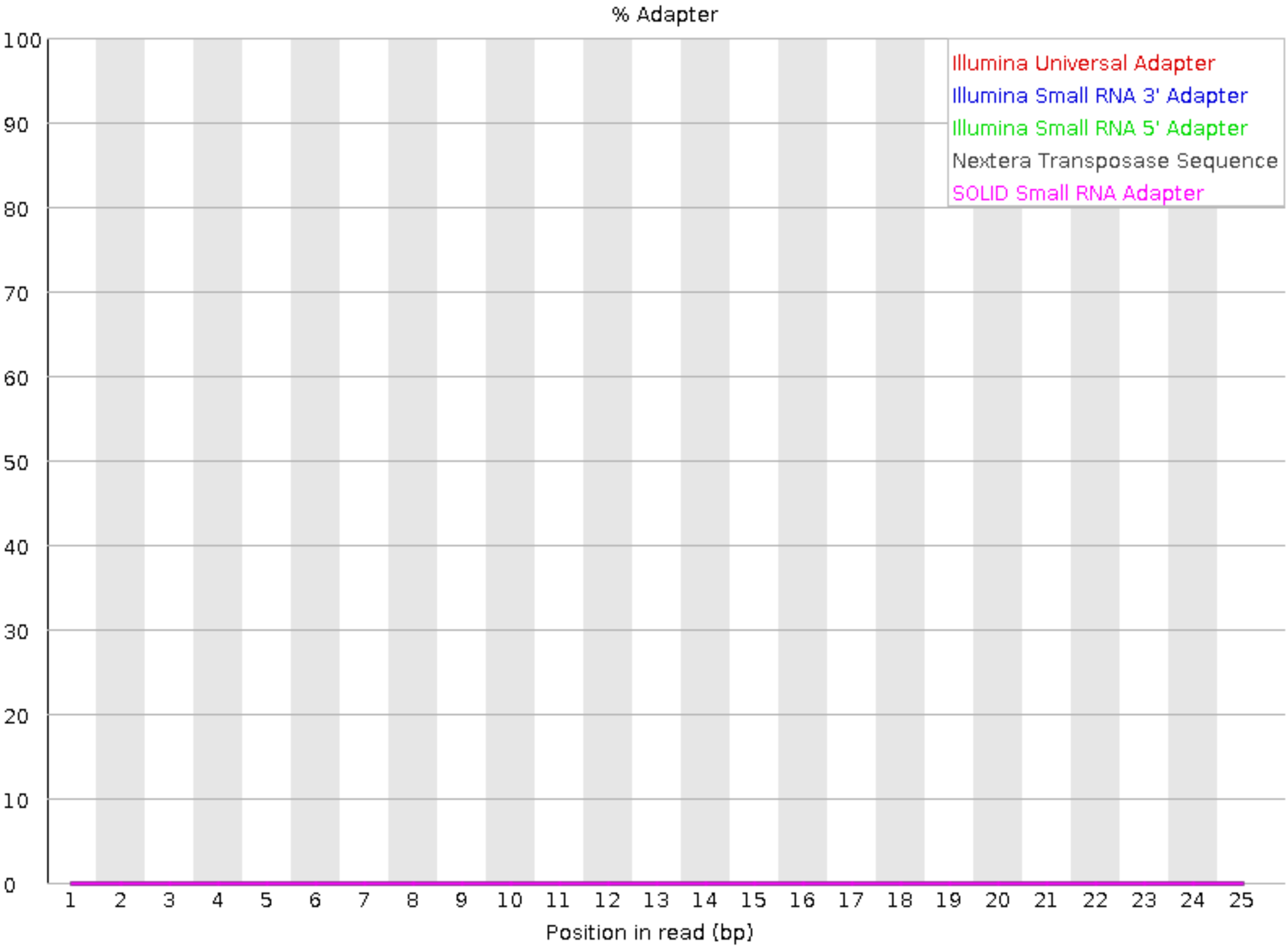

Produced by FastQC (version 0.11.9)
