# Supplementary material for: CD44 knockdown alters miRNA expression and their target genes in colon cancer
Source: Front Immunol. 2025 May 14;16:1552665. doi: 10.3389/fimmu.2025.1552665 (PMC12116639; doi:10.3389/fimmu.2025.1552665)
Supplement: Supplementary file 5 [file DataSheet5.zip › QC reports/shCD44_7.fastq.gz FastQC Report.pdf]

FastQC Report

Mon 31 Mar 2025
shCD44_7.fastq.gz

Summary

- Basic Statistics
- Per base sequence quality
- Per tile sequence quality
- Per sequence quality scores
- Per base sequence content
- Per sequence GC content
- Per base N content
- Sequence Length Distribution
- Sequence Duplication Levels
- Overrepresented sequences
- Adapter Content

Basic Statistics

Measure	Value
Filename	shCD44_7.fastq.gz
File type	Conventional base calls
Encoding	Sanger / Illumina 1.9
Total Sequences	28808107
Sequences flagged as poor quality	0
Sequence length	18-36
%GC	48

Per base sequence quality

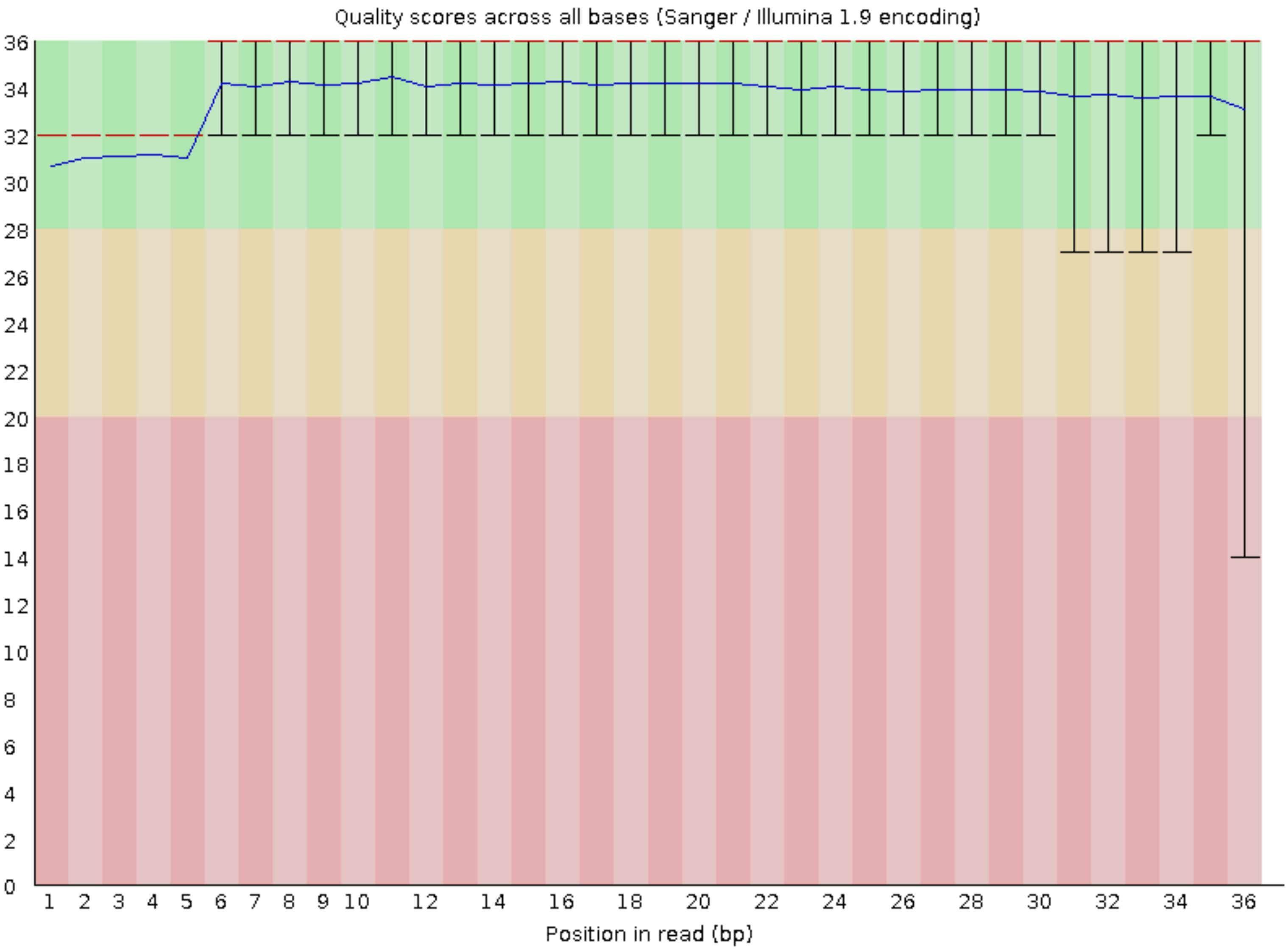

Per tile sequence quality

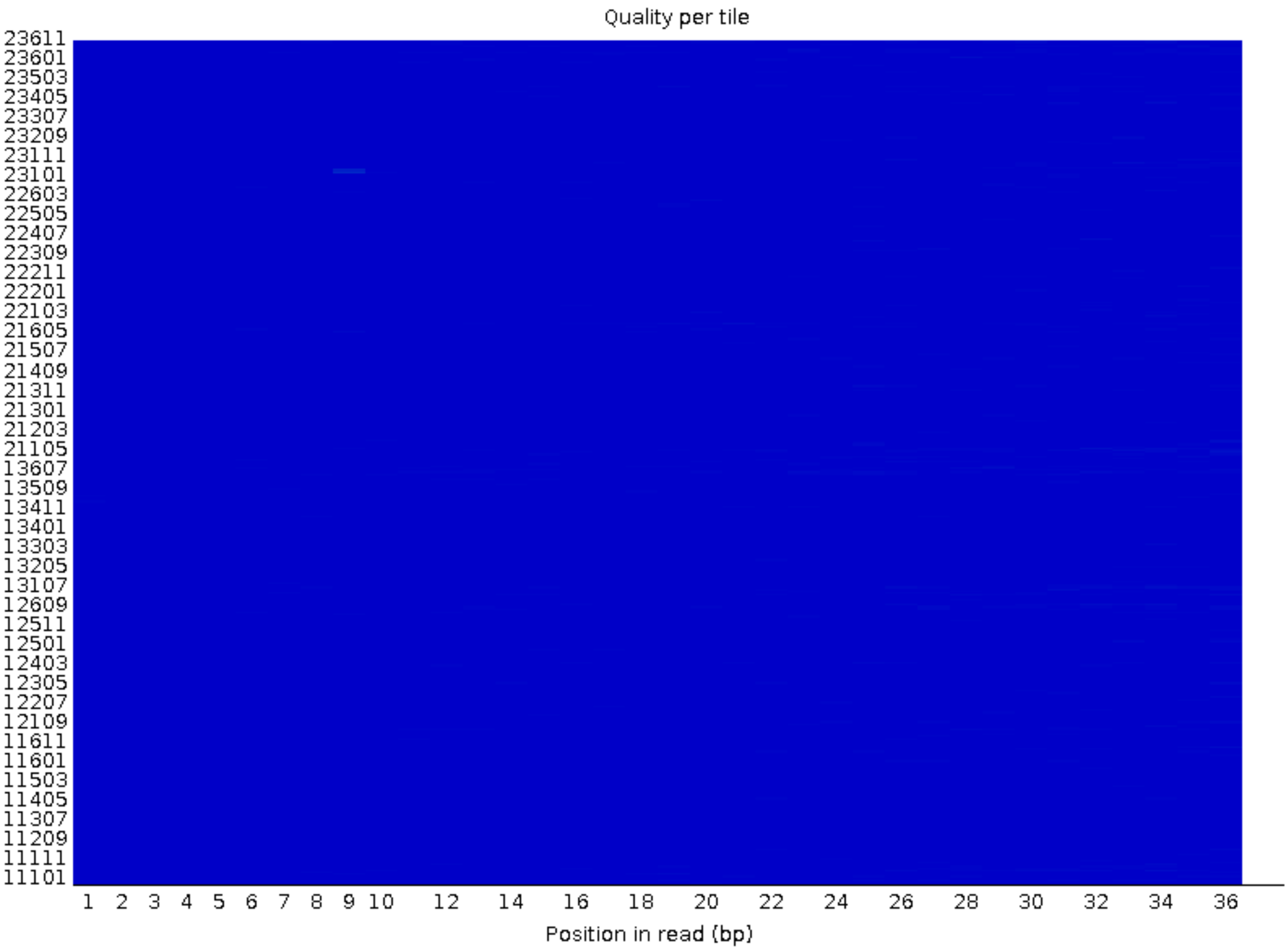

Per sequence quality scores

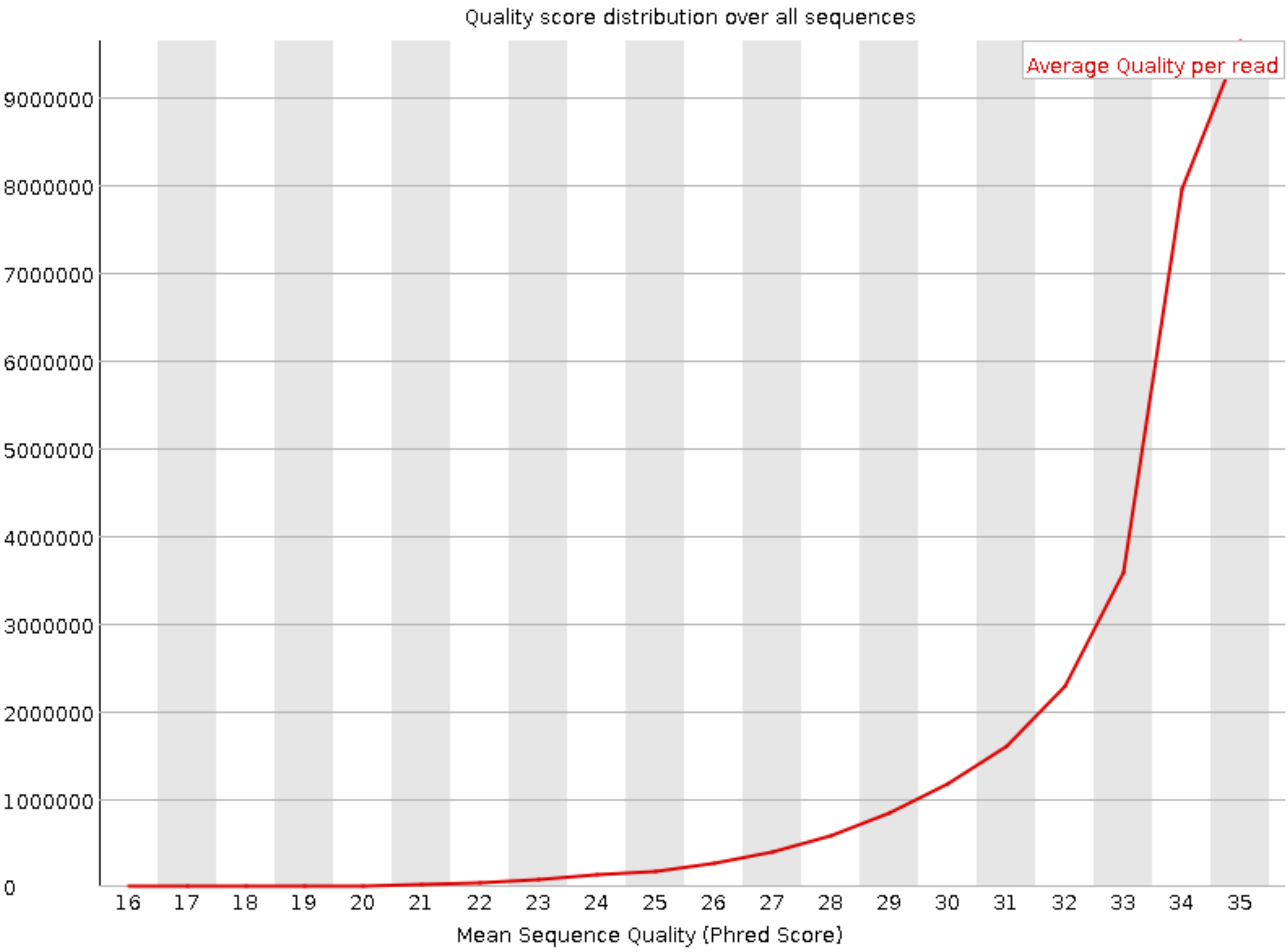

Per base sequence content

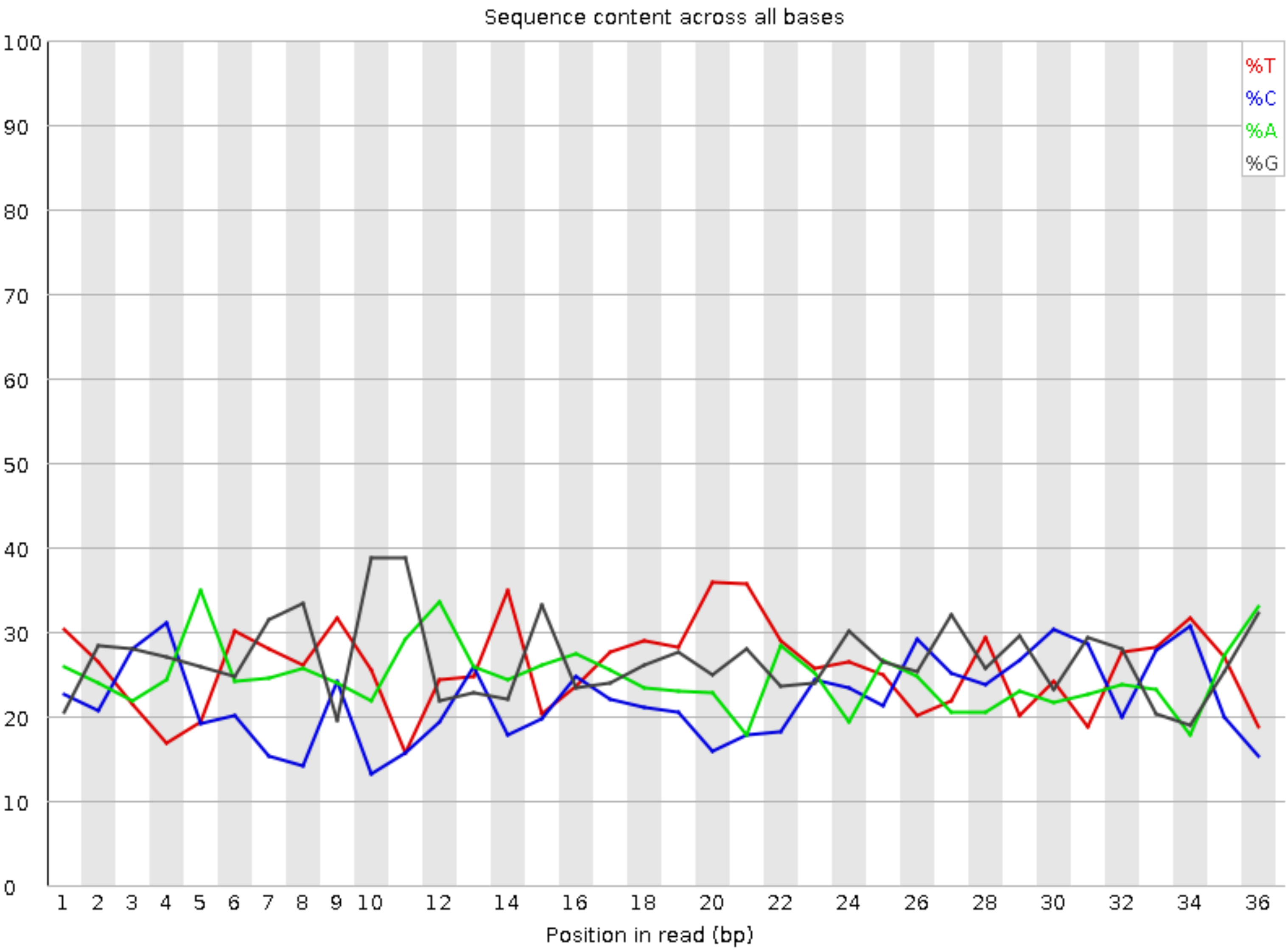

Per sequence GC content

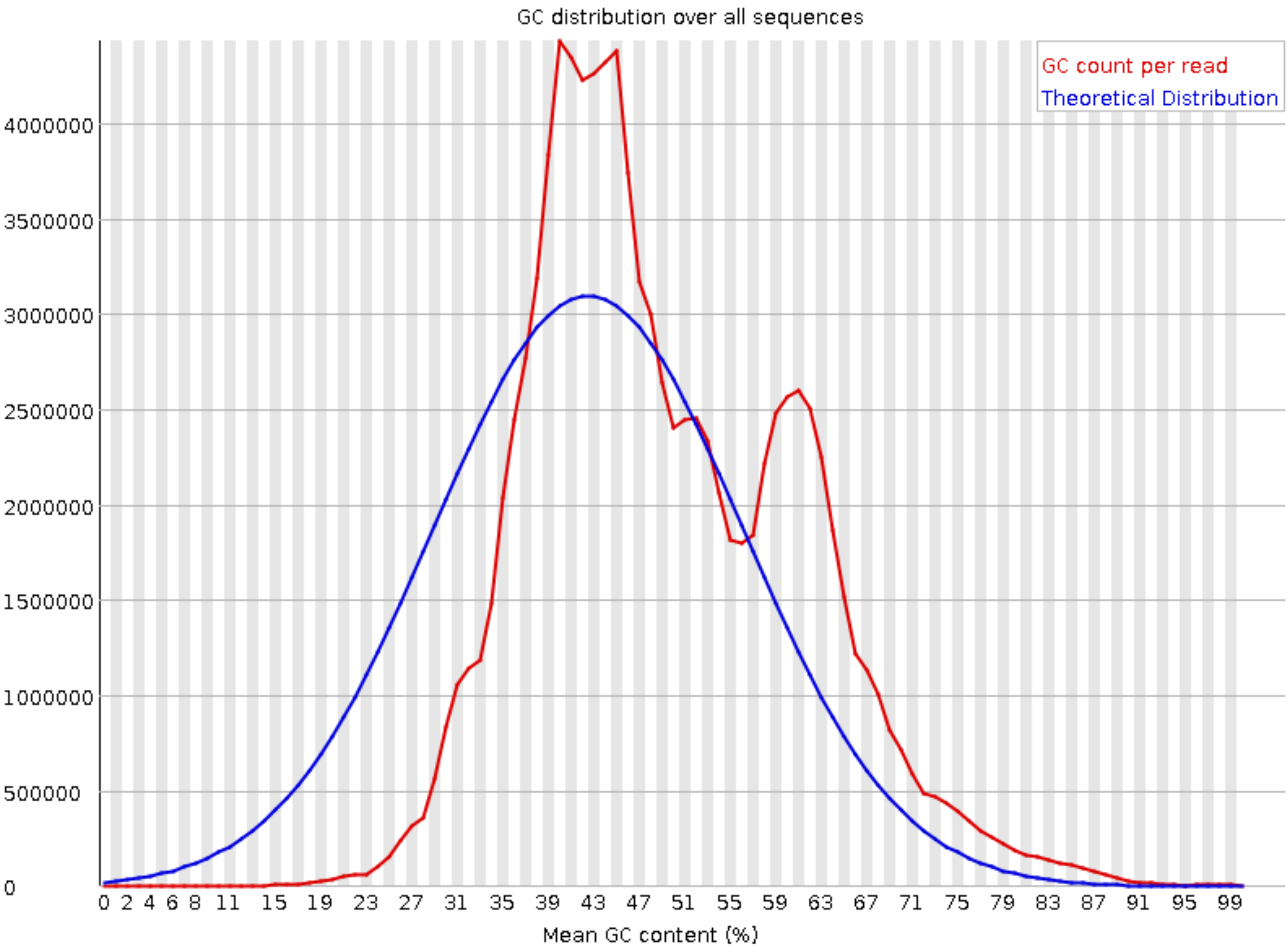

Per base N content

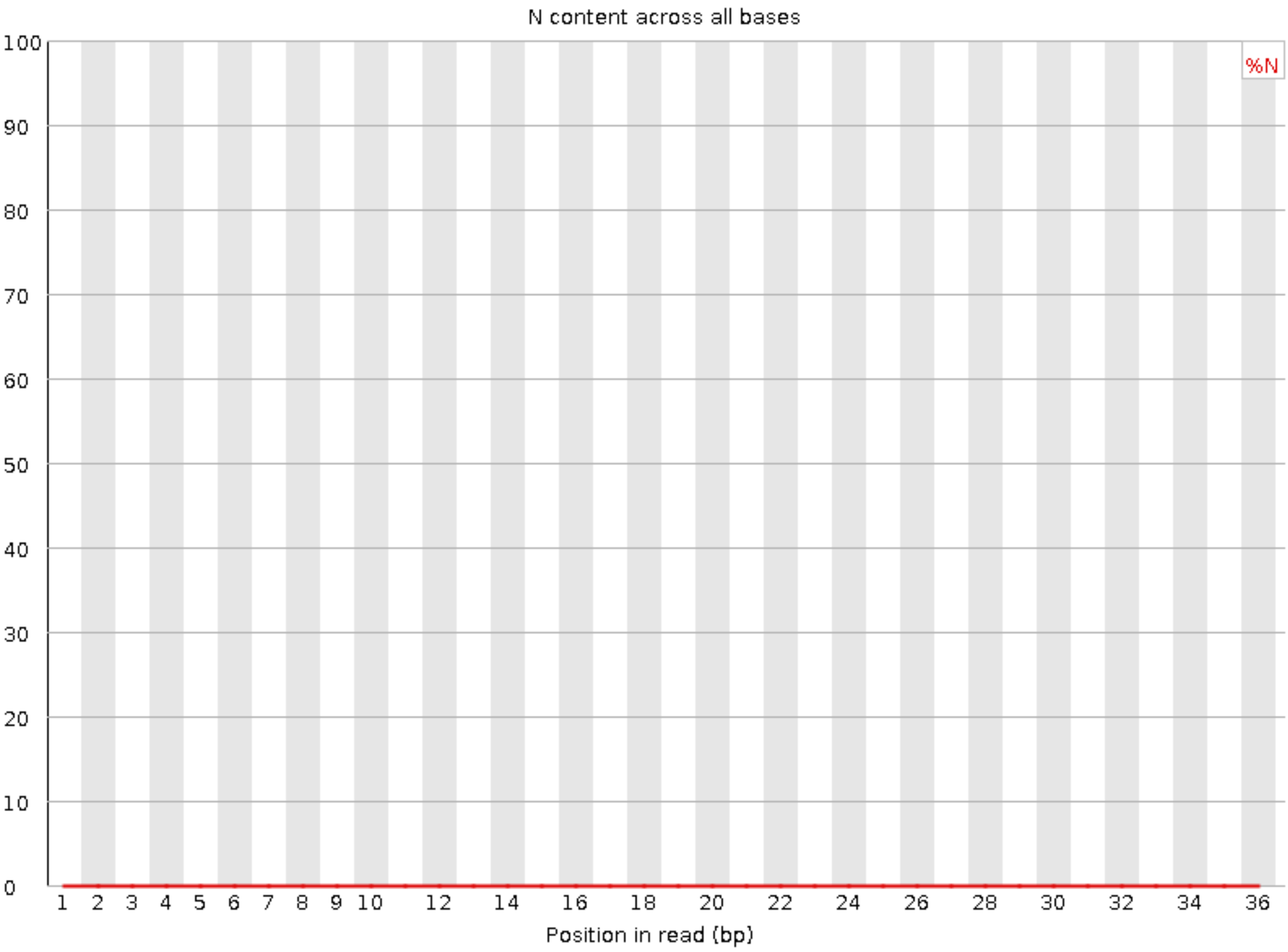

Sequence Length Distribution

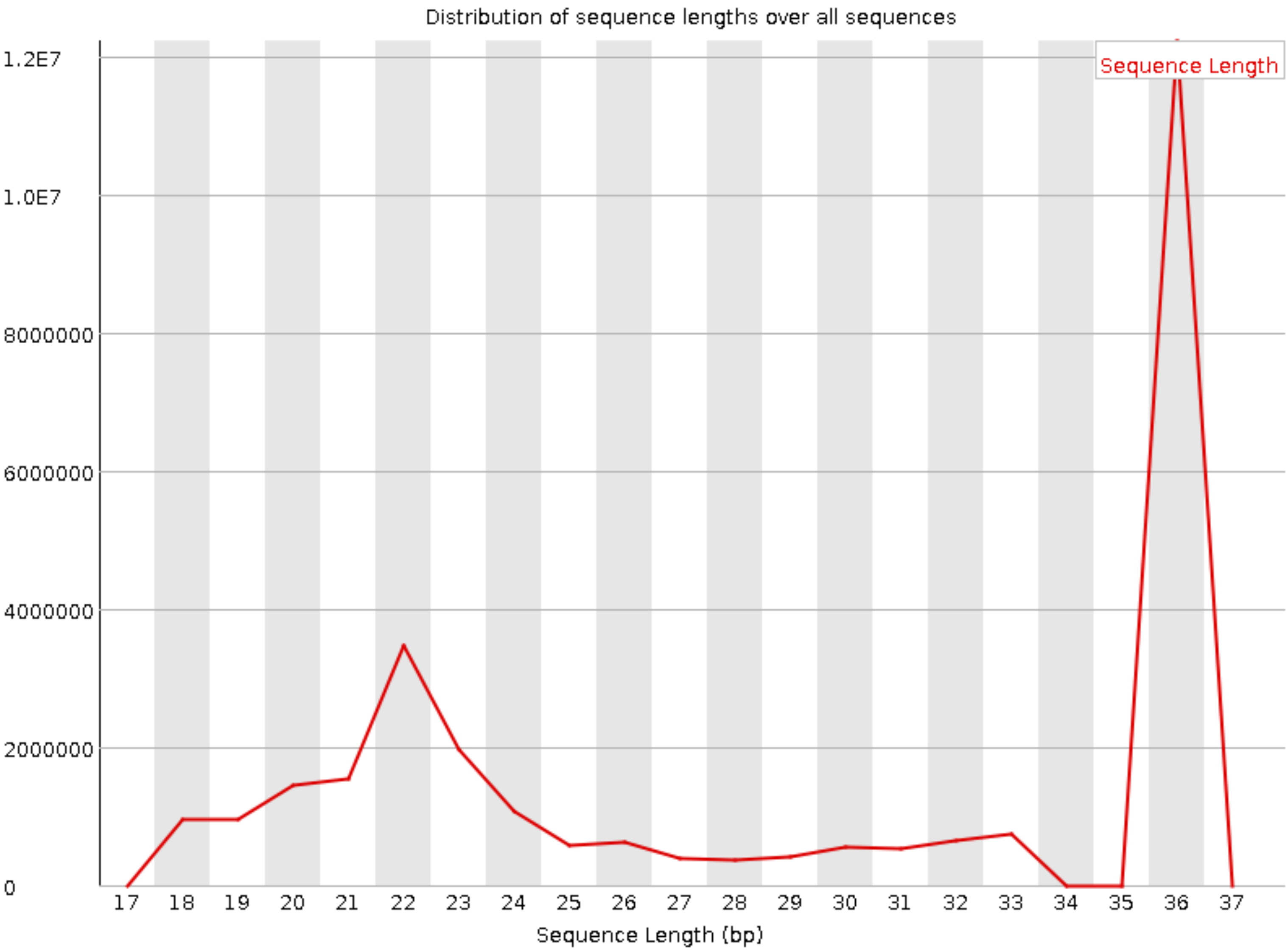

Sequence Duplication Levels

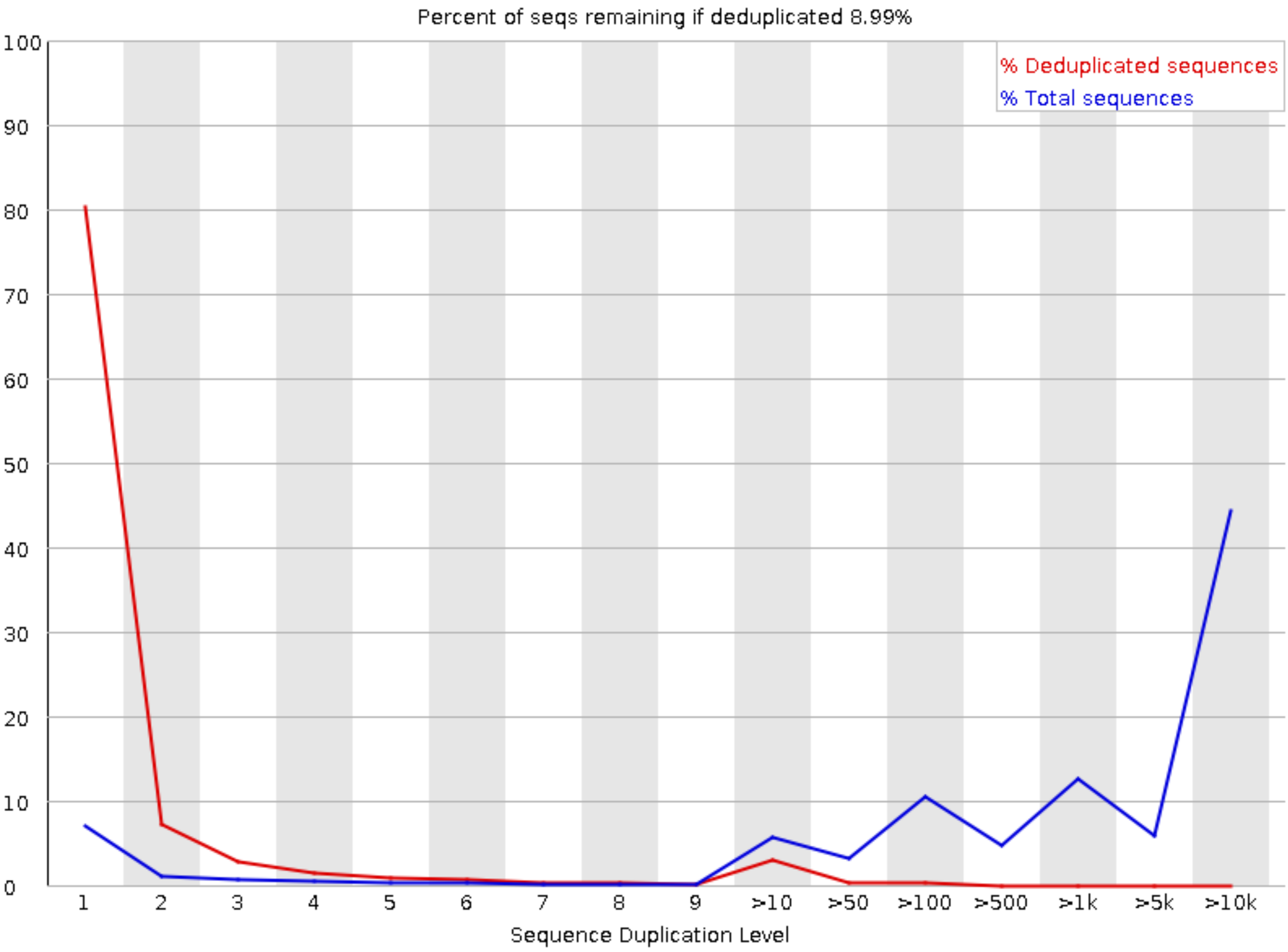

Overrepresented sequences

Sequence	Count	Percentage	Possible Source
ATTCAAATCGATCTGCGCCTTT	540618	1.8766175785170474	No Hit
TGCTCTGATGAAATCACTAATAGGAAGTGCCGTCAG	530703	1.8422001834414181	No Hit
CGCGACCTCAGATCAGACGT	436509	1.5152297233553043	No Hit
TAGCTTATCAGACTGATGTTGAC	419484	1.456131775683838	No Hit
GTGAAATGATGGCAATCATCTTTCGGGACTGACCTG	332725	1.1549700228480821	No Hit
TAGCTTATCAGACTGATGTTGA	248465	0.8624829114943234	No Hit
GTTTGTGATGACTTACATGGAATCTCGTTCGGCTGA	239854	0.832592020017143	No Hit
AGTAGTGATGAAATTCCACTTCATTGGTCCGTGTTT	214954	0.746158017255351	No Hit
CCTGGATGATGATAAGCAAATGCTGACTGAACATGA	209168	0.7260733931597796	No Hit
GAGAAGACGGTCGAACTTGACTATCT	198306	0.6883687289831296	No Hit
GCCTCTGATGAAGCCTGTGTTGGTAGGGACATCTGA	187618	0.6512680614522849	No Hit
ACCGGGTGCTGTAGGCTT	185984	0.6455960469738605	No Hit

Sequence	Count	Percentage	Possible Source
TATCTGTGATGATCTTATCCCGAACCTGAACTTCTG	182799	0.6345401313595509	No Hit
TCGCTGCGATCTATTGAAAGTCAGCCCTCGACACAA	180860	0.6278093871284218	No Hit
GTGCAATGATGTATTTTATTCAACACATCATTCTGA	180602	0.6269138058949865	No Hit
ATTCAAATCGAACTGCGCCTTT	172209	0.5977796458476081	No Hit
CGACTCTTAGCGGTGGATCACTCGGCTCGTGCGTCG	168900	0.586293295842035	No Hit
ACCGGGTGCTGTAGGCTTT	153004	0.5311143838781216	No Hit
TTGAATGATGACTTTAATTGTCGGATACCCCTTCAC	147813	0.5130951506115969	No Hit
ATACATGATGATCTCAATCCAACTTGAACTCTCTCA	146355	0.5080340752691594	No Hit
TGGAAGACTAGTGATTTTGTTGTT	145387	0.5046739100212312	No Hit
CTCCTACTTGGATAACTGTGGTAATTCTAGAGCTAA	134263	0.46605977963078243	No Hit
TTTCTATGATGAATCAAACTAGCTCACTATGACCGA	124181	0.4310626866249837	No Hit
AGAAGACGGTCGAACTTGACTATCT	110884	0.38490554065215044	No Hit
CGCTGCGATCTATTGAAAGTCAGCCCTCGACACAAG	101458	0.35218558442593956	No Hit
GATGGGAGACCGCCTGGGAATACCGGGTGCTGTAGG	101196	0.3512761182121408	No Hit
TGAGGTAGTAGATTGTATAGTT	100682	0.34949189823545157	No Hit
TGAAATGATGGCAATCATCTTTCGGGACTGACCTGA	96891	0.336332408096096	No Hit
CTACGGGGATGATTTTACGAACTGAACTCTCTCTTT	94861	0.3292857805617009	No Hit
CAGGACGGTGGCCATGGAAGTCGGAATCCGCTAAGG	89543	0.3108256991686403	No Hit
GCAAATGATGATAAACTGGATCTGACTGACTGTGCT	88477	0.3071253519018101	No Hit
CTCGCTGCGATCTATTGAAAGTCAGCCCTCGACACA	88393	0.30683376731418	No Hit
AATGGATTTTTGGAGCAGG	85324	0.2961805161304073	No Hit
TGAGGTAGTAGTTTGTGCTGTT	84488	0.2932785552344692	No Hit
TGCCTCTGATGAAGCCTGTGTTGGTAGGGACATCTG	83035	0.28823483611748596	No Hit
TAGCTTATCAGACTGATGTTGACA	80831	0.28058421193728555	No Hit
CGCGACCTCAGATCAGACGTGGCGACCCGCTGAATT	79007	0.2742526608916025	No Hit
TAGCTTATCAGACTGATGTTGAT	77729	0.26981640966551534	No Hit
ATTCAAATCGATCTGCGCCTTC	73161	0.25395976209058096	No Hit
TAGCTTATCAGACTGATGTTGACT	72744	0.25251225288770274	No Hit
TGGGAGACCGCCTGGGAATACCGGGTGCTGTAGGCT	72039	0.2500650250986641	No Hit
CTGGATGATGATAAGCAAATGCTGACTGAACATGAA	70675	0.24533024679476512	No Hit
ACAAATGATGAATAACAAAGGGACTTAATACTG	70026	0.24307740873081318	No Hit
CTAGACTGAAGCTCCTTGAGG	68514	0.23782888615347061	No Hit
TCTCCTACTTGGATAACTGTGGTAATTCTAGAGCTA	66611	0.23122310674561158	No Hit
TACCCTGTAGATCCGAATTTGT	62820	0.218063616606256	No Hit
TTTGAATGATGACTTTAATTGTCGGATACCCCTTCA	62662	0.2175151598819041	No Hit
CTGCAGTGATGACTTTCTTAGGACACCTTTGGATTT	62438	0.216737600981557	No Hit
GTGAAATGATGGCAAATCATCTTTCGGGACTGACCT	62198	0.21590450215975662	No Hit

Sequence	Count	Percentage	Possible Source
TCCTACTTGGATAACTGTGGTAATTCTAGAGCTAAT	62078	0.21548795274885643	No Hit
TTCAAGTAATCCAGGATAGGCT	59985	0.20822263677373873	No Hit
TCAGTGCACTACAGAACTTTGT	58954	0.2046437830850878	No Hit
GGCTGGTCCGATGGTAGTGGGTTATCAGAACT	57513	0.19964171890919455	No Hit
ACTCCATGATGAACACAAAATGACAAGCATATGGCT	54960	0.19077963019229274	No Hit
CACAGATGATGAACTTATTGACGGGCGGACAGAAAC	54855	0.1904151494577551	No Hit
CGCGACCTCAGATCAGACGC	54640	0.18966883176322555	No Hit
GCATTGGTGGTTCAGTGGTAGAATTCTCGCCT	53844	0.18690572067092087	No Hit
TGAGGTAGTAGGTTGTATAGTT	53707	0.18643016009347646	No Hit
CTCACTGATGAGTACGTTCTGACTTTCGTTCTTCTG	53648	0.1862253566331172	No Hit
TGGAAGACTAGTGATTTTGTTGT	52251	0.18137602724122068	No Hit
AACTGTGATGAAAGATTTGGTCTGTATGTAAT	51020	0.17710292453440277	No Hit
CTGAATGATGATATCCCACTAACTGAGCAGTCAGTA	50672	0.17589493124279218	No Hit
TCGCGAAGGCCCGCGGCGGGTGTTGACGCGATGTGA	50399	0.17494728133299423	No Hit
TAACACTGTCTGGTAACGATGTT	49660	0.17238203121086715	No Hit
TGAAATGATGGCAAATCATCTTTCGGGACTGACCTG	49119	0.1705040876167254	No Hit
ATTCAAATCGATCTGCGCCTT	48035	0.16674125793826022	No Hit
TAATACTGCCTGGTAATGATGAC	47720	0.16564781573464718	No Hit
CTGACCTATGAATTGACAGCC	47495	0.16486678558920934	No Hit
TGAGGTAGTAGTTTGTACAGTT	47395	0.1645196610801258	No Hit
TTCAAATCGATCTGCGCCTTT	46908	0.16282916472088915	No Hit
GCAGCTGATGATACAGCTTCTTTCCCCATC	46699	0.16210367449690466	No Hit
TAGCTTATCAGACTGATGTTG	45163	0.15677184203738204	No Hit
TAGGGTGATGAAAAAGAATCCTTAGGCGTGGTTGTG	44821	0.15558467621631647	No Hit
GACTCTTAGCGGTGGATCACTCGGCTCGTGCGTCGA	41684	0.144695380366367	No Hit
CTTAATGATGACTGTTTTTTTTGATTGCTTGAAGCA	40716	0.14133521511843872	No Hit
ATATATGATGACTTAGCTTTTTTCCCCGAC	39807	0.13817985333086968	No Hit
TTCAAATCGAACTGCGCCTTT	39370	0.13666291922617477	No Hit
TAATACTGCCGGGTAATGATGGA	38621	0.13406295665313936	No Hit
ACGGCCCTGGCGGAGCGCTGAGAAGACGGTCGAACT	36876	0.12800563396963222	No Hit
TAATACTGTCTGGTAAAACCGT	36366	0.12623529897330638	No Hit
AGACGTGGCGACCCGCTGAATTT	36008	0.12499259323078744	No Hit
CCTACTTGGATAACTGTGGTAATTCTAGAGCTAATA	35845	0.12442678028098132	No Hit
CTGACCTATGAATTGACAGCCAT	35204	0.12220171217775608	No Hit
ATGGATTTTTGGAGCAGG	35176	0.1221045173152127	No Hit
CGACTCTTAGCGGTGGATCACTCGGCTCGTG	35036	0.12161854300249579	No Hit
TACCCTGTAGATCCGAATTTGTG	34729	0.12055287075960944	No Hit

Sequence	Count	Percentage	Possible Source
TGTAACAGCAACTCCATGTGGA	34599	0.12010160889780089	No Hit
TTCACAGTGGCTAAGTTCTGC	34328	0.1191609014781846	No Hit
TTCAAATCGATCTGCGCCTTTT	34138	0.11850136491092593	No Hit
TCGTACGACTCTTAGCGGTGGATCACTCGGCTCGTG	33760	0.11718923426659031	No Hit
CTGCTGTGATGACATTCCAATTAAAGCACGTGTTAG	33683	0.116921948394596	No Hit
AGCGCTGAGAAGACGGTCGAACTTGACTATCT	33575	0.11654705392478582	No Hit
TTCCTATGATGAGGACCTTTTCACAGACCTGTACTG	33381	0.11587363237716382	No Hit
TAATACTGCCTGGTAATGATGA	33092	0.11487044254591251	No Hit
TTCAAATCGAACTGCGCCTTTT	32300	0.11212121643397116	No Hit
TCGCGTGATGACATTCTCCGGAATCGCTGTACGGCC	32232	0.11188517176779439	No Hit
CGCGACCTCAGATCAGACG	32094	0.11140613994525916	No Hit
TGTAAACATCCCCGACTGGAAGC	31713	0.11008359556565102	No Hit
TGTAAACATCCCCGACTGGAAG	31238	0.10843475414750439	No Hit
GCATTGGTGGTTCAGTGGTAGAATTCTCGCC	31158	0.10815705454023758	No Hit
GCATATGATGGAAAAGTTTTAATCTCCTGACACTTG	30928	0.10735866816934553	No Hit
TACGGGGATGATTTTACGAACTGAACTCTCTCTTTC	30549	0.10604306627991905	No Hit
GCAGCCGACTTAGAACTGGTGCGGACCAGGGGAATC	30456	0.1057202404864714	No Hit
GGGAGACCGCCTGGGAATACCGGGTGCTGTAGGCTT	29581	0.10268290103199076	No Hit
AAGCTATGATGAATTTGATTGCATTGATCGTCTGAC	28966	0.1005480853011272	No Hit

Adapter Content

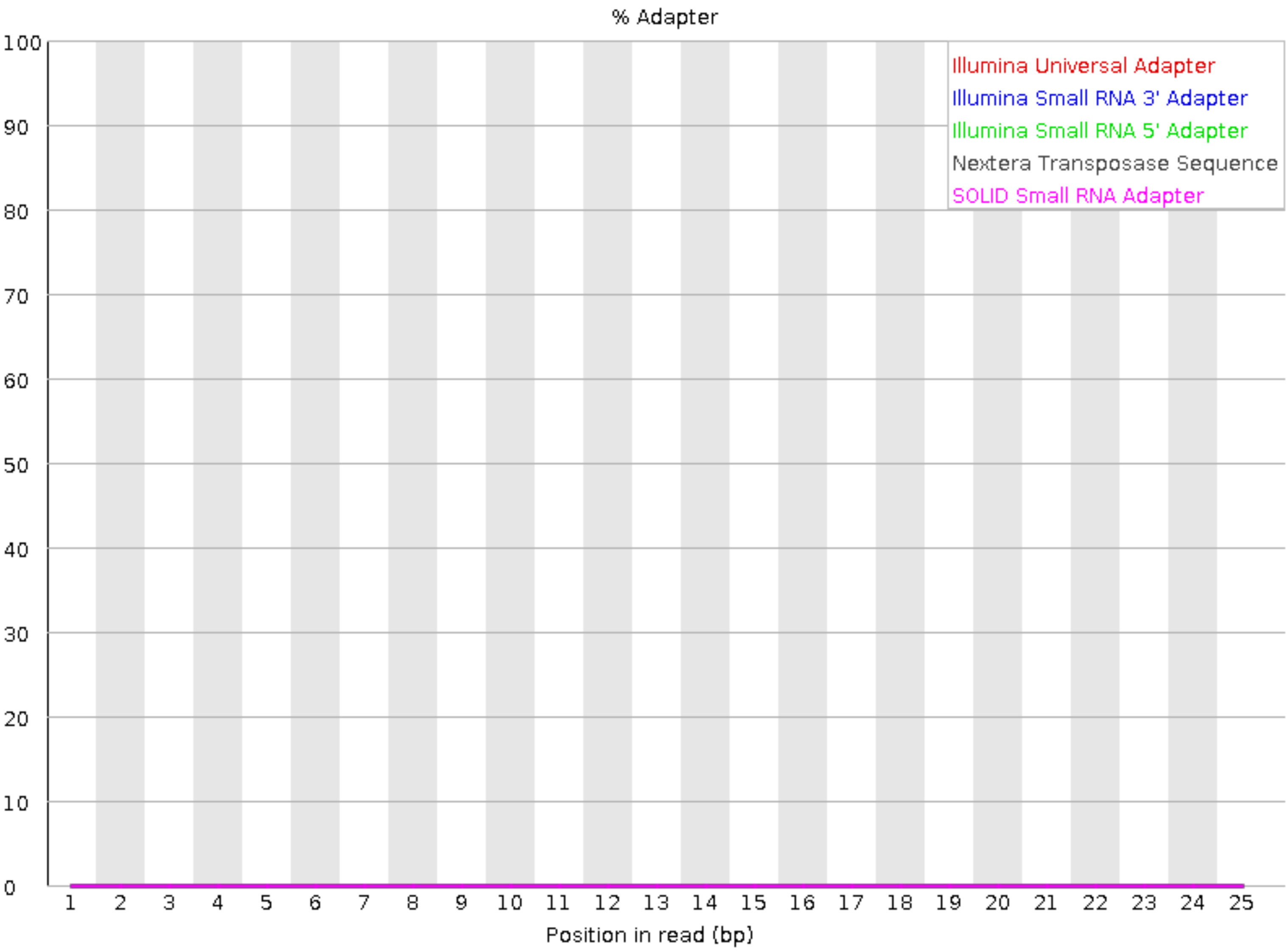
% Adapter
Illumina Universal Adapter
Illumina Small RNA 3' Adapter
Illumina Small RNA 5' Adapter
Nextera Transposase Sequence
SOLID Small RNA Adapter
100
90
80
70
60
50
40
30
20
10
0
1 2 3 4 5 6 7 8 9 10 11 12 13 14 15 16 17 18 19 20 21 22 23 24 25
Position in read (bp)
