# Supplementary material for: CD44 knockdown alters miRNA expression and their target genes in colon cancer
Source: Front Immunol. 2025 May 14;16:1552665. doi: 10.3389/fimmu.2025.1552665 (PMC12116639; doi:10.3389/fimmu.2025.1552665)
Supplement: Supplementary file 5 [file DataSheet5.zip › QC reports/shLUC_3.fastq.gz FastQC Report.pdf]

FastQC Report

Mon 31 Mar 2025
shLUC_3.fastq.gz

Summary

- Basic Statistics
- Per base sequence quality
- Per tile sequence quality
- Per sequence quality scores
- Per base sequence content
- Per sequence GC content
- Per base N content
- Sequence Length Distribution
- Sequence Duplication Levels
- Overrepresented sequences
- Adapter Content

Basic Statistics

Measure	Value
Filename	shLUC_3.fastq.gz
File type	Conventional base calls
Encoding	Sanger / Illumina 1.9
Total Sequences	26969953
Sequences flagged as poor quality	0
Sequence length	18-36
%GC	50

Per base sequence quality

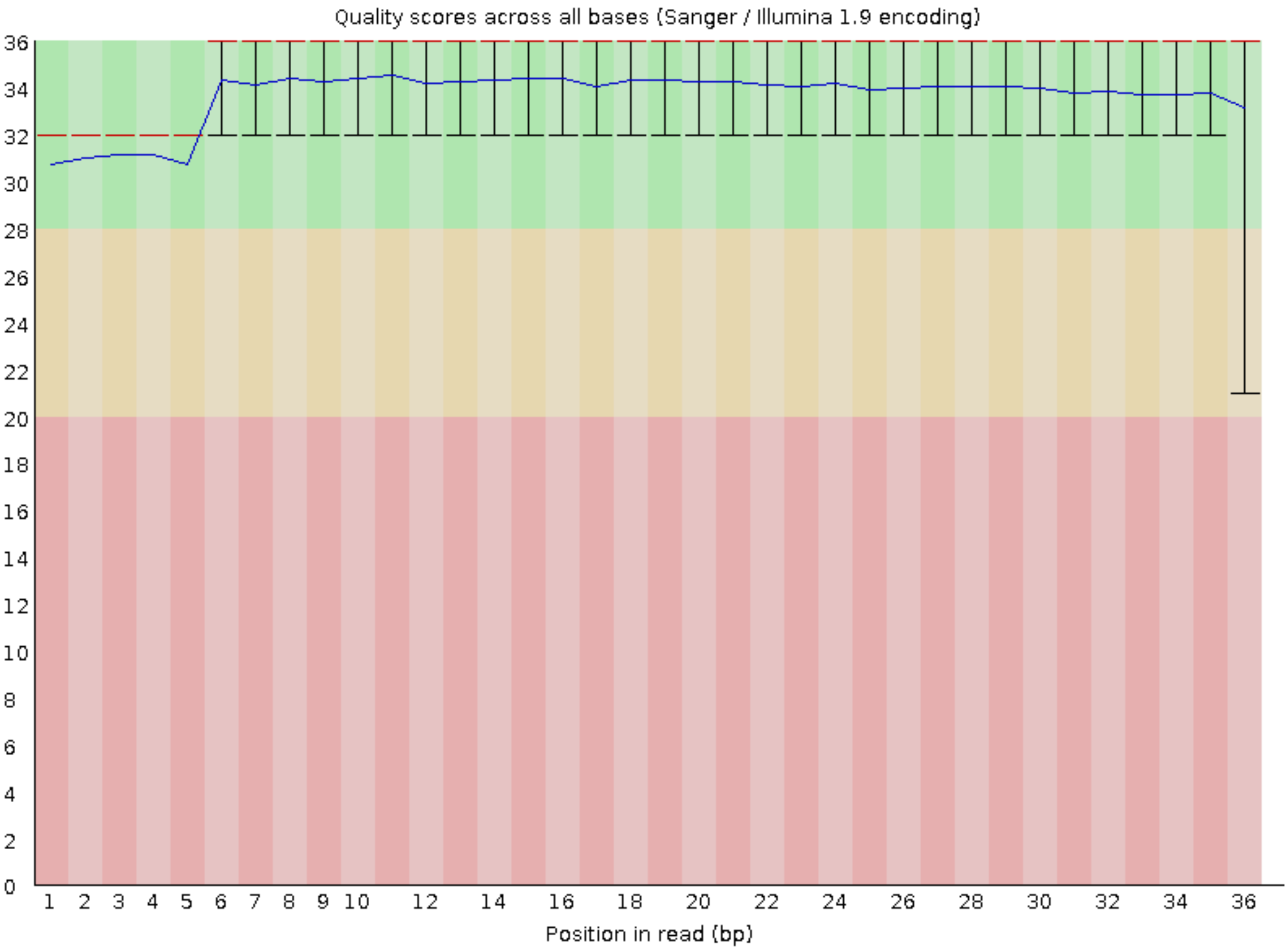

Per tile sequence quality

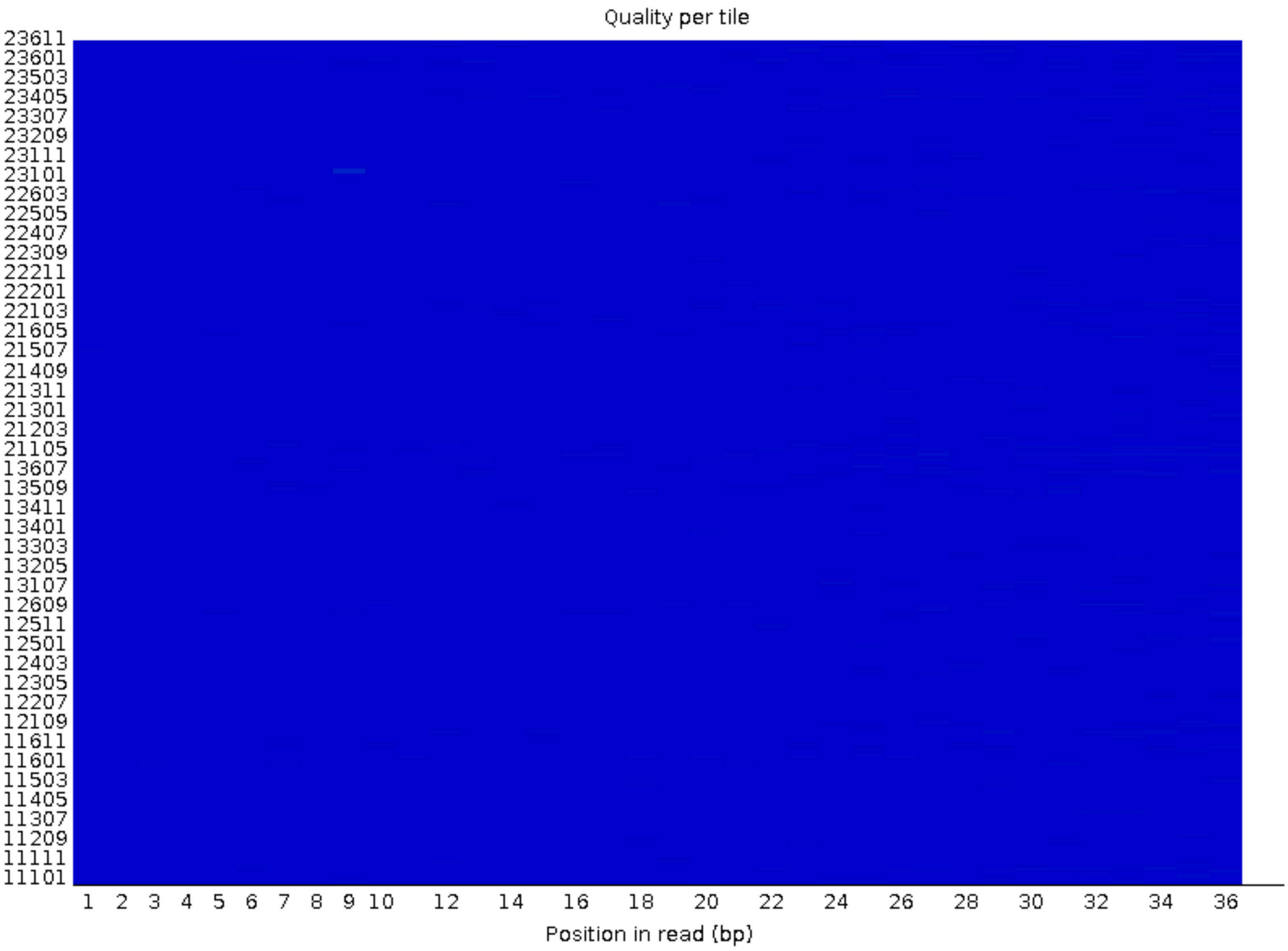

Per sequence quality scores

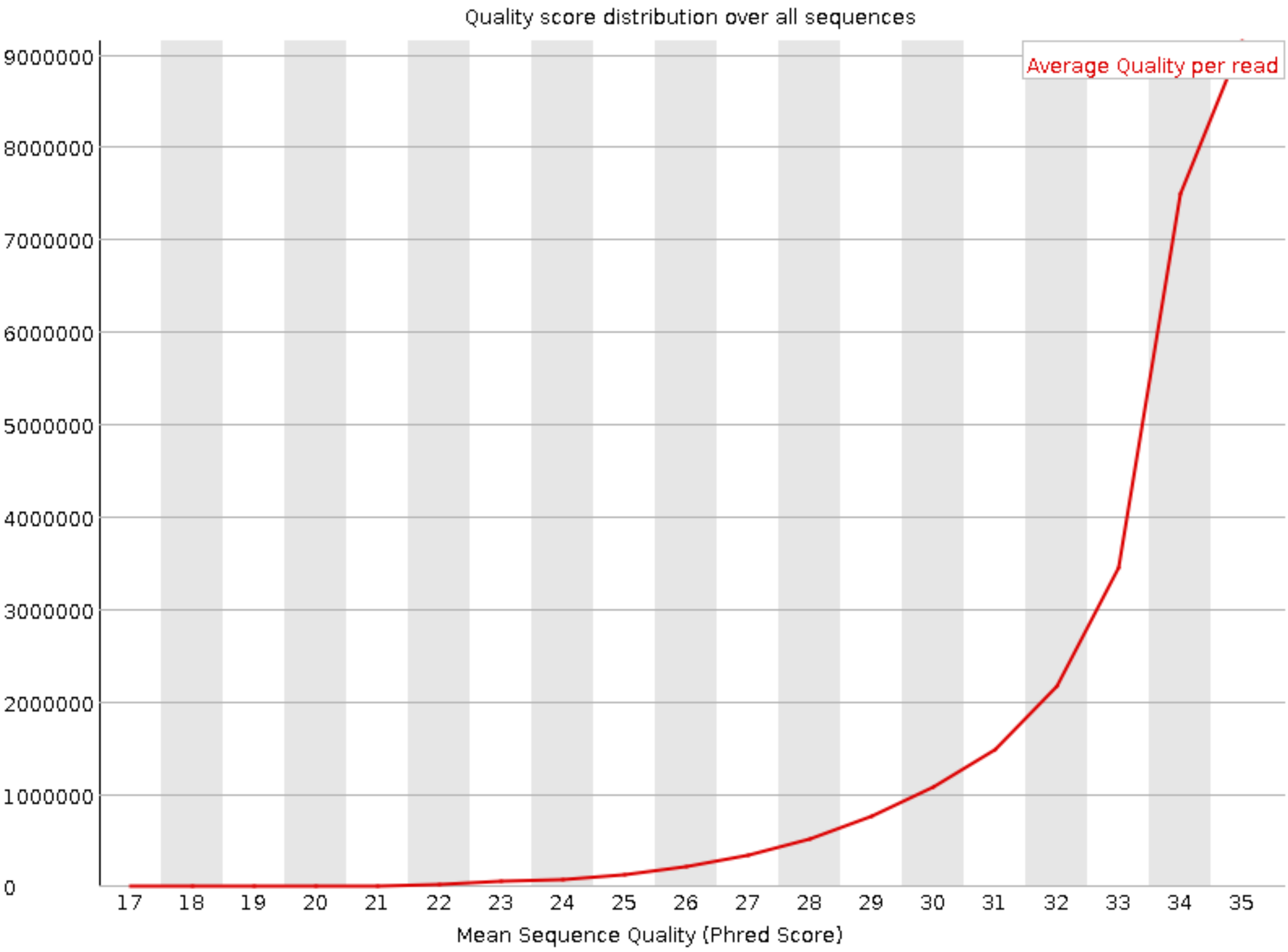

Per base sequence content

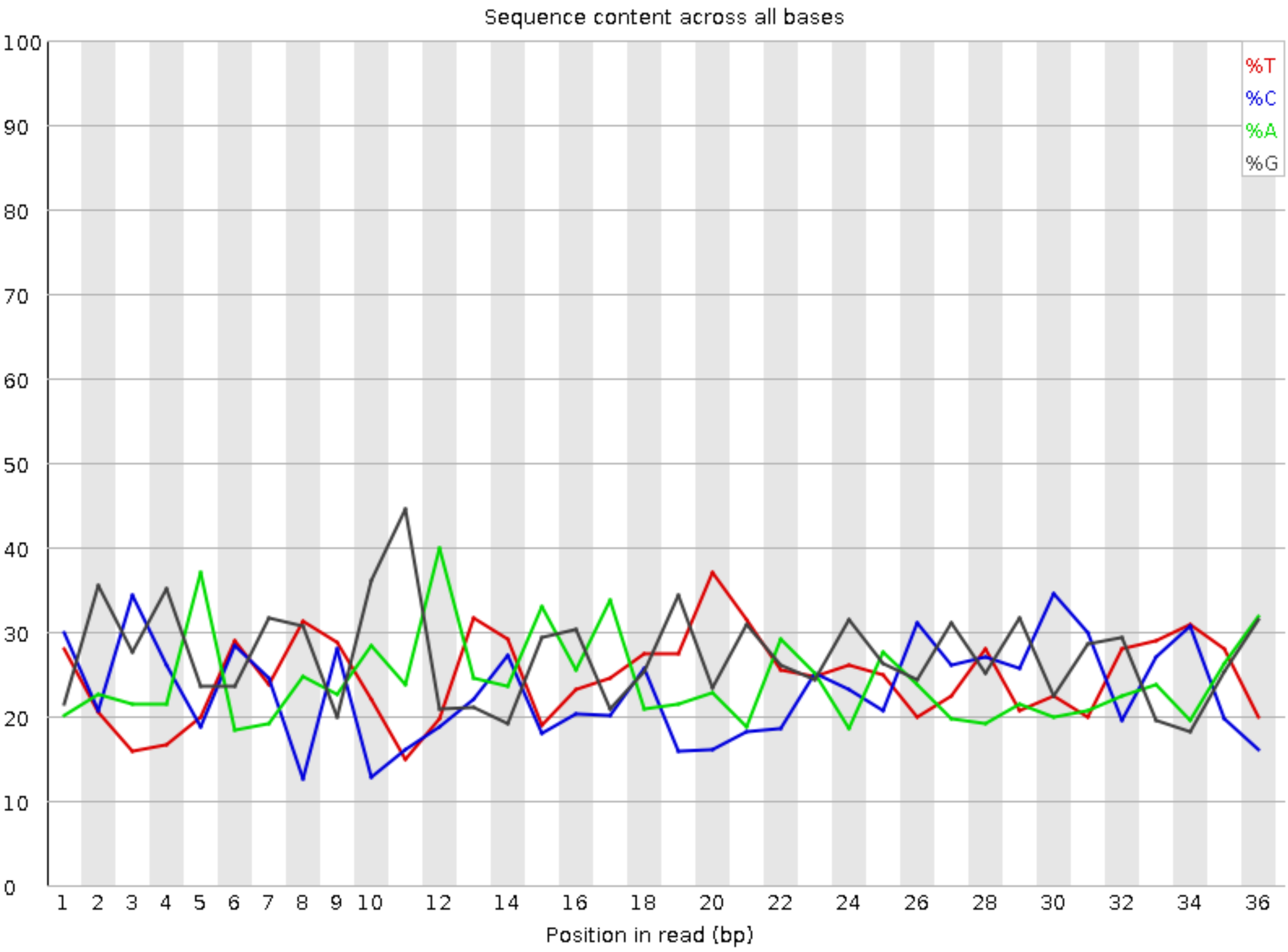

Per sequence GC content

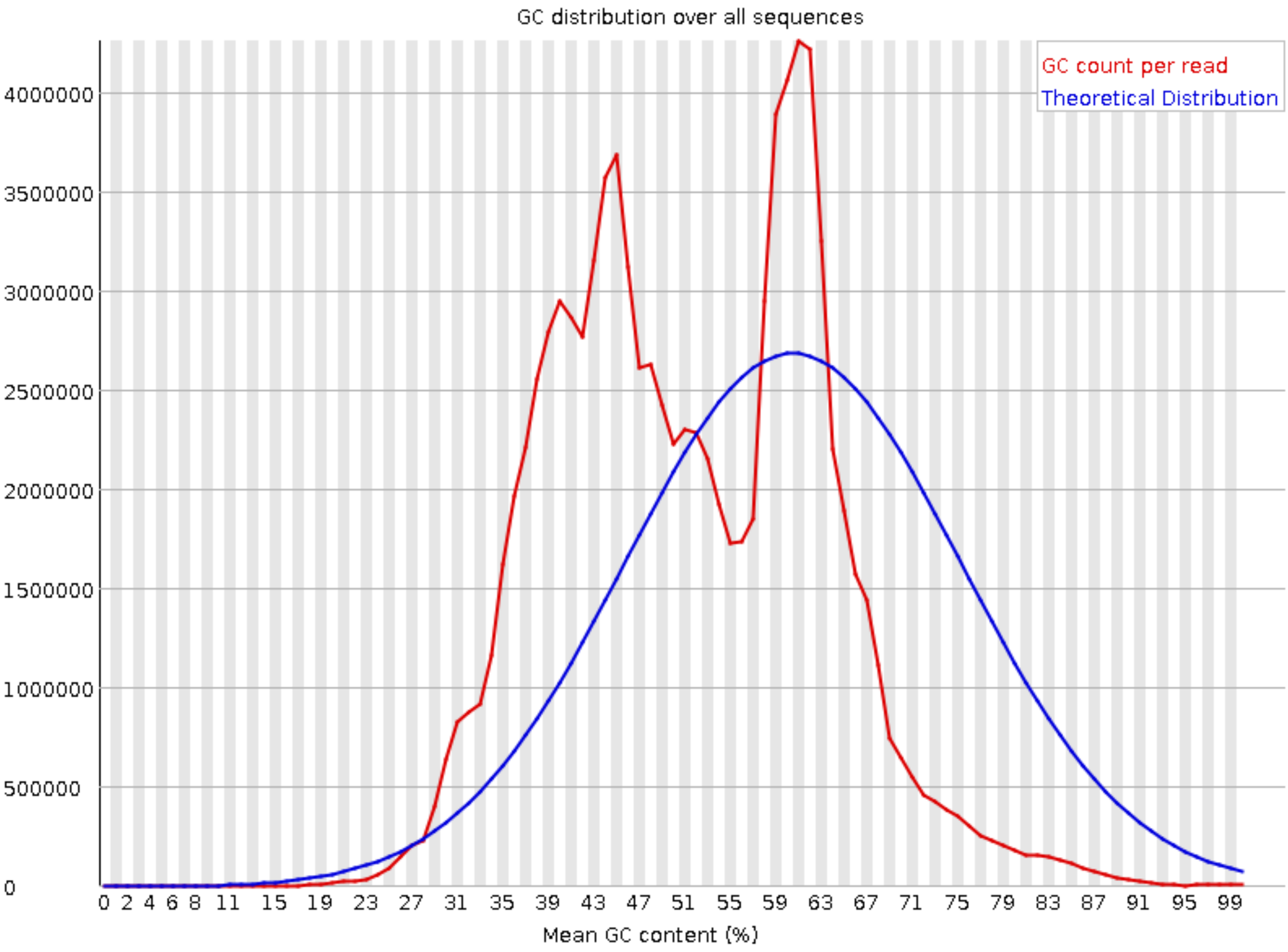

Per base N content

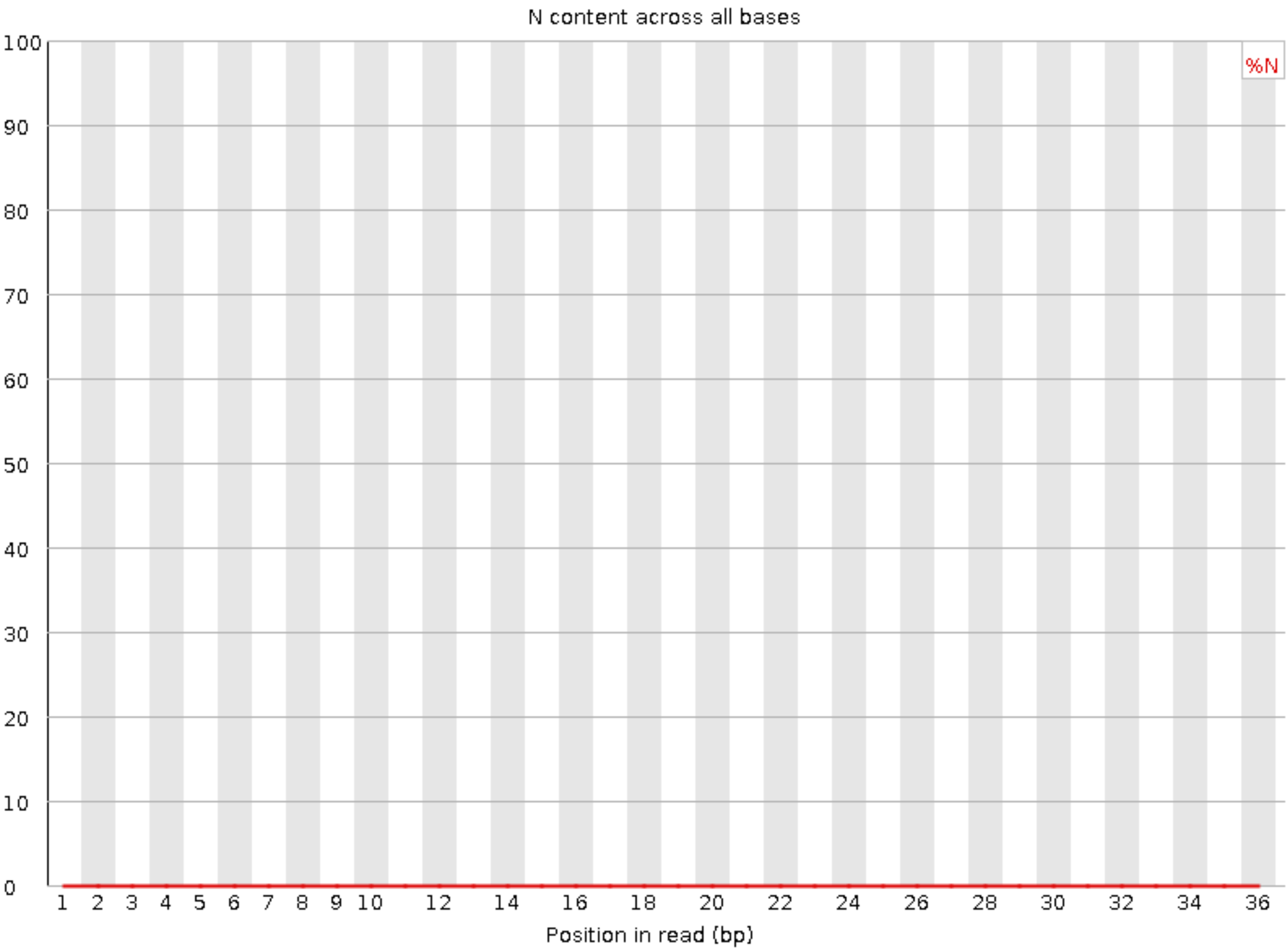

Sequence Length Distribution

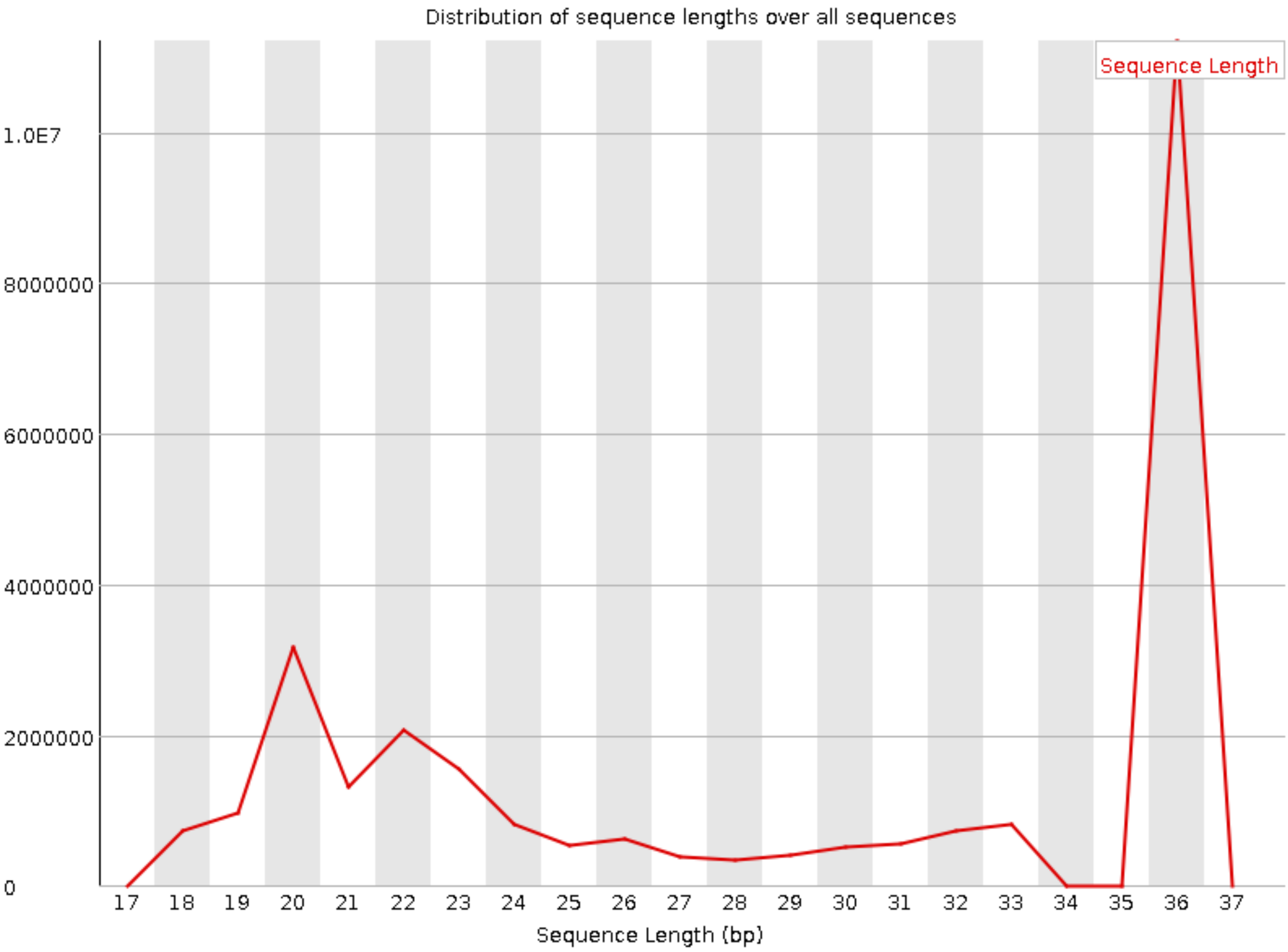

Sequence Duplication Levels

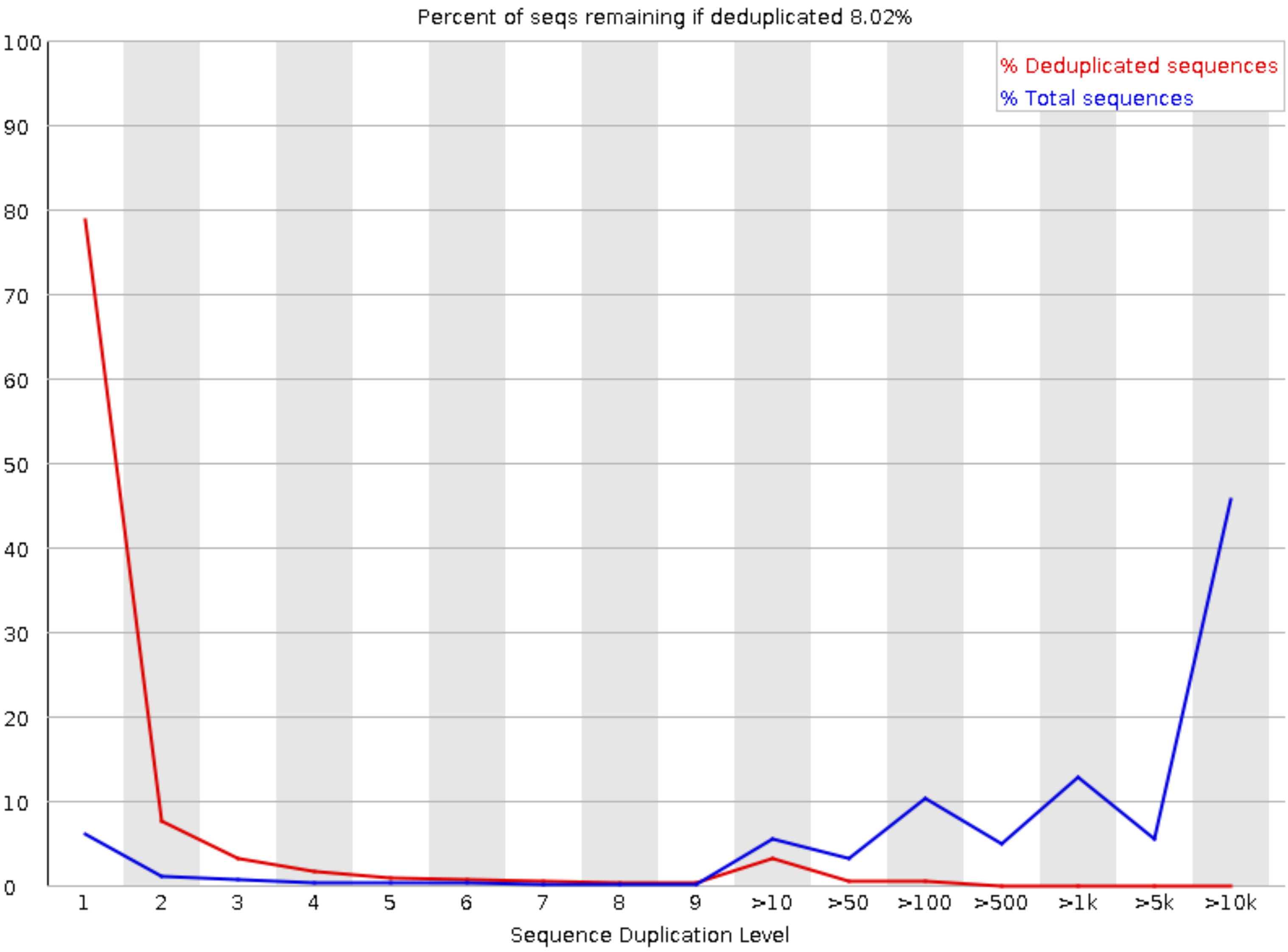

Overrepresented sequences

Sequence	Count	Percentage	Possible Source
CGCGACCTCAGATCAGACGT	1822123	6.756122266879738	No Hit
TGCTCTGATGAAATCACTAATAGGAAGTGCCGTCAG	498911	1.8498771577392068	No Hit
TAGCTTATCAGACTGATGTTGAC	350141	1.2982632932285794	No Hit
GTGAAATGATGGCAATCATCTTTCGGGACTGACCTG	267925	0.9934203444848421	No Hit
GTTTGTGATGACTTACATGGAATCTCGTTCGGCTGA	253370	0.9394528792838461	No Hit
TAGCTTATCAGACTGATGTTGA	236790	0.8779770583953187	No Hit
CGCGACCTCAGATCAGACGC	230187	0.8534942571090132	No Hit
CGCGACCTCAGATCAGACGTGGCGACCCGCTGAATT	205214	0.7608986192893996	No Hit
GAGAAGACGGTCGAACTTGACTATCT	197102	0.7308207025796448	No Hit
TTGAATGATGACTTTAATTGTCGGATACCCCTTCAC	184473	0.683994517899234	No Hit
GCCTCTGATGAAGCCTGTGTTGGTAGGGACATCTGA	183383	0.6799529832328591	No Hit
AGTAGTGATGAAATTCCACTTCATTGGTCCGTGTTT	179964	0.6672759125683312	No Hit

Sequence	Count	Percentage	Possible Source
CCTGGATGATGATAAGCAAATGCTGACTGAACATGA	176816	0.6556036638254431	No Hit
CGCGACCTCAGATCAGACG	164424	0.6096562348477211	No Hit
TATCTGTGATGATCTTATCCCGAACCTGAACTTCTG	161859	0.6001456509768482	No Hit
GTGCAATGATGTATTTTATTCAACACATCATTCTGA	149089	0.552796662270787	No Hit
CGACTCTTAGCGGTGGATCACTCGGCTCGTGCGTCG	140517	0.5210131437752228	No Hit
ATACATGATGATCTCAATCCAACTTGAACTCTCTCA	130462	0.4837309134354072	No Hit
TCGCTGCGATCTATTGAAAGTCAGCCCTCGACACAA	114506	0.42456877844763025	No Hit
GCATTGGTGGTTCAGTGGTAGAATTCTCGCCT	107728	0.3994371069167232	No Hit
TTTCTATGATGAATCAAACTAGCTCACTATGACCGA	105819	0.3923588595056135	No Hit
AGAAGACGGTCGAACTTGACTATCT	105107	0.3897188845675778	No Hit
ACCGGGTGCTGTAGGCTT	102026	0.37829506043262295	No Hit
CAGGACGGTGGCCATGGAAGTCGGAATCCGCTAAGG	101101	0.37486531771115805	No Hit
GATGGGAGACCGCCTGGGAATACCGGGTGCTGTAGG	97859	0.36284453295116975	No Hit
TGGAAGACTAGTGATTTTGTTGTT	96723	0.358632438106214	No Hit
TGAAATGATGGCAATCATCTTTCGGGACTGACCTGA	96624	0.35826536293926803	No Hit
TGAGGTAGTAGTTTGTGCTGTT	94986	0.3521919374497983	No Hit
TTGGTACTAGCAACGCACTTT	92670	0.3436046032412441	No Hit
CTACGGGGATGATTTTACGAACTGAACTCTCTCTTT	89683	0.33252931512338935	No Hit
CGCGACCTCAGATCAGACGA	89294	0.33108696926538955	No Hit
TGAGGTAGTAGATTGTATAGTT	88167	0.3269082448901561	No Hit
TAGCTTATCAGACTGATGTTGAT	86617	0.3211611084379717	No Hit
ACCGGGTGCTGTAGGCTTT	82251	0.3049727227926574	No Hit
CGCGACCTCAGATCAGACGG	82250	0.3049690149626883	No Hit
CTCGCTGCGATCTATTGAAAGTCAGCCCTCGACACA	76211	0.28257742977898404	No Hit
ACAAATGATGAATAACAAAGGGACTTAATACTG	75967	0.28167271926651116	No Hit
CTGGATGATGATAAGCAAATGCTGACTGAACATGAA	70823	0.26259964190519725	No Hit
TTTGAATGATGACTTTAATTGTCGGATACCCCTTCA	70354	0.2608606696496653	No Hit
CTCCTACTTGGATAACTGTGGTAATTCTAGAGCTAA	70189	0.26024887770475535	No Hit
ACGGCCCTGGCGGAGCGCTGAGAAGACGGTCGAACT	70092	0.25988921819774774	No Hit
CGCTGCGATCTATTGAAAGTCAGCCCTCGACACAAG	69941	0.25932933587240586	No Hit
TGCCTCTGATGAAGCCTGTGTTGGTAGGGACATCTG	64513	0.23920323479985303	No Hit
GCAAATGATGATAAACTGGATCTGACTGACTGTGCT	63618	0.23588472697746266	No Hit
GTGAAATGATGGCAAATCATCTTTCGGGACTGACCT	61646	0.22857288627829644	No Hit
TCTCCTACTTGGATAACTGTGGTAATTCTAGAGCTA	57764	0.21417909033805138	No Hit
TGGGAGACCGCCTGGGAATACCGGGTGCTGTAGGCT	57012	0.21139080220124967	No Hit
TAGCTTATCAGACTGATGTTGACT	54223	0.2010496644172869	No Hit
AATGGATTTTTGGAGCAGG	54198	0.2009569686680581	No Hit

Sequence	Count	Percentage	Possible Source
TCAGTGCACTACAGAACTTTGT	53180	0.19718239775946217	No Hit
TAGCTTATCAGACTGATGTTG	52804	0.1957882536910613	No Hit
CTGCAGTGATGACTTTCTTAGGACACCTTTGGATTT	52375	0.19419759463429545	No Hit
GCATTGGTGGTTCAGTGGTAGAATTCTCGCC	51514	0.19100515303085625	No Hit
ACTCCATGATGAACACAAAATGACAAGCATATGGCT	51166	0.1897148282015916	No Hit
TGAAATGATGGCAAATCATCTTTCGGGACTGACCTG	50478	0.18716384118281557	No Hit
CTAGACTGAAGCTCCTTGAGG	49848	0.1848279083022503	No Hit
TTCAAGTAATCCAGGATAGGCT	49552	0.18373039063138152	No Hit
TACCCTGTAGATCCGAATTTGT	49333	0.18291837586813742	No Hit
TAGCTTATCAGACTGATGTTGACA	47758	0.17707854366672424	No Hit
GCAGCTGATGATACAGCTTCTTTCCCCATC	47281	0.1753099087714391	No Hit
TCCCTGGTGGTCTAGTGGTTAGGATTCGGCGCT	47270	0.17526912264177844	No Hit
CTCACTGATGAGTACGTTCTGACTTTCGTTCTTCTG	46277	0.17158724748241125	No Hit
CTGAATGATGATATCCCACTAACTGAGCAGTCAGTA	45821	0.16989647701647828	No Hit
CACAGATGATGAACTTATTGACGGGCGGACAGAAAC	45764	0.16968513070823668	No Hit
CGGCCCTGGCGGAGCGCTGAGAAGACGGTCGAACTT	44860	0.16633325241612398	No Hit
AACTGTGATGAAAGATTTGGTCTGTATGTAAT	43953	0.1629702506341038	No Hit
GGCTGGTCCGATGGTAGTGGGTTATCAGAACT	40001	0.14831690659601818	No Hit
CGACTCTTAGCGGTGGATCACTCGGCTCG	39932	0.14806106632814672	No Hit
GACTCTTAGCGGTGGATCACTCGGCTCGTGCGTCGA	38120	0.1413424784240447	No Hit
ATATATGATGACTTAGCTTTTTTCCCCGAC	37282	0.138235316909896	No Hit
TAGGGTGATGAAAAAGAATCCTTAGGCGTGGTTGTG	36395	0.13494647172725885	No Hit
TGAGGTAGTAGTTTGTACAGTT	35751	0.13255862922712547	No Hit
TAACACTGTCTGGTAACGATGTT	35528	0.13173178314400474	No Hit
GCAGCCGACTTAGAACTGGTGCGGACCAGGGGAATC	35336	0.1310198797899277	No Hit
TACGGGGATGATTTTACGAACTGAACTCTCTCTTTC	34317	0.1272416010513626	No Hit
CGCGACCTCAGATCAGACGTGGCGACCCGCTGAATA	34188	0.1267632909853421	No Hit
TTGGTACTAGCAACGCACTTTT	34168	0.12668913438595908	No Hit
AGACGTGGCGACCCGCTGAATTT	34107	0.12646295675784086	No Hit
TGAGGTAGTAGGTTGTATAGTT	34080	0.12636284534867376	No Hit
TCCCACATGGTCTAGCGGTTAGGATTCCTGGTT	34052	0.12625902610953751	No Hit
TAATACTGCCTGGTAATGATGAC	33656	0.12479072544175364	No Hit
ATACATGATGATCTCACACAACTTGAACTCTCTCAC	33327	0.1235708493819029	No Hit
AAGCTATGATGAATTTGATTGCATTGATCGTCTGAC	33324	0.12355972589199544	No Hit
CTGACCTATGAATTGACAGCC	32848	0.12179479882667946	No Hit
CGCGACCTCAGATCAGACA	32643	0.12103469368300344	No Hit
GAGAAGACGGTCGAACTTGACTATCTAGAGGAAGTA	32439	0.1202782963692966	No Hit

Sequence	Count	Percentage	Possible Source
TCGTACGACTCTTAGCGGTGGATCACTCGGCTCGTG	32221	0.11946998943602163	No Hit
GCCGCCGGTGAAATACCACTACTCTGATCGTTTTTT	31078	0.11523193978128178	No Hit
TCCTACTTGGATAACTGTGGTAATTCTAGAGCTAAT	31002	0.11495014470362629	No Hit
TTGCATGATGACTTGAATTGTCGGATACCCCTTCAC	30846	0.11437172322843871	No Hit
GCATTGGTGGTTCAGTGGTAGAATTCTCGCT	30741	0.11398240108167783	No Hit
AGCGCTGAGAAGACGGTCGAACTTGACTATCT	30543	0.11324825074778587	No Hit
GCATATGATGGAAAAGTTTTAATCTCCTGACACTTG	30365	0.11258825701327697	No Hit
GACGTGGCGACCCGCTGAATTT	30133	0.11172804046043387	No Hit
GAGAAGACGGTCGAACTTGACTATCC	30093	0.11157972726166783	No Hit
CTTAATGATGACTGTTTTTTTTGATTGCTTGAAGCA	29125	0.10799054785152944	No Hit
TCGCGTGATGACATTCTCCGGAATCGCTGTACGGCC	28313	0.10497978991657864	No Hit
TGGAAGACTAGTGATTTTGTTGT	27671	0.10259936307638357	No Hit
TCGCGAAGGCCCGCGGCGGGTGTTGACGCGATGTGA	27160	0.10070466196214728	No Hit

Adapter Content

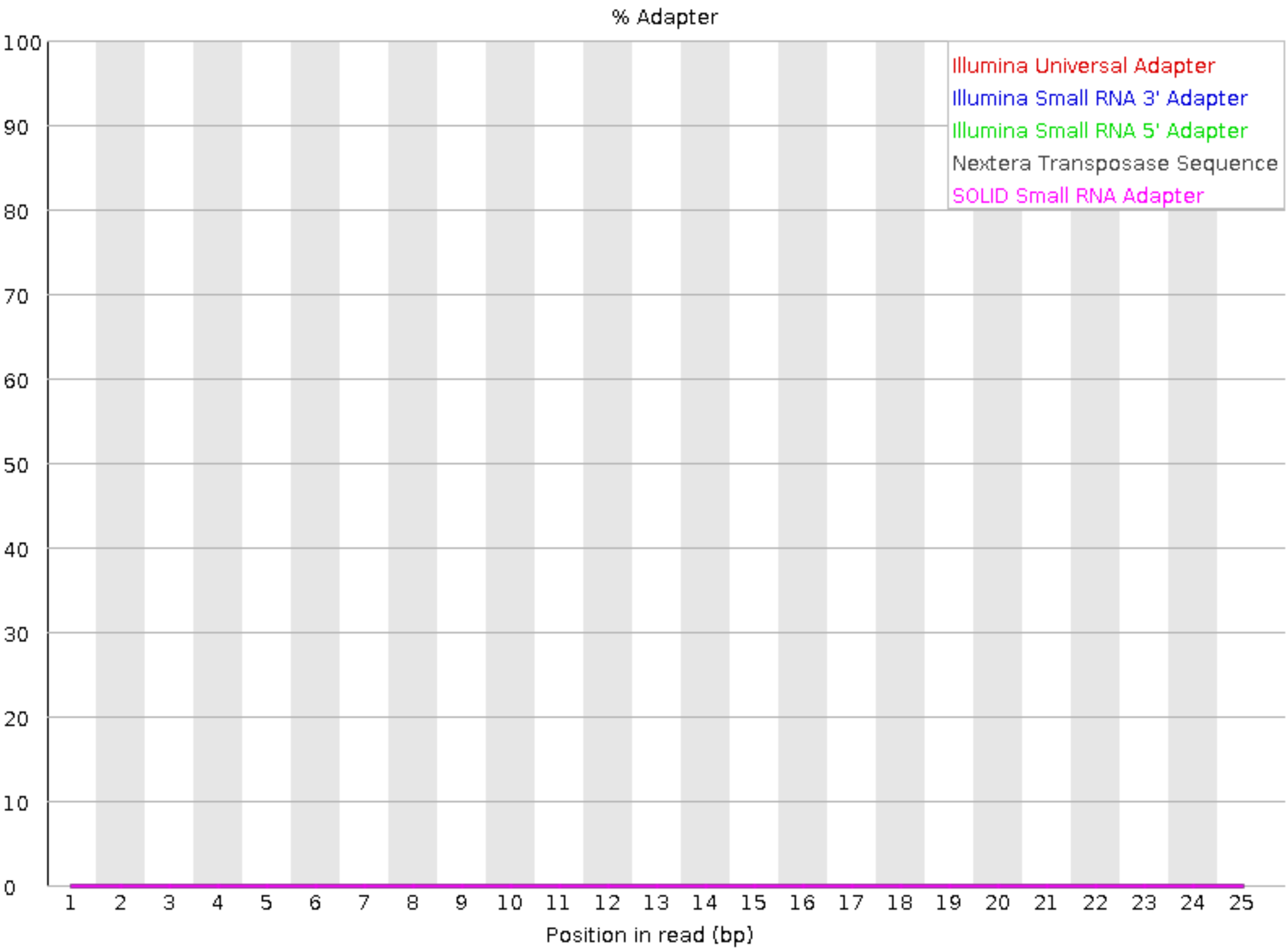

Produced by FastQC (version 0.11.9)
