# Supplementary material for: CD44 knockdown alters miRNA expression and their target genes in colon cancer
Source: Front Immunol. 2025 May 14;16:1552665. doi: 10.3389/fimmu.2025.1552665 (PMC12116639; doi:10.3389/fimmu.2025.1552665)
Supplement: Supplementary file 5 [file DataSheet5.zip › QC reports/shLUC_4.fastq.gz FastQC Report.pdf]

FastQC Report

Mon 31 Mar 2025
shLUC_4.fastq.gz

Summary

- Basic Statistics
- Per base sequence quality
- Per tile sequence quality
- Per sequence quality scores
- Per base sequence content
- Per sequence GC content
- Per base N content
- Sequence Length Distribution
- Sequence Duplication Levels
- Overrepresented sequences
- Adapter Content

Basic Statistics

Measure	Value
Filename	shLUC_4.fastq.gz
File type	Conventional base calls
Encoding	Sanger / Illumina 1.9
Total Sequences	27909830
Sequences flagged as poor quality	0
Sequence length	18-36
%GC	51

Per base sequence quality

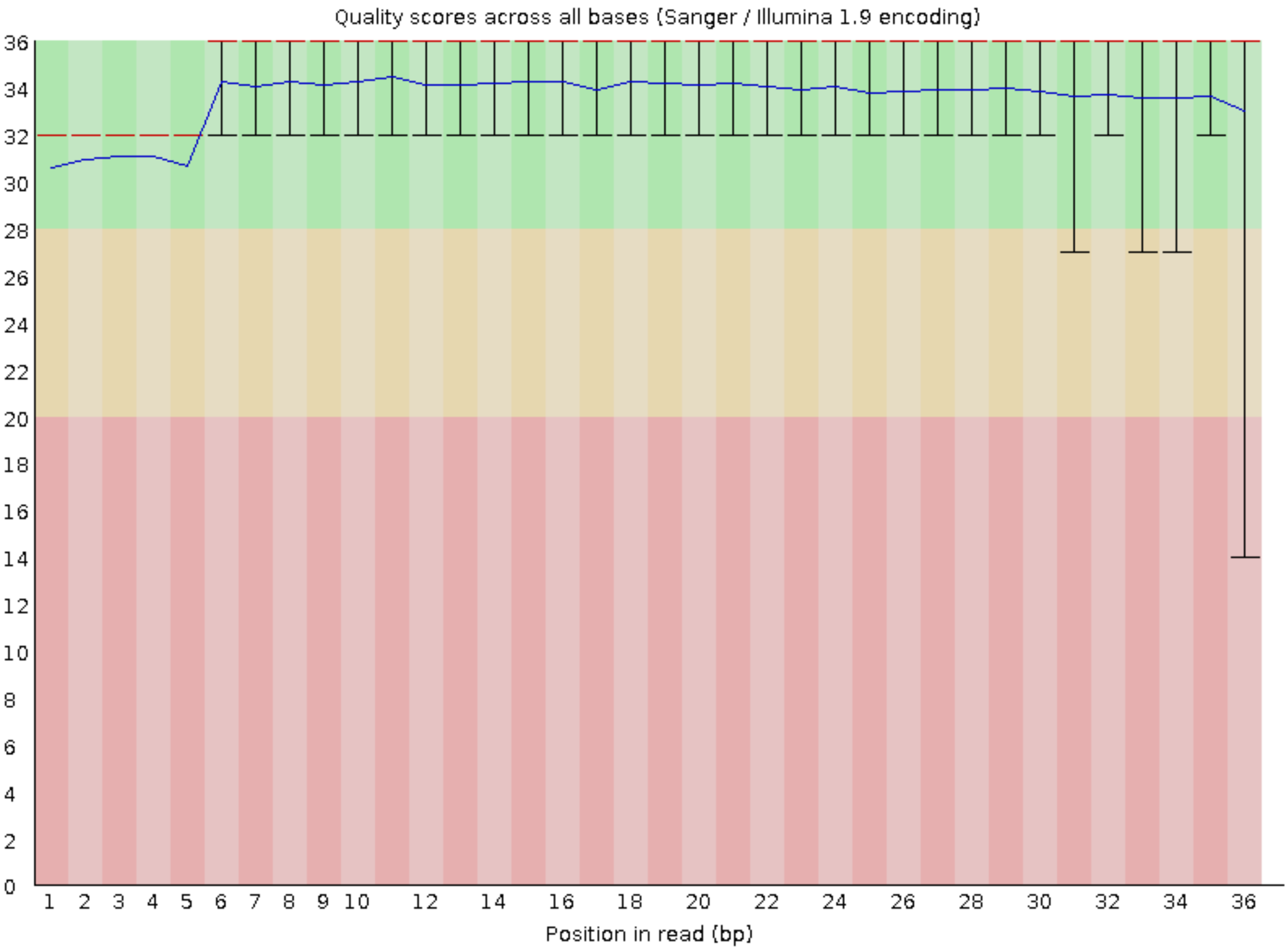

Per tile sequence quality

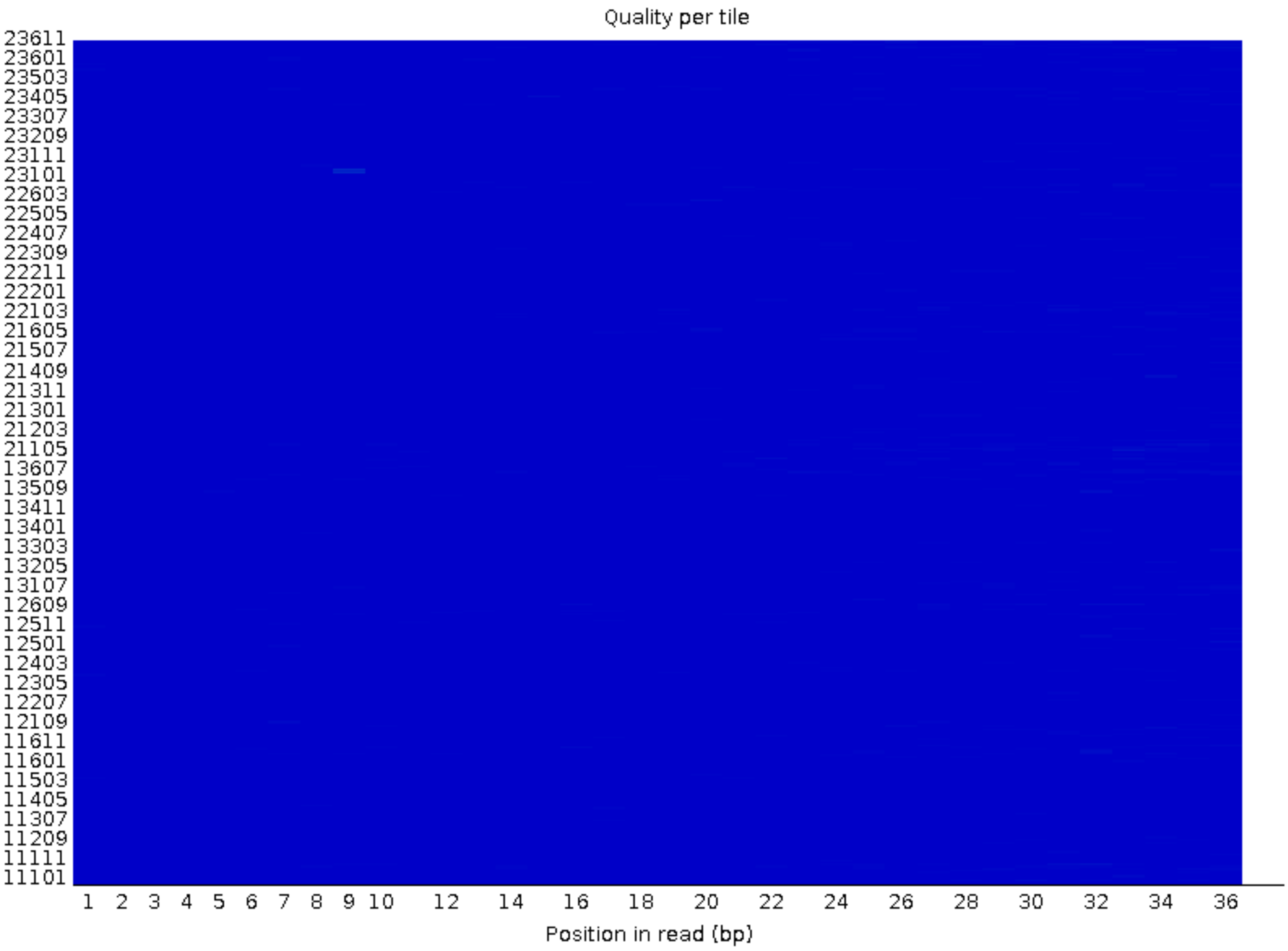

Per sequence quality scores

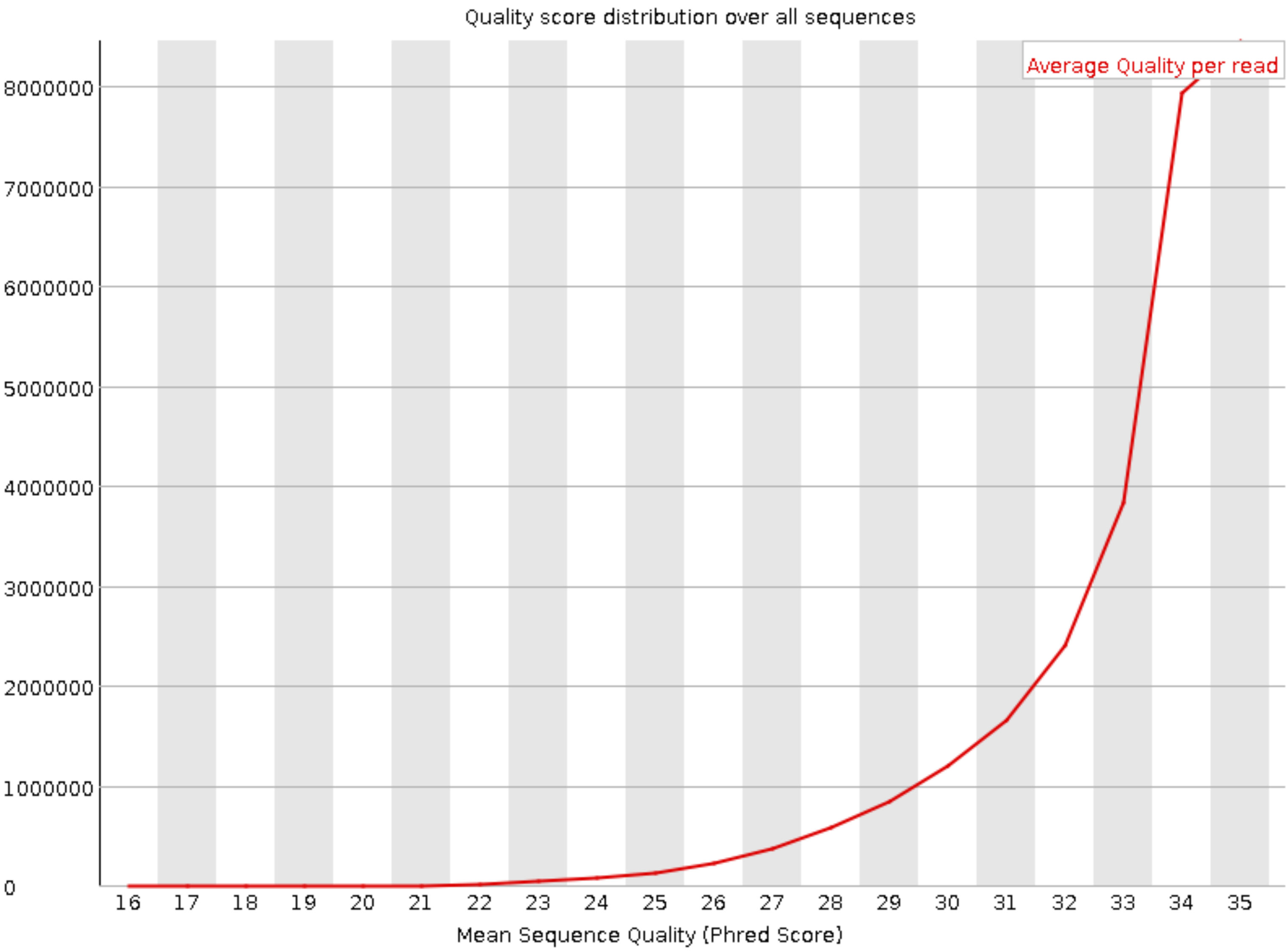

Per base sequence content

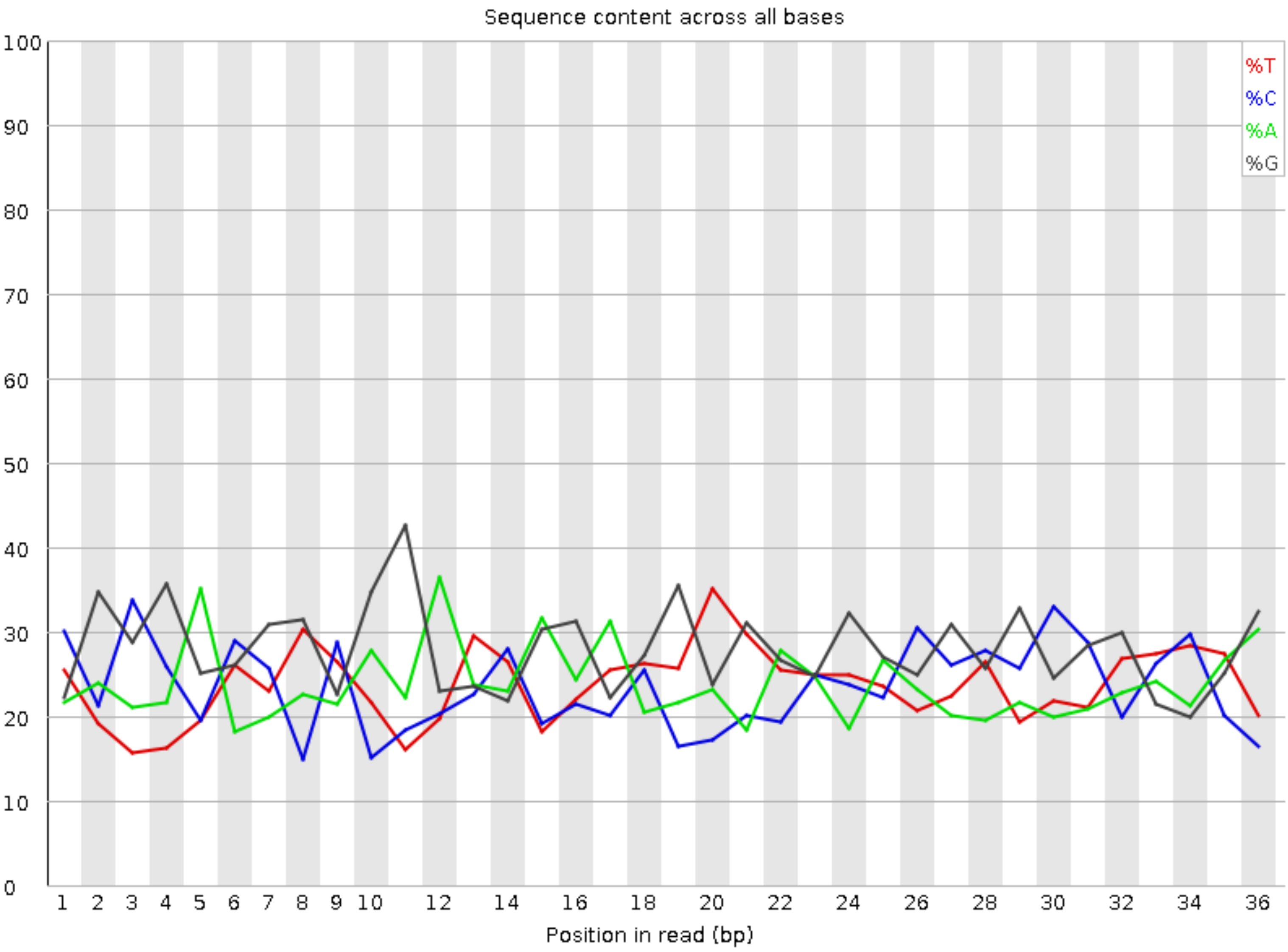

Per sequence GC content

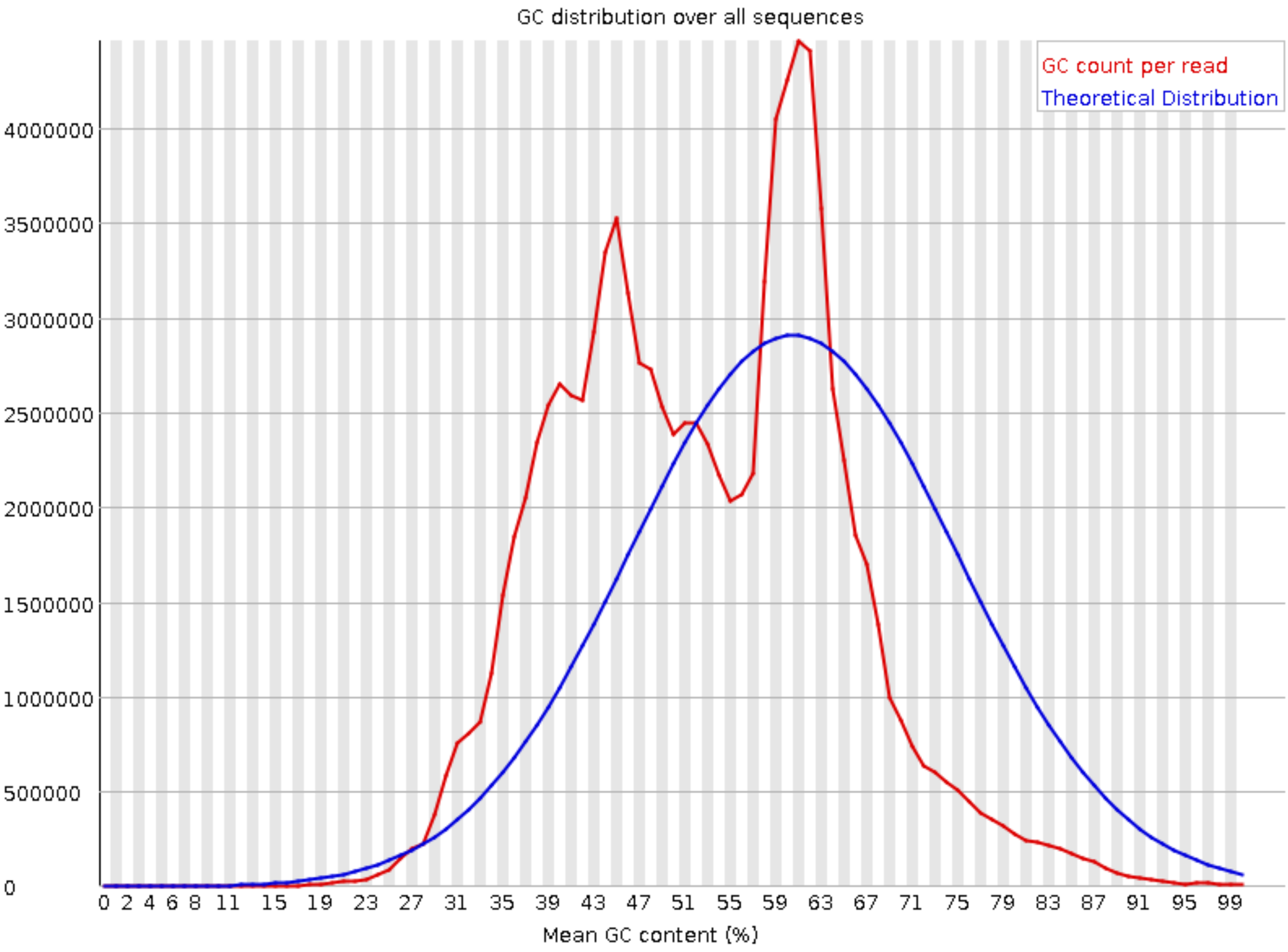

Per base N content

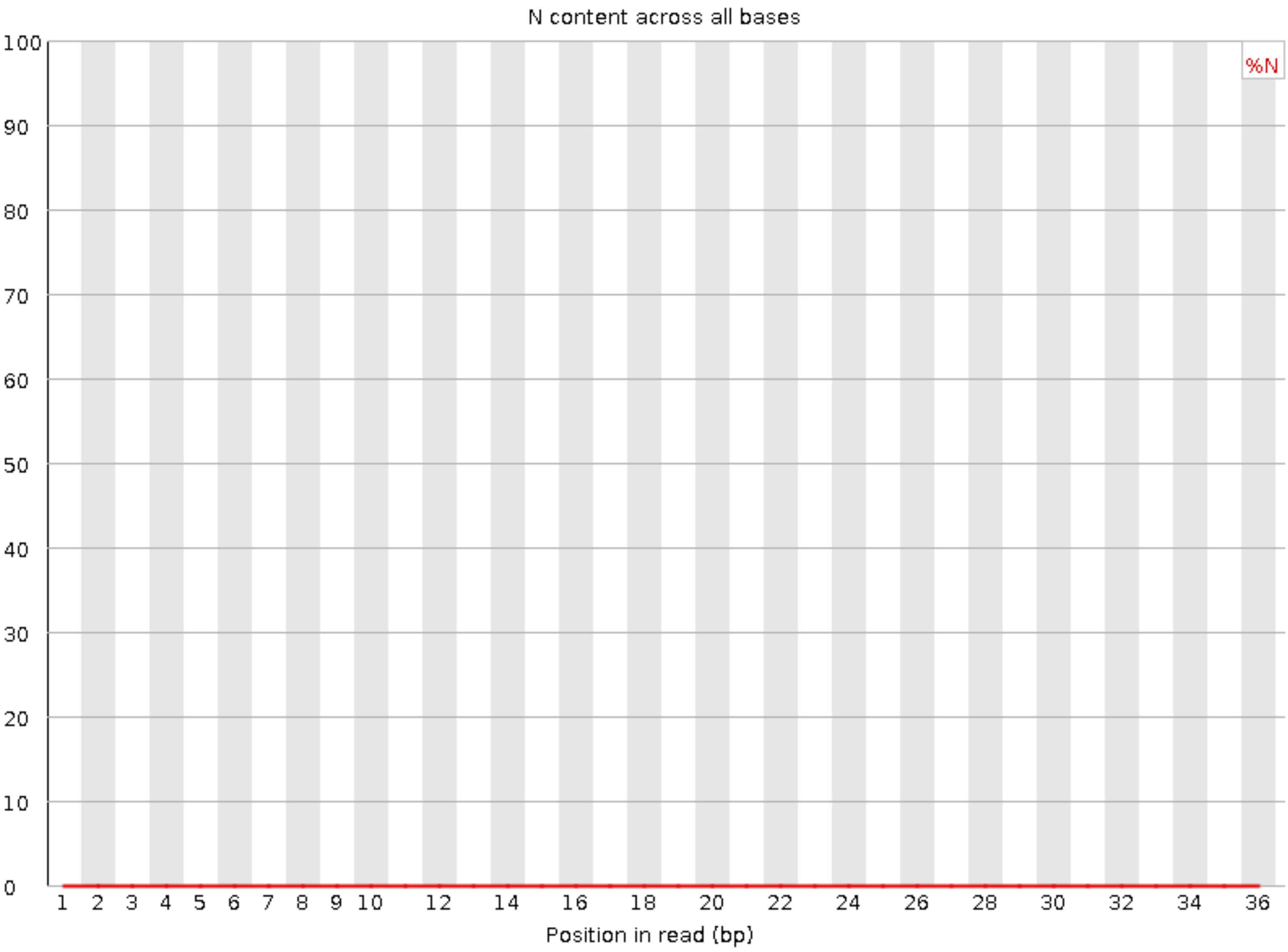

Sequence Length Distribution

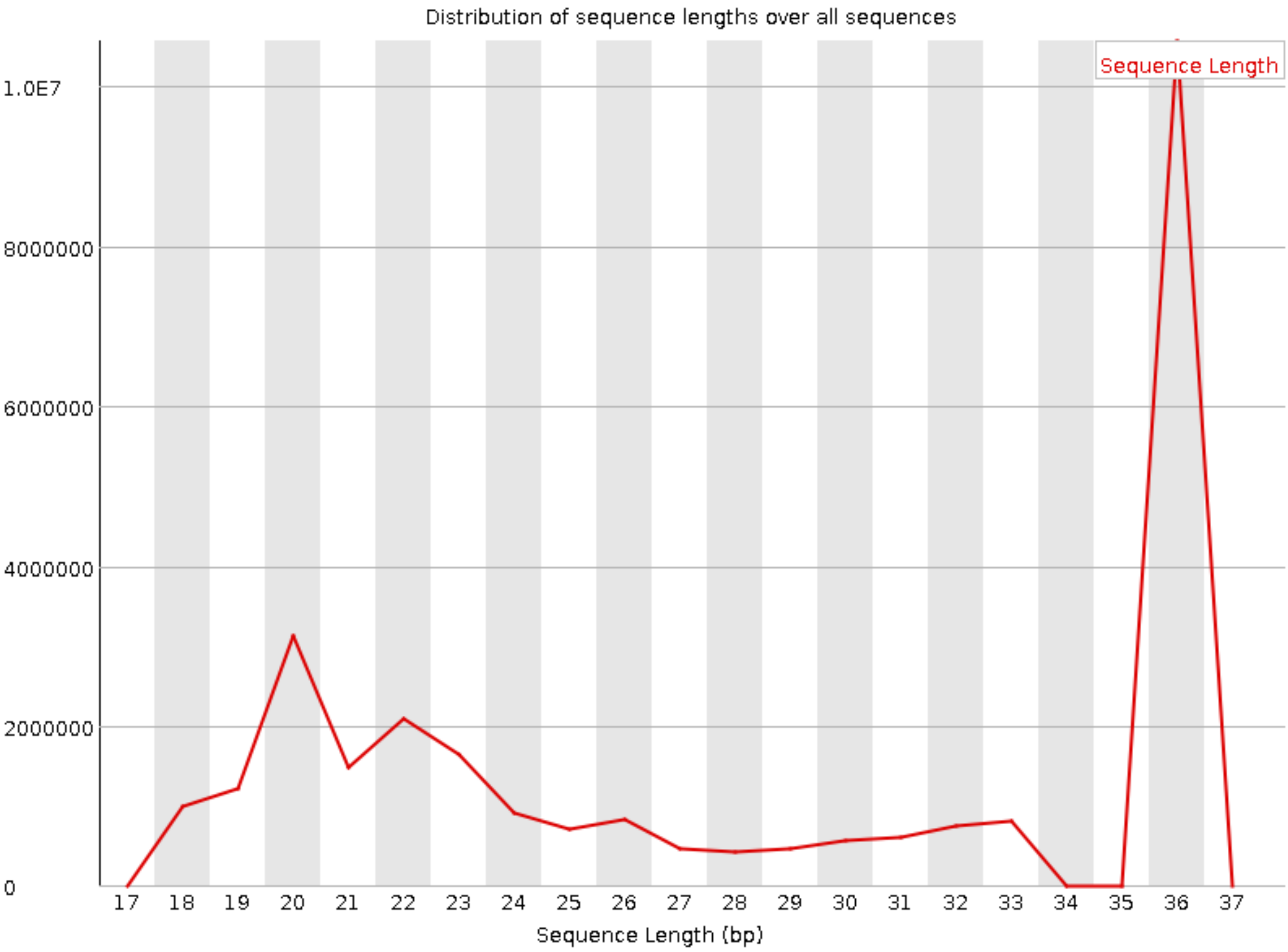

Sequence Duplication Levels

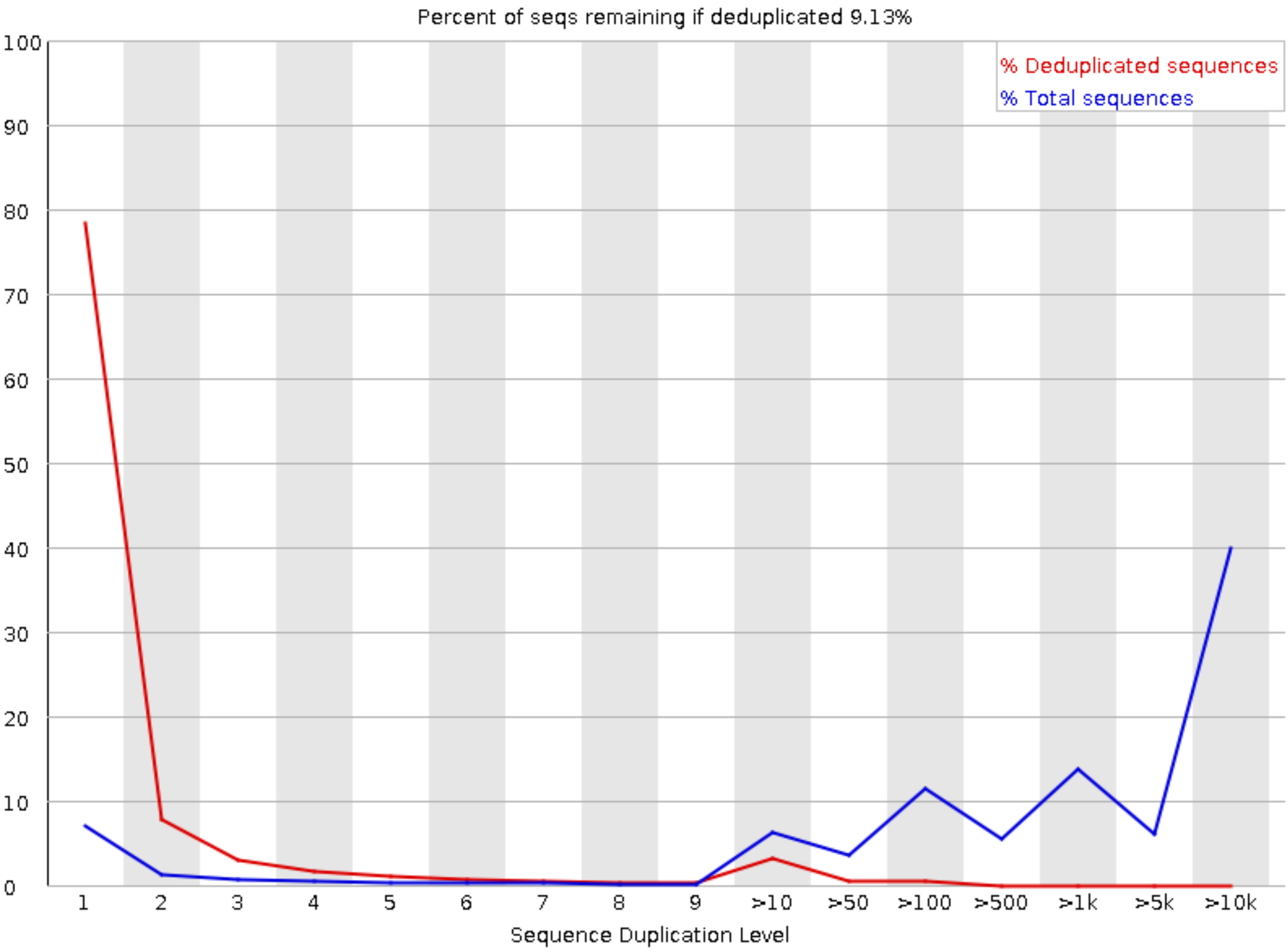

Overrepresented sequences

Sequence	Count	Percentage	Possible Source
CGCGACCTCAGATCAGACGT	1581714	5.667229073054189	No Hit
TGCTCTGATGAAATCACTAATAGGAAGTGCCGTCAG	394366	1.4130003658209311	No Hit
TAGCTTATCAGACTGATGTTGAC	278638	0.9983507602876837	No Hit
GAGAAGACGGTCGAACTTGACTATCT	270111	0.9677988006376249	No Hit
CGCGACCTCAGATCAGACGTGGCGACCCGCTGAATT	232430	0.8327890209291853	No Hit
TAGCTTATCAGACTGATGTTGA	223604	0.8011657541446867	No Hit
CGCGACCTCAGATCAGACGC	220434	0.7898077487394226	No Hit
GTGAAATGATGGCAATCATCTTTCGGGACTGACCTG	219851	0.7877188789756154	No Hit
CGCGACCTCAGATCAGACG	163652	0.5863597162720088	No Hit
GTTTGTGATGACTTACATGGAATCTCGTTCGGCTGA	160667	0.5756645597626355	No Hit
GCCTCTGATGAAGCCTGTGTTGGTAGGGACATCTGA	157338	0.563736862603606	No Hit
CGACTCTTAGCGGTGGATCACTCGGCTCGTGCGTCG	152342	0.5458363594475495	No Hit

Sequence	Count	Percentage	Possible Source
TTGAATGATGACTTTAATTGTCGGATACCCCTTCAC	147433	0.5282475744209119	No Hit
ACCGGGTGCTGTAGGCTT	145403	0.5209741514011372	No Hit
AGAAGACGGTCGAACTTGACTATCT	144542	0.5178892168099913	No Hit
TATCTGTGATGATCTTATCCCGAACCTGAACTTCTG	136739	0.4899313252714187	No Hit
AGTAGTGATGAAATTCCACTTCATTGGTCCGTGTTT	124756	0.4469966316527188	No Hit
GTGCAATGATGTATTTTATTCAACACATCATTCTGA	124350	0.4455419470487638	No Hit
GATGGGAGACCGCCTGGGAATACCGGGTGCTGTAGG	118443	0.4243773609513207	No Hit
ACCGGGTGCTGTAGGCTTT	116722	0.41821107473603386	No Hit
TCGCTGCGATCTATTGAAAGTCAGCCCTCGACACAA	114687	0.41091973688123506	No Hit
CCTGGATGATGATAAGCAAATGCTGACTGAACATGA	106075	0.380063225035767	No Hit
CAGGACGGTGGCCATGGAAGTCGGAATCCGCTAAGG	99754	0.35741529059833044	No Hit
TAGCTTATCAGACTGATGTTGAT	96050	0.3441439808124951	No Hit
CGCGACCTCAGATCAGACGA	95479	0.34209810665274565	No Hit
ATACATGATGATCTCAATCCAACTTGAACTCTCTCA	90726	0.3250682644788592	No Hit
TGGAAGACTAGTGATTTTGTTGTT	85686	0.3070101107745909	No Hit
CTCCTACTTGGATAACTGTGGTAATTCTAGAGCTAA	82021	0.29387853670194336	No Hit
TTTCTATGATGAATCAAACTAGCTCACTATGACCGA	81115	0.29063236859558084	No Hit
CGCTGCGATCTATTGAAAGTCAGCCCTCGACACAAG	79708	0.28559113401980596	No Hit
GCATTGGTGGTTCAGTGGTAGAATTCTCGCCT	78562	0.2814850538322878	No Hit
CGCGACCTCAGATCAGACGG	75532	0.27062866380769784	No Hit
TGAAATGATGGCAATCATCTTTCGGGACTGACCTGA	74617	0.267350248998292	No Hit
CTACGGGGATGATTTTACGAACTGAACTCTCTCTTT	73321	0.26270672376005155	No Hit
TTGGTACTAGCAACGCACTTT	72892	0.26116963091498585	No Hit
TGGGAGACCGCCTGGGAATACCGGGTGCTGTAGGCT	70431	0.2523519491161358	No Hit
TGAGGTAGTAGATTGTATAGTT	68975	0.24713514915712492	No Hit
TCTCCTACTTGGATAACTGTGGTAATTCTAGAGCTA	61018	0.2186254806998108	No Hit
ACAAATGATGAATAACAAAGGGACTTAATACTG	58374	0.2091521159390795	No Hit
GCAAATGATGATAAACTGGATCTGACTGACTGTGCT	56362	0.20194318632539143	No Hit
TAGCTTATCAGACTGATGTTG	55658	0.1994207775540016	No Hit
CTCGCTGCGATCTATTGAAAGTCAGCCCTCGACACA	54149	0.19401408034373552	No Hit
AATGGATTTTTGGAGCAGG	54074	0.1937453578183744	No Hit
ACGGCCCTGGCGGAGCGCTGAGAAGACGGTCGAACT	53690	0.1923694984885254	No Hit
CTAGACTGAAGCTCCTTGAGG	53182	0.19054935125007927	No Hit
TGCCTCTGATGAAGCCTGTGTTGGTAGGGACATCTG	52957	0.18974318367399587	No Hit
TTTGAATGATGACTTTAATTGTCGGATACCCCTTCA	52407	0.18777255182134753	No Hit
TGAGGTAGTAGTTTGTGCTGTT	52209	0.18706312435439412	No Hit
AGACGTGGCGACCCGCTGAATTT	50036	0.1792773370529308	No Hit

Sequence	Count	Percentage	Possible Source
TACCCTGTAGATCCGAATTTGT	49981	0.17908027386766598	No Hit
TAGCTTATCAGACTGATGTTGACT	48426	0.1735087601751784	No Hit
TTCAAGTAATCCAGGATAGGCT	44378	0.1590049097396867	No Hit
AGCGCTGAGAAGACGGTCGAACTTGACTATCT	42913	0.1537558630776325	No Hit
GACGTGGCGACCCGCTGAATTT	42849	0.15352655318932432	No Hit
GTGAAATGATGGCAAATCATCTTTCGGGACTGACCT	42266	0.1514376834255171	No Hit
GAGAAGACGGTCGAACTTGACTATCC	42254	0.15139468782145932	No Hit
CTGGATGATGATAAGCAAATGCTGACTGAACATGAA	41549	0.14886869608306463	No Hit
TAACACTGTCTGGTAACGATGTT	41411	0.14837424663640017	No Hit
TCAGTGCACTACAGAACTTTGT	41069	0.14714887192075338	No Hit
TAGCTTATCAGACTGATGTTGACA	40122	0.1437558021671934	No Hit
CGCGACCTCAGATCAGACGTGGCGACCCGCTGAATA	38890	0.13934158681726116	No Hit
GACGTGGCGACCCGCTGAATT	36845	0.13201441929241417	No Hit
TCGCGAAGGCCCGCGGCGGGTGTTGACGCGATGTGA	36463	0.1306457258965748	No Hit
CGGCCCTGGCGGAGCGCTGAGAAGACGGTCGAACTT	36252	0.1298897198585588	No Hit
AGACGTGGCGACCCGCTGAATT	36112	0.12938810447788468	No Hit
CTGCAGTGATGACTTTCTTAGGACACCTTTGGATTT	35805	0.12828813360740643	No Hit
ATATATGATGACTTAGCTTTTTTCCCCGAC	35594	0.12753212756939042	No Hit
TCCTACTTGGATAACTGTGGTAATTCTAGAGCTAAT	35194	0.12609894076746436	No Hit
TCGTACGACTCTTAGCGGTGGATCACTCGGCTCGTG	35119	0.12583021824210322	No Hit
ACTCCATGATGAACACAAAATGACAAGCATATGGCT	34903	0.12505629736906315	No Hit
CGCGACCTCAGATCAGACA	34733	0.12444719297824457	No Hit
CACAGATGATGAACTTATTGACGGGCGGACAGAAAC	34544	0.12377001221433451	No Hit
TGAAATGATGGCAAATCATCTTTCGGGACTGACCTG	34540	0.12375568034631526	No Hit
CTGAATGATGATATCCCACTAACTGAGCAGTCAGTA	34411	0.1232934776026941	No Hit
GCATTGGTGGTTCAGTGGTAGAATTCTCGCC	34256	0.12273811771694774	No Hit
GACTCTTAGCGGTGGATCACTCGGCTCGTGCGTCGA	33408	0.1196997616968645	No Hit
AACTGTGATGAAAGATTTGGTCTGTATGTAAT	33396	0.11965676609280672	No Hit
GAGAAGACGGTCGAACTTGACTATCA	31863	0.11416407767442512	No Hit
GCAGCCGACTTAGAACTGGTGCGGACCAGGGGAATC	31558	0.11307127273795649	No Hit
CTGACCTATGAATTGACAGCC	31518	0.11292795405776389	No Hit
GGGAGACCGCCTGGGAATACCGGGTGCTGTAGGCTT	31511	0.11290287328873017	No Hit
GCCGCCGGTGAAATACCACTACTCTGATCGTTTTTT	31392	0.11247650021515716	No Hit
TCCCTGGTGGTCTAGTGGTTAGGATTCGGCGCT	30716	0.11005441451990214	No Hit
GCAGCTGATGATACAGCTTCTTTCCCCATC	30060	0.1077039881647434	No Hit
CGACTCTTAGCGGTGGATCACTCGGCTCG	30033	0.10760724805561339	No Hit
GTCTACGGCCATACCACCCTGAACGCGCCCGATCTC	29020	0.10397770247973565	No Hit

Sequence	Count	Percentage	Possible Source
GAGAAGACGGTCGAACTTGACTATCTAGAGGAAGTA	28879	0.10347250413205669	No Hit

Adapter Content

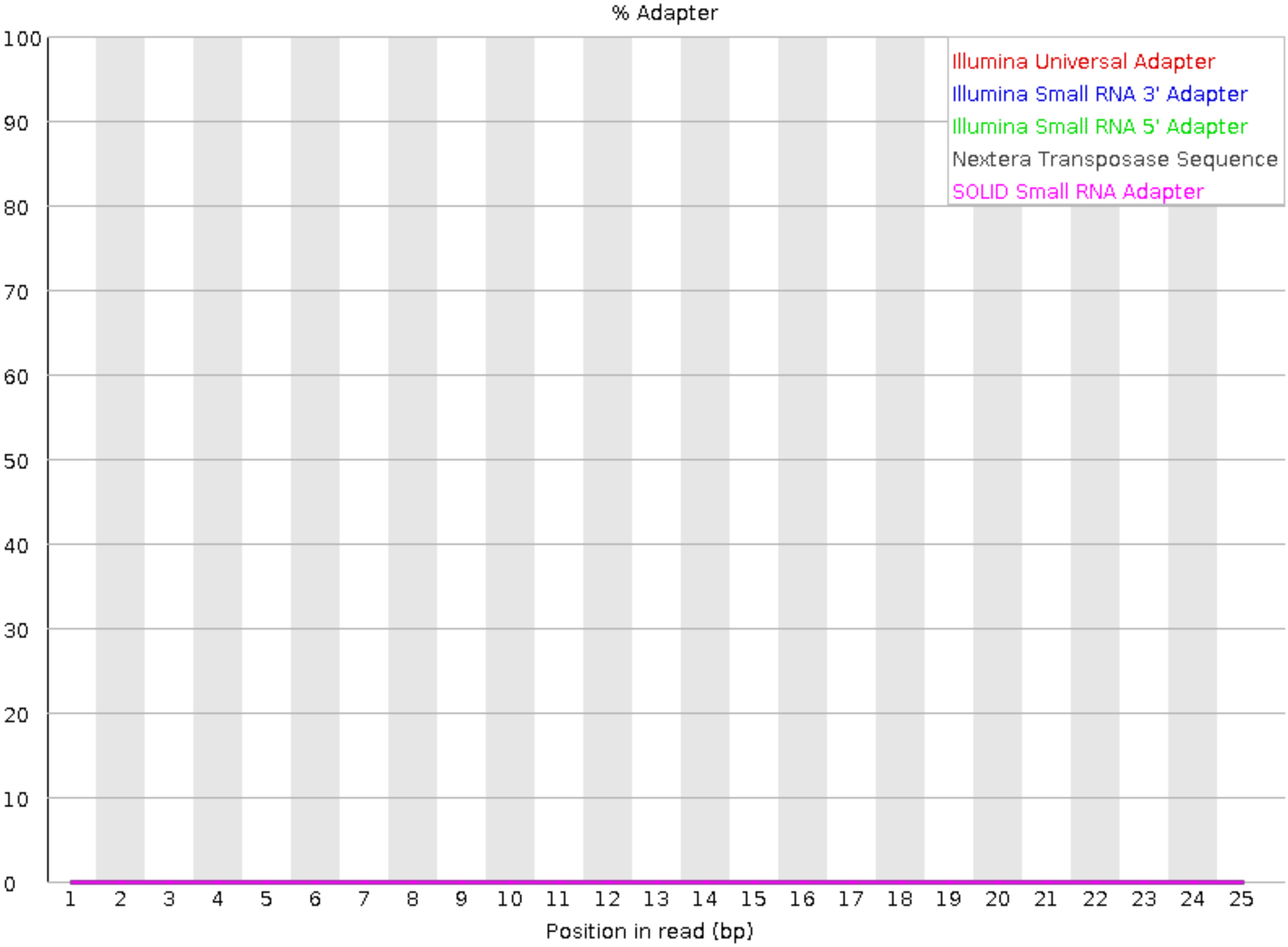

Produced by FastQC (version 0.11.9)
